# Genomic Confirmation of Hybridisation and Recent Inbreeding in a Vector-Isolated *Leishmania* Population

**DOI:** 10.1371/journal.pgen.1004092

**Published:** 2014-01-16

**Authors:** Matthew B. Rogers, Tim Downing, Barbara A. Smith, Hideo Imamura, Mandy Sanders, Milena Svobodova, Petr Volf, Matthew Berriman, James A. Cotton, Deborah F. Smith

**Affiliations:** 1 Wellcome Trust Sanger Institute, Wellcome Trust Genome Campus, Cambridge, United Kingdom; 2 Centre for Immunology and Infection, Department of Biology, University of York, York, United Kingdom; 3 Unit of Molecular Parasitology, Department of Parasitology, Institute of Tropical Medicine, Antwerp, Belgium; 4 Department of Parasitology, Fac. Sci., Charles University, Prague, Czech Republic; Imperial College London, United Kingdom

## Abstract

Although asexual reproduction via clonal propagation has been proposed as the principal reproductive mechanism across parasitic protozoa of the *Leishmania* genus, sexual recombination has long been suspected, based on hybrid marker profiles detected in field isolates from different geographical locations. The recent experimental demonstration of a sexual cycle in *Leishmania* within sand flies has confirmed the occurrence of hybridisation, but knowledge of the parasite life cycle in the wild still remains limited. Here, we use whole genome sequencing to investigate the frequency of sexual reproduction in *Leishmania*, by sequencing the genomes of 11 *Leishmania infantum* isolates from sand flies and 1 patient isolate in a focus of cutaneous leishmaniasis in the Çukurova province of southeast Turkey. This is the first genome-wide examination of a vector-isolated population of *Leishmania* parasites. A genome-wide pattern of patchy heterozygosity and SNP density was observed both within individual strains and across the whole group. Comparisons with other *Leishmania donovani* complex genome sequences suggest that these isolates are derived from a single cross of two diverse strains with subsequent recombination within the population. This interpretation is supported by a statistical model of the genomic variability for each strain compared to the *L. infantum* reference genome strain as well as genome-wide scans for recombination within the population. Further analysis of these heterozygous blocks indicates that the two parents were phylogenetically distinct. Patterns of linkage disequilibrium indicate that this population reproduced primarily clonally following the original hybridisation event, but that some recombination also occurred. This observation allowed us to estimate the relative rates of sexual and asexual reproduction within this population, to our knowledge the first quantitative estimate of these events during the *Leishmania* life cycle.

## Author Summary

Sexual reproduction is predicted to be a rare event in *Leishmania* parasites, as evidenced by detection of rare parasite hybrids in natural populations using molecular methods. Recently, a sexual cycle has been detected experimentally in parasites within the sand fly vector (that transmits this pathogenic microorganism to mammalian species including man, causing human leishmaniasis). In this study, we have used whole genome sequencing to investigate genetic variation at the highest level of resolution in *Leishmania* parasites isolated from sand flies in a defined focus of leishmaniasis in southeast Turkey. Using a range of analytical tools, we show that variation in these parasites arose following a single cross between two diverse strains and subsequent recombination between the progeny, despite mainly clonal reproduction in the parasite population. We have thus been able to derive quantitative estimates of the relative rates of sexual and asexual reproduction during the *Leishmania* life cycle for the first time, information that will be critical to our understanding of the epidemiology and evolution of this genus.

## Introduction

*Leishmania* parasites are among the most important vector-borne pathogens in the developing world, giving rise to a spectrum of diseases ranging from the dermal lesions of cutaneous leishmaniasis to the life-threatening organ failure of visceral leishmaniasis [1]. These protozoan organisms are transmitted between mammalian hosts (including man) as flagellated extracellular parasites within sand fly vectors [2]. In the host, *Leishmania* parasites live in professional phagocytic cells such as macrophages that can disseminate to different tissue sites, giving rise to the range of pathologies associated with parasite infection. Both parasite species and the immune response of the host are critical factors in determining the clinical presentation of the leishmaniases [3].

Despite the medical importance of these parasites, knowledge of their life-history in the wild, specifically the importance of a sexual cycle, remains largely unexplored. While asexual reproduction via clonal propagation has been proposed as the principal reproduction mechanism across the genus [4], [5], sexual recombination has long been suspected, based on hybrid marker profiles detected in field isolates from a range of geographical locations [6], [7], [8], [9], [10], [11], [12], [13]. These observations suggest that hybridisation can occur within and between a number of different species. Importantly, a sexual cycle has now been demonstrated experimentally [14]. This shows features consistent with a meiotic process and is detectable in vector-stages of *L. major*, with hybrids competent for transmission to the mammalian host by sand fly bite [14]. In a subsequent study, putative *L. donovani* hybrids have also been detected in the vector, early after infection [15].

While it thus seems certain that sexual reproduction occurs in natural populations of *Leishmania*, uncertainty remains over the exact role of this process in the epidemiology and evolution of these parasites. One consequence of recombination has been shown to be enhancement of transmission potential and fitness of hybrids in the vector: as one example, *Leishmania major*/*L. donovani* hybrids expressing *L. major* lipophosphoglycan as their major surface glycoconjugate have been shown to develop well in *Phlebotomus papatasi*, a sand fly species refractory for *L. donovani* [16]. The relatively low resolution of the markers used in *Leishmania* molecular typing to date, however, means that hybridisation events will be missed with existing tools unless they involve recombination between very different genetic backgrounds. The rate of out-crossing between different strains in the sand fly vector will depend on the frequency of co-infection, but the low levels of heterozygosity observed in *Leishmania* populations could be due to frequent sexual reproduction by selfing [17], [18], although other explanations are possible (reviewed in [10]). The experimental conditions in which successful crosses have been generated may be rather different to natural conditions but the rate of hybrid formation in these analyses seems to depend on the choice of parental species. In *L. major*, ∼26% of sand flies yielded hybrid progeny in crosses between two lines of *L. major* [14] while recovery was also consistently high with other *L. major* parental lines in the most recent study from the same team [19]. By contrast, only 0.3% sand flies contained recoverable drug-resistant hybrid parasites in the first study with *L. donovani* strains [15].

Here, we use whole genome sequencing to investigate the frequency and importance of sexual reproduction in *Leishmania*. This constitutes the first whole genome analysis of population diversity from vector-derived strains of the *L. donovani* complex. Our analysis is based on 12 strains of *L. infantum* that were isolated from a focus of cutaneous leishmaniasis (CL) in the Çukurova province of southeast Turkey as part of a large-scale survey of both vectors and patients in this region ([20], Figure S1, Table S1). There are no visceral leishmaniasis (VL) cases reported in this region. Unusually, 11 of these 12 CUK strains were isolated from sand flies (specifically *Phlebotomus tobbi*), while only a single isolate was of human origin, the only isolate recovered from a total of 128 samples obtained from patients with suspected CL. These strains were originally typed as *L. infantum* by PCR-RFLP [20]. *L. infantum* has been widely implicated as a causative agent of both VL and CL in the Mediterranean basin, with relatively low incidence of zoonotic VL interspersed with periodic focal outbreaks of human disease [21]. In contrast, *L. donovani* is often anthroponotic, causes VL, CL and post-kala-azar dermal leishmaniasis (PKDL) in different geographical regions, and is generally considered more aggressive than *L. infantum* [22], [23].

The Çukurova focus is geographically isolated, being bordered by the Taurus and Antitaurus mountains in the north-west, north and north-east, the Amanos mountains in the east and the Cukurova lowland in the south. At an altitude of 200–330 m above sea level and mean temperature of 18.5–20°C, land use in the focus is predominantly agricultural including cattle breeding. The human population, comprising different ethnic groups, typically live in single-family houses surrounded by gardens with hen houses and sheep or cattle sheds. Sleeping outside without bednets, living close to a barn and storage of dried dung (suitable as a food for sand fly larvae) are factors associated with significantly increased risk of *Leishmania* infection in the area [24]. Thousands of new CL cases have emerged in this region since 1985 and while it was originally suggested that these were due to *Leishmania tropica* infection, the causative agent has now been unequivocally identified as *L. infantum* transmitted by *Phlebotomus tobbi* [20].

Previous multi-locus sequence typing (MLST) of three of the CUK strains (including the human isolate) has revealed identical sequence at a panel of five loci. When compared to a panel of *L. donovani* complex strains, the CUK strains are most closely related to strain MHOM/IT/93/ISS800 (MON-188) isolated from an HIV-positive patient presenting with VL in Sicily [20]. Subsequent analysis by multi-locus enzyme electrophoresis (MLEE) has confirmed that the CUK strains form a novel zymodeme (MON-309) that is related to the MON-37 zymodeme of *L. donovani* from Cyprus [25]. More sensitive microsatellite typing methods (MLMT) have further confirmed designation of the Çukurova strains as part of a genetically-distinct monophyletic group of the *L. donovani* complex [25].

Here, we have exploited the high marker density provided by whole genome sequencing to investigate variation in the Çukurova *Leishmania* population at the highest level of resolution. A genome-wide pattern of patchy heterozygosity strongly suggests that these strains originated from a cross between two genetically distinct *L. donovani* complex species. We characterise the recombinational process that founded this population, investigate the potential parents of the original hybridisation and show that subsequent recombination has occurred among the descendants. Finally, the relative frequency of these subsequent recombination events, and of segregating polymorphisms within these strains, allow us to estimate the relative frequencies of sexual and asexual reproduction within this population. These results are the most direct evidence to date of sexual recombination in natural populations of *Leishmania* and we use the extensive diversity within the population to provide initial estimates of its relative frequency.

## Results

### Genome-wide patterns of polymorphism and divergence in the CUK population

Each CUK strain was sequenced to a mean depth of coverage of 49-fold (Table S2), covering 98% of the reference genome sequence. We identified 17,333 single-nucleotide variants (SNPs) segregating within the population, of which 2,747 were heterozygous in all 12 strains and 4,463 were unique to individual strains (Table S3). Recent genome analysis has shown low levels of heterozygosity within Old World *Leishmania* populations [26], [27] consistent with pervasive inbreeding [18]. This low heterozygosity was also observed genome-wide in the reference strains *L. infantum* JPCM5 [28] and *L. major* Friedlin [29] and in Indian *L. donovani* strains [18], [30], but the CUK strains show a much higher level of variability: the mean number of differences between strain pairs is 7,288 homozygous and 850 heterozygous SNPs per genome (Table S4). We also identify variation in chromosome copy number between strains (see below). This surprising diversity makes the CUK strains excellent candidates for investigating the genetics of a natural *Leishmania* population.

**Figure 1:**
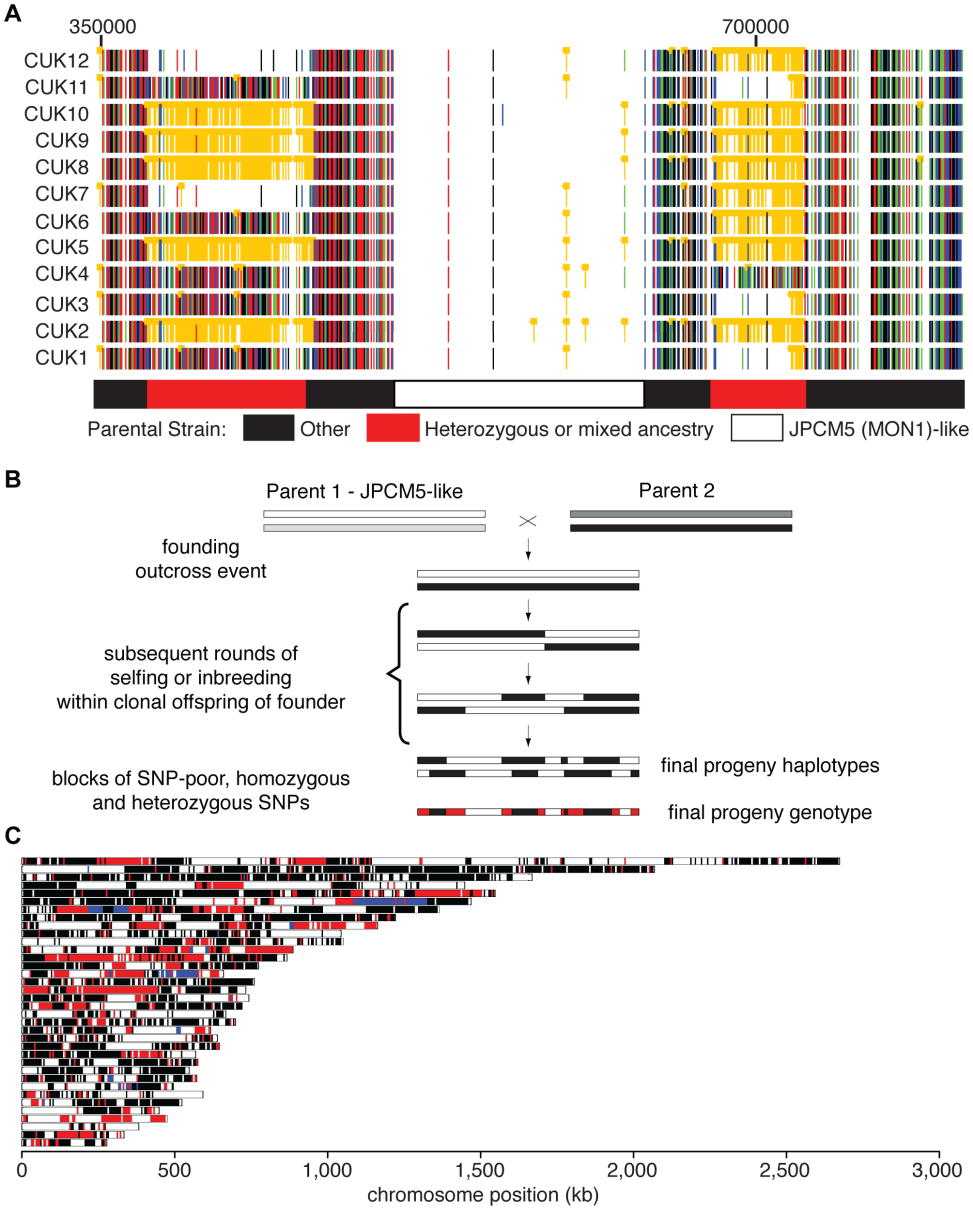
Genome-wide patterns of polymorphism and divergence show patchy heterozygosity symptomatic of hybrid ancestry. (A) Plot of single-nucleotide polymorphisms (SNPs) with respect to *L. infantum* JCPM5 in a section of chromosome 28 across the 12 strains. Orange ‘blocked’ bars indicate heterozygous positions; green, red, blue and black indicate homozygous variant calls of A, C, G and T respectively. Regions derived from one or both parental strains are shown in the block diagram below. CUK strain numbers (left) and chromosomal position (above, nucleotides) are as indicated. (B). Schematic of putative hybridization/inbreeding model, illustrating how a single hybridization event followed by crossing within the offspring could result in patchy heterozygosity in the progeny sampled here. (C) Parentage blocks inferred from mixture model predictions. White corresponds to regions that are similar to the JPCM5 parent (SNP sparse) in all isolates; black similar to the unknown parent (SNP dense) in all isolates; blue are heterozygous in all isolates. Red indicates regions that vary between isolates, and so are either SNP dense and SNP sparse in different isolates, or heterozygous in some isolates.

The Turkish CUK samples display considerable genetic divergence from the Spanish *L. infantum* and Nepalese *L. donovani* reference genomes, and from the Ethiopian *L. donovani* isolate LV9, confirming that these parasites form a single population genetically distinct from other sequenced *L. donovani* complex isolates (Figure S2). Neither the 41,142 fixed SNPs identified against the JPCM5 genome nor the 17,333 that varied among the 12 CUK strains were uniformly distributed across the genome (Figure 1A, Figure S3).

### Patchy heterozygosity symptomatic of hybrid ancestry

The SNP analyses described above identified both heterozygous sites and sites showing homozygous differences to the reference genome sequences, and these each generally occupied discrete genomic regions in the CUK strains (Figure 1). When viewed in comparison to *L. infantum* JPCM5 (the closest related reference sequence available [28]), the genomes of these strains had a mosaic structure with three major patterns. These were (i) SNP-poor regions (where both haplotypes were similar to the consensus sequence of JPCM5); (ii) those dense in homozygous SNPs but low in heterozygous SNPs (both haplotypes were different to JPCM5); (iii) loci dense in heterozygous SNPs but low in homozygous SNPs (where one haplotype was similar to JPCM5 and the other different). By integrating these data across the 12 strains, “parentage blocks” were defined as regions that vary between strains, but are clearly derived from an *L. infantum*-like parent (i.e. are SNP-poor regions) in some, but in others contain either dense homozygous SNPs or heterozygous SNPs (see Figure 1A for chromosome 28 as an example). Similar patterns can be seen on many other chromosomes (20 out of 36; Figure 1C), confirming that this is a genome-wide phenomenon (Figures S4, S5).

To further investigate the genetic make-up of the CUK strains, we modelled the density of heterozygous and homozygous SNPs compared to the JPCM5 reference genome using a mixture model of the number of independent patterns of homozygous and heterozygous SNPs (see Material and Methods for further details). This model confirmed that the presence of three independent components (three patterns of SNP density) explained the data significantly better than either a one- or a two-component model, with the three components representing the three categories of regions we identified visually. Adding additional components to the model resulted in more modest improvements in global model fit, and these additional components captured relatively small proportions of the genomes. This modelling thus allowed us to assign each 5 kb window along each genome into one of these two likely ancestries – the first originating from the JPCM5-like parent and the second from an unknown parent. Regions dense in heterozygous SNPs are due to hypothetical recombination events since the original hybridisation. Some windows (9.5% of the genome) could not be unambiguously assigned to any of the three components of the model using this method. Our mixture model predicts that between 54.2% and 53% of the hybrid genome comes from the JPCM5 ancestor, depending on the strain, but bootstrap estimates of the confidence interval for this estimate show that a null hypothesis of exactly 50% contribution from each parent cannot be strongly rejected by our data (p = 0.013; see Figure S6). Those regions predicted by this model to be SNP sparse (related to JPCM5), SNP dense (the unknown parent) or uniformly heterozygous (shared ancestry from both parents) across strains or mixed (heterozygous, SNP dense and SNP sparse) are shown across all *L. infantum* chromosomes (Figure 1C). These data are broadly robust for choices of window size between 1 kb and 50 kb, although the estimated ancestry from JPCM5 is higher, up to 60%, for small window sizes within this range (possibly because many of these small windows cannot be assigned with high probability to any model component).

Taken together, these results suggest that the CUK strains originated from a single out-crossing event between one parasite strain related to the JPCM5 *L. infantum* (MON-1) zymodeme and another unknown strain. Our conceptual model for the evolution of these strains shows how the ‘mosaic’ structure of the extant genomes evolved by limited recombination via inbreeding between the offspring strains (Figure 1B). While other contrived processes could explain these data, the most parsimonious explanation for the maintenance of an almost exactly 50∶50 contribution of the two parental genotypes in these strains is that recombination has occurred exclusively between the progeny of the original cross. In this context, the high heterozygosity (for many isolates, approximately half of all variants are heterozygous; Table S3) and large, widely-spaced blocks we identify, together with the divergence between isolates (Figure S2, Table S4), suggests that few recombination events have occurred since the original hybridisation event, a long enough period for a substantial number of mutations to accumulate in the population.

### Identifying the parental genomes of an ancestral hybridisation event

The low number of SNPs called in the low-density blocks in Figure 1(A,C) is consistent with one of the parental genotypes being closely related to *L. infantum* JPCM5, but the identity of the other parent in the original cross is uncertain. The kinetoplast has been proposed to be uniparentally inherited in *Leishmania* [14], allowing analysis of sequence data from one parent without the complication of recombination in the nuclear DNA. Analysis of the constant region of the kDNA of the CUK strains indicated strong similarity to the *L. infantum* JPCM5 genome (Figure S7).

**Figure 2:**
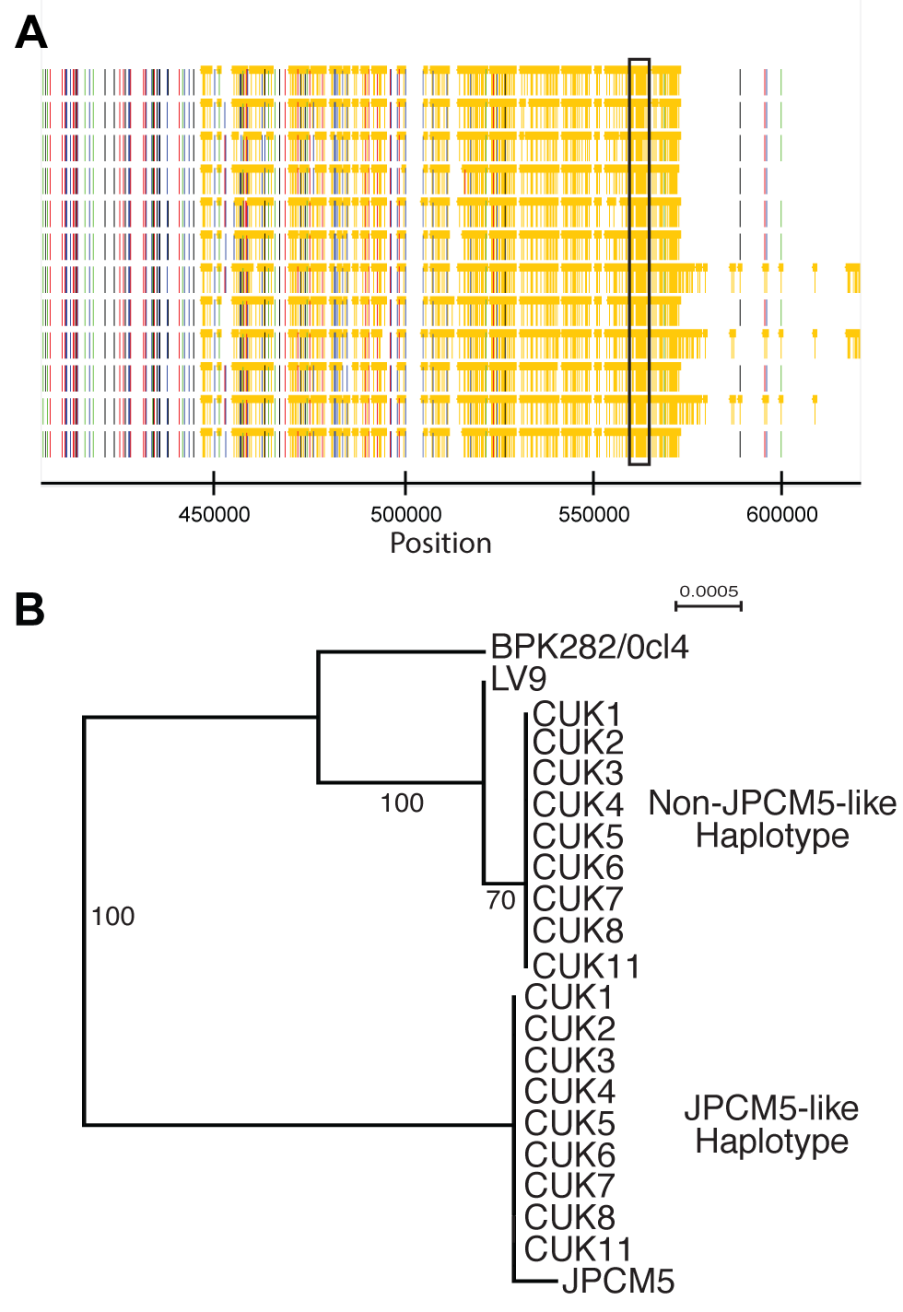
Phased haplotypes help identify one of the parental strains. (A) A heterozygous SNP-dense region of chromosomes 22 (boxed) was one of 86 regions that could be successfully phased to infer haplotypes of at least 5 kb in at least one CUK strain. This region could be phased in 9 strains. (B) Phylogenetic analysis of this region confirms that the two haplotypes have different evolutionary histories: one related to *L. infantum* JPCM5 and the other distantly related to the two *L. donovani* reference genomes.

While the identity of the parental strain seems clear in the SNP-poor regions of the genome, we attempted to confirm that the heterozygous regions of the genome represented mixed ancestry, with one ancestor represented by the expected parental genotype related to JPCM5. This was done by reconstructing haplotypes through phasing the diploid genotype calls. The inference of extended haplotypes using phasing was restricted to only regions that displayed sufficient levels of heterozygosity. Phased haplotypes were investigated for 86 regions where the lengths were at least 5 kb in one of the CUK samples (Figure S8). Of these, 76.7% (66/86) segregated the 12 samples into two clades, one similar to JPCM5 and the other phylogenetically distinct (see Figure 2 for an example on chromosome 22). The inferred haplotypes for the CUK strains were not closely related to other sequenced members of the *L. donovani* complex (BPK282/0cl4 and LV9) [26].

**Figure 3:**
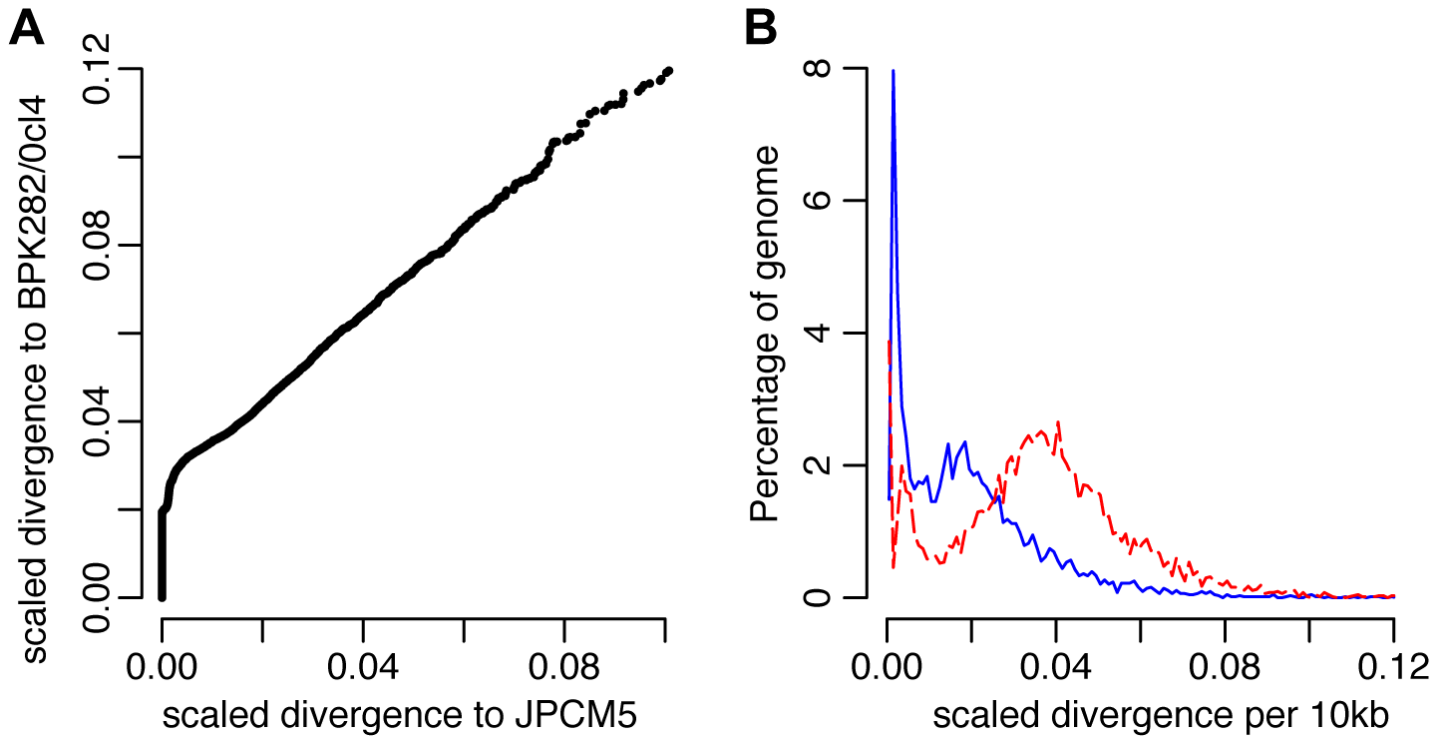
Genome-wide divergence between the CUK strains and other *L. donovani* complex reference genomes. (A) Divergence between the Turkish population and the *L. donovani* BPK282/0cl4 and *L. infantum* JPCM5 reference genomes. Values shown are numbers of SNPs in non-overlapping 10 kb windows, scaled using the divergence to *L. major* for each window. A higher fraction of blocks show no more difference between the Turkish population and JPCM5 than to BPK282/0cl4, but other blocks are significantly divergent from both references. (B) Frequency distribution of divergence between the CUK strains and the reference genomes, *L. infantum* JPCM5 (blue solid line) and *L. donovani* BPK282/0cl4 (red dashed line) for 10 kb windows, scaled using the divergence with *L. major*. Divergence to JPCM5 shows a significantly bimodal pattern, with sets of very low divergence blocks (mode 0.1 SNPs/kb) and higher divergence (mode 1.7 SNPs/kb), while BPK282/0cl4 divergence cannot reject unimodal high divergence (2.0 SNPs/kb). Genome-wide patterns suggest one ancestor closely related to JPCM5 and a second ancestor not closely related to either of the two references.

A direct assessment of genome-wide variation between the CUK strains and the two *L. donovani* complex reference genomes (JPCM5 from Spain and BPK282/0cl4 from Nepal) confirmed the presence of one JPCM5-like parent and a second genetically distinct parent. For JPCM5, this analysis revealed a patchy distribution of variants, in which 18% of the 3,260 10 kb windows were entirely SNP-free when called against this reference. In contrast, the same analysis for SNPs called against *L. donovani* BPK282/0cl4 showed that just 1% of windows contained no variants. This pattern persisted after scaling for ancestral rates of substitution using *L. major* (Figure 3), demonstrating that a significant portion of each CUK genome was identical to the Spanish reference but not to the Nepalese one. The shape of the distribution of (scaled) nucleotide divergence across windows of the genome for these two *L. donovani* complex reference sequences and the more divergent *L. major* to the Turkish strains verified this pattern. The distribution of divergence to *L. donovani* showed a unimodal distribution (AIC = −3891.5 vs −3808.0 for bimodal) with mode at 1.8 SNPs/kb, and divergence to *L. major* also showed a unimodal distribution (AIC −2886.5 vs −2821.9; Figure 3), while a bimodal distribution was better supported for *L. infantum* (AIC −3852.5 vs −3872.5) with peaks at 0.1/kb and 1.7/kb. These genome-wide statistics supported the pattern seen in the phased regions – with one of the CUK strains closely related to JPCM5, and a second approximately equally divergent from both JPCM5 and BPK282/0cl4. We have not yet identified the second parent of the CUK strains with confidence; a much wider screen of related strains would be required for this analysis and these are not currently available.

### Intraspecific linkage patterns show low levels of recent recombination

Despite their putative ancestry derived from a single out-crossing event, the 12 strains in this study demonstrated a higher level of nucleotide diversity (π = 4.87 per Mb, Watterson's θ = 11.48 per Mb) than the only previous study of population genomic variation in *Leishmania* [31] but this varied among chromosomes (Figures S9, S10). Inspection of genome-wide 10 kb windows of different strains confirmed the mosaic structure. While many blocks were conserved across all of the strains, there were many examples of clear recent recombination within the population with only short haplotypes shared between strains, and of blocks where an extended region of predicted heterozygous SNPs in some isolates were represented by homozygous equivalents of the two alleles in some of the other isolates (Figure 1A; compare for example CUK2, 5, 8–10 with CUK 1,3,4,6,11 in left-hand region of chromosome 28). These signals confirmed that recombination has occurred within the CUK population, but the extended nature of the parental blocks suggests that this may have been an infrequent event. We explored this observation further by performing the first genome wide examination of linkage between SNPs in *Leishmania*.

Levels of recombination were inferred using the linkage between pairs of mutations (see Methods). The relative correlation between SNPs across the population within (mean r^2^ = 0.63±0.41; Figure S11) and between (mean r^2^ = 0.58±0.44) chromosomes differed only slightly, indicating that linkage persisted across the entire genome and suggesting that events of recombination have occurred only rarely in this population (Figure S12). Certain blocks within chromosomes 4, 22, 25, 28, 30 and 31 had higher linkage levels with blocks on different chromosomes than with those on the same chromosome (Figure S13), symptomatic of a few recent recombination events. This population-level mixing was examined by evaluating the decay of linkage disequilibrium (LD) between SNP pairs with distance for each chromosome (Figure S14). LD decay was distinctly higher for these six chromosomes and this significantly disrupted the ancestral haplotype blocks, so that these segments tended to be of undetermined ancestry according to the ancestry assignment model, and showed high nucleotide diversity. This interpretation was supported by LD patterns between each 10 kb segment pair both within (Figure S13) and between (Figure S12) chromosomes.

### Read-depth coverage frequencies pinpoint major recombination breakpoints

**Figure 4:**
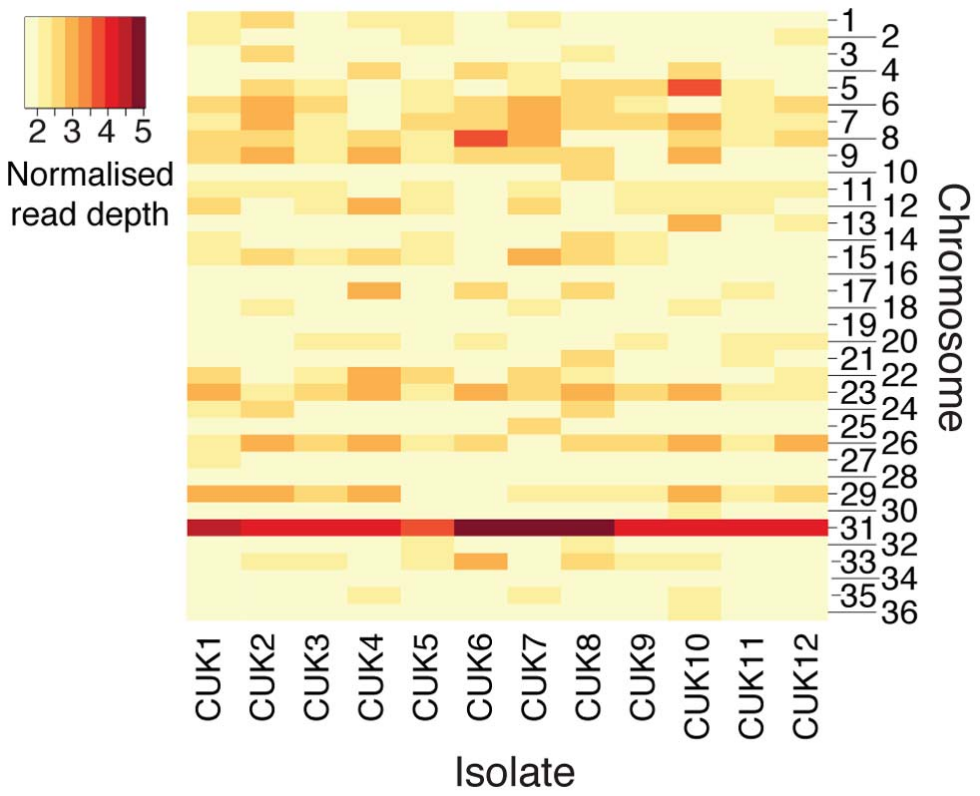
Aneuploidy in the CUK strains. The heatmap shows chromosomal copy number for all 36 chromosomes across the 12 CUK strains, based on normalized chromosome read-depth. Copy number varies from disomic (light yellow) to pentasomic (dark-red).

Aneuploidy has previously been shown to be a common phenomenon within *Leishmania* genomes [27], [32] and this is also observed in the CUK population (Figure 4). Here, aneuploidy is highest for chromosome 31, which was pentasomic for three of the 12 strains analysed (CUK6, 7 and 8); this chromosome has previously been shown to be tetrasomic in the *L. donovani* complex [26], [27]. In this study, only 6 out of the 36 chromosomes analysed were uniformly diploid among all CUK strains (3, 16, 19, 28, 30 and 34; Figure 4). Only the latter four chromosomes listed were uniformly disomic in 17 *L. donovani* clinical strains [26]. This trend indicates that some aneuploidy at every chromosome is the norm rather than the exception for natural *L. donovani* complex strains, and this may apply across the genus [33], [34].

We exploited this novel aspect of *Leishmania* biology to further resolve recombination breakpoints at loci with sufficient levels of heterozygosity. Plots of allele frequencies were used as an alternative approach to predict which chromosomes were trisomic or tetrasomic in each strain (Figure S15). After verifying that the median read depth of trisomic chromosomes was 1.5-fold higher than the median disomic read depth across the strains, we deduced their haplotypes from the distribution of allele frequencies for each heterozygous SNP [17] on chromosomes with at least 100 heterozygous sites (chromosomes 2, 4, 6, 15, 22, 23, 24, 25, 31, 33). Considering the frequency of JPCM5-like alleles for a trisomic chromosome, frequencies distributed with modes of 0.33 and 0.67 should be observed (in contrast to those for disomic chromosomes with a single mode of 0.5). Shifts in the read-depth allele frequency distribution between these two modes along chromosomes should represent recombination events within the population [17]. This approach will miss some recombination events: an allele frequency change from 0.33 to 0.67 present in the CUK set but absent in the JPCM5 reference will represent a breakpoint. However, for a major allele at a frequency of 0.67, recombination events could have occurred between chromosomes carrying this allele that would not be visible in any change of allele frequency.

**Figure 5:**
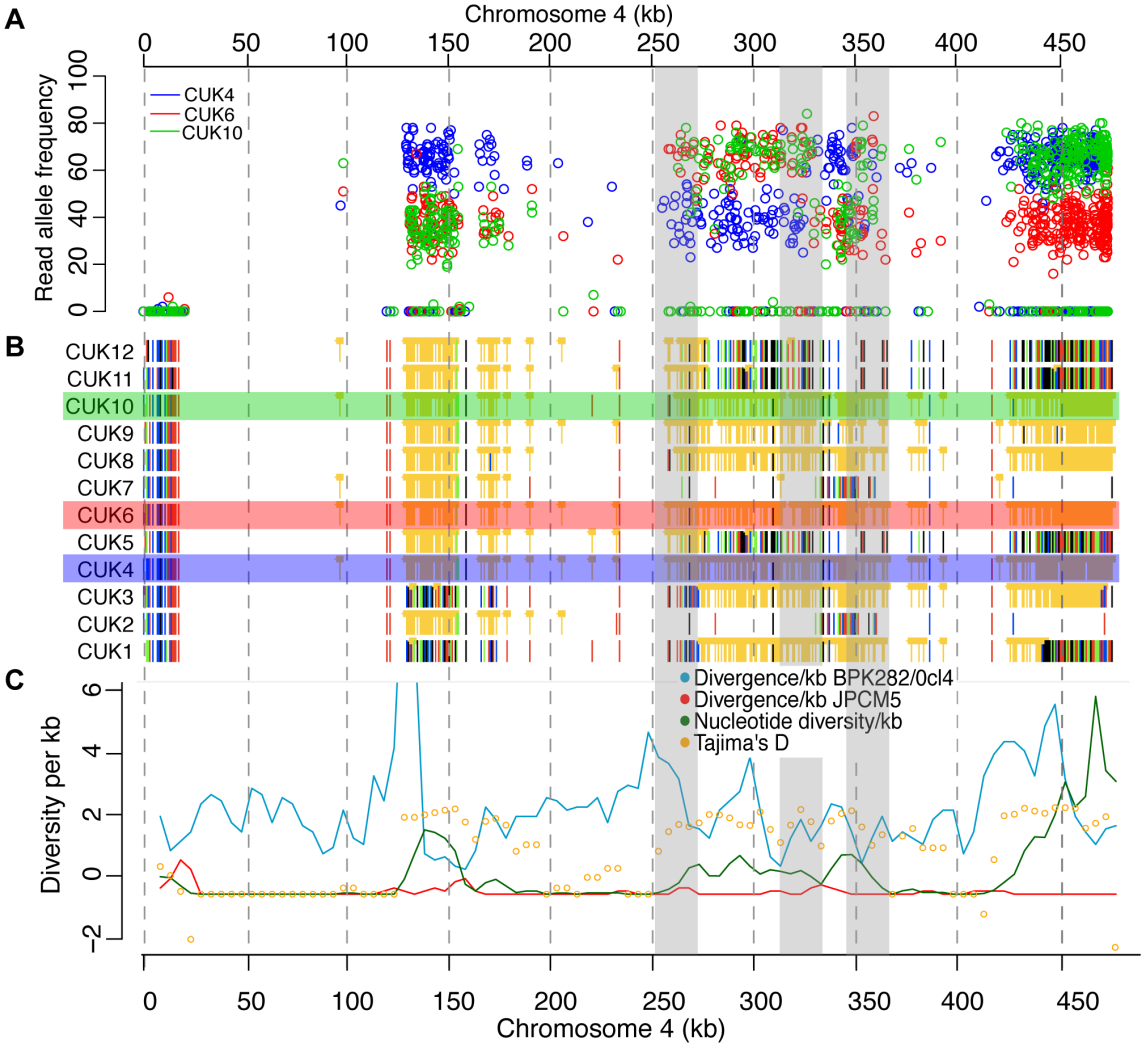
Changes in SNP coverage depth pinpoint recombination breakpoints. Chromosome 4 is trisomic in three of the strains, so changes in read depth for derived (non JPCM5-reference) alleles (A) show changes within some otherwise unphased blocks of heterozygous SNPs (B), showing recombination breakpoints not visible from simple plots of variant calls and not identifiable by other summary statistics (C). (A) shows derived allele read depth for the three trisomic strains, where changes in read depth inside the shaded boxes indicate presumed recombination events, where allele calls change from being present in 1/3 or 2/3 homologous chromosome copies. (B) Single-nucleotide polymorphisms (SNPs) with respect to *L. infantum* JCPM5 in a section of chromosome 4 across the 12 strains. Orange ‘blocked’ bars indicate heterozygous positions; green, red, blue and black indicate homozygous variant calls of A, C, G and T respectively. (C) Summary statistics for variant calls in 5 kb windows.

Samples with trisomy at chromosomes 4, 6, 15, 22–25, 31–33 (Figure S15) showed tightly clustered allele frequencies for the alleles shared with JPCM5, suggesting close linkage between the distinct heterozygous regions on these chromosomes. This observation was supported by step-wise LD patterns across chromosomes, and also by the decay of linkage with distance (Figure S14). Conversely, chromosomes 22, 23 and 32 (Figure S15) had allele frequency distributions at heterozygous regions suggestive of recombination. Chromosomes 4, 24, 25, 31 and 33 also displayed sudden and extended shifts in the read-depth allele frequency at likely recombination breakpoints (Figure S15) supported by step-wise LD patterns across chromosomes, and the decay of LD with distance (Figure S14). Shifts in read-depth allele frequencies common to several strains were clearly also visible in genome-wide statistics for the population: for example, at chromosome 31 a change in frequency at position 1,081,293 from 0.5 to 0.25 and 0.75 (or 0.2 and 0.8 for pentasomy) was matched by high divergence to JPCM5, positive Tajima's D values and low LD. We identified some breakpoints within extended blocks of homozygous SNPs at trisomic and tetrasomic chromosomes that were not evident in population-wide analyses of linkage, for example on chromosome 4 at 325 kb in CUK6 (Figure 5), confirming that this approach can identify individual recombination events missed by other statistics. However, it should be noted that this method will only be able to identify recombination on chromosomes with high somy levels, and will miss many events where somy levels are even. Hence, this does not allow systematic identification of recombination events occurring across the genome.

### Mitotic and meiotic cell division during the *Leishmania* life cycle

Each eukaryotic cell division introduces additional mutations into the genome, but crossing-over events occur principally during meiotic events. While evidence of meiotic crossing-over has been reported in *Leishmania* [14], [19], there is no evidence that this is particularly common in the field. Genomic data from a discrete population, together with estimates of the number of mutations or recombination events introduced by each cell division event, can thus give insight into the relative numbers of mitotic and meiotic cell divisions during the recent history of a population. Such data provide quantitative information about the relative importance of sexual and asexual reproduction in the parasite life cycle that are hard to obtain using other means. To estimate the relative rates of meiotic and mitotic cell division in the CUK *Leishmania* population, we derived independent estimates of the effective population size (*N_e_*) based on observed levels of recombination and mutation (nucleotide diversity) in the CUK samples (see Methods). Using comparative data from a range of eukaryotic species (see Figure S16), we estimate the genome-wide mutation rate in *Leishmania* to be around 1.99×10^−9^ per bp per generation (best-fit regression model μ = 0.043*x* ^−1.310^ for genome size *x* in Mb, 95% prediction interval, 5.16×10^−10^–7.64×10^−9^). We also estimate the per-generation number of crossing-over events at 1 per 2.3 Mb (best-fit model *r* = 2.586×10^−10^ *x* ^0.584^, 95% prediction interval 0.00019–0.00104 per kb). Our genome-wide mean estimate of the inbreeding coefficient, F, is 0.884, a similar figure to that derived from other *L. donovani* complex populations [25], [35], [36], [37], [38].

We attempted to estimate nucleotide diversity and recombination statistics for the 23 chromosomes for which somy was invariant across the CUK samples (Table 1) but the MCMC (Markov Chain Monte Carlo) estimates of the recombination parameter ρ did not converge in at least four of five replicate runs for a number of the larger chromosomes, presumably due to the difficulty in reliably estimating haplotype phase over long genetic distances from a relatively small sample of segregating sites. A larger collection of samples would improve this analysis. Estimates of recombinational and mutational effective population sizes are broadly consistent across the 14 chromosomes for which estimates of ρ are available, with the exception of chromosome 3 for which nucleotide diversity (π) in our sample was conspicuously lower than for other chromosomes. Our estimates of the effective population size for all other chromosomes are between 10^4^ and 10^5^. This value is significantly smaller than that previously estimated for *L. donovani* [39] because it reflects the single focus under investigation here, rather than being based on data from across the geographic range of the species [40], [41]. In any case, the previously published effective population size is probably an overestimate, given the significant population structure within the *L. donovani* complex. Recombination effective population sizes are all very small, reflecting the rarity of crossing-over within the population. For the 13 chromosomes analysed, we estimate that between 1.5×10^4^ and 1.82×10^5^ (mean 7.5×10^4^) mitotic cell divisions occur for every meiotic event. The figures are similar if we restrict this analysis to those chromosomes that are uniformly diploid across the population, suggesting that polysomy is not having a major effect in biasing these estimates.

**Table 1:** Mutation and recombination diversity give two different estimates of effective population size of the Çukurova population.

Chromosome	Length (Kb)	# variable sites included	Θ per Kb	Posterior median ρ	95% HPD CI for ρ	N_e_(θ)	N_e_(ρ)	Ratio N_e_ρ/N_e_θ
**1**	269	138	0.089	0.000745	(0.000438,0.001121)	1.82×10^5^	3.73	4.88×10^4^
**2**	336	121	0.039	0.000316	(0.000222,0.000440)	7.99×10^4^	1.55	5.16×10^4^
**3**	384	83	0.029	0.000524	(0.000151,0.001514)	5.94×10^4^	3.71	1.60×10^4^
**4**	473	17	0.021	0.000531	(0.000152,0.001063)	4.30×10^4^	2.85	1.51×10^4^
**10**	571	515	0.066	0.000343	(0.000209,0.000513)	1.35×10^5^	1.71	7.90×10^4^
**11**	583	565	0.066	0.000373	(0.000229,0.000556)	1.35×10^5^	1.87	7.23×10^4^
**12**	675	666	0.057	0.000387	(0.000234,0.000557)	1.17×10^5^	1.93	6.05×10^4^
**14**	623	525	0.067	0.000188	(0.000112,0.000369)	1.35×10^5^	1.01	1.36×10^5^
**15**	630	375	0.05	0.000154	(0.000066,0.000368)	1.02×10^5^	0.86	1.19×10^5^
**16**	715	537	0.059	0.000206	(0.000120,0.000367)	1.21×10^5^	1.03	1.17×10^5^
**17**	685	292	0.032	0.000270	(0.000124,0.000501)	6.55×10^4^	1.42	4.62×10^4^
**19**	702	630	0.053	0.000142	(0.000094,0.000197)	1.09×10^5^	0.70	1.55×10^5^
**21**	773	738	0.115	0.000264	(0.000196,0.000344)	2.36×10^5^	1.30	1.82×10^5^
**27**	1,130	921	0.075	0.000240	(0.000170,0.000330)	1.54×10^5^	1.19	1.29×10^5^

Estimates of mutation and recombination diversity per chromosome, effective population size estimates, and the estimated ratio of meiosis to mitosis in the CUK population. Entries are for those chromosomes that show stable disomy across our sample collection, and for which PHASE estimates of the recombination parameter ρ converged in at least four out of five MCMC estimates.

## Discussion

Our data show that a population sampled at a single focus (Çukurova, Turkey) are descended from a hybrid strain of *Leishmania*, produced from a single outcrossing between one parent related to *L. infantum* JPCM5 (derived from Spain) and a second as yet unidentified parent most likely from the *L. donovani* complex. Subsequent inbreeding or selfing has produced a patchy distribution of homozygous and heterozygous variation with respect to JPCM5, producing a genome-wide mosaic of ancestry from the two parents. We hypothesize that these regions remain from the outcross between two genetically distinct *Leishmania*. Haplotype inference at regions with sufficient heterozygosity shows that linkage within these blocks is high and that they produce phylogenetic profiles consistent with one of the parents being an *L. infantum* JPCM5-like isolate and the other of unknown origin. We demonstrate that recent recombination within the CUK population subsequent to the hybrid cross is present although at a low level. Some of these recombination events have only been revealed using a novel approach that exploits the aneuploidy of *Leishmania* to phase regions of chromosomes. This method is limited to multisomic chromosomes, however, and may underestimate recombination occurring between pairs of similar homologous chromosomes (e.g. recombination between two homologous chromosomes from a set of three that show similar SNP blocks).

The CUK strains studied here represent the first genome-wide analysis of *Leishmania* strains isolated from sand flies in the wild. While only CUK1 is derived from a human patient, this isolate is otherwise highly similar to the sand fly isolates, indicating that the population under study is transmissible to man and the causative agent of clinical leishmaniasis in this region. The opportunity for this study of *L. donovani* complex strains from the vector (in this case, *Phlebotomus tobbi*; Table S1) arose from the availability of multiple isolates collected from infected sand flies in a single disease focus. Relatively few sand fly strains have been isolated and identified with this level of rigour. An exception is the isoenzyme analysis of 21 *L. infantum* strains collected from sand flies in Southern Spain which showed a wider range of *L. infantum* zymodemes (4 in total) than in isolates collected from the vertebrate host (dog and human) in the same region [28], [42].

The higher levels of vector-stage nucleotide diversity detected in the current study as compared with that found in other *Leishmania* populations tested to date might reflect their biological origin in the sand fly, in which sexual recombination has been shown to occur [14], [15], although the similar high diversity in CUK1 does not support this hypothesis. Alternatively, the same variety of parasite strains may be circulating in both vector and man in disease foci but sampling methods may favour selection of pathogenic strains in man as compared to all circulating strains in vector populations. In addition, the possibility of independent transmission cycles involving vertebrates other than man cannot be discounted. Further analyses of larger populations will be necessary to confirm these hypotheses although our current data highlight the considerable genetic diversity found in parasites circulating in sand flies caught in the same location on the same night (e.g. chromosome 28 of CUK 4, 6, 7; Table S1, Figure 1A). The relevance of these observations to the transmission of *L. infantum* in the Çukurova focus, where dogs appear to play a role in the transmission cycle [24] in spite of earlier observations indicating an anthroponotic mode of transmission for these CL-causing parasites [20], remains to be determined. However, we hypothesise that this focus was invaded by a unique hybrid strain with low virulence for humans, which nonetheless transmitted very well due to specific factors (sleeping outside, low virulence of parasites, relatively many people infected, possibly also involvement of a different vector, *P. tobbi*). Further studies on the epidemiology of CL in this region are in progress.

While our data show clear signs of the hybrid ancestry of these strains, we note that this pattern is only obvious because the cross was recent and between two divergent genetic backgrounds. Crosses between more similar genotypes would leave a weaker signal when contrasting SNP-rich and SNP-poor regions, making these events difficult to detect without the availability of genomic data for strains closely related to both parents. The recent origin of the hybrid CUK population is important, because insufficient time has passed since the initial outcrossing for recombination to break down the haplotype blocks observed in this study: recombination will eventually produce blocks too small to distinguish from background variation, so this signal of hybridisation will only be visible for a limited time following the founding of the population. Indeed, the size of haplotype blocks visible in a genome has been used in human genetic analyses to date particular admixture events [43] and quantify the contribution of different ancestries to present-day populations. However, these quantitative models require knowledge of putative parental population genotypes and estimates of per-generation recombination rates, while assuming diploid, panmictic, obligately sexual populations. Given these factors, their application to the analysis of *Leishmania* species is challenging.

Other data are not helpful in identifying the ‘missing’ parent of these strains. Previously published Bayesian STRUCTURE analysis, based on MLMT data that included 3 of the CUK samples analysed here, implicated a relationship with a population of *L. infantum* from Cyprus that gives rise to both visceral and cutaneous disease and more distantly, with a population of ‘non MON-1’ strains from the Western Mediterranean [25]. Phylogenetic analysis of MLMT patterns suggests that these isolates are all part of a diverse *L. infantum* group, so we speculate that these analyses may reflect the ‘JPCM5’ ancestry we identify here. Similarly, the MLEE profile for the CUK strains shows affinity to a zymodeme shared by the Cypriot isolates above and by *L. donovani* strains from India and Kenya (MON-37), which are similar to MON-3, a zymodeme exemplified by an *L. donovani* from Iraq and other zymodemes from East Africa [44] that appear distinct by MLMT analysis [13]. While our deep DNA sequencing approach provides a high density of genetic markers and so should overcome the low resolution and potential homoplasy associated with MLEE typing [45], it is clear that denser sampling of the *L. donovani* species genomes will help resolve the identity of the unknown ancestor of the CUK strains. It is notable that previous MLMT and MLEE typing failed to establish the hybrid origin of this population.

Interestingly, Gouzelou *et al.* [25] did identify a hybrid strain of VL-causing *L. donovani* (EP59) from the Kusadasi province of Turkey. Analysis of MLMT markers suggested that this strain may be a hybrid of the Turkish/Cypriot (TR/CY) strains and MON-1, potentially making this the descendant of an outcross of a recent hybridisation event. Genomic characterization of this strain may shed some light on the complex ancestry of *Leishmania* parasites in Turkey. The presence of two hybrid populations in close geographic proximity, in contrast to the genetic homogeneity observed elsewhere [26], [27], reflects Turkey's geographical position as a crossroads between the Mediterranean populations of *L. infantum* and the *L. donovani* strains from East Africa and the Indian sub-continent. Consequently, this region may harbour *Leishmania* of particularly complex genetic ancestry, mirroring human trends. The particular features of endemic leishmaniasis, episodic human migration patterns within Turkey and partial MLMT profile similarity to the CUK strains [25] present Cyprus as one possible location for the unknown ancestor.

Our population genetic approach gives the first estimates (to our knowledge) of the relative importance of sexual and asexual reproduction in the *Leishmania* life cycle in a non-lab-adapted parasite population. By necessity, these estimates are approximate given the number of caveats to this analysis. The most obvious is the absence of direct estimates of mutation or crossing-over rates, so we have relied on comparative data to obtain these values with a confidence interval of around one order of magnitude in each parameter. More precise estimates could be obtained directly via a mutation accumulation experiment or through genomic analysis of progeny from a genetic cross but such data are not currently available in the literature or public domain. More precise estimates of the other parameters (θ, ρ and *F*) could be obtained by sequencing a larger sample of individual genotypes from a population. Estimating the relative rates of recombinational and mutational cell division requires some assumptions about the pattern of crossing-over within the population – effectively, that recombination occurs as would be expected in normal meiotic recombination. The pattern that we observe is entirely consistent with that expected from meiosis but we cannot exclude the involvement of other processes, such as mitotic recombination [46]. However, data from *Saccharomyces cerevisiae* (in which this process is best understood) show a different pattern to that which we observed in this study [47]. It is clear that *Leishmania* parasites show an unusual level of aneuploidy and we see somy variation within this set of isolates. The cause of this variation is unclear, and indeed some of the observed variation in this and other studies could be generated in vitro [32]. Somy level affects the two N_e_ estimates similarly, so the ratio we calculate should be unaffected by differences in somy *per se*, but the effect of dynamic somy on this estimate will depend on the precise pattern of variation through time. The extent and impact of these unusual features on the analyses we present is uncertain, and in this context, it is encouraging, but perhaps coincidental, that our estimate (equivalent to 1.3×10^−5^ meioses per mitosis) is close to the experimental estimate of the frequency of mating in a co-infected sand fly (∼2.5×10^−5^; from [14]).

The growing number of reports of potentially hybrid *Leishmania* parasites, together with the analyses presented here, support the view that sexual recombination, while probably rare in the field, may play an important role in the evolution of the genus, helping to drive the expansion of vector and reservoir range and the clinical presentation of disease in new foci. While our data cannot directly demonstrate the mechanism of crossing-over in *Leishmania*, we consider the presence of apparently normal meiotic recombination as a null hypothesis, and eagerly await new data – for example from analysis of experimental crosses [14] – that we expect will shed new light on this issue. We note that African trypanosomes appear to undergo normal meiotic recombination [48], [49] but the mechanism in American trypanosomes appears to be different [50]. Wider genomic investigation of populations from different disease foci, including other putative hybrid populations, will be needed to establish the generality of these findings. It is clear that genome analyses will provide essential information for unravelling the genetics, epidemiology and evolution of *Leishmania* species, just as they have revolutionised the study of *Plasmodium falciparum* (causative agent of cerebral malaria [51]), methicillin-resistant *Staphylococcus aureus* [52] and other pathogenic microorganisms.

## Materials and Methods

### Sample collection and DNA preparation

The CUK isolates were collected from sand flies and a single human patient in the Çukurova province of Turkey as described [20]. For the sand fly isolates, 1130 female flies were collected and dissected over 3 seasons (2005–2007); 13 of these flies (1.1%) had *Leishmania* promastigotes in their midguts. Parasites from individual midguts were cultured (with no pooling) resulting in 11 independent isolates i.e. a success rate of 85%. For the human isolate, biopsies were obtained for culture from 128 individuals with putative CL. Promastigotes grew in six of these cultures but thrived in only one (CUK1, as described in [20]). There are no cases of visceral disease in the Çukurova region ([20], [24], see Figure S1). This area has been described as a single focus, with eastern and western parts, because the disease presentation and the vector are the same throughout. The distance between the most westerly (Otluk) and most easterly (Boyali) villages where isolates were collected is 77 km (by air). The origin, location and date of isolation of these CUK strains are provided in Table S1.

After parasite cryopreservation, samples were shipped to York, thawed and promastigotes cultured at 26°C in modified Eagle's medium (HOMEM) supplemented with 10% heat-inactivated foetal calf serum (Invitrogen) and penicillin-streptomycin (Invitrogen). All isolates, which were not cloned before use, were grown for the minimum time necessary (10 days from first culture inoculation for CUK 1–8; 13 days for CUK 9–12) to produce adequate parasite numbers for isolation of genomic DNA for library preparation.

### DNA sequencing, variant screening and discovery

DNA library preparation, sequencing, quality control and variant discovery was carried out as outlined previously [26], [27] with several significant adjustments to improve quality. 76 bp paired-end reads for each sample were produced as a tagged pool of 12 on the same lane to minimise run- and lane-specific variation using the Illumina Hiseq 2000 platform. ENA accession numbers for the sequence data generated in this project are listed in Table S1, or can be accessed via study accession number ERP000588. These reads were mapped to the reference genomes with Smalt v0.6.1 (www.sanger.ac.uk/resources/software/smalt/) including small contigs to prevent non-chromosomal reads being aligned to chromosomes. The reads were mapped to both the *L. infantum* JPCM5 (MCAN/ES/98/LLM724) and *L. donovani* BPK282/0cl4 (MHOM/NP/03/BPK282/0cl4) reference genomes [26], [27], [28] for analysis, with each reference indexed by a kmer of 13 and skip size of six. JPCM5 was cloned from an isolate from the spleen of a dog in central Spain and was characterised as *L. infantum* zymodeme 1 (MON-1) by isoenzyme analysis [53]. BPK282/0cl4 was cloned from an isolate taken from a Nepalese patient with visceral leishmaniasis [26].

Deep genome-sequencing ensured that a conservative variant screening approach could be implemented as previously outlined for genotype calling, variant detection and population-wide allele frequency inference [26]. Only variants within two standard deviations of the normalised median chromosome coverage for each strain and also with no evidence of strand-specific mapping (determined from the ratio of reads mapping to forward and reverse strands and employing a cutoff of at least 3 variant calls on each strand for each SNP) were examined. To ensure the neutrality of the variants observed, the population-wide allele frequency spectra were inferred for both reference genomes: these displayed exponential patterns of decay (Figure S2). In addition, 30.6% of SNPs were singletons, in line with previous work [26]. The low rate of singleton discovery, coupled with neutral summary statistics (Tajima's D = 0.12, Fu and Li's F* = 0.24 and D* = 0.26) and allele frequency spectra (Figure S3), support the neutrality of the inferred SNPs.

### *De novo* assembly and intra-population variation patterns

SNPs were visualised with Artemis [54]. Heterozygous SNPs were phased with Samtools, with haplotypes phased over at least 5 Kb in at least one strain kept for analysis. A custom Perl script was used to retrieve the sequence from the phased region and replace consensus bases with SNPs. Where a strain was phased, the script generated two sequences for that strain, substituting base predictions for each haplotype into each sequence. Maximum-likelihood phylogenetic analysis of the sequences for each phased block was performed with PHYML v3.69 using the GTR model, and a 4-category approximation to a gamma distribution of rates across sites. Intraspecific diversity was measured as Watterson's θ [55] and nucleotide diversity (π [56]). Recombination tends to inflate intermediate-frequency variants (π) but less so for low-frequency ones (theta), and so we applied Tajima's D [57] to compare π and theta (D>0 if π>θ). The relative neutrality of θ and π with respect to singletons was examined using Fu and Li's D* and F*[58] - these are more likely to be recent heterozygous mutations and thus to be linked to recombination in this study. The genome-wide scans were implemented with a window size of 10 kb and a step size of 5 kb for 3,260 windows in total.

Patterns of linkage between individual SNPs and 10 kb blocks were inferred with BCFtools [59]. LD between 10 kb blocks within and between chromosomes was examined and LD decay with distance was measured for each chromosome. Extensive LD between chromosomes is consistent with rare recombination and supports clonality as the primary reproductive mode.

kDNA assemblies were generated by assembling the paired end reads for all CUK strains, *L. infantum* JPCM5, *L. donovani* LV9 and *L. major* LV39 with Velvet [60] using a kmer of 55. Contigs matching kDNA were identified by BlastP search against the complete *Leishmania tarentolae* kinetoplast genome [61]. A phylogenetic tree of the constant region of the kDNA was generated using PHYML with the GTR substitution model and site rates modelled on an approximate gamma distribution.

### Inferring ancestry contributions using interspecific divergence rates

Levels of divergence of the CUK set to the two *L. donovani* complex genomes (JPCM5 and BPK282/0cl4) were quantified using the number of SNPs between each reference in 10 kb windows and divergence of the CUK set from the genotypes inferred from the mapped read data. Consequently, these data were subject to the conservative variation discovery criteria outlined above. Levels of divergence from *L. major* were determined as described previously [26] to provide a neutral ancestral calibration that had no evidence of recent gene flow with the CUK population, and so reduce the effect of any selective processes on the divergence values. Large variability in the rate of genealogical coalescence of different genomic segments should be reflected in the distribution of divergence values. Consequently, these distributions for each reference genome (*L. infantum* JPCM5, *L. donovani* BPK282/0cl4, *L. major* Friedlin [26], [28], [29]) were fitted to unimodal normal, bimodal normal, gamma, Poisson and Weibull distributions. To allow for some variability in the distributions, the differences between two means for the biomodal model were at least twice the standard deviation, and the standard deviations were set to be at least 0.7 kb. The corrected Akaike Information Criterion (AICc) was used to determine the most likely model.

To objectively assign regions of the CUK genomes to different ancestry, we developed a Poisson mixture modelling approach. This modelled the density of homozygous and heterozygous SNPs in non-overlapping 5 kb windows along the genome as two independent Poisson variables, and allowed the total distribution of homozygous and heterozygous SNP density to be modelled as a mixture of multiple 2-dimensional passion process components. Parameters for these mixture models were estimated using the flexmix package [62] in R v2.11.1 [63]. Individual windows in these samples can then be assigned to their maximum posterior probability components, and the uncertainty in these assignments (and so uncertainty in the underlying ancestry of particular windows) assessed using the non-parametric bootstrap. See Figure S17 for ternary plots of the precision of component assignments and other model criticism.

### Mitotic and meiotic cell division during the *Leishmania* life cycle

To estimate the relative rates of meiotic and mitotic cell division in these strains, we derived independent estimates of the effective population size based on observed levels of recombination and mutation (nucleotide diversity) in our set of samples, following the approach of Tsai *et al.* [64]. As recombination occurs only in meiosis, while both processes introduce mutations, the ratio of these two effective population size estimates indicates the relative proportion of cell divisions within this population that either involve recombination (meioses) or do not (mitoses). The two diversity measures are θ = 4.*N_e_.μ*/(1+*F*) and *ρ* = 4.*N_e_.r.*(1−*F*), where N_e_ is the effective population size, μ is the mutation rate per bp per generation, *r* is the average number of crossing-overs per bp per generation and *F* is Wright's inbreeding coefficient. Here, we estimate θ per chromosome using Watterson's estimator from all silent sites, and estimate ρ per chromosome using the MCMC approach implemented in PHASE v2.1 [65], [66] with default parameters, except that the final run with all loci was set to 25 times as long as other iterations (-X25 command-line parameter). Five independent runs were used for each chromosome and the estimate used only if at least four runs appeared to converge on the same posterior distribution on inspection of the pseudo-likelihoods per generation and the posterior distribution of the parameter *ρ*. Due to the low number of samples, numerical underflow errors were encountered in calculating some likelihoods. *F* was estimated as 1 minus the ratio of observed heterozygosity at silent sites across the genome to the expected heterozygosity at these sites at Hardy-Weinberg equilibrium. For our estimates of F and θ to reflect variation segregating within the Çukurova population, rather than that stemming from the founding admixture event, we calculated these statistics based on those regions of the genome identified as being homozygous for one of the two parental genotypes in the mixture model analysis. Direct estimates of *μ* and *r* are available for many model species, from mutation-accumulation experiments [67] for *μ* and from genetic crosses for *r*, but these data are not available for any *Leishmania* species. Fortunately, there is a strong relationship between these parameters and genome size, so we estimate these parameters, and a prediction interval around each from a log-log regression, based on a genome of 32.8 Mb, following the approach of and using data on recombination rates from [68] and on mutation rates from [69]. In the first case [68], the outlier value for *Toxoplasma gondii* was replaced with a more recent estimate [70] (see Figure S16).

## Supporting Information

Figure S1 Geographical distribution of isolation sites for the CUK isolates used in this study. The arrowed numbers (1–12) correspond to the villages where the CUK isolates were collected; the coordinates and other details are listed in Table S1.
(PDF)

Figure S2 Phylogeny of CUK strains and other sequenced *L. donovani* complex genomes. Neighbour-joining network based on genome-wide SNPs for all 12 CUK isolates, the Spanish *L. infantum* reference genome strain JPCM5 (MCAN/ES/98/LLM877), the Nepalese *L. donovani* reference genome strain MHOM/NP/03/BPK282/0cl4 and an Ethiopian *L. donovani* strain LV9 (MHOM/ET/67/HU3) [26], [27]. Inferred using Splitstree v4.12.6 [71]. The network reflected the considerable genetic differences between the CUK set compared to other sequenced strains, confirming that they represent a genetically-distinct group in comparison to other sequenced *L. donovani* complex genomes. After excluding sites with unknown genotypes (15,468), a total of 179,158 SNPs were used including 68,550 non-parsimony-informative sites. The genetic distance between strains is indicated: that from the CUK group to JPCM5 was much lower than to BPK282/0cl4 (41,142 vs 98,001) but far fewer than the number of sites varying within the CUK group (17,333). The genetic diversity between the Turkish CUK strains is highlighted in the inset.
(PDF)

Figure S3 Derived allele frequency spectra for 12 CUK isolates. Derived allele frequency spectra for 12 CUK strains, based on reads mapped to (A) the Spanish *L. infantum* JPCM5 and (B) Nepalese *L. donovani* BPK282/0cl4reference genomes. Both spectra indicate a pattern of exponential decay supporting the action of purifying selection in the population, although with a slight disparity between the genome-wide (black) and coding sequence (grey and dashed) for the BPK282/0cl4 reference. This is a result of the greater genetic distance between BPK282/0cl4 and the Turkish set in comparison to the JPCM5 reference.
(PDF)

Figure S4 Genome-wide patterns of polymorphism in the CUK strains. (A) All single-nucleotide polymorphisms (SNPs) are shown with respect to *L. infantum* JCPM5 in all chromosomes for all 12 strains. Orange ‘blocked’ bars indicate heterozygous positions; green, red, blue and black bars indicate homozygous variant calls of A, C, G and T respectively. Isolates 1–12 are shown in order from the bottom to top track of the figures for each chromosome. (B) The same plots in (A) are shown with orange ‘blocked’ bars indicating heterozygous positions and black bars indicating homozygous variant calls.
(ZIP)

Figure S5 Non-reference allele frequencies for each chromosome. Plots represent the mean frequency of non-JPCM5 alleles at variable sites for each 1 kb window across each chromosome for the 12 CUK isolates.
(PDF)

Figure S6 Non-parametric bootstrap analysis of mixture model parameters and cluster assignments. (A) Uncertainty in parameter estimates based on bootstrap replicates of CUK1 for the three model components, confirming that classification into high- and low- homozygous SNP and high-heterozygous SNP components is robust. Coloured dots represent parameter estimates for components of bootstrap replicates, crosses indicate parameter estimates based on observed data. Note that the points represent uncertainty in the centres of each component, and so are more compact that the probability distributions for each component shown in Figure S17(A) with a different scale for the heterozygous SNPs per window (y-axis). (B) Distribution of bootstrap estimates of the proportion of the CUK1 genome derived from the ‘low SNP density’ ancestor, based on maximum posterior probability assignment under mixture model. Dotted line indicates the combined estimate for observed data. Figures are all based on 1,000 non-parametric bootstrap replicates, resampling randomly from the set of 5 kb genomic windows.
(PDF)

Figure S7 Maximum-likelihood phylogeny of assembled kDNA constant regions for the CUK strains and sequenced *L. donovani* complex genomes. PHYML Maximum likelihood tree of the constant region of the kDNA shows that the kDNA of the CUK strains share a closer common ancestry with *L. infantum* JPCM5 than they do with *L. donovani* BPK282 or *L. donovani* LV9. *L. tarentolae* was chosen to root the tree as it is the most closely related outgroup to the sub-genus *Leishmania* for which whole-genome sequence data are available (reviewed in [72]). In addition, the kDNA maxicircle is fully assembled for this species.
(PDF)

Figure S8 Maximum-likelihood phylogenies of CUK isolates and sequenced *L. donovani* complex genomes based on phased haplotypes. Maximum-likelihood phylogenies are shown for all regions in which at least one CUK isolate could be phased to produce haplotype sequences of at least 5 kb. (A) 60 out of 74 phylogenies show different haplotypes for CUK isolates from two different clades, while (B) 14 do not show this pattern. Titles of each phylogeny indicate the chromosome number and location of phased region on the JPCM5 reference sequence. Colours indicate sets of different haplotypes, with the three reference genomes consistently coloured throughput.
(PDF)

Figure S9 Extensive variability of diversity between chromosomes in the CUK strains. Diversity for each chromosome was calculated for the 12 Turkish strains using the mean nucleotide diversity (Pi, π) [55] and Watterson's theta (θ) [56] per kb for each chromosome. π most strongly reflected intermediate-level variants, and θ lower-frequency ones: under neutrality these should be approximately equal but recombination generally will increase the π to θ ratio. 4,268 genes contained fixed SNPs compared to the *L. infantum* genome and 1,820 had variation within the 12 strains – 1,042 genes had both inter- and intra-specific variation.
(PDF)

Figure S10 Heterozygosity across chromosomes across the 12 CUK strains for each chromosome using *L. infantum* JPCM5 and *L. donovani* BPK282/0cl4 reference genomes. Heterozygosity within the 12 Turkish strains for each chromosome using *L. infantum* JPCM5 (blue) and *L. donovani* BPK282/0cl4 (red) reference genomes was measured per kb (upper panel) and in total numbers (lower panel). The *L. infantum* JPCM5 (blue) reference genome provided a better resolution of heterozygous alleles than *L. donovani* BPK282/0cl4 (red) because of its closer genetic distance. No association between SNP heterozygosity and chromosome copy number was evident, suggesting that chromosome copy number variation was recent rather than ancestrally stable.
(PDF)

Figure S11 Mean intra- and inter-chromosomal pairwise linkage disequilibrium in the CUK strains. Intra- and inter-chromosomal linkage disequilibrium (LD) in the CUK strains was calculated for each SNP with all others on the same (intra) or a different (inter) chromosome for (A) variants segregating within the Turkish population only (“recent” polymorphism) and (B) including those fixed differences between the CUK isolates and the *L. infantum* JPCM5 genome (“hybrid” polymorphism). Values displayed are the averages across all SNP pairs for each comparison. Interchromosomal LD for both comparisons was higher on all 36 chromosomes except for 22, 25, 30 and 31, suggesting that recombination may be more frequent on the latter relative to other chromosomes. Mean LD for “recent” pairs on the same chromosomes (0.633±0.415 for 7,080,857 pairs) was higher than for pairs on separate chromosomes (0.584±0.441 for 143,126,165 pairs; t-test p<0.0001). Standard deviation values for “recent” LD within chromosomes were large but showed little variation, ranging from 0.42 to 0.52. Similarly, those between chromosomes were also uniform (0.44–0.50). This suggested inherited recombination events that occurred since the original hybridisation event were specific to certain chromosomes only: extensive recombination would result in intrachromosomal LD values much higher than interchromosomal ones.
(PDF)

Figure S12 Genome-wide linkage disequilibrium in the CUK population. Intra-and inter-chromosomal linkage disequilibrium (LD) across the genome using SNPs variable within the 12 CUK strains. Values displayed are the average LD (r^2^) for all SNPs between pairs of 10 kb blocks scaled from low (blue) to high (beige) LD. Intra-chromosomal LD is shown in boxes along the diagonal; inter-chromosomal values are shown above this. The LD patterns provide evidence of extensive LD between chromosomes. Blocks with low LD represent putative recombination breakpoints that occurred since the hybridisation event.
(PDF)

Figure S13 Linkage disequilibrium per chromosome in the CUK population. Values displayed are the average LD (r^2^) for all SNPs segregating in the CUK population, between pairs of 10 kb blocks scaled from low (blue) to high (beige) LD for each pair of blocks on the same chromosome, for each chromosome in the *L. infantum* genome.
(ZIP)

Figure S14 Decay of linkage equilibrium between SNP pairs with distance for each chromosome. Linkage disequilibrium (LD) decay with distance for the entire chromosome. The LD (r^2^ between SNP pairs, y-axis) is on the inset (ranging from 0 to 1). The distance (x-axis) is shown in units of 10 kb. LD decay varied considerably between chromosomes. Bottom: Diversity at each chromosome region shown in 10 kb blocks for the fixed SNPs per kb compared to *L. infantum* JPCM5 (red), those relative to *L. donovani* BPK282/0cl4 (blue), the nucleotide diversity (π) per 10 kb for variation within the CUK population (green), and the summary statistic Tajima's D. Note the difference scale for π compared to the fixed SNPs. Tajima's D compares the level of intermediate- and low-frequency variants; here positive D values tended to occur at possible recombination blocks.
(PDF)

Figure S15 Variation in allele frequencies for heterozygous sites on multisomic CUK chromosomes. Analyses are shown for Chromosomes 4,6,15,22–25,31–33. Shifts in allele frequencies from 0.33 to 0.66 on trisomic chromosomes or 0.25 to 0.75 or 0.5 on tetrasomic chromosomes underscore recombination sites.
(PDF)

Figure S16 Regression model estimates of mutation rate and recombination rate in *Leishmania infantum*. (A) Recombination rate and (B) mutation rate both show a consistent relationship with genome size across eukaryotic species. (A) Points show data for eukaryotic species from [55]; (B) Points show data from [56]. In both panels, the solid line represents the predicted relationship based on a log-log regression model, with dark and light shading represent 95% and 99% prediction intervals, respectively. Dashed line indicates the intercept for *Leshmania infantum*, with an estimated genome size of 32.8 Mb.
(PDF)

Figure S17 Detailed results of the mixture model analysis. (A) Counts of heterozygous and homozygous SNPs in non-overlapping 5 kb windows along the genomes of 12 CUK strains (points), assigned to three classes (point colours) by maximum posterior probability of cluster membership under the 3-component 2-dimensional Poisson mixture model. Ellipses represent density of each of the three model components, with different shading representing the 95%, 90% and 75% quantiles of the probability distributions for each. (B) Proportion of each genome assigned to each of the three categories (i) by maximum posterior probability and (ii) where windows with cluster assignment probability <0.9 are treated as ambiguous. (C) Posterior probability of cluster membership for each 5 kb window (points), represented as a ternary plot. Point colours represent maximum posterior probability cluster assignment; triangle shading density is proportional to number of windows assigned with equivalent probabilities to the three clusters.
(PDF)

Table S1 Source of the CUK strains analysed in this study. Data are provided on the WHO code, source (vector or host), geographical location and coordinates, date of isolation for each strain used (referred to as the CUK strains (1–12) throughout) and accession numbers for the DNA sequence data.
(PDF)

Table S2 Genome-wide read-mapping coverage levels for all 12 CUK strains compared to (A, top) the *L. infantum* and (B, bottom) the *L. donovani* reference genomes.
(PDF)

Table S3 Numbers of variable sites in each CUK strain.
(PDF)

Table S4 The number of pairwise differences between CUK strains.
(PDF)
