# Supplementary figures and images for: Genomic Confirmation of Hybridisation and Recent Inbreeding in a Vector-Isolated Leishmania Population
Source: PLoS Genet. 2014 Jan 16;10(1):e1004092. doi: 10.1371/journal.pgen.1004092 (PMC3894156; doi:10.1371/journal.pgen.1004092)

Figure S1

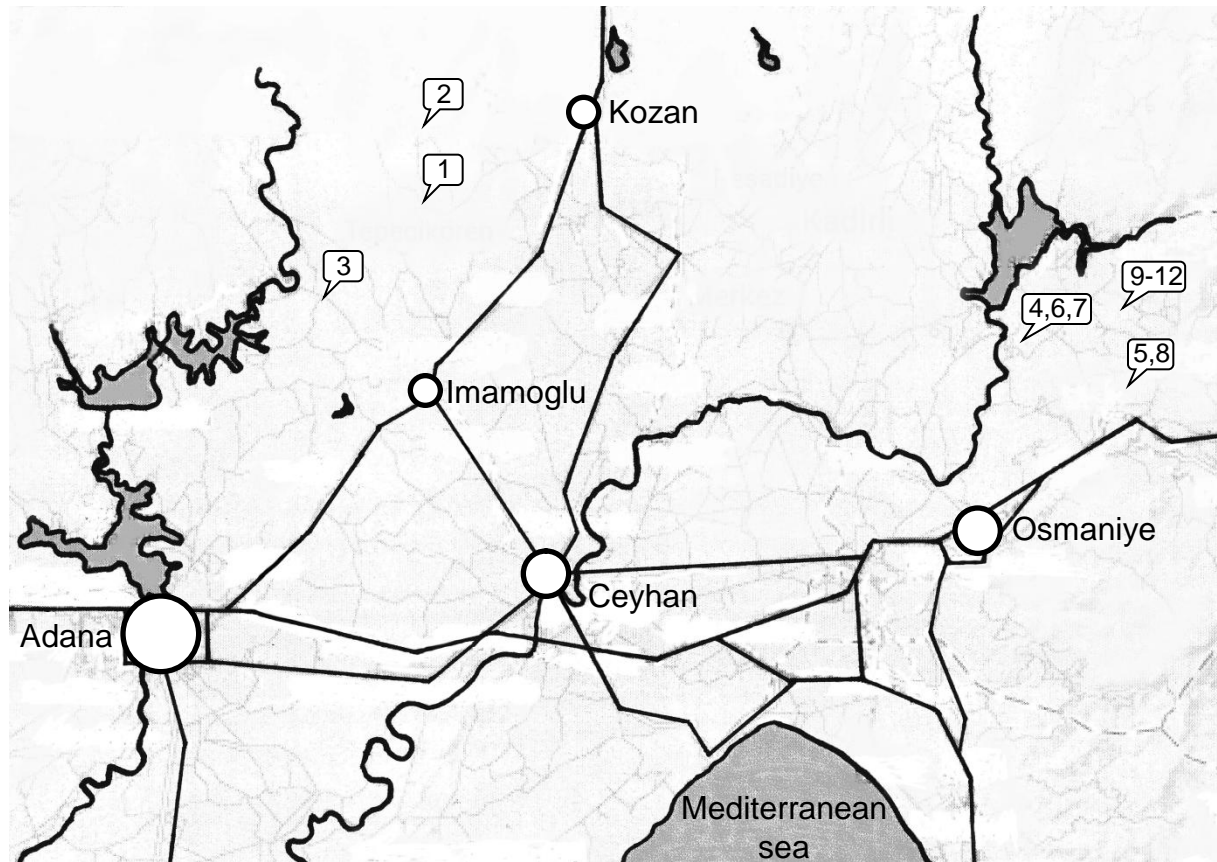

Supplement: Figure S1 — Geographical distribution of isolation sites for the CUK isolates used in this study. The arrowed numbers (1–12) correspond to the villages where the CUK isolates were collected; the coordinates and other details are listed in Table S1. (PDF) [file pgen.1004092.s001.pdf]

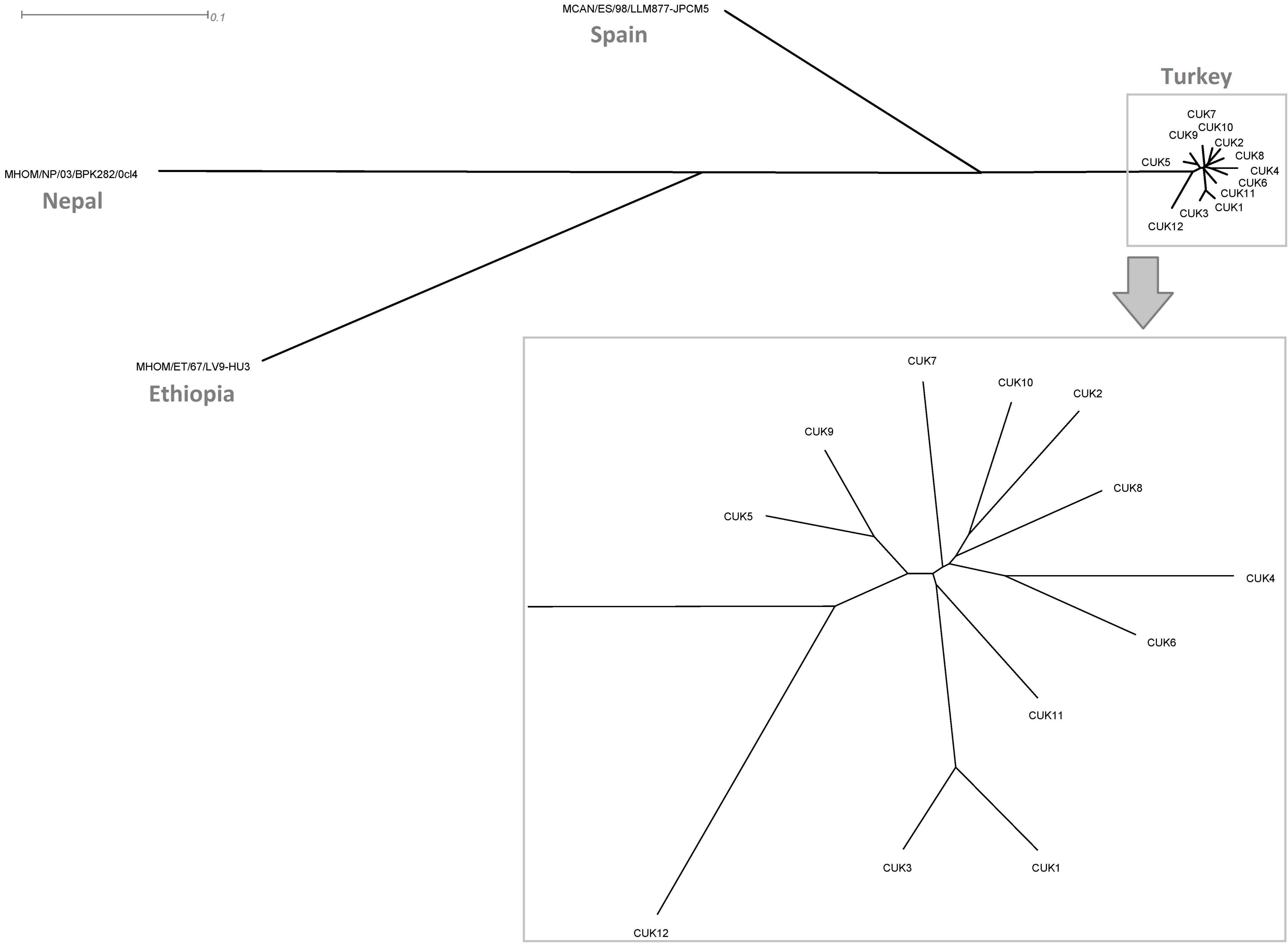

Supplement: Figure S2 — Phylogeny of CUK strains and other sequenced L. donovani complex genomes. Neighbour-joining network based on genome-wide SNPs for all 12 CUK isolates, the Spanish L. infantum reference genome strain JPCM5 (MCAN/ES/98/LLM877), the Nepalese L. donovani reference genome strain MHOM/NP/03/BPK282/0cl4 and an Ethiopian L. donovani strain LV9 (MHOM/ET/67/HU3) [26], [27]. Inferred using Splitstree v4.12.6 [71]. The network reflected the considerable genetic differences between the CUK set compared to other sequenced strains, confirming that they represent a genetically-distinct group in comparison to other sequenced L. donovani complex genomes. After excluding sites with unknown genotypes (15,468), a total of 179,158 SNPs were used including 68,550 non-parsimony-informative sites. The genetic distance between strains is indicated: that from the CUK group to JPCM5 was much lower than to BPK282/0cl4 (41,142 vs 98,001) but far fewer than the number of sites varying within the CUK group (17,333). The genetic diversity between the Turkish CUK strains is highlighted in the inset. (PDF) [file pgen.1004092.s002.pdf]

**A**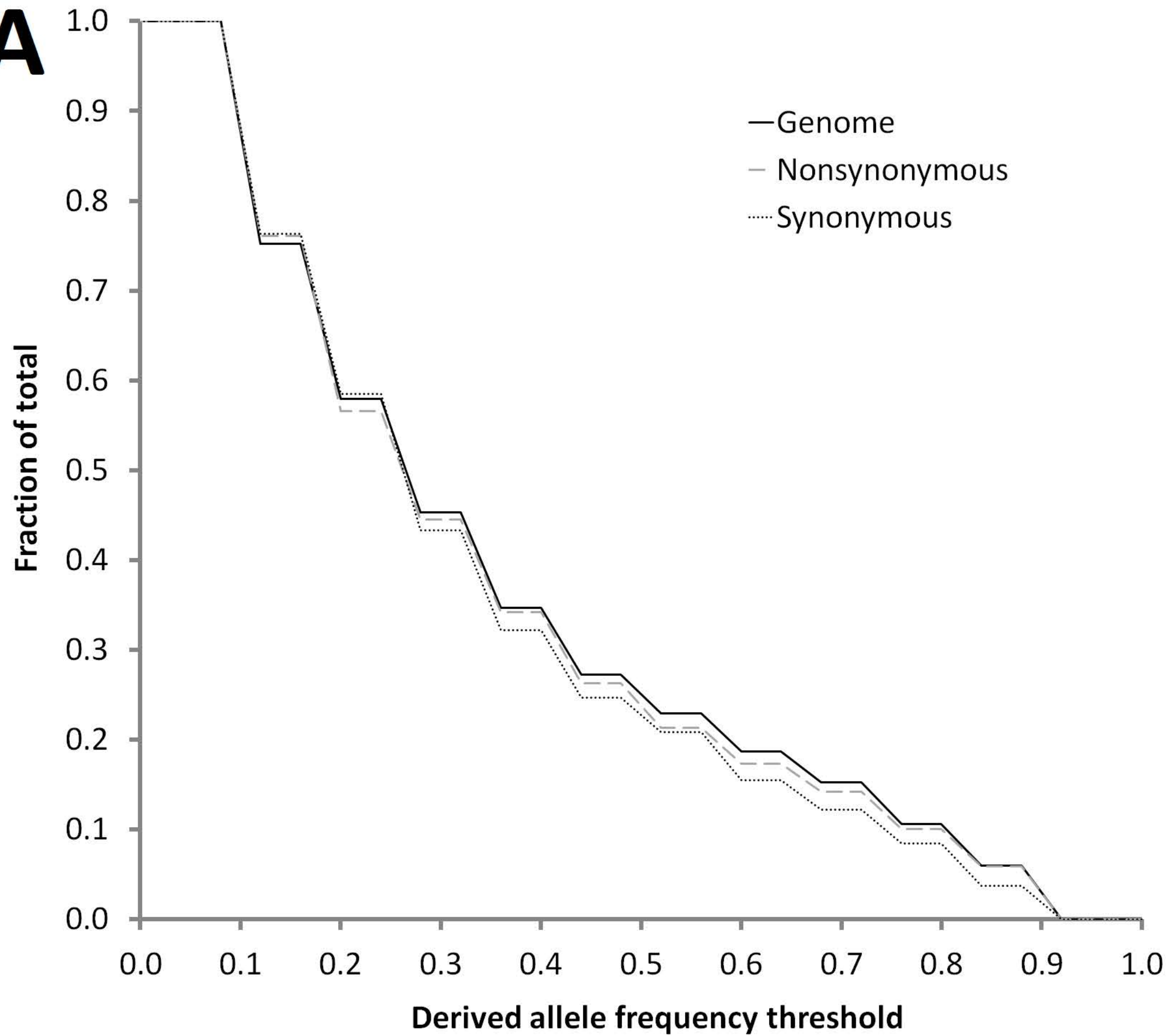**B**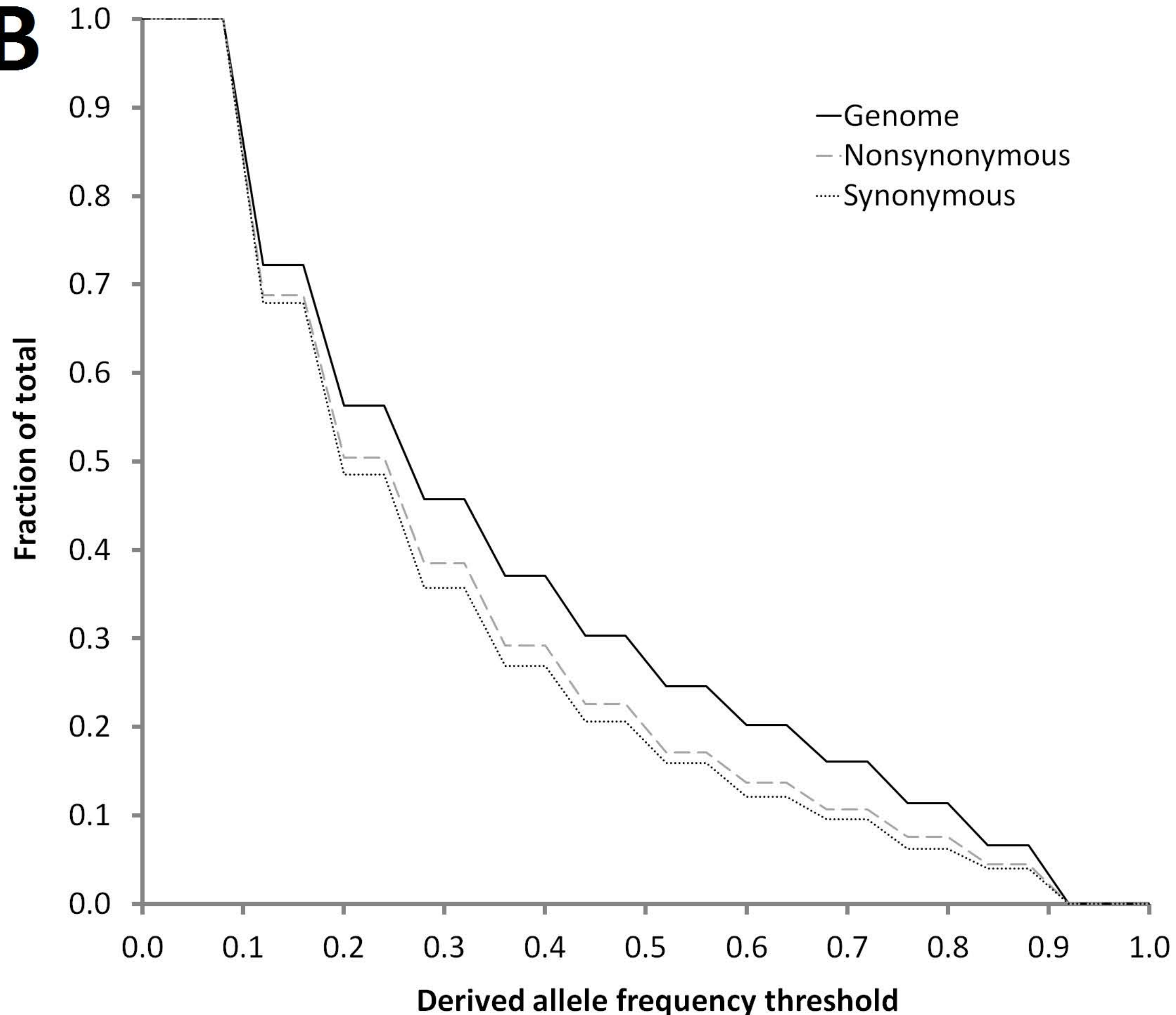

Supplement: Figure S3 — Derived allele frequency spectra for 12 CUK isolates. Derived allele frequency spectra for 12 CUK strains, based on reads mapped to (A) the Spanish L. infantum JPCM5 and (B) Nepalese L. donovani BPK282/0cl4reference genomes. Both spectra indicate a pattern of exponential decay supporting the action of purifying selection in the population, although with a slight disparity between the genome-wide (black) and coding sequence (grey and dashed) for the BPK282/0cl4 reference. This is a result of the greater genetic distance between BPK282/0cl4 and the Turkish set in comparison to the JPCM5 reference. (PDF) [file pgen.1004092.s003.pdf]

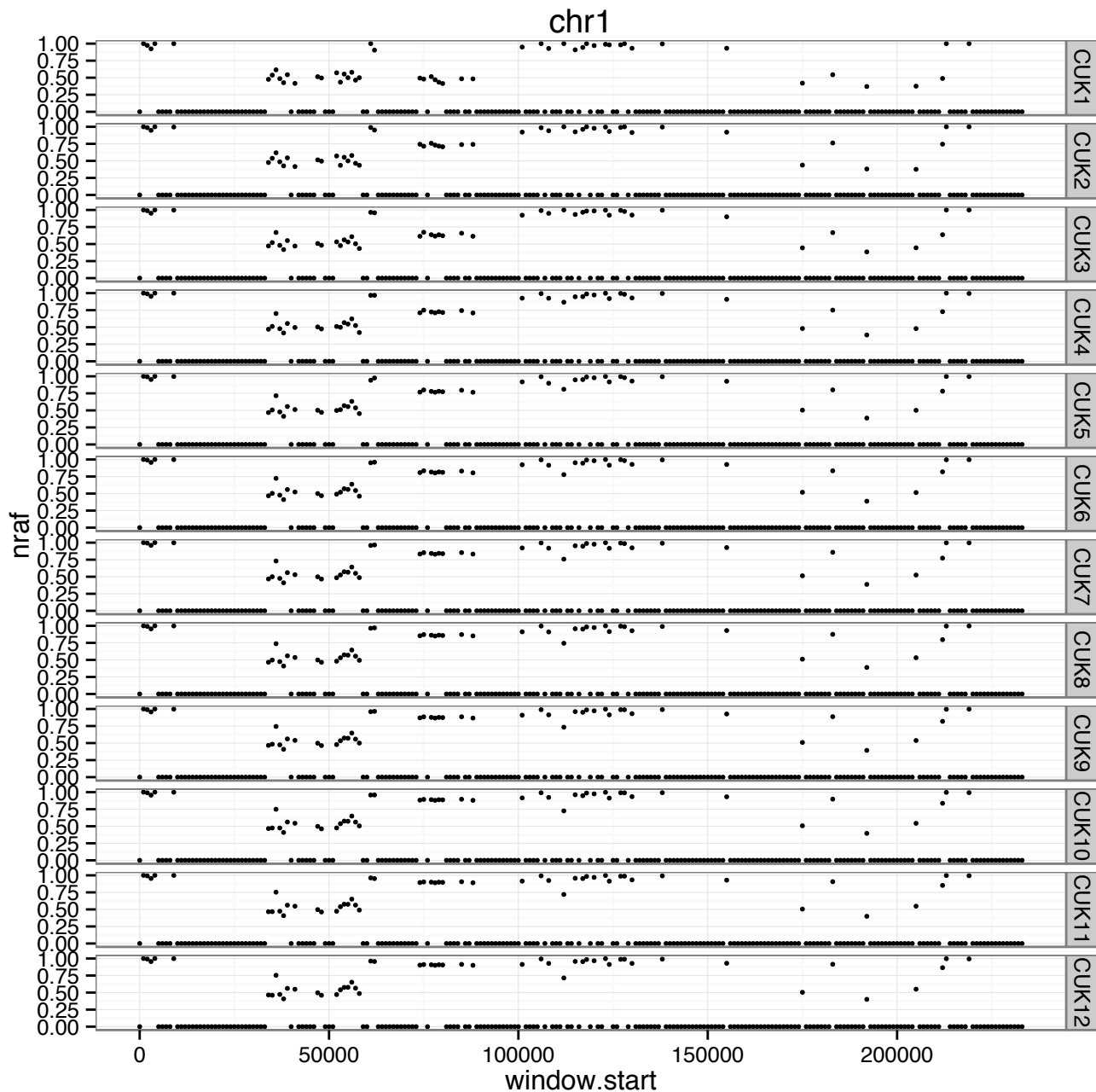

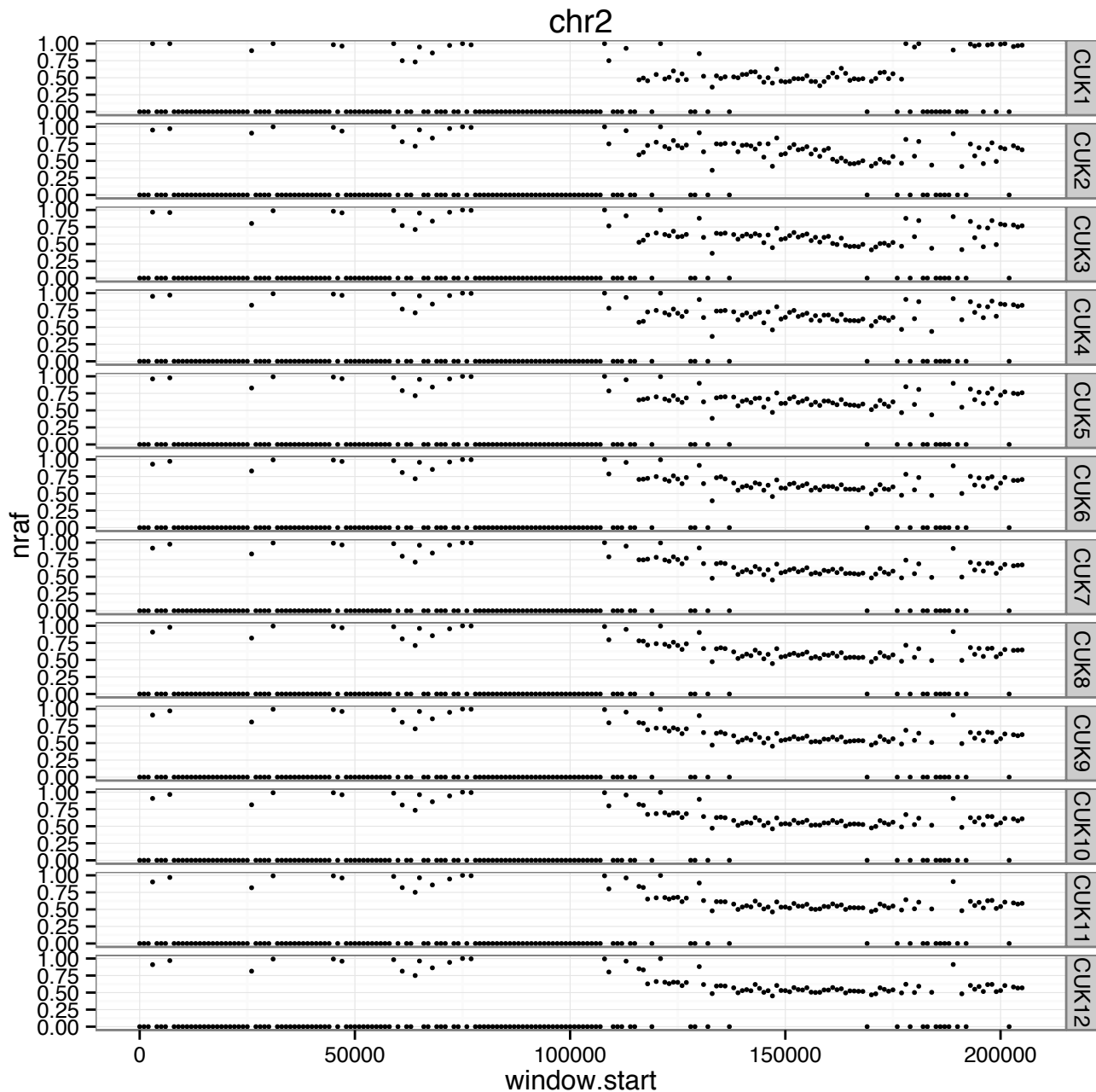

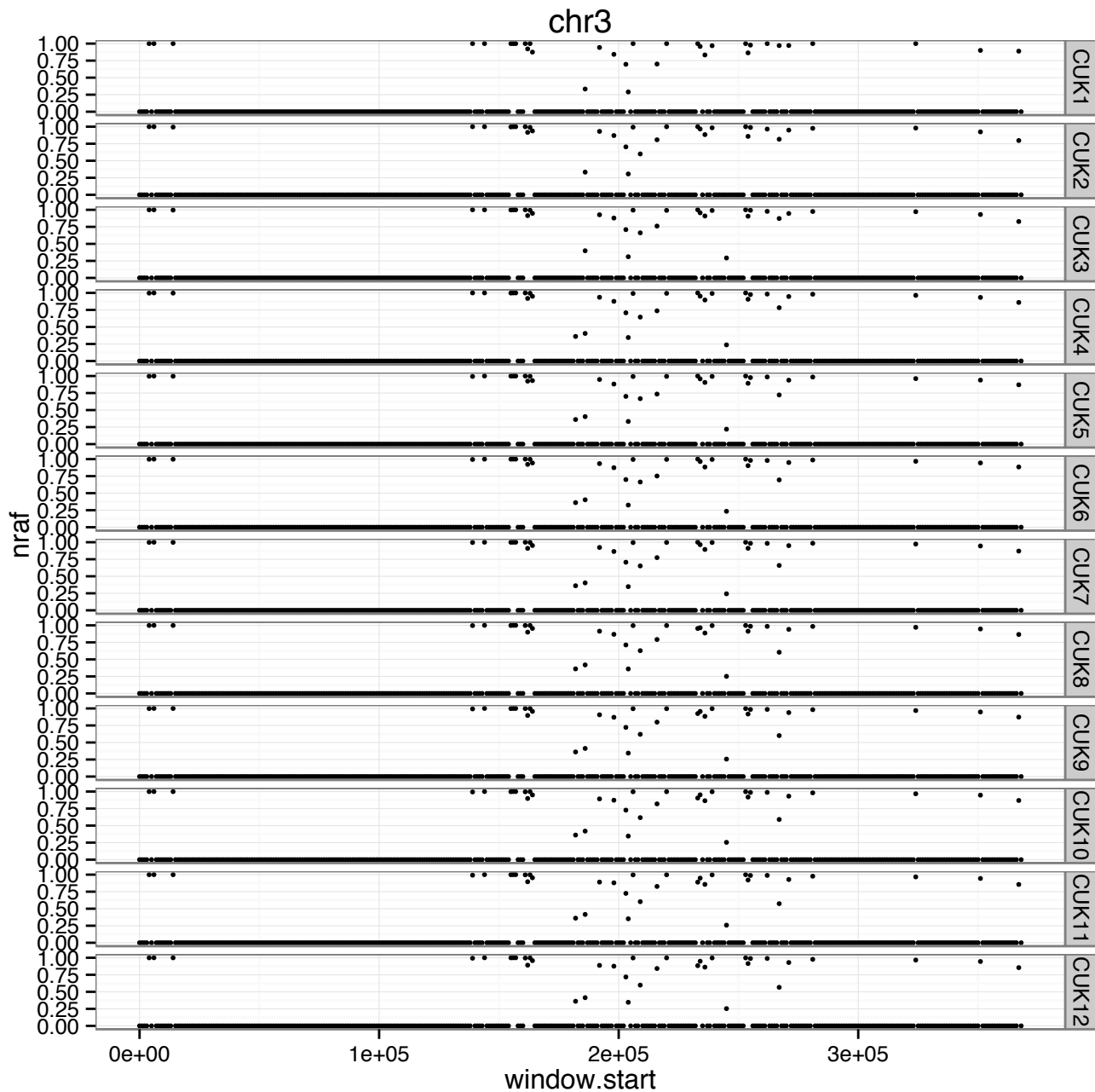

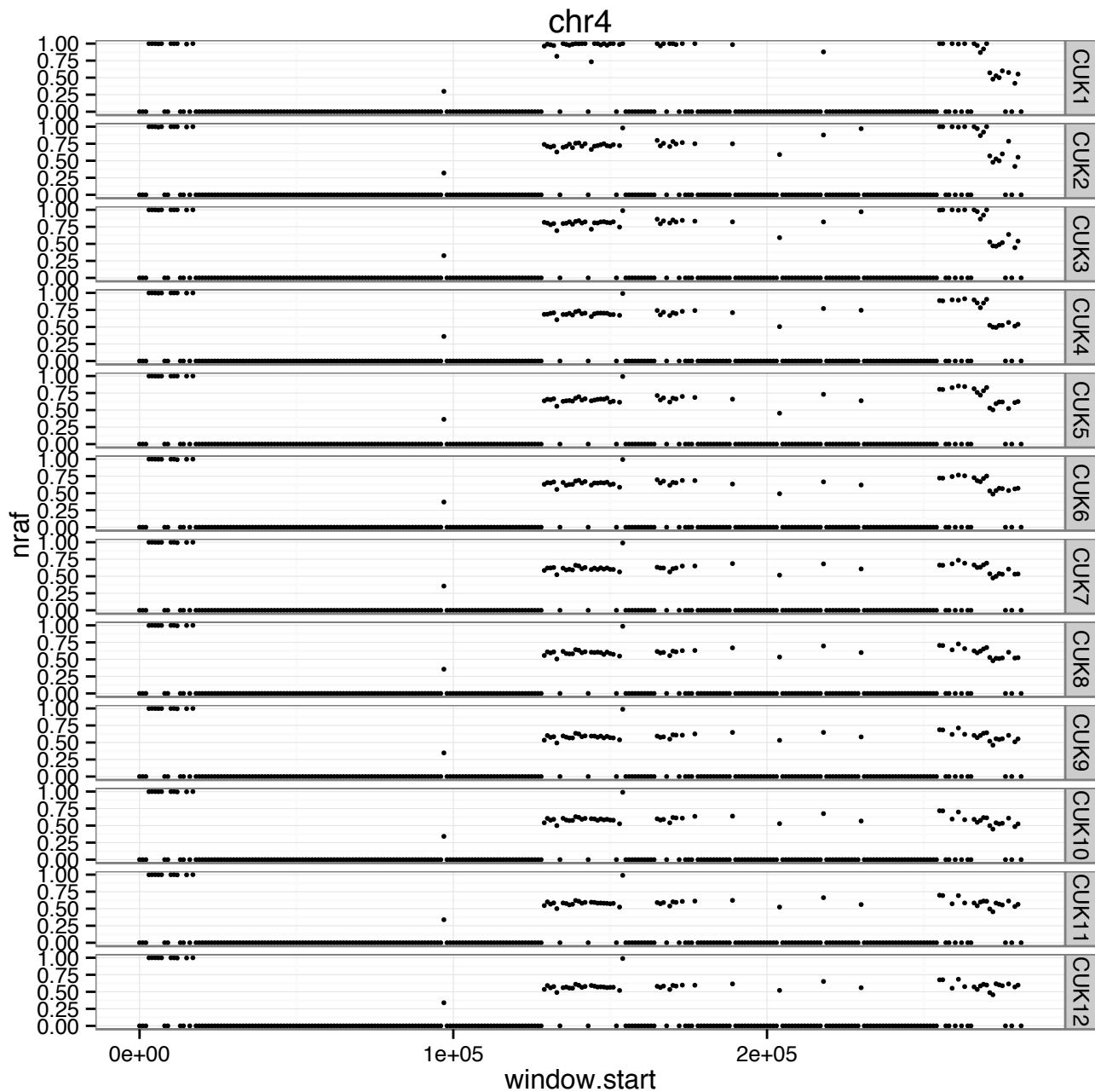

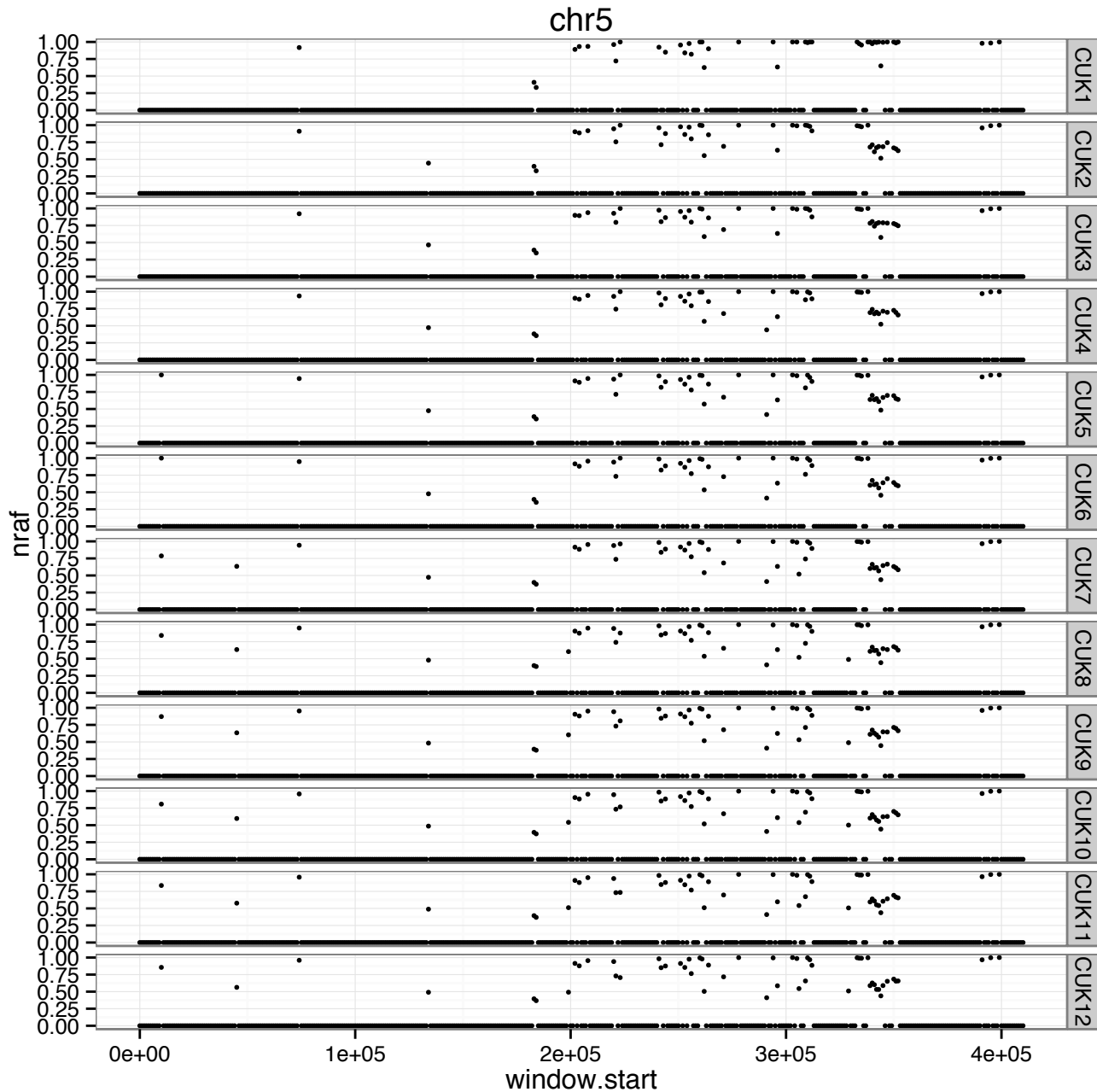

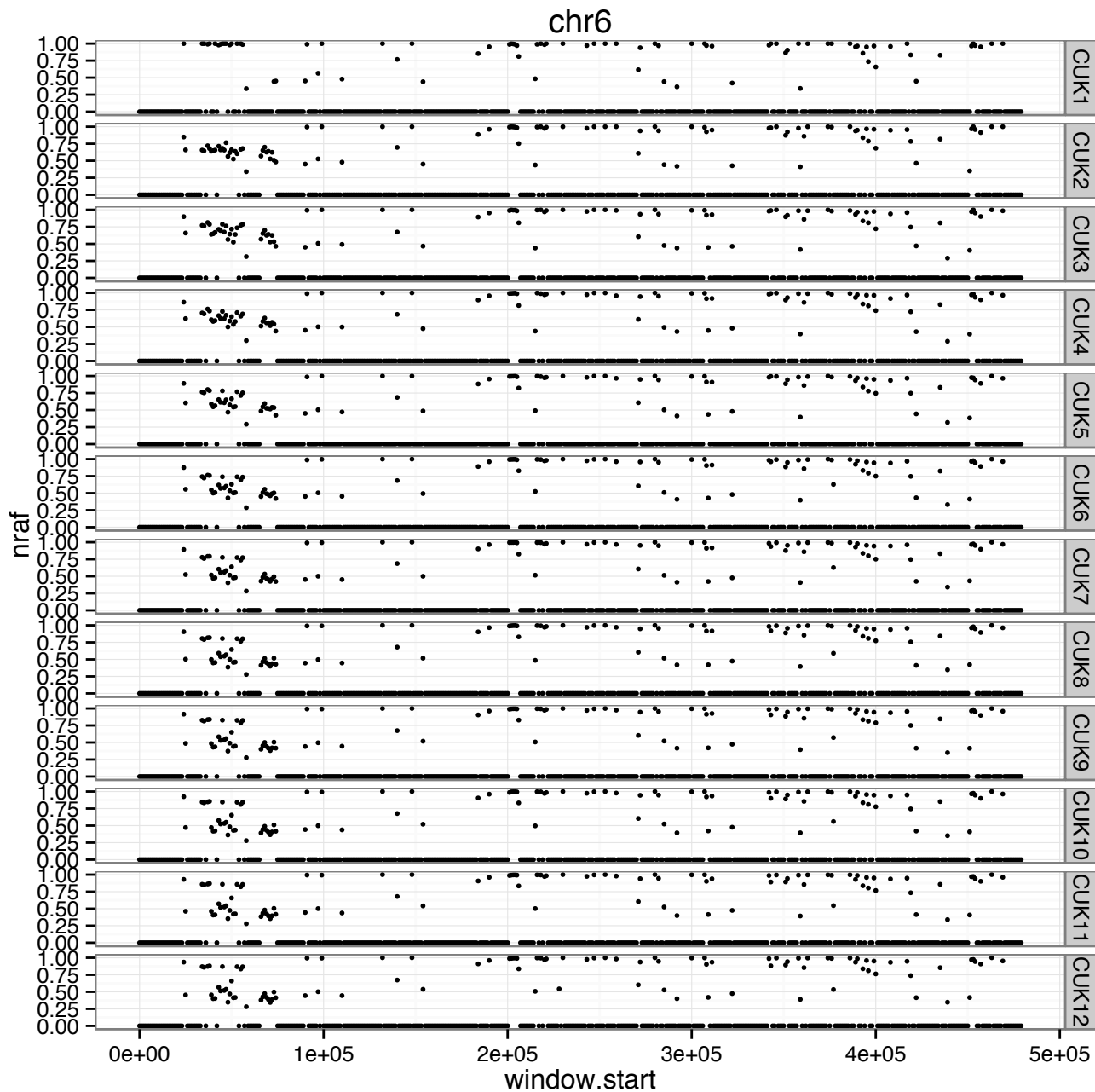

chr7

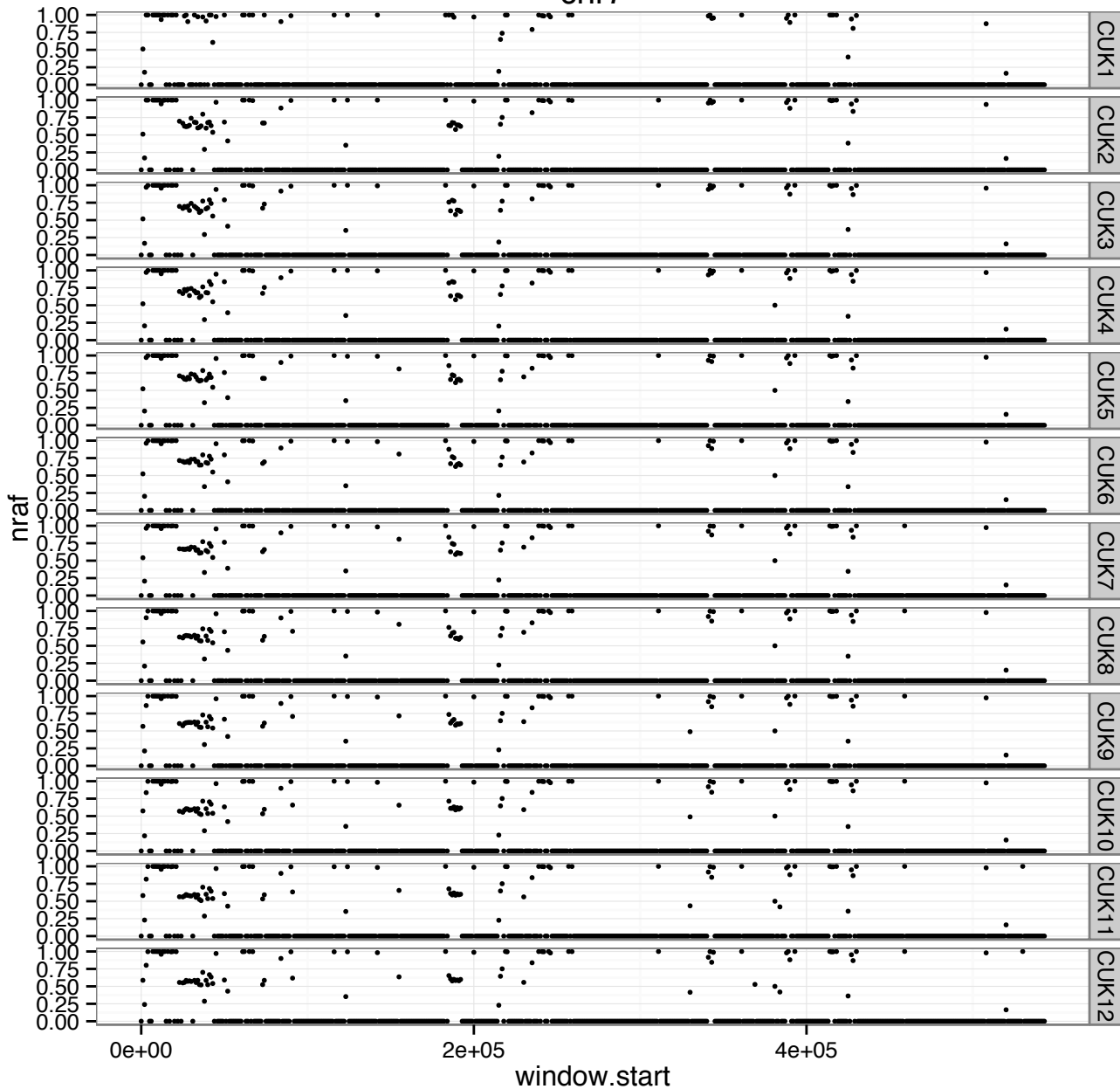

chr8

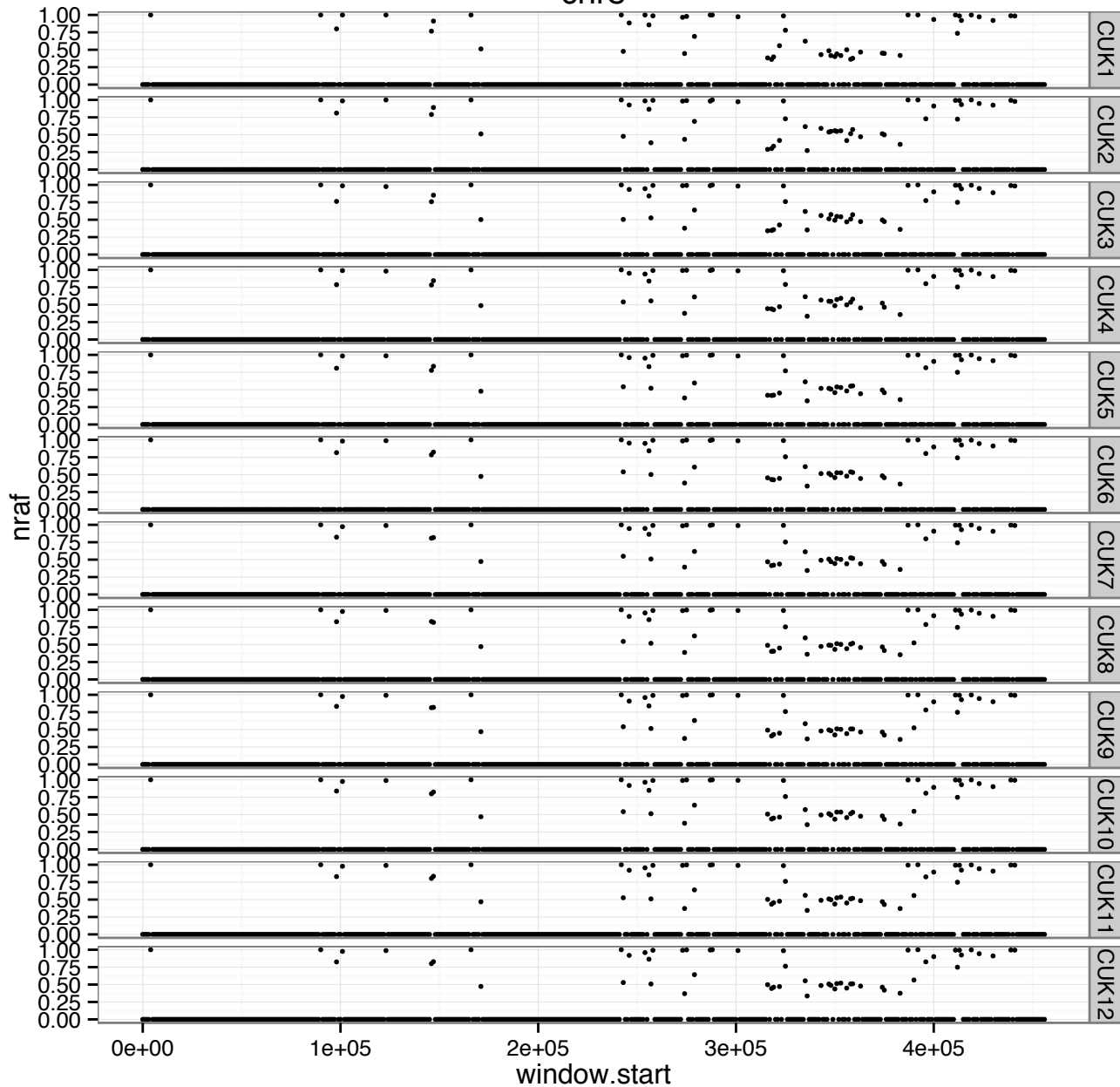

chr9

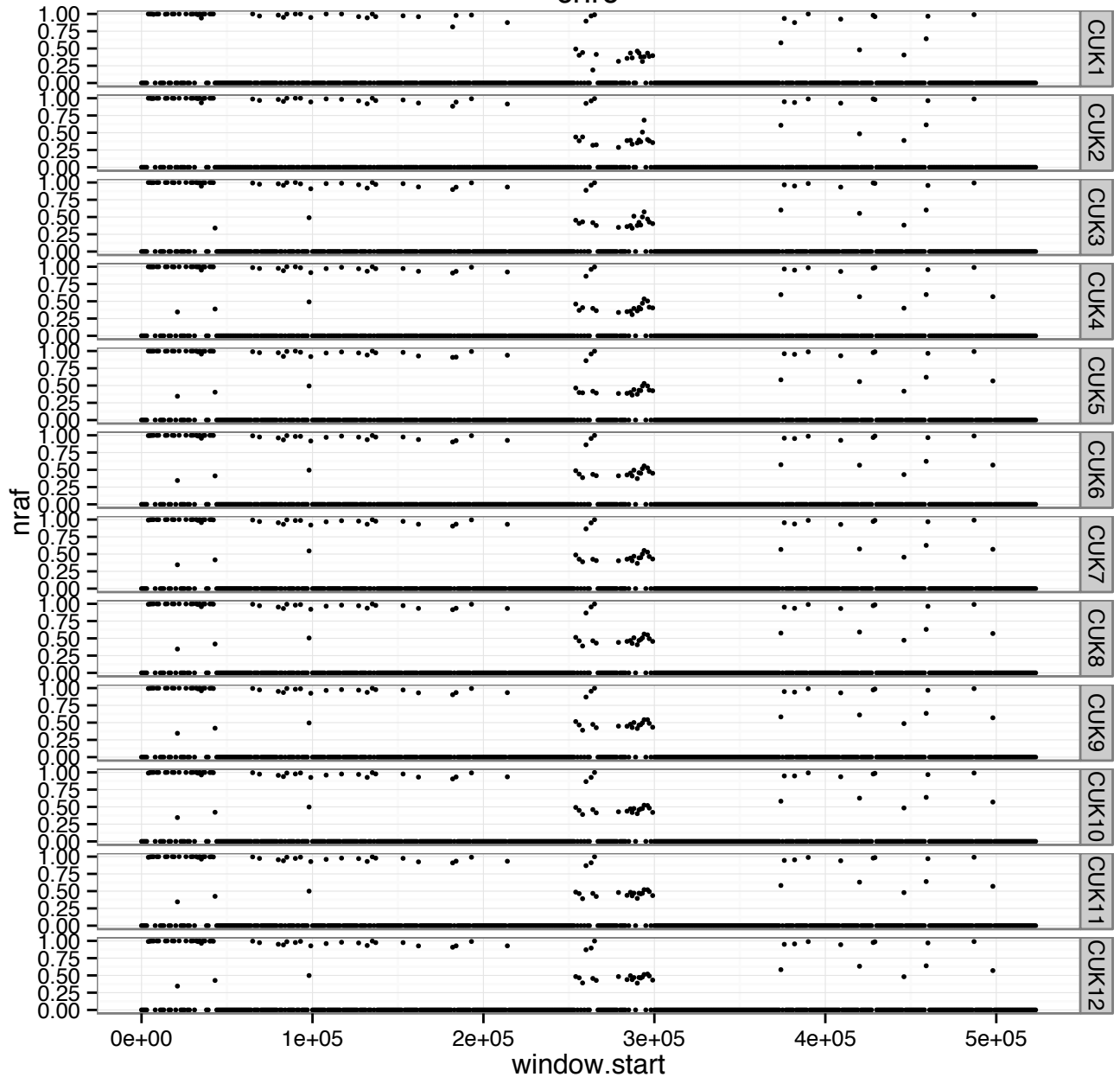

chr10

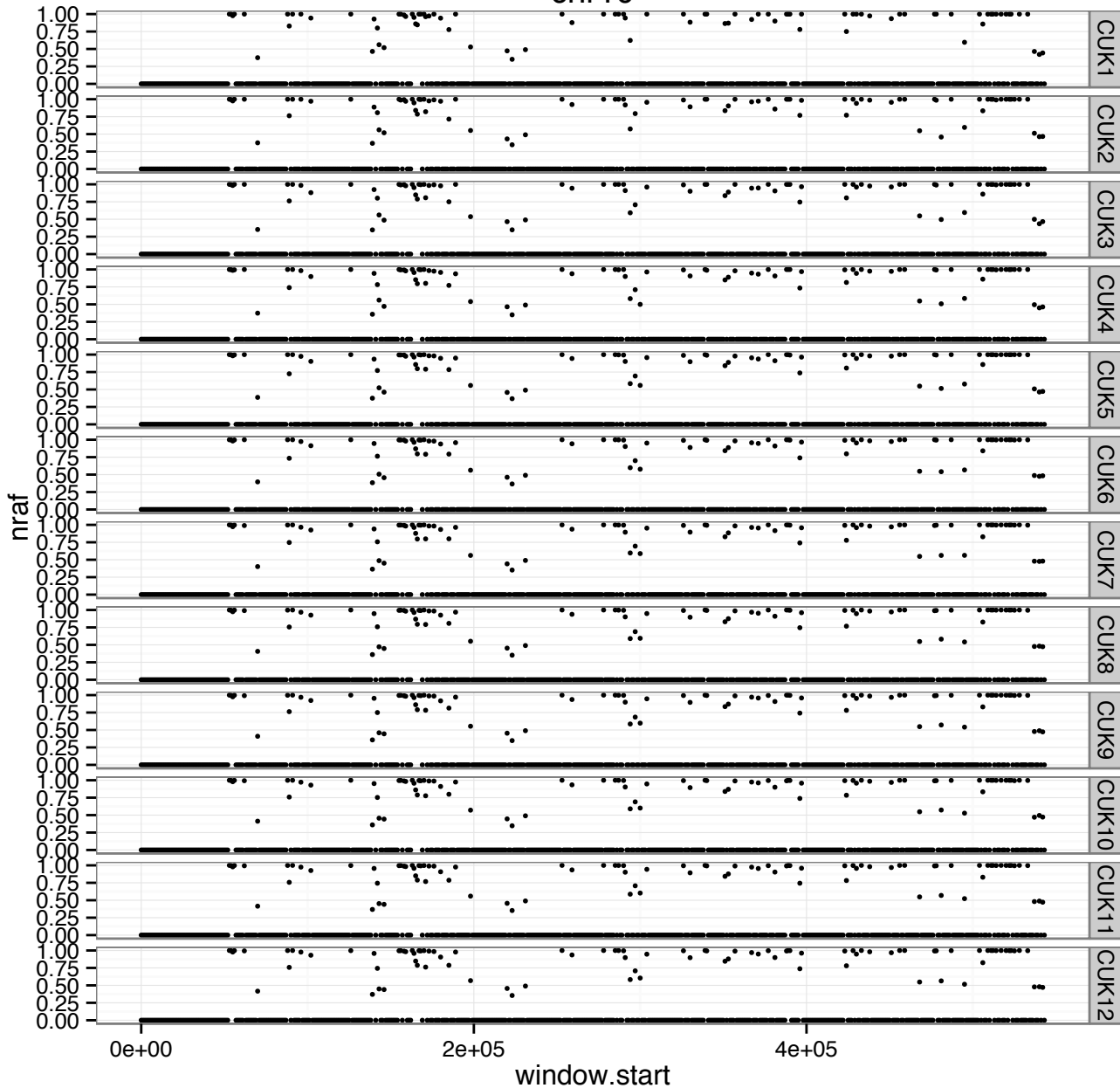

chr11

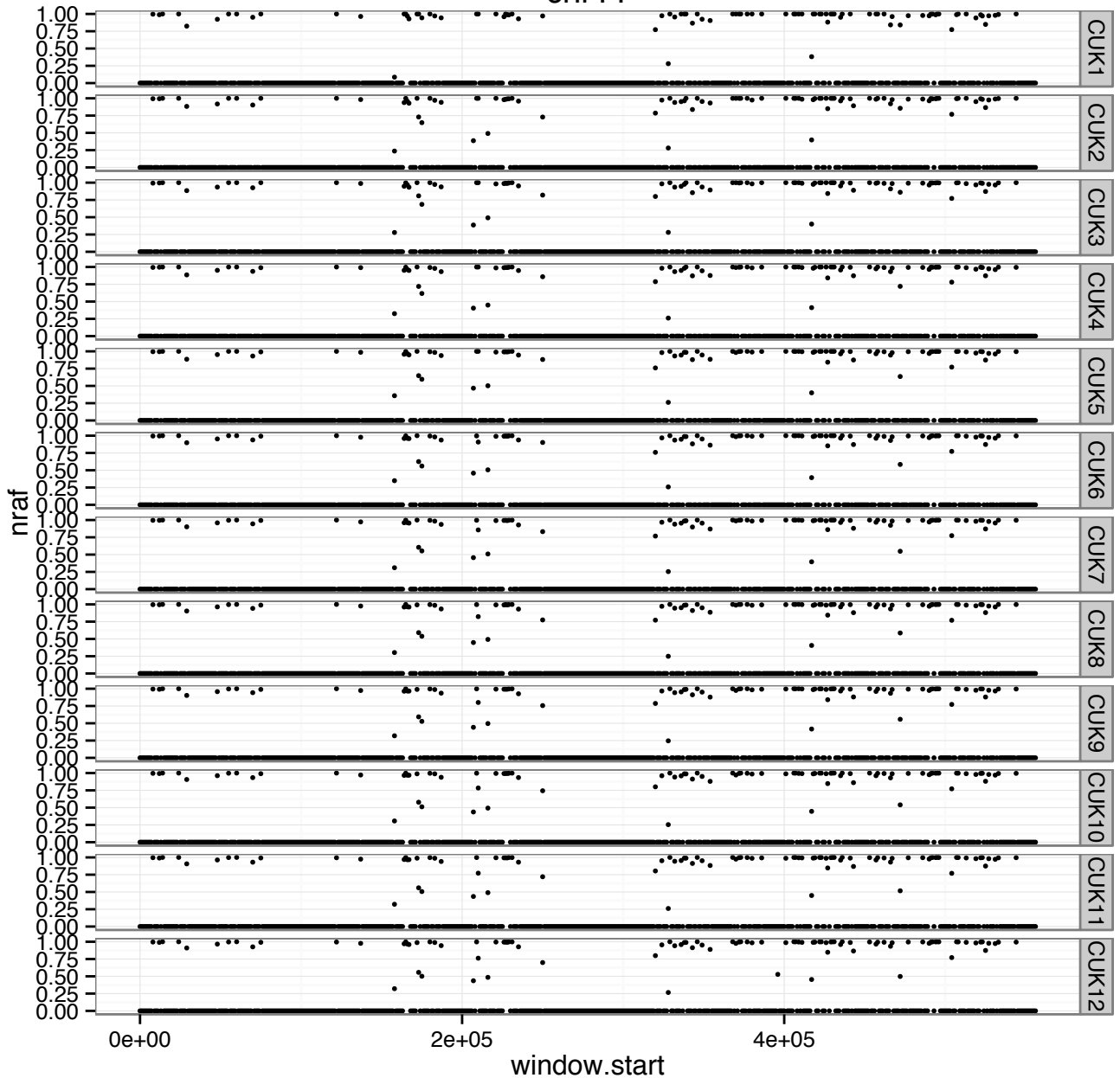

chr12

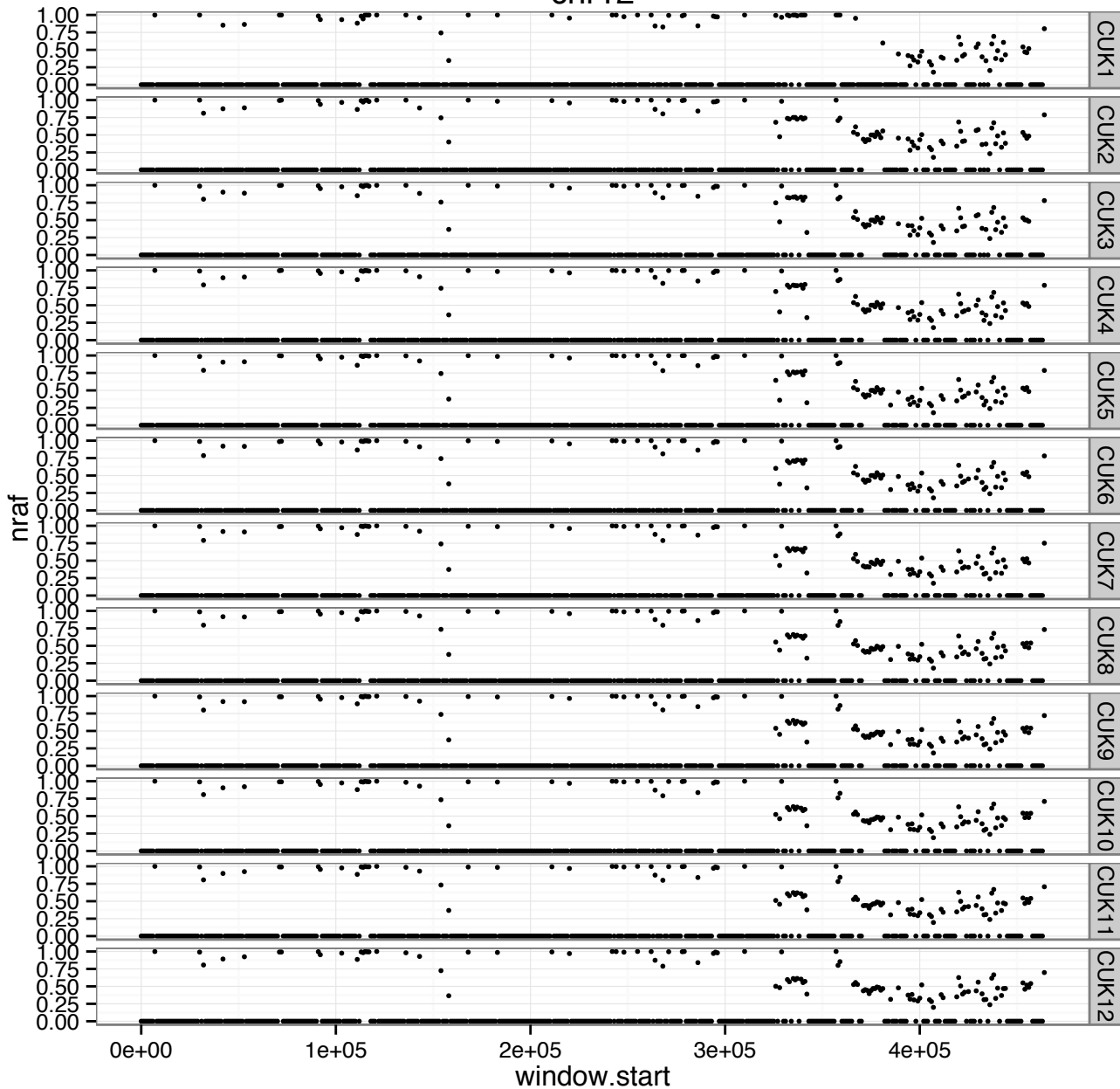

chr13

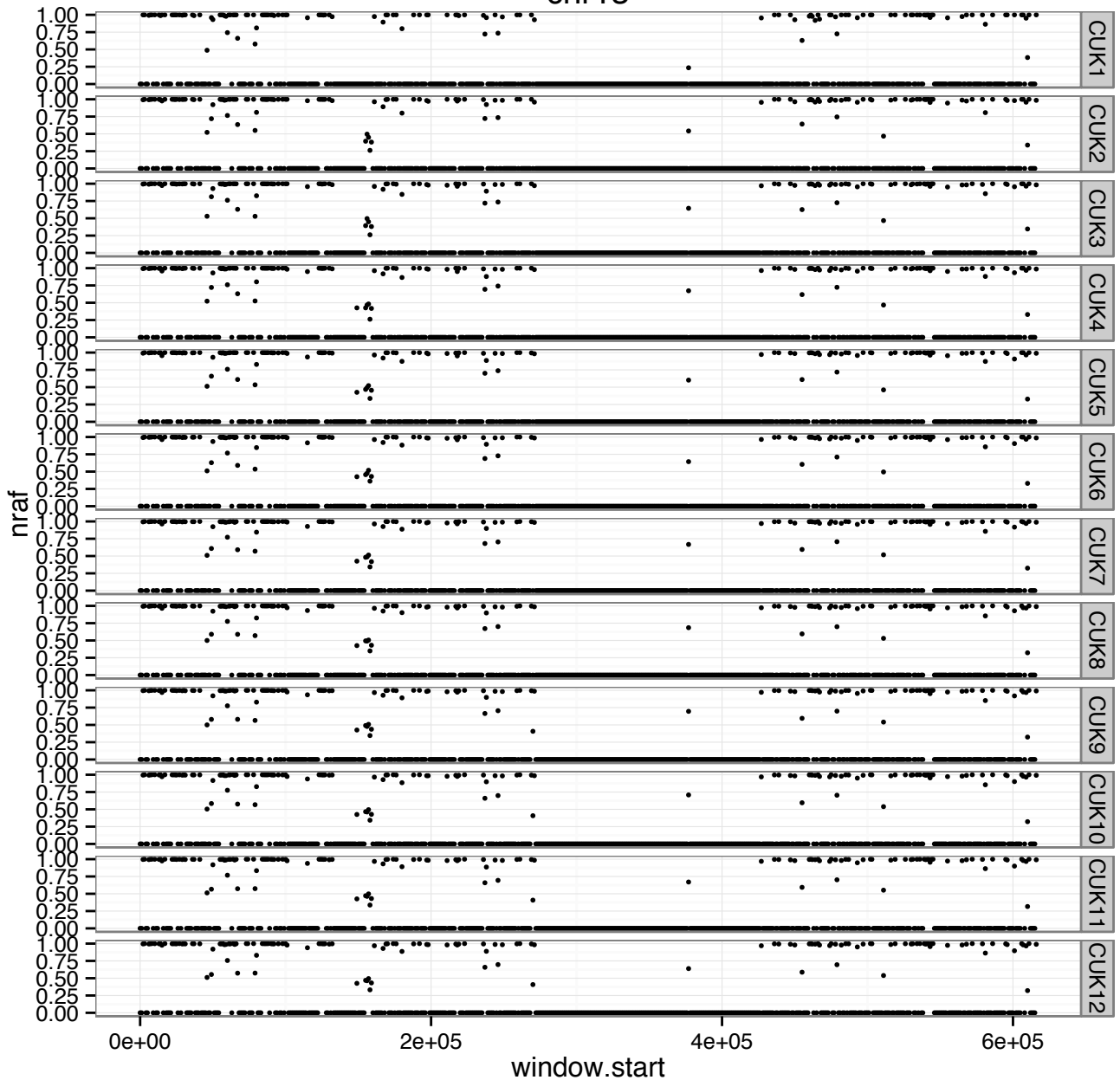

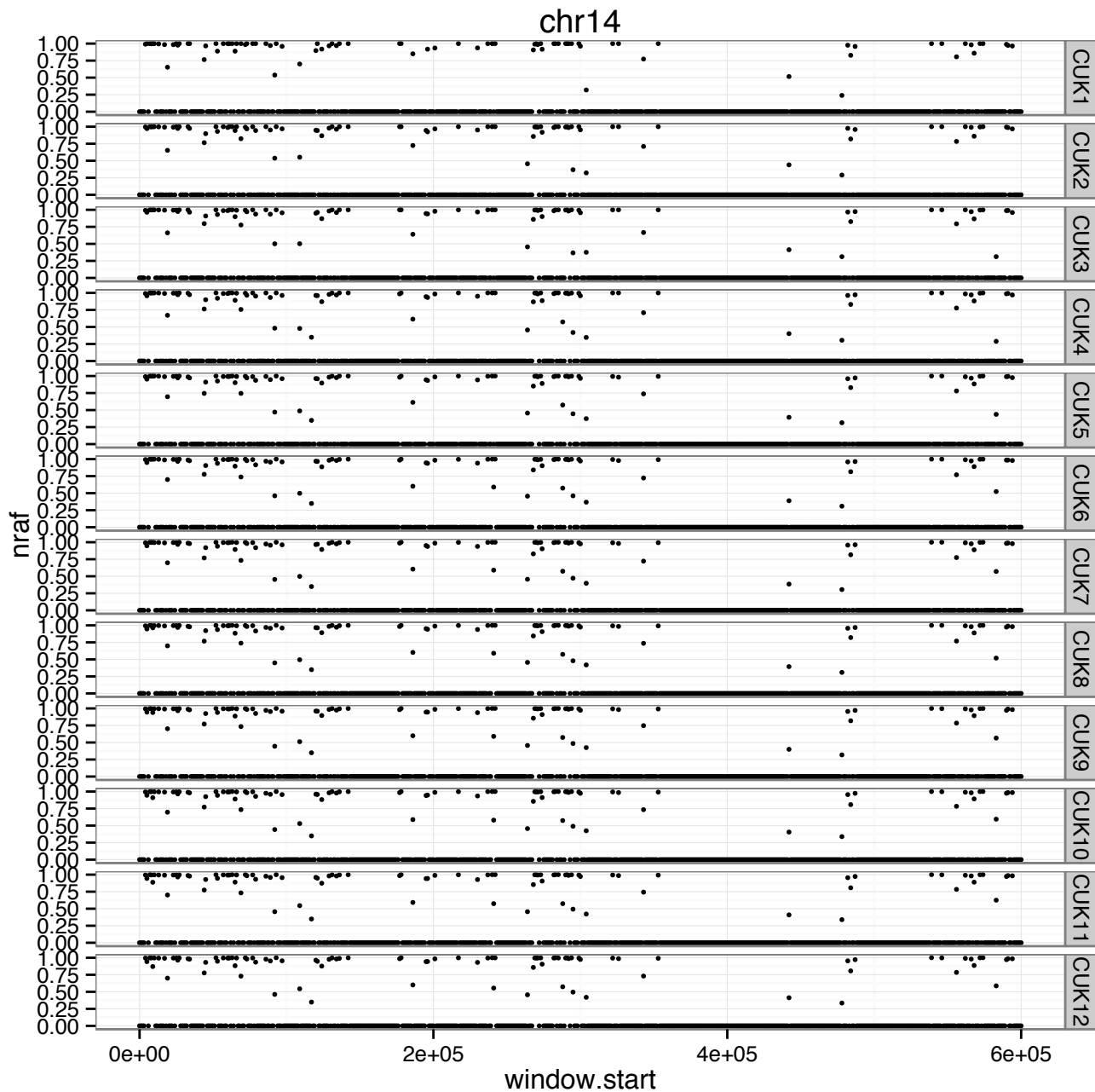

chr15

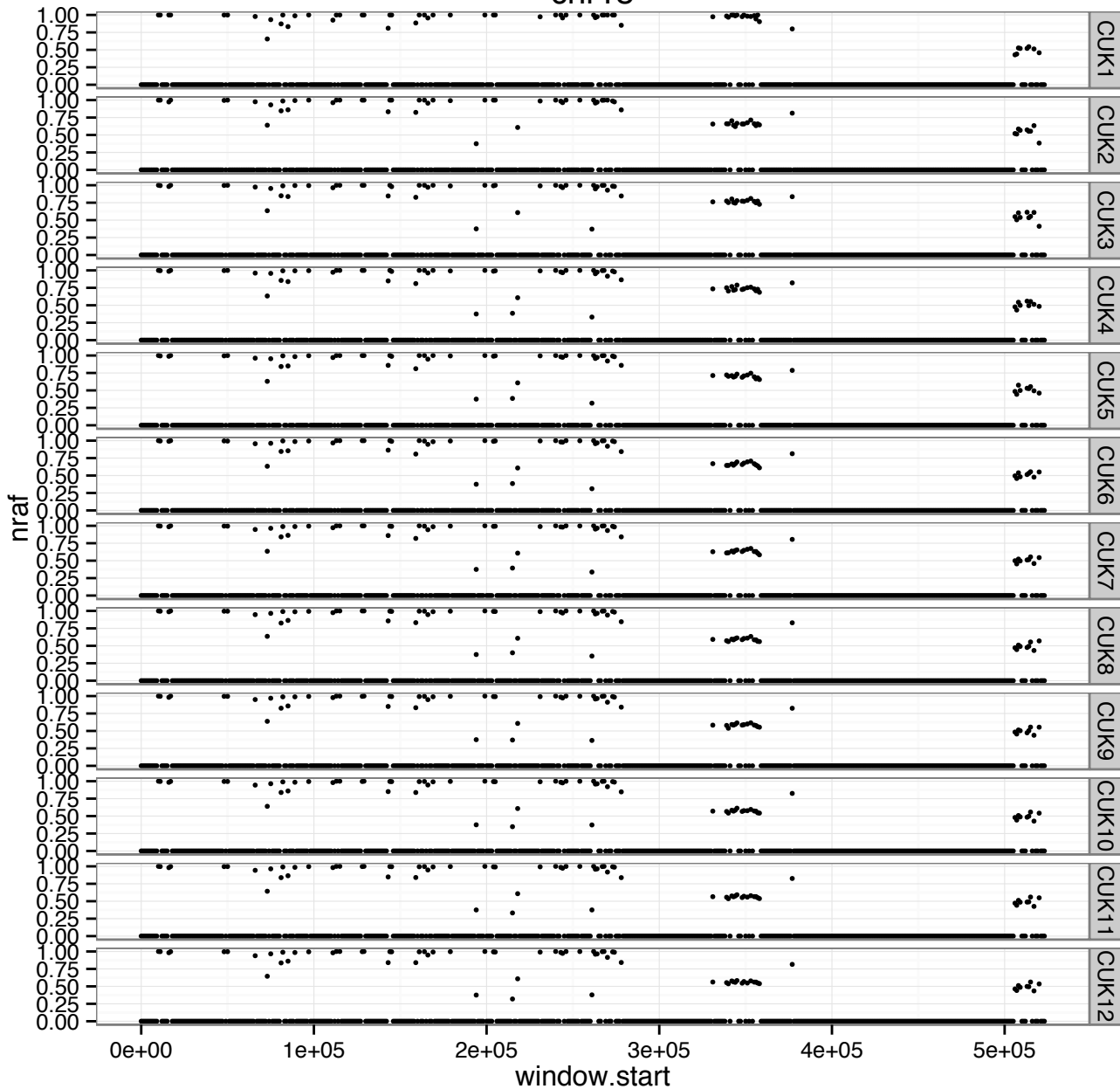

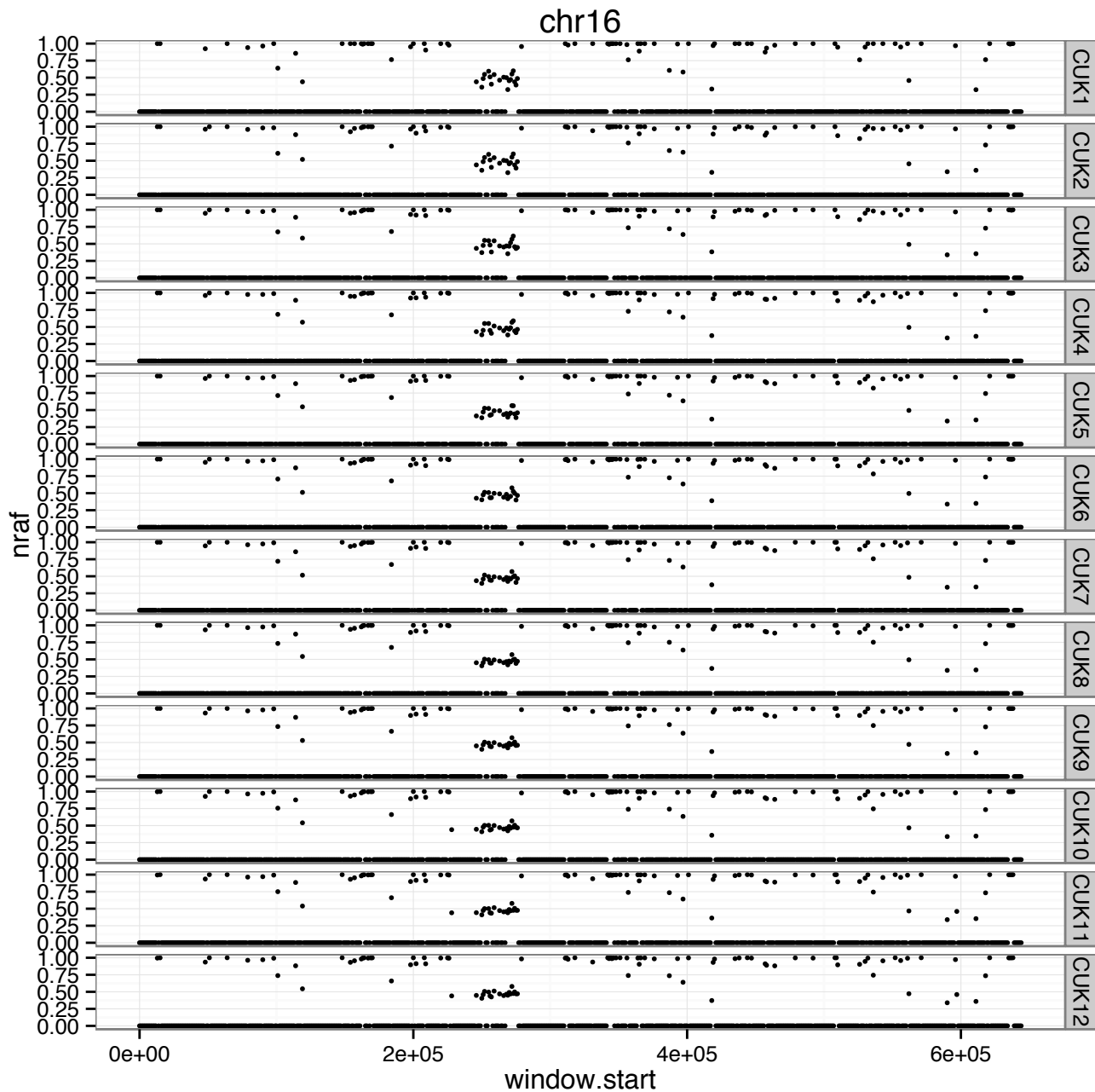

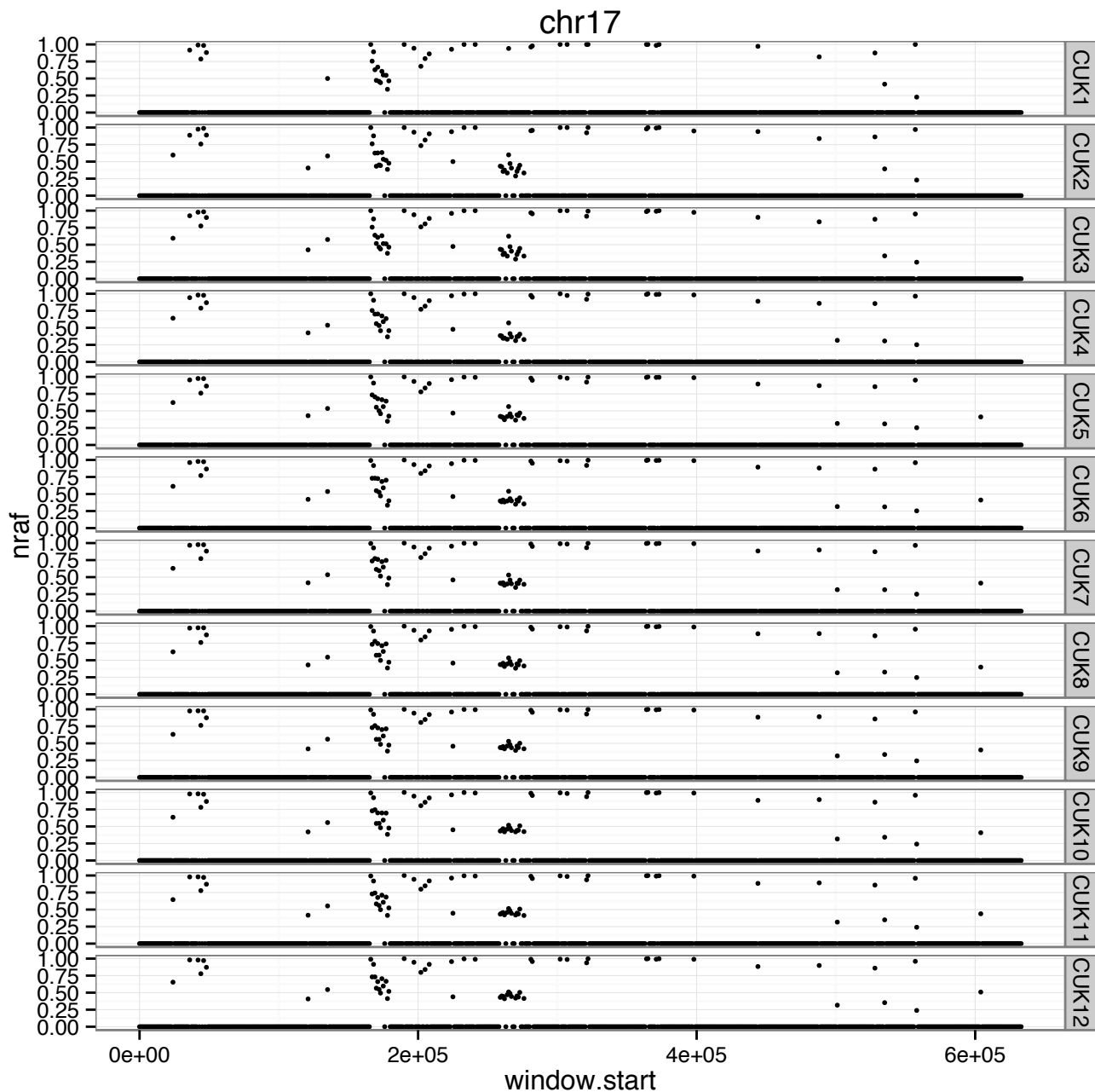

chr18

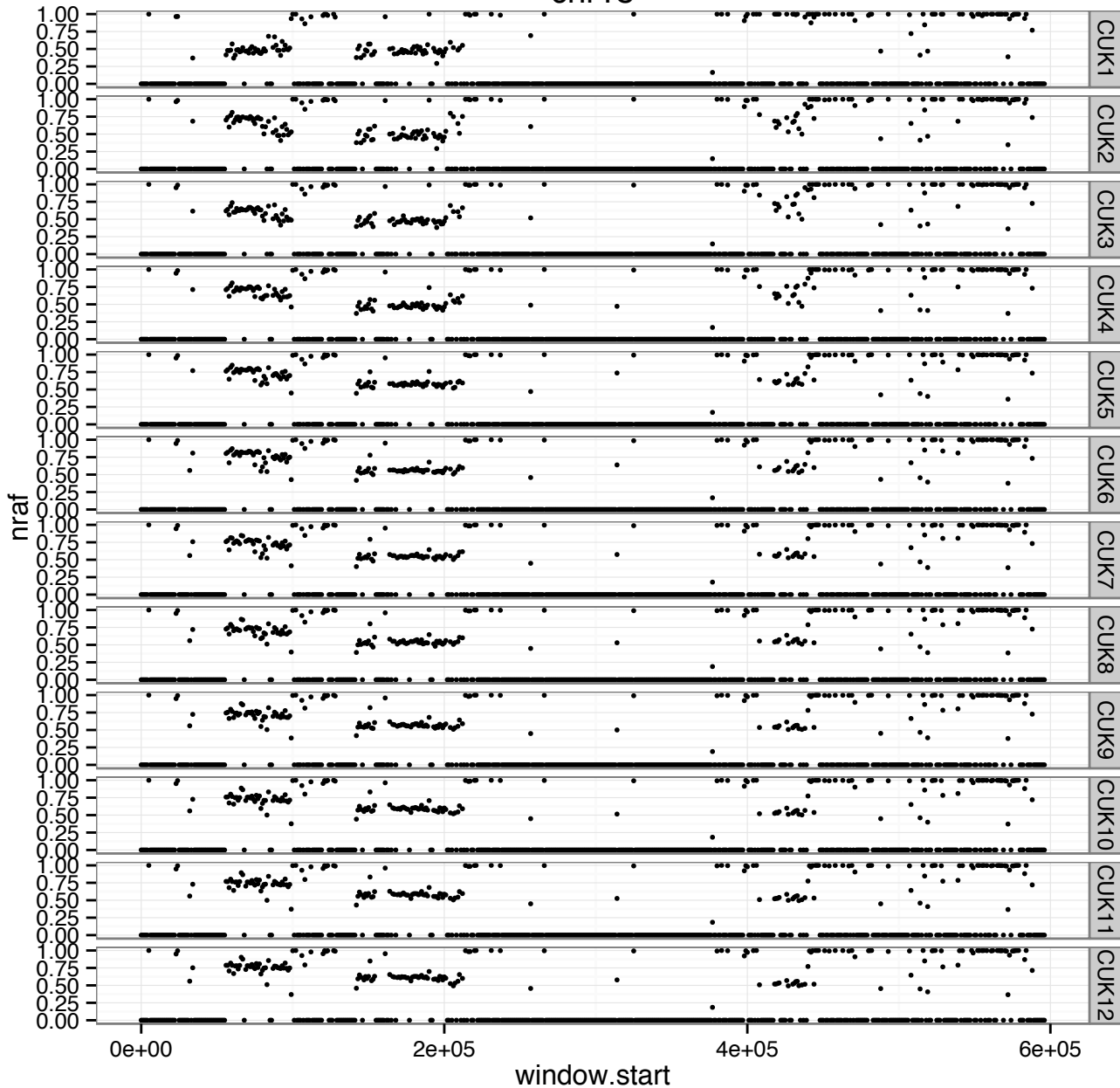

chr19

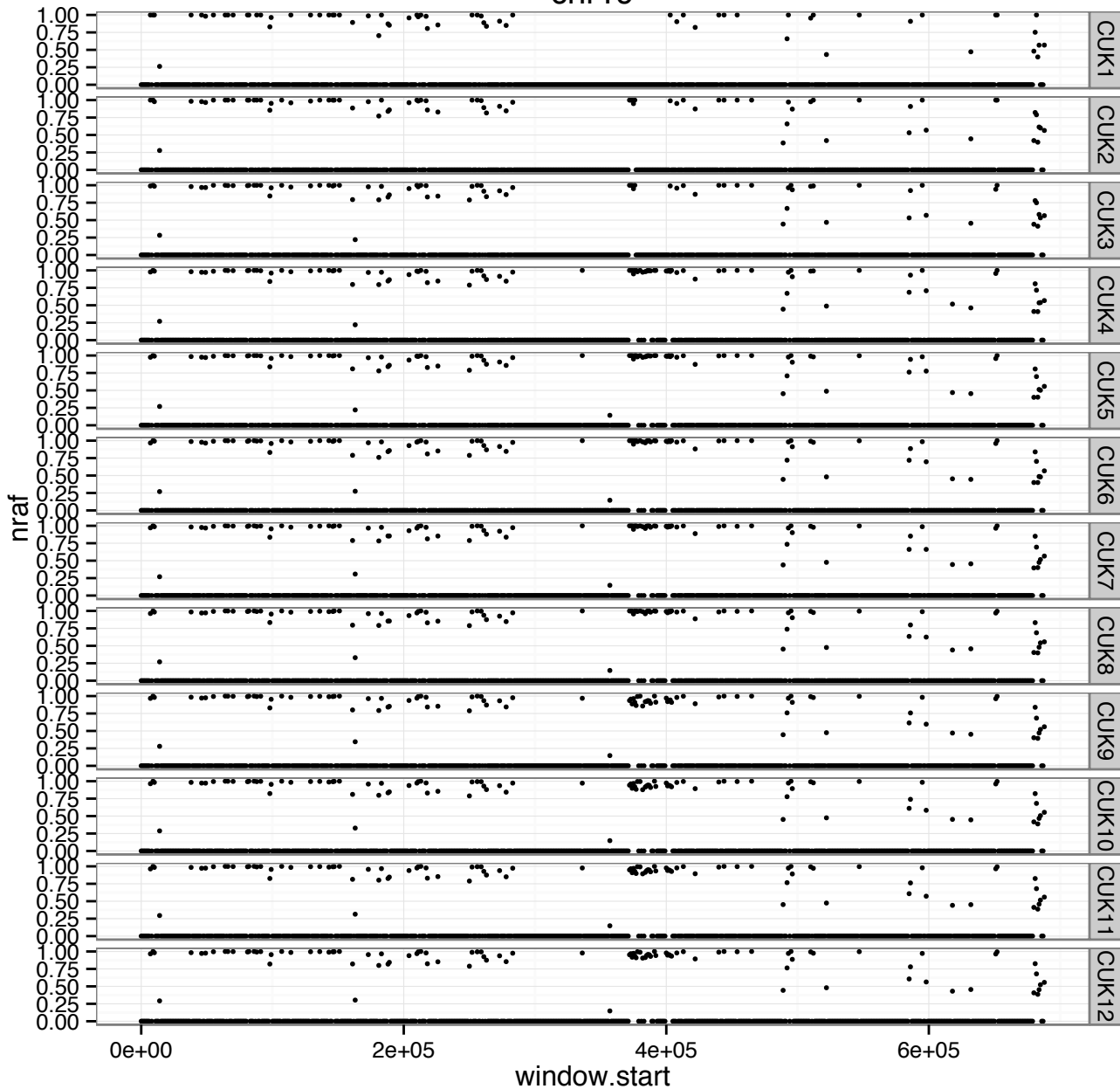

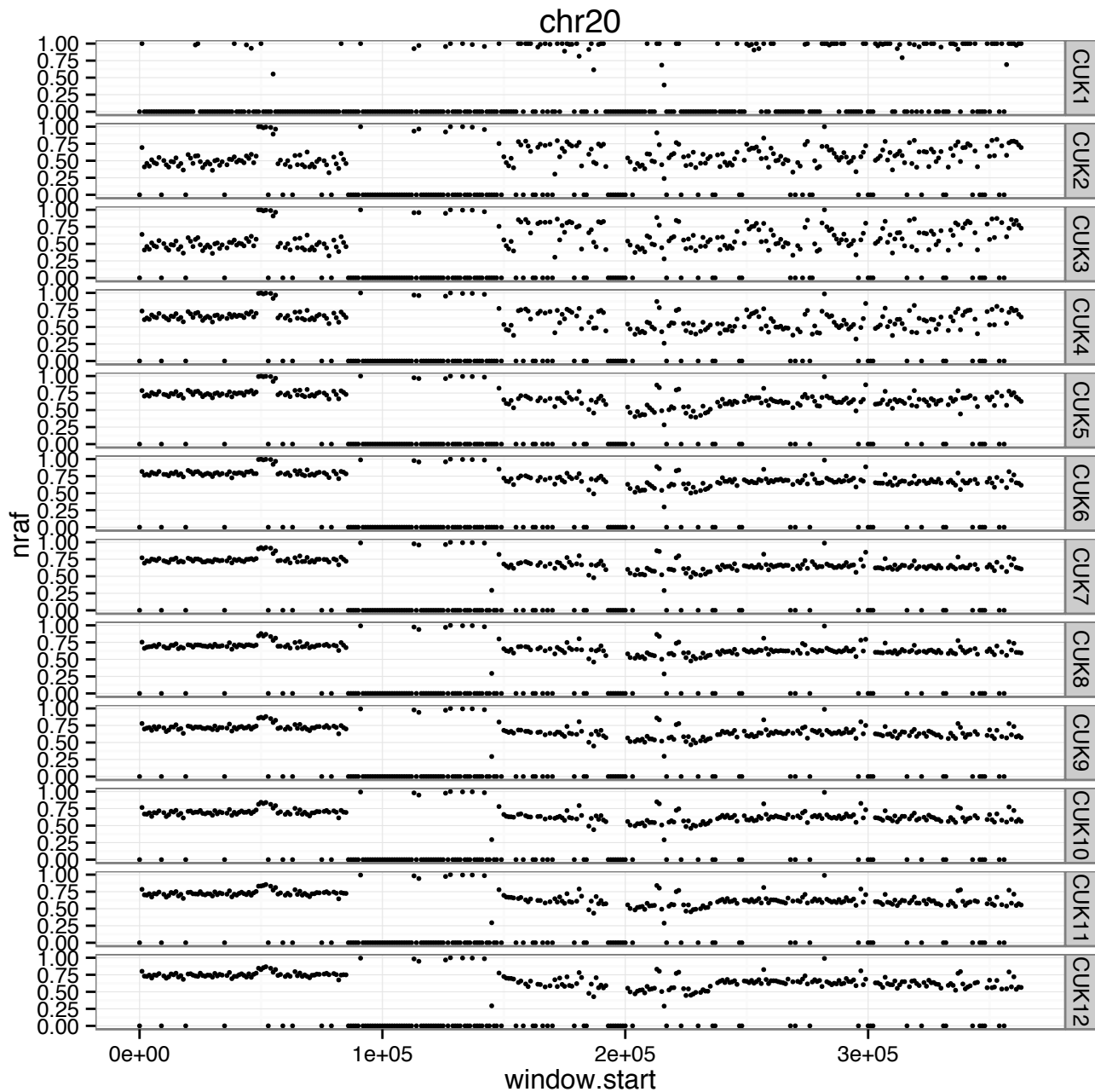

chr21

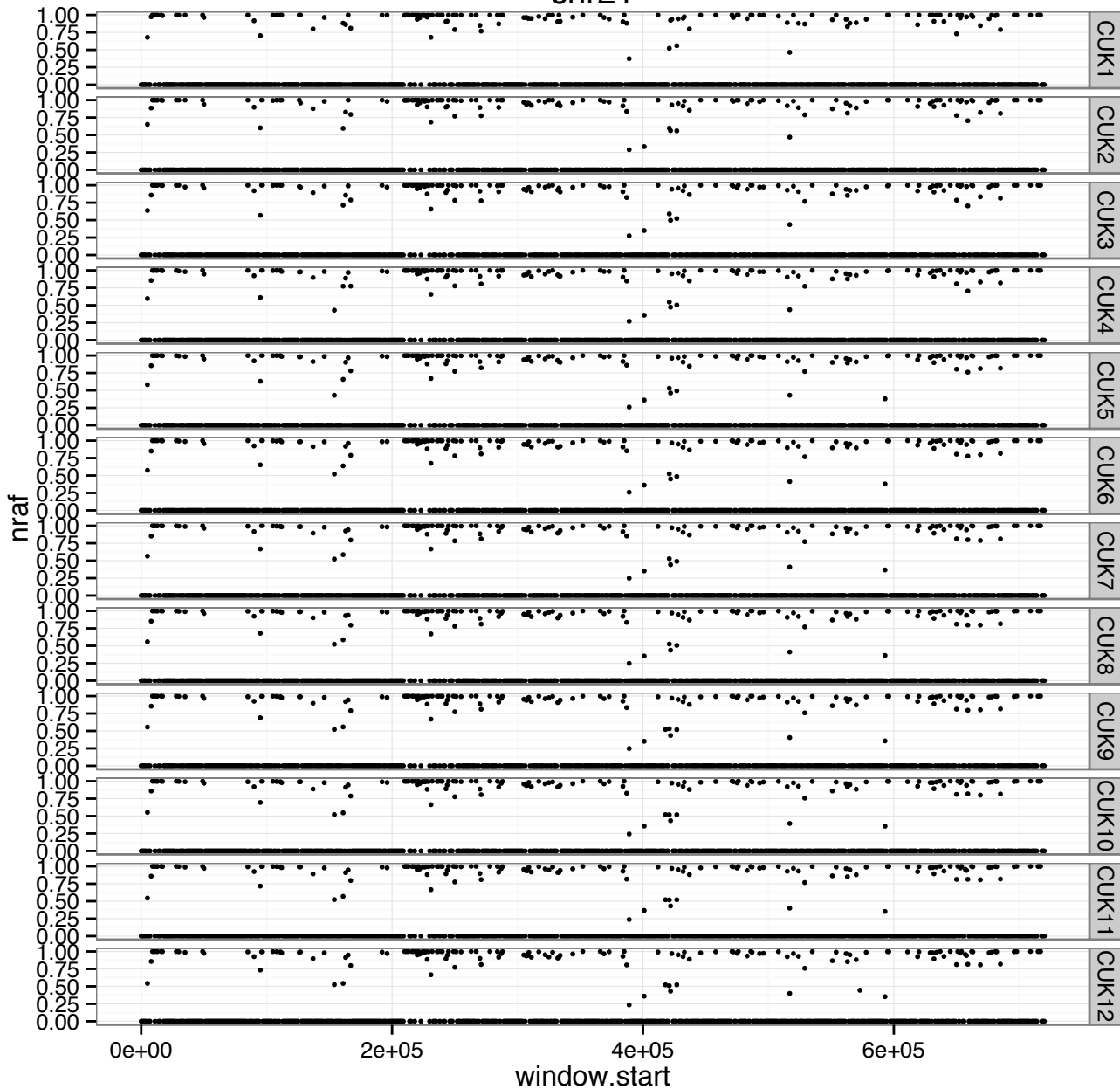

chr22

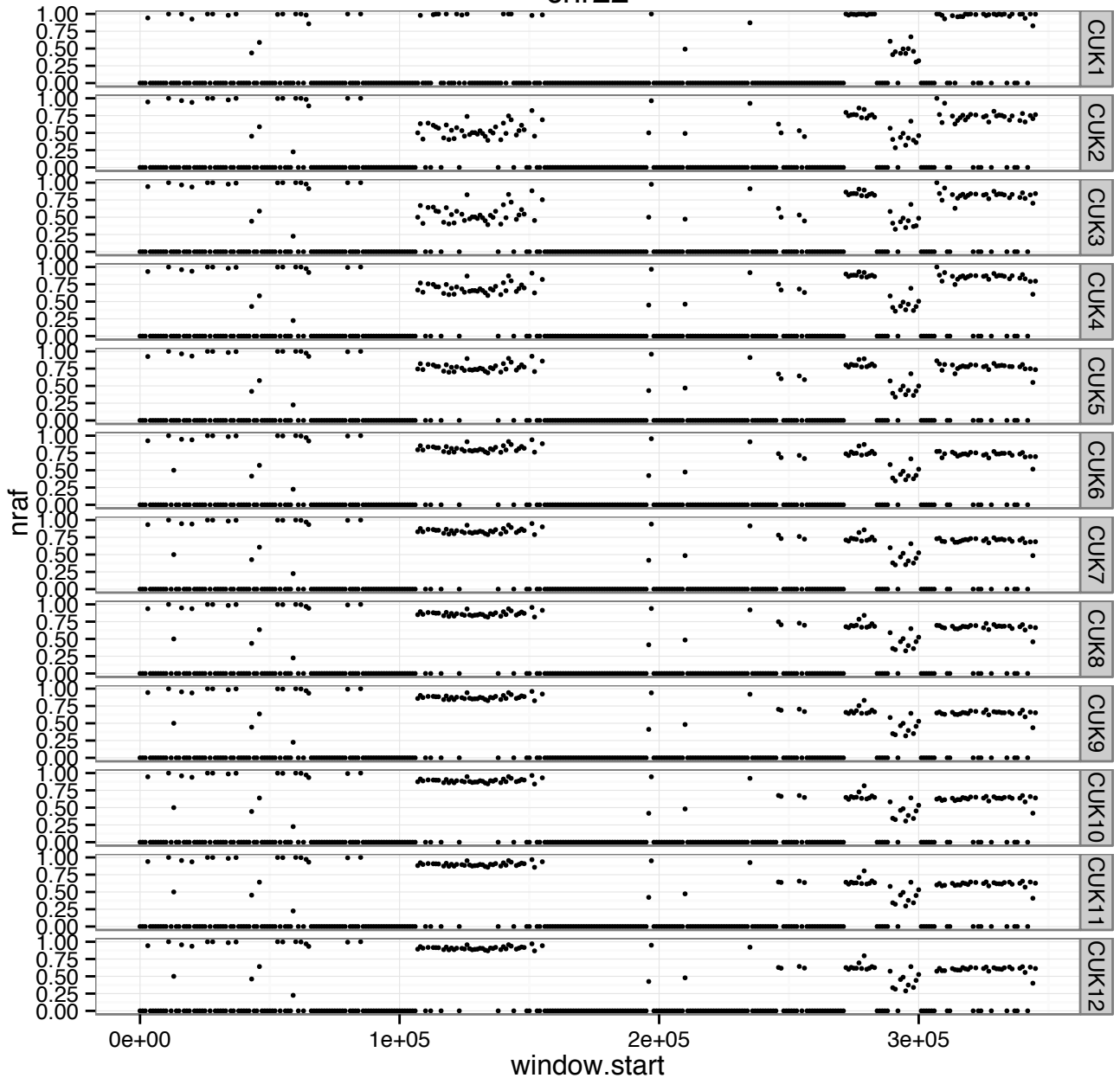

chr23

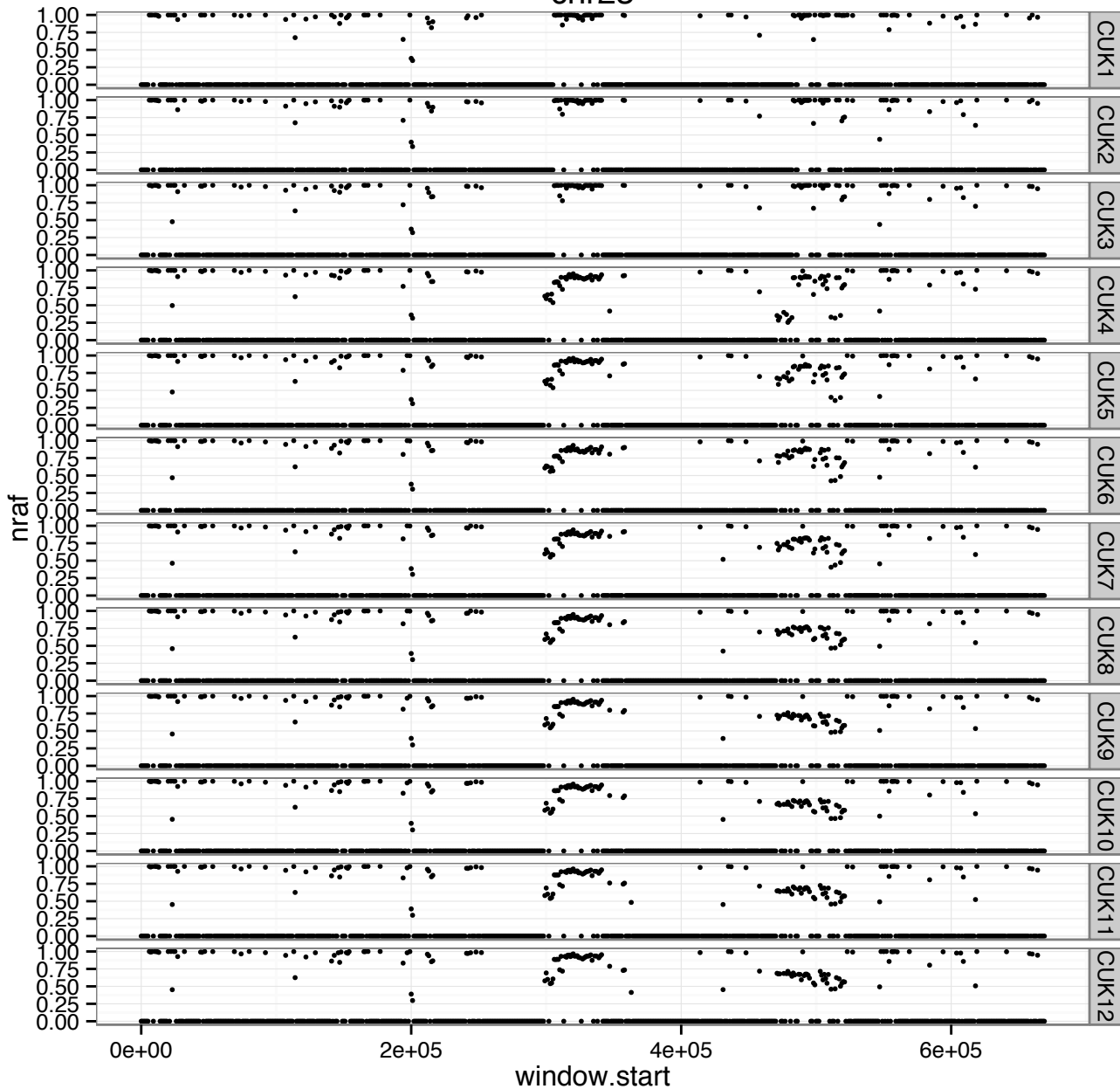

chr24

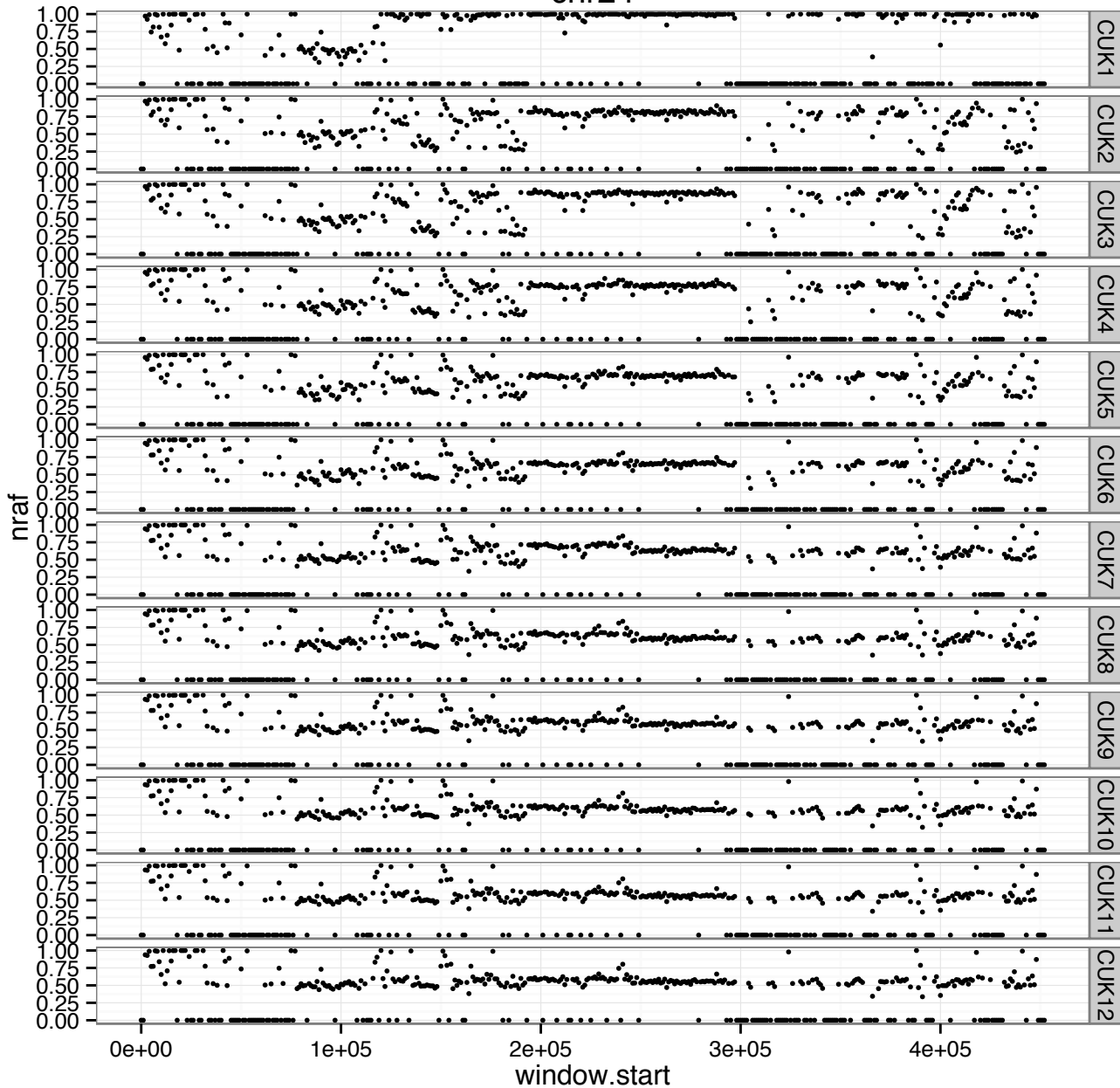

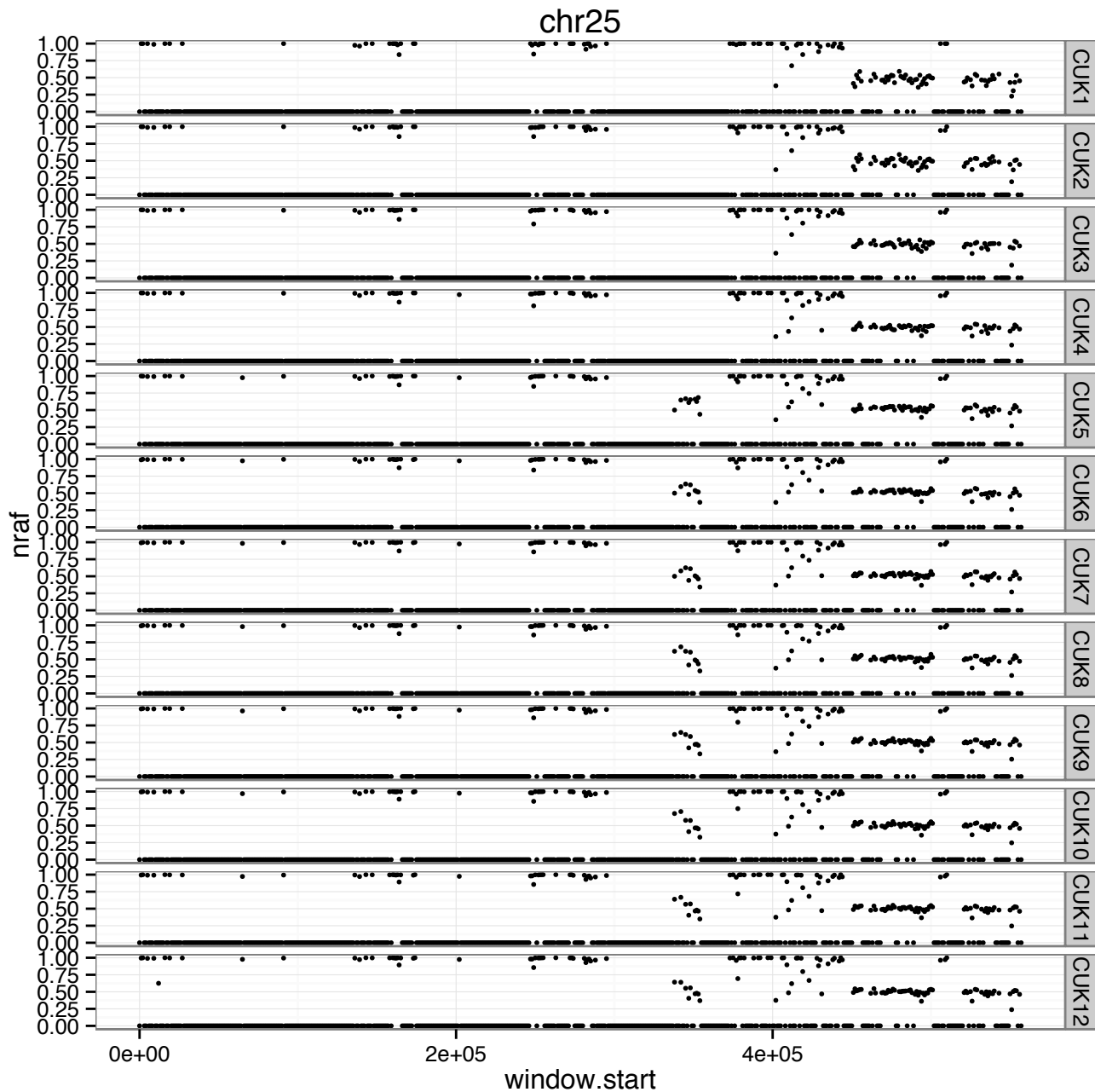

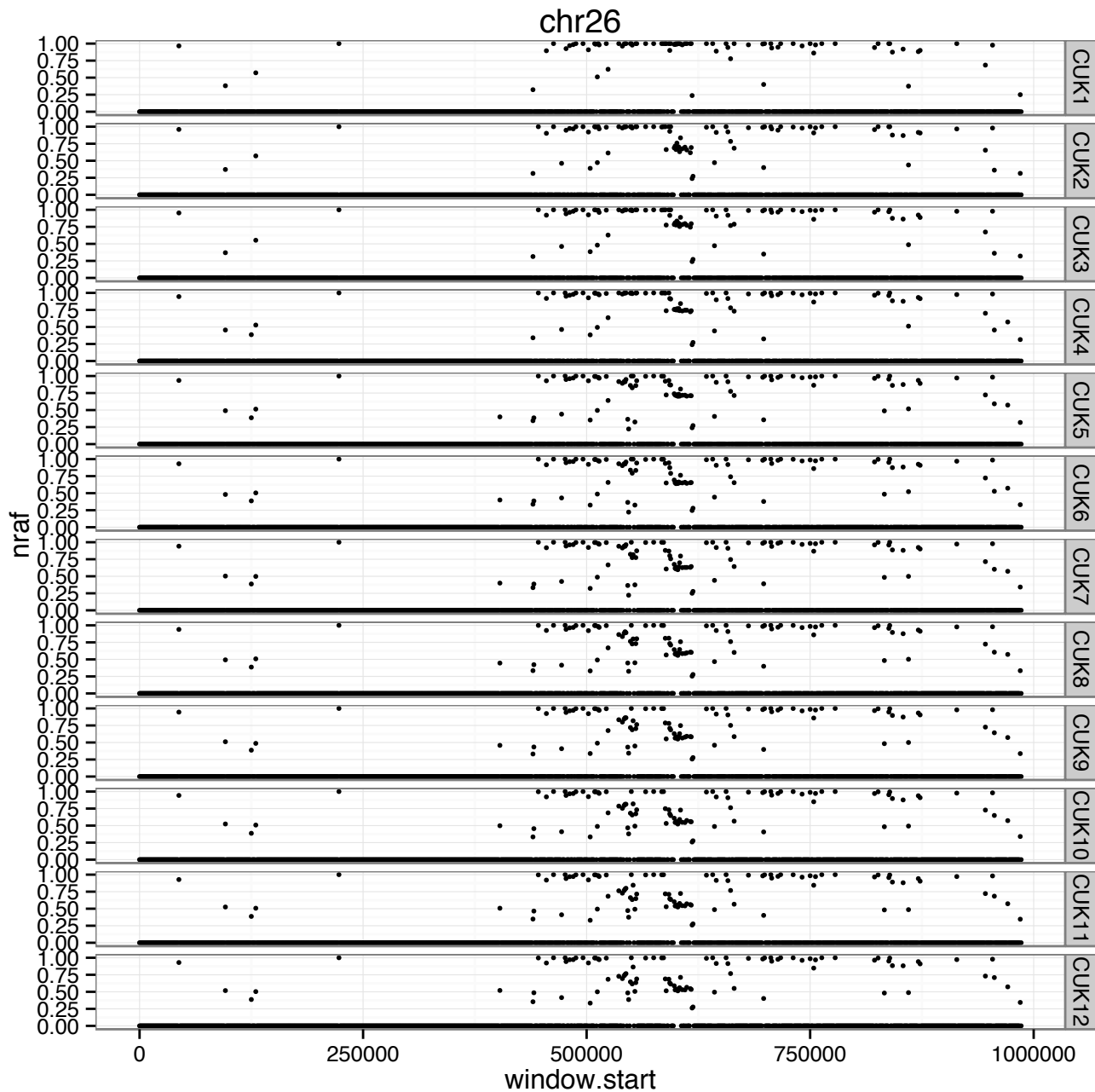

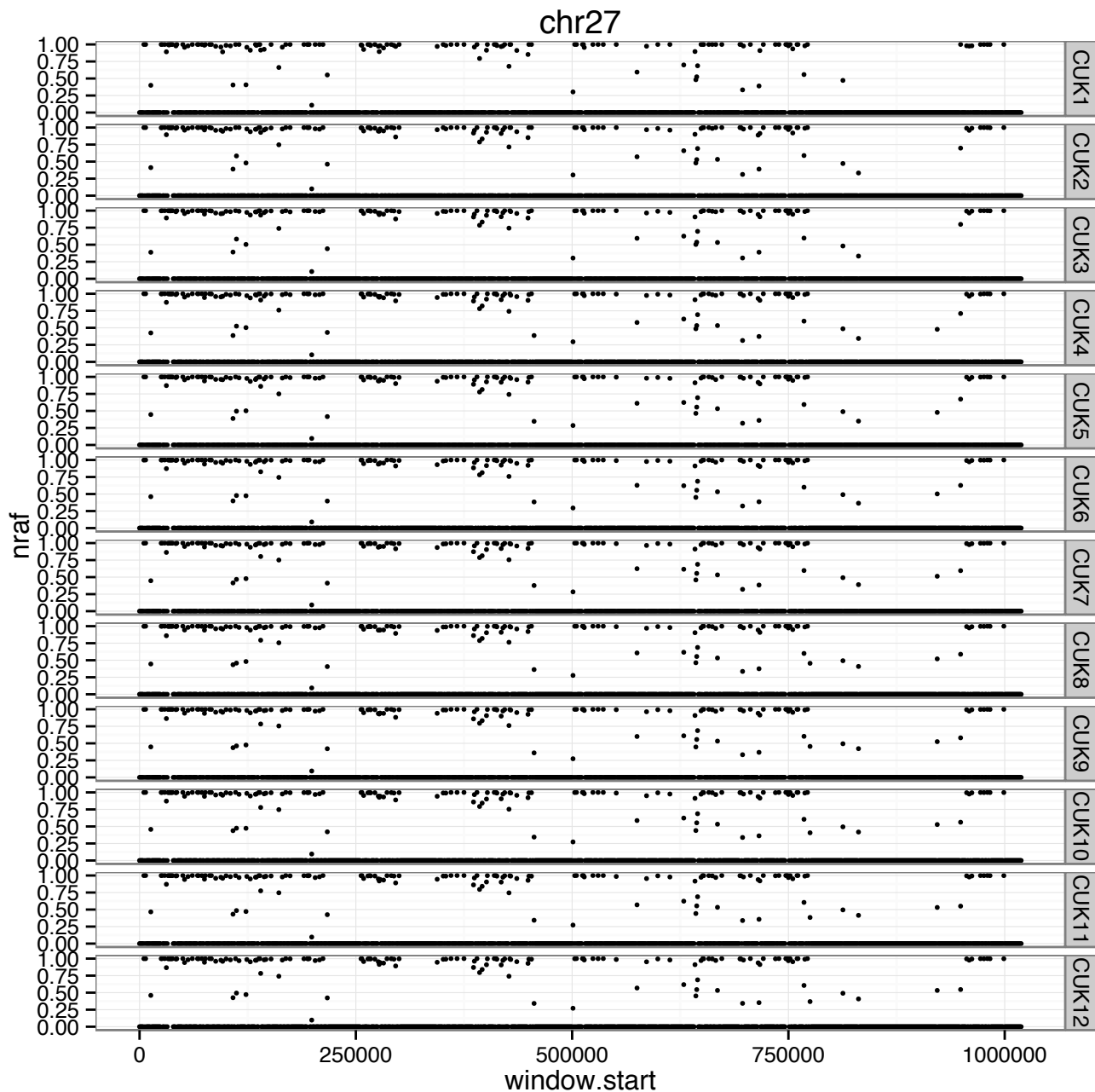

chr28

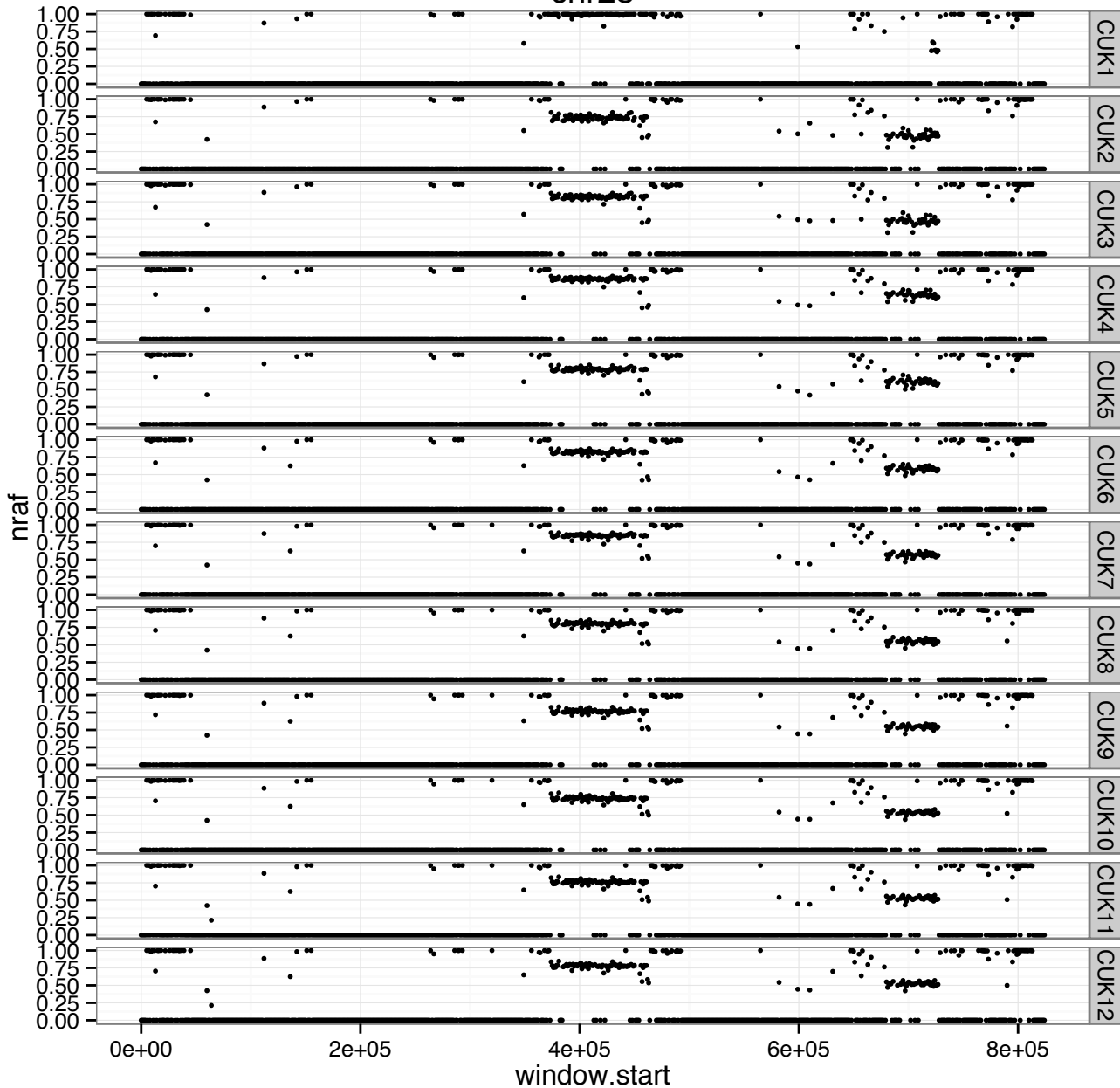

chr29

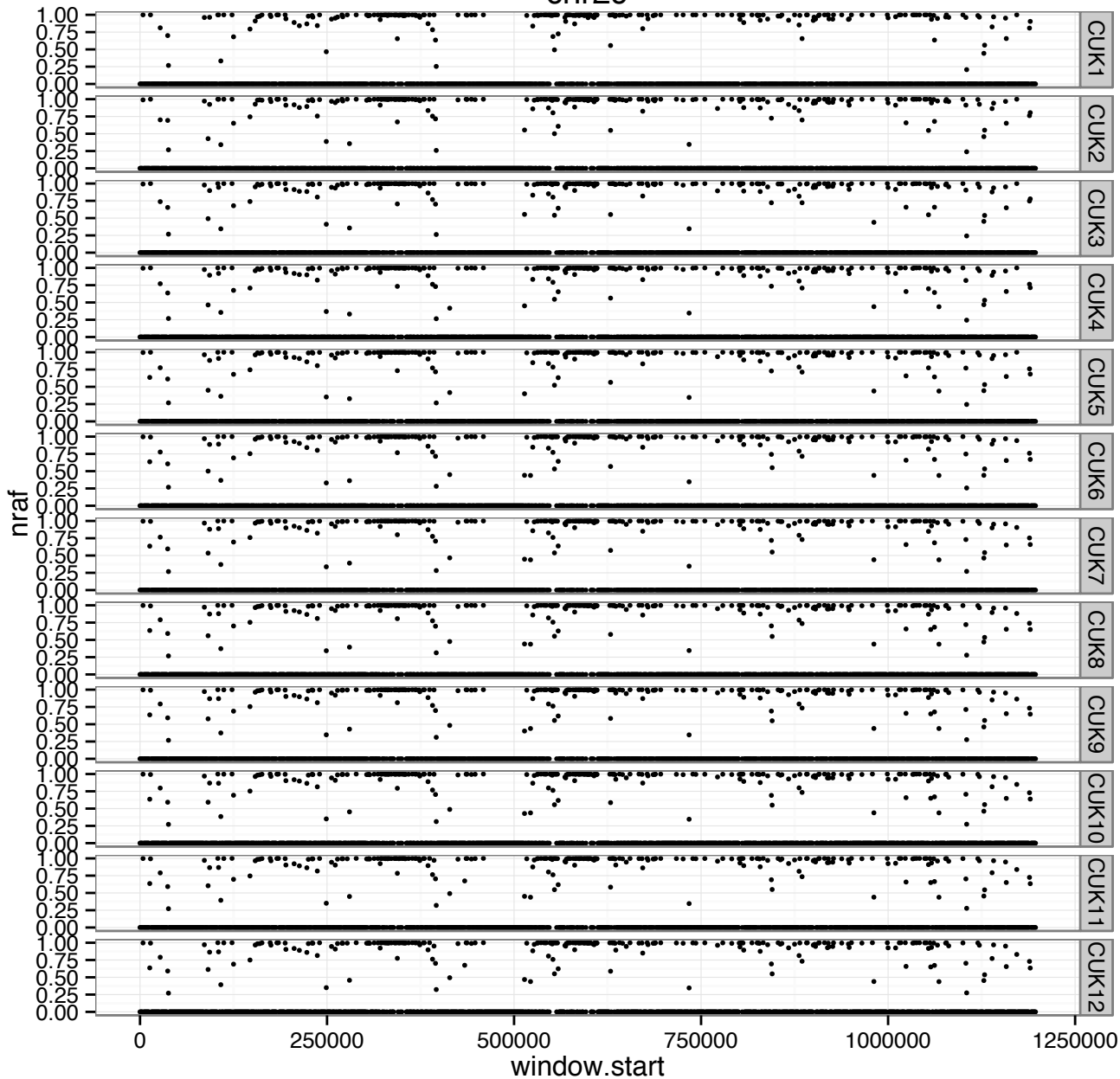

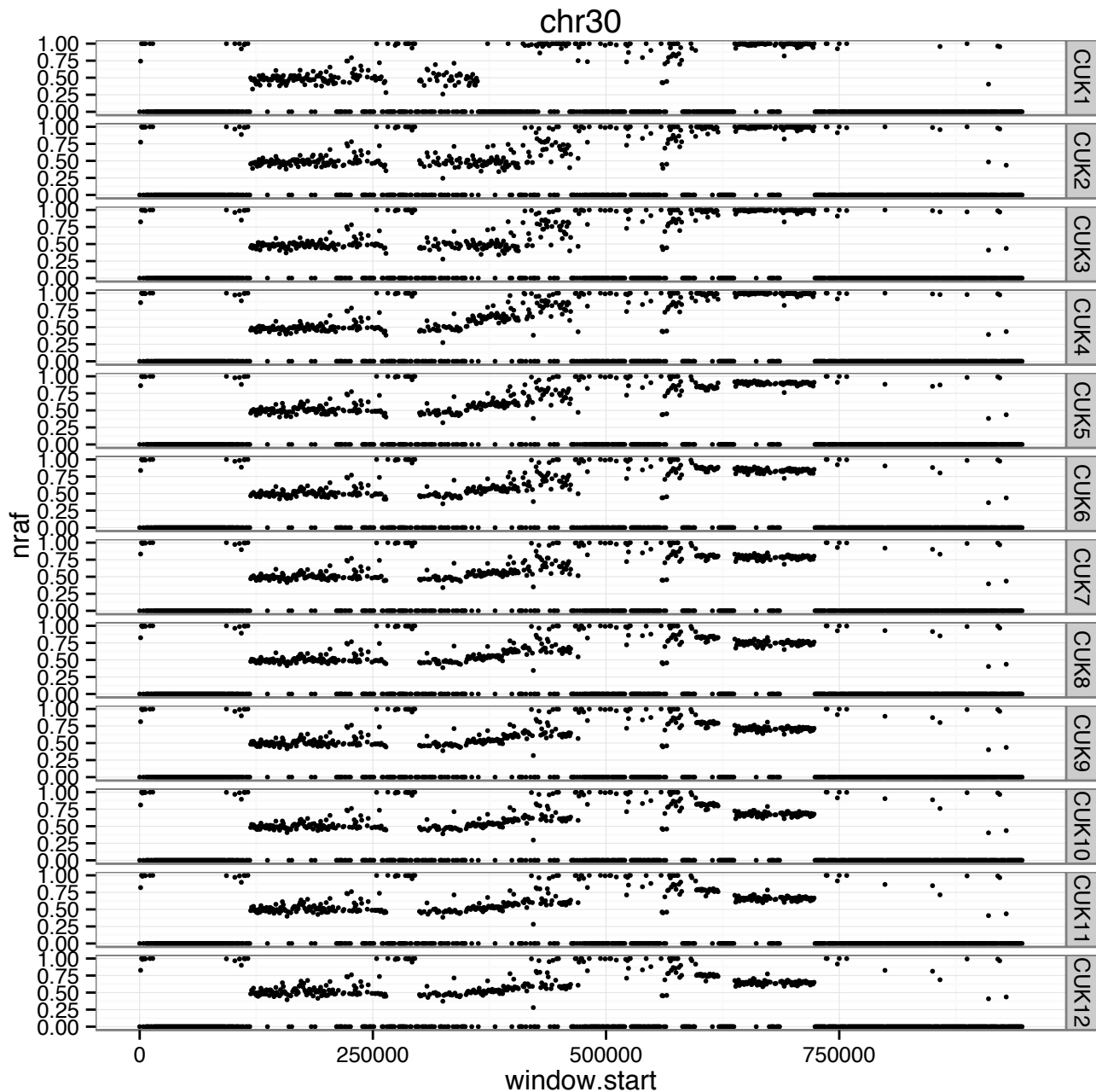

chr31

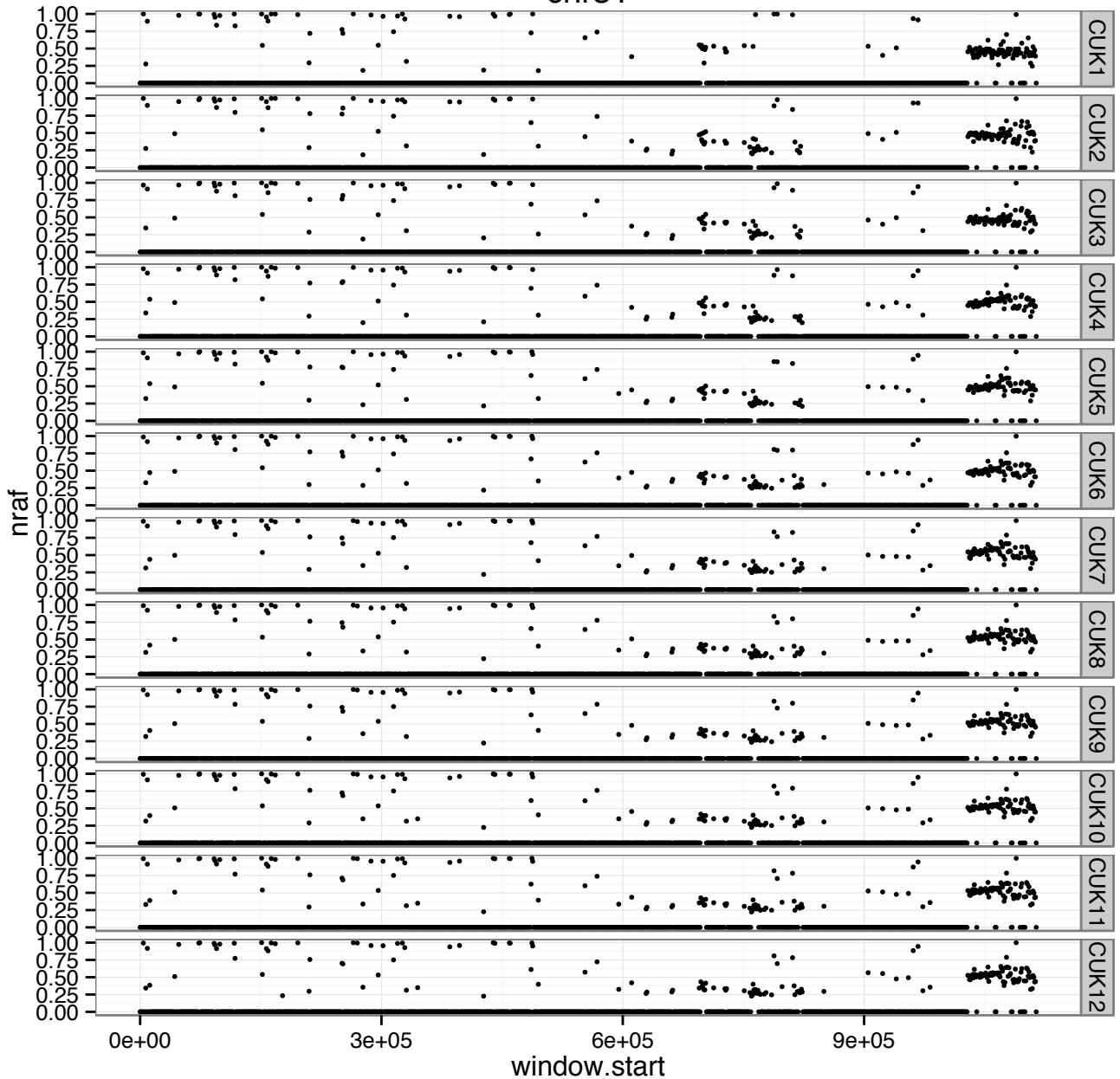

chr32

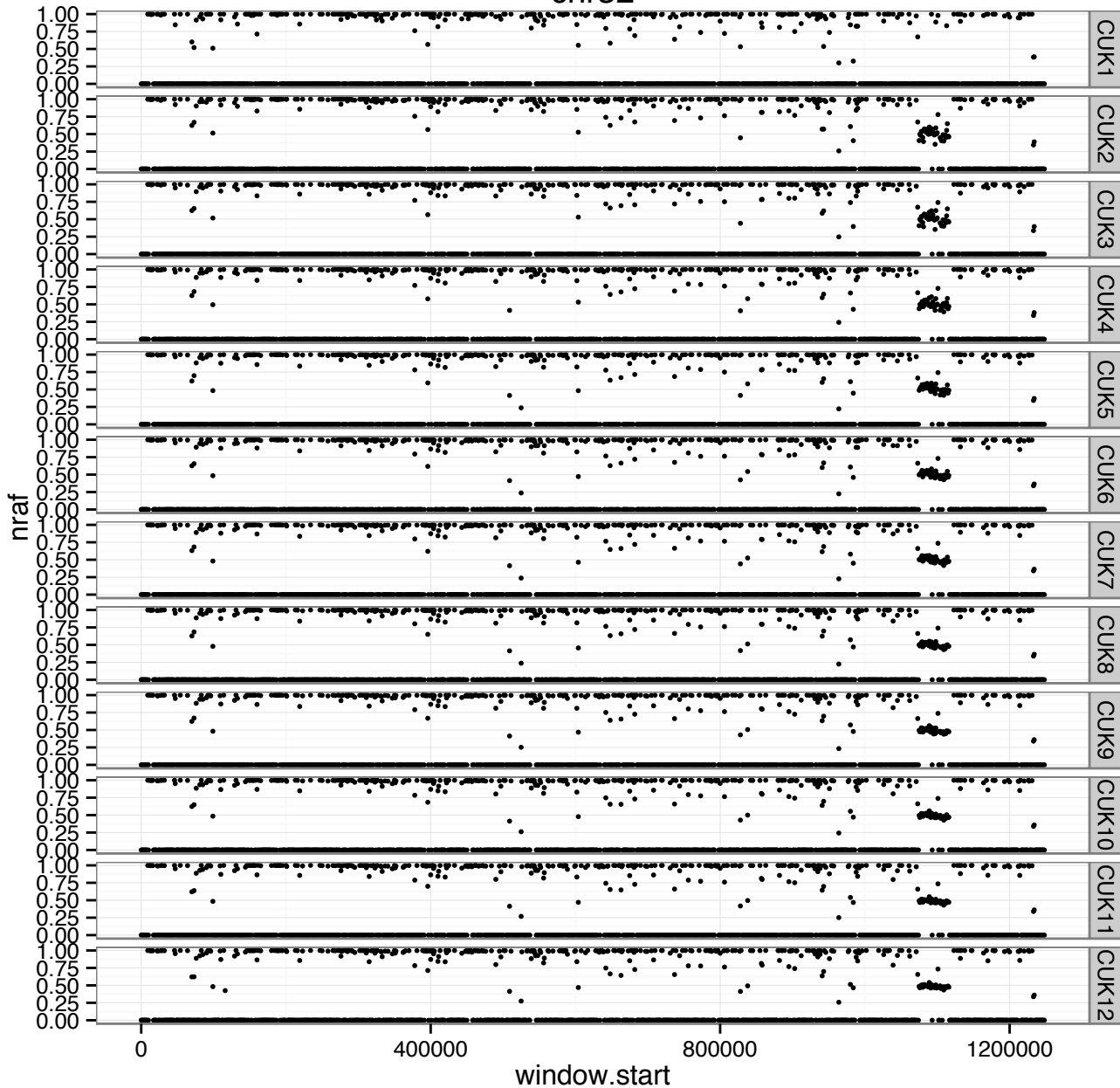

chr33

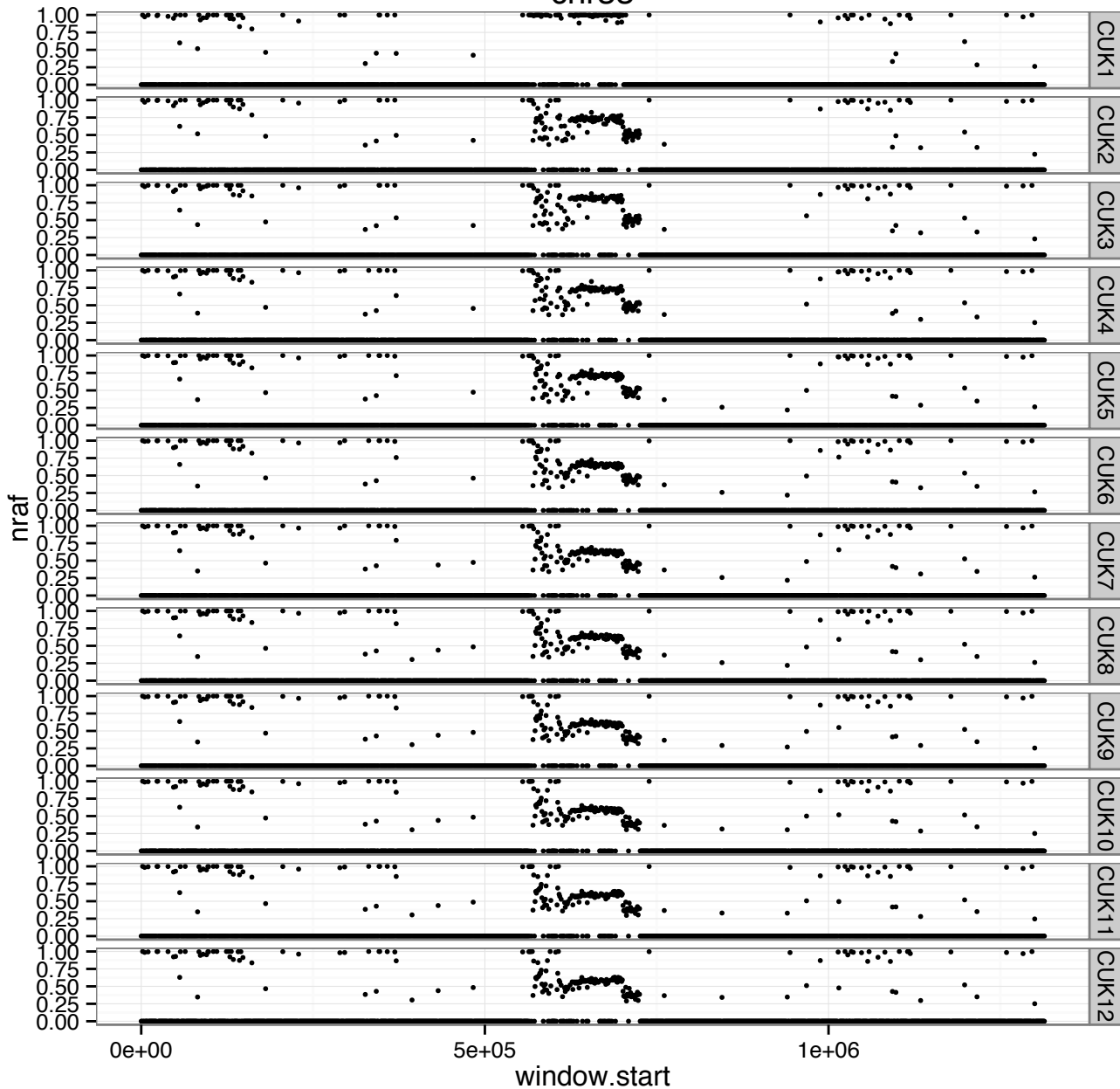

chr34

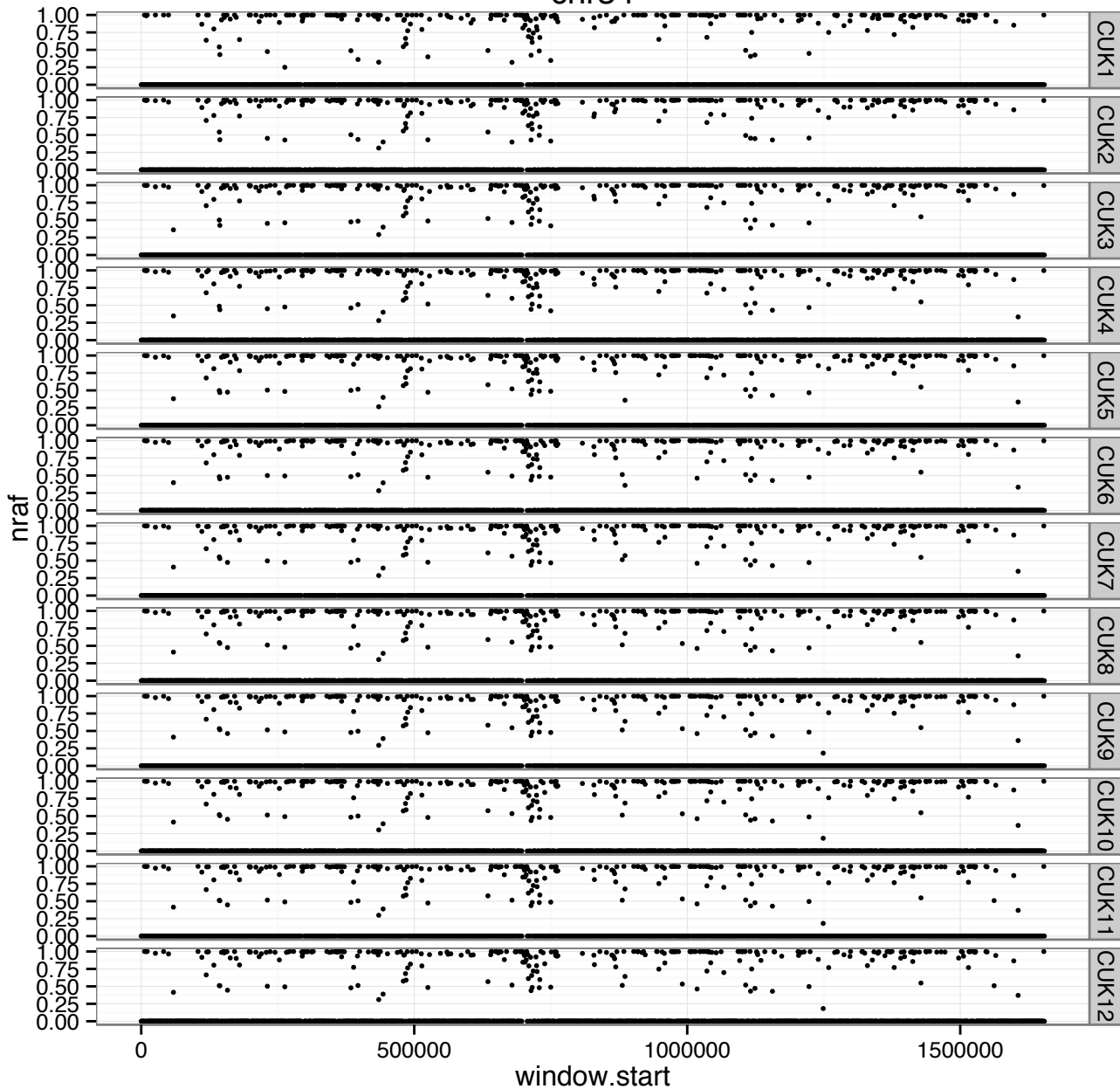

chr35

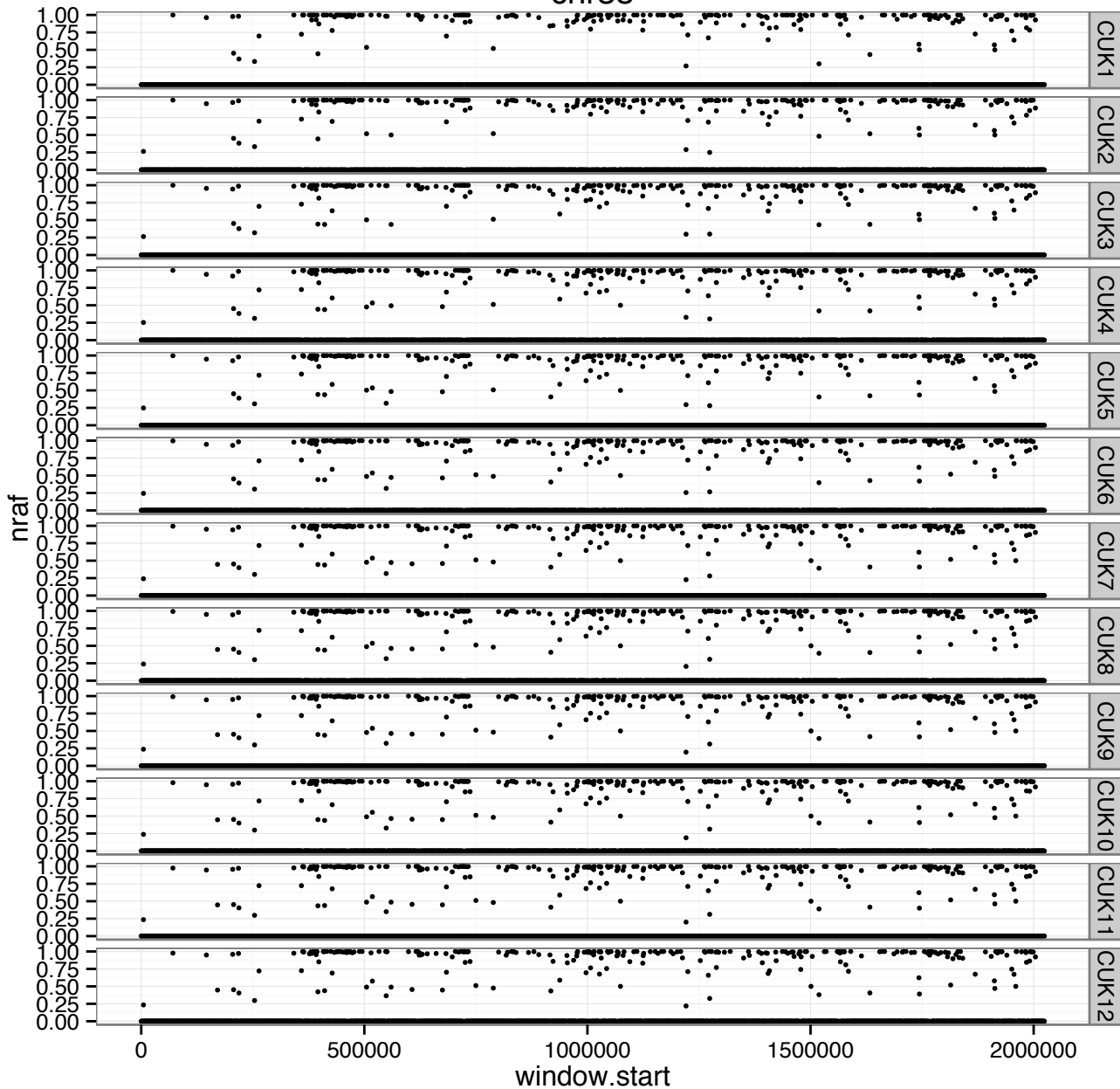

chr36

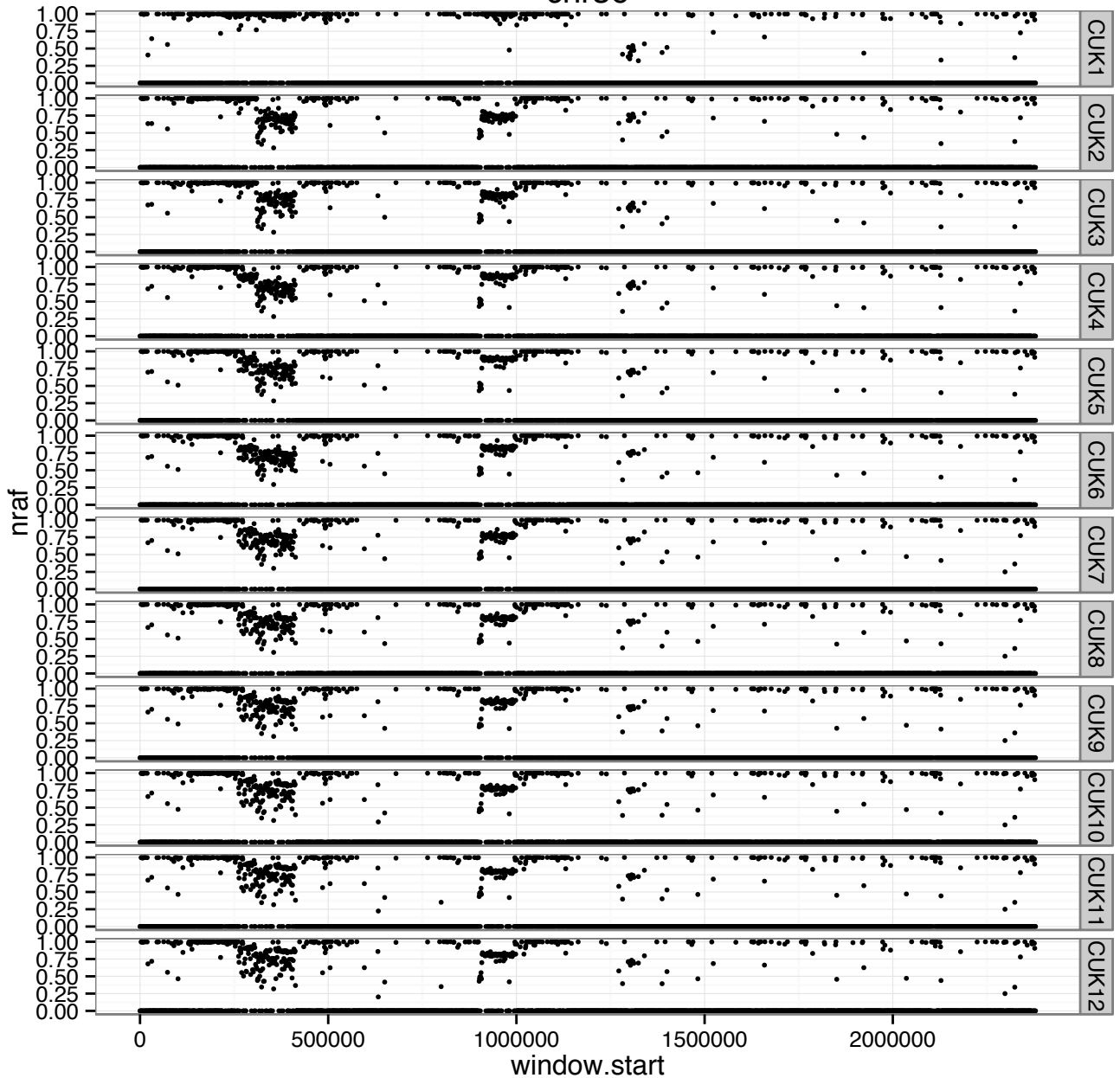

Supplement: Figure S5 — Non-reference allele frequencies for each chromosome. Plots represent the mean frequency of non-JPCM5 alleles at variable sites for each 1 kb window across each chromosome for the 12 CUK isolates. (PDF) [file pgen.1004092.s005.pdf]

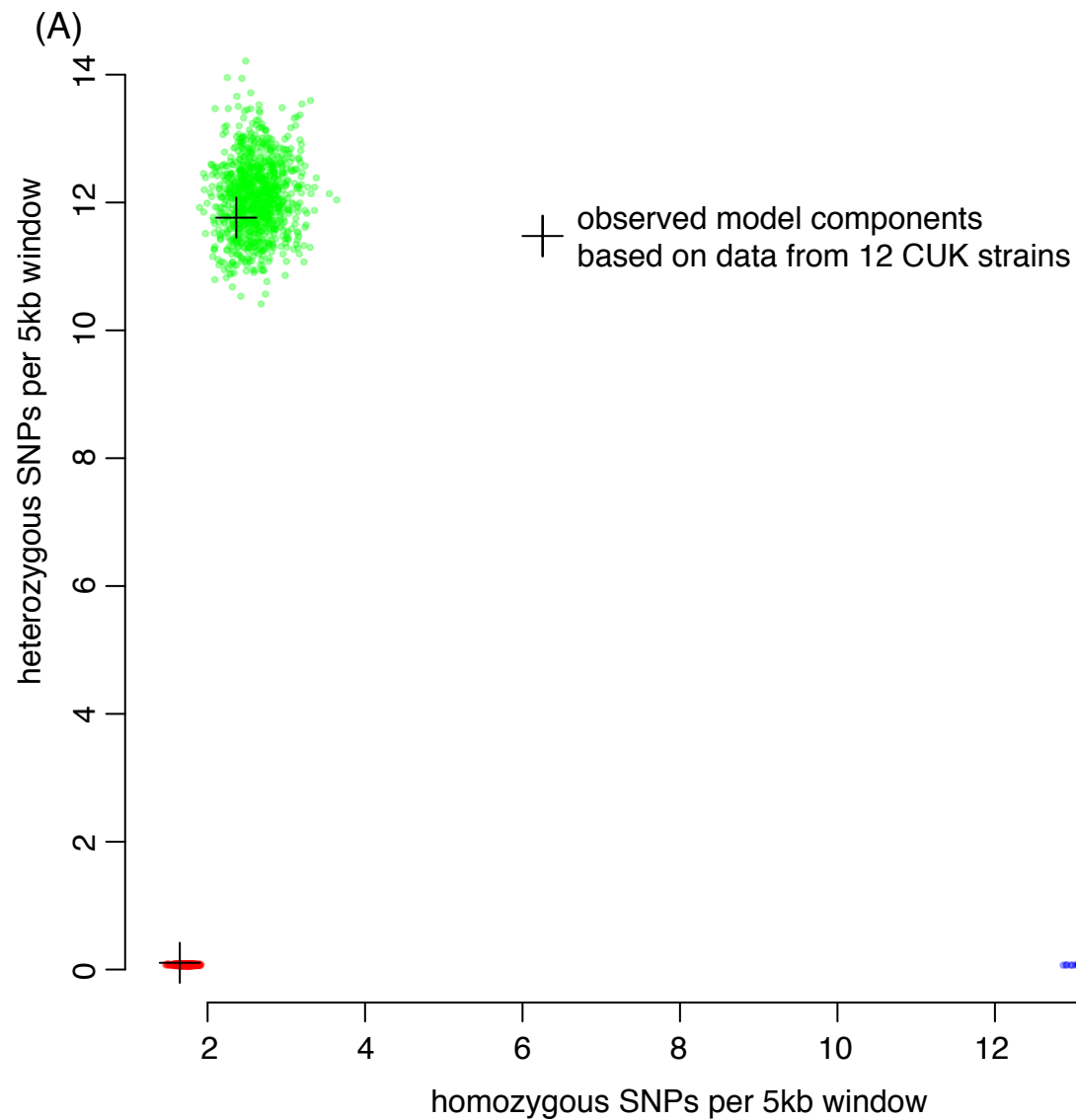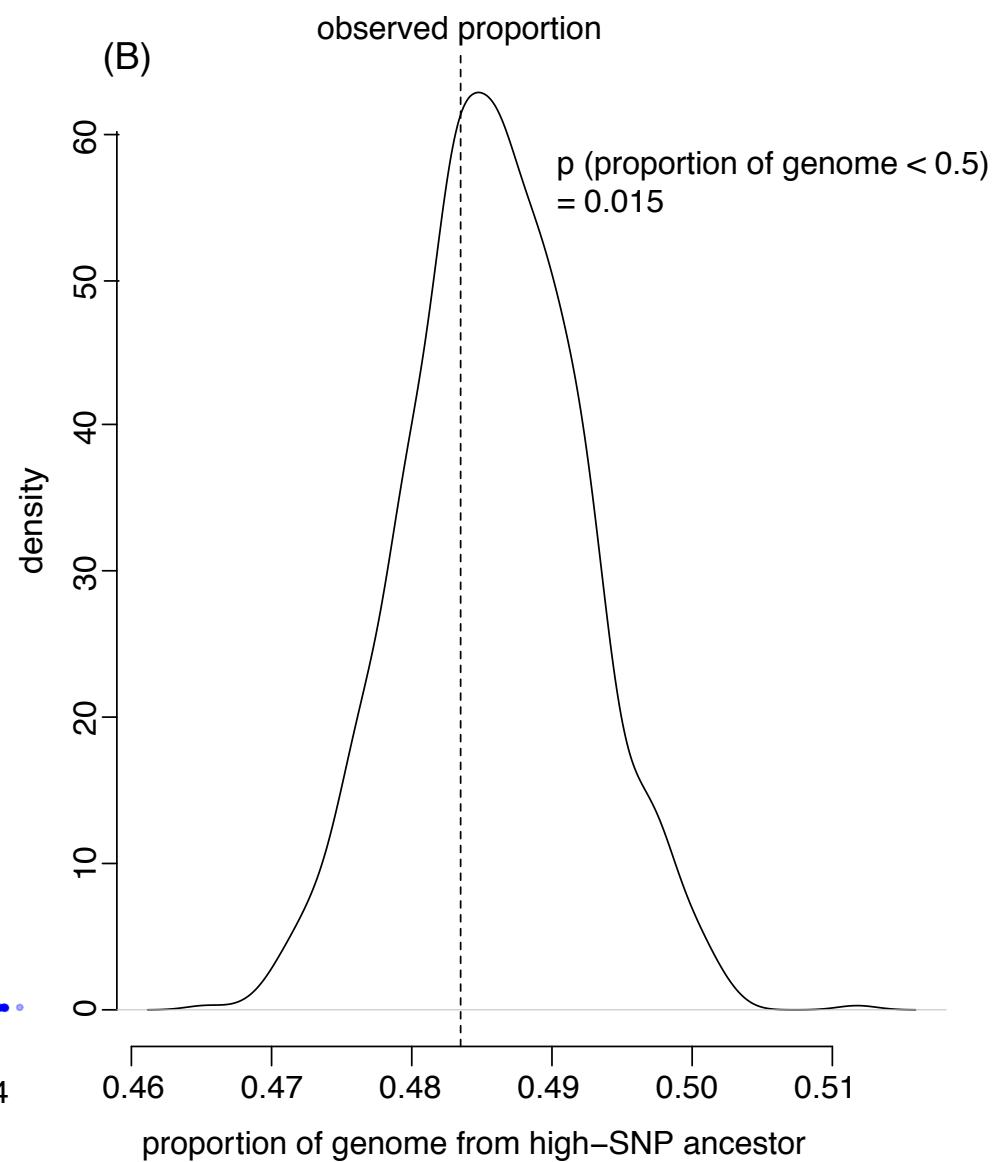

Supplement: Figure S6 — Non-parametric bootstrap analysis of mixture model parameters and cluster assignments. (A) Uncertainty in parameter estimates based on bootstrap replicates of CUK1 for the three model components, confirming that classification into high- and low- homozygous SNP and high-heterozygous SNP components is robust. Coloured dots represent parameter estimates for components of bootstrap replicates, crosses indicate parameter estimates based on observed data. Note that the points represent uncertainty in the centres of each component, and so are more compact that the probability distributions for each component shown in Figure S17(A) with a different scale for the heterozygous SNPs per window (y-axis). (B) Distribution of bootstrap estimates of the proportion of the CUK1 genome derived from the ‘low SNP density’ ancestor, based on maximum posterior probability assignment under mixture model. Dotted line indicates the combined estimate for observed data. Figures are all based on 1,000 non-parametric bootstrap replicates, resampling randomly from the set of 5 kb genomic windows. (PDF) [file pgen.1004092.s006.pdf]

0.02

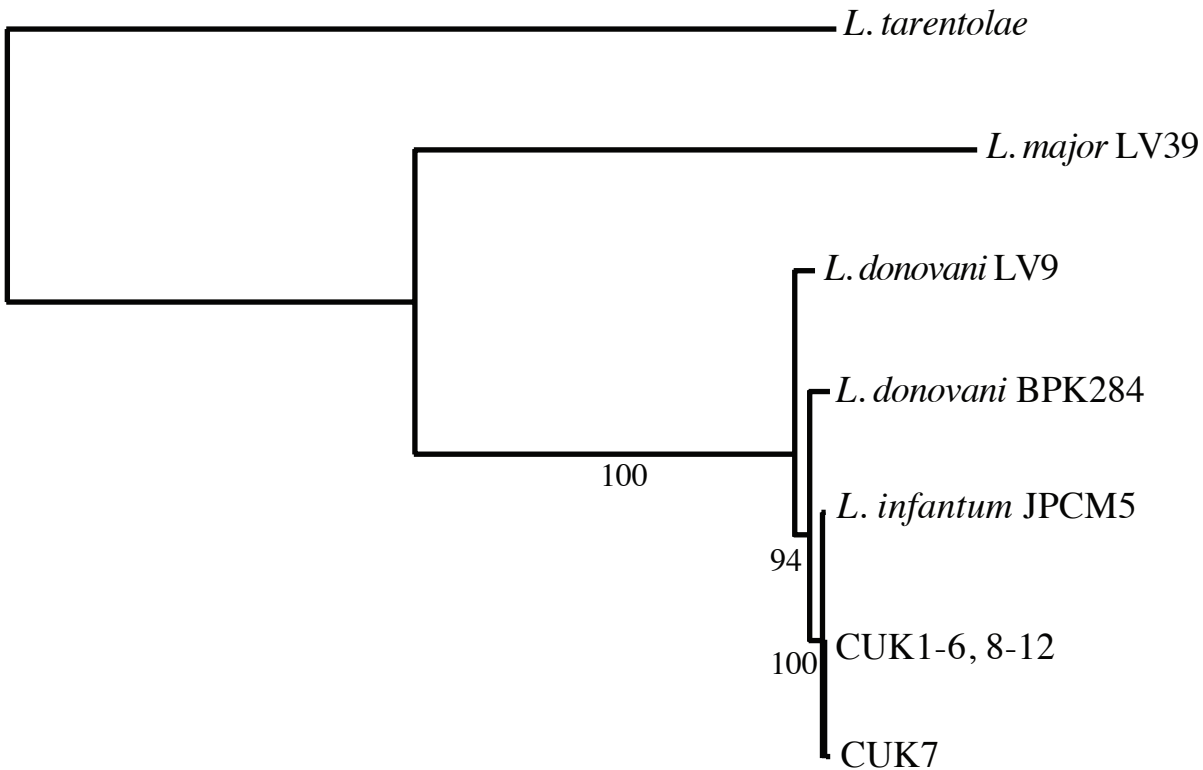

Supplement: Figure S7 — Maximum-likelihood phylogeny of assembled kDNA constant regions for the CUK strains and sequenced L. donovani complex genomes. PHYML Maximum likelihood tree of the constant region of the kDNA shows that the kDNA of the CUK strains share a closer common ancestry with L. infantum JPCM5 than they do with L. donovani BPK282 or L. donovani LV9. L. tarentolae was chosen to root the tree as it is the most closely related outgroup to the sub-genus Leishmania for which whole-genome sequence data are available (reviewed in [72]). In addition, the kDNA maxicircle is fully assembled for this species. (PDF) [file pgen.1004092.s007.pdf]

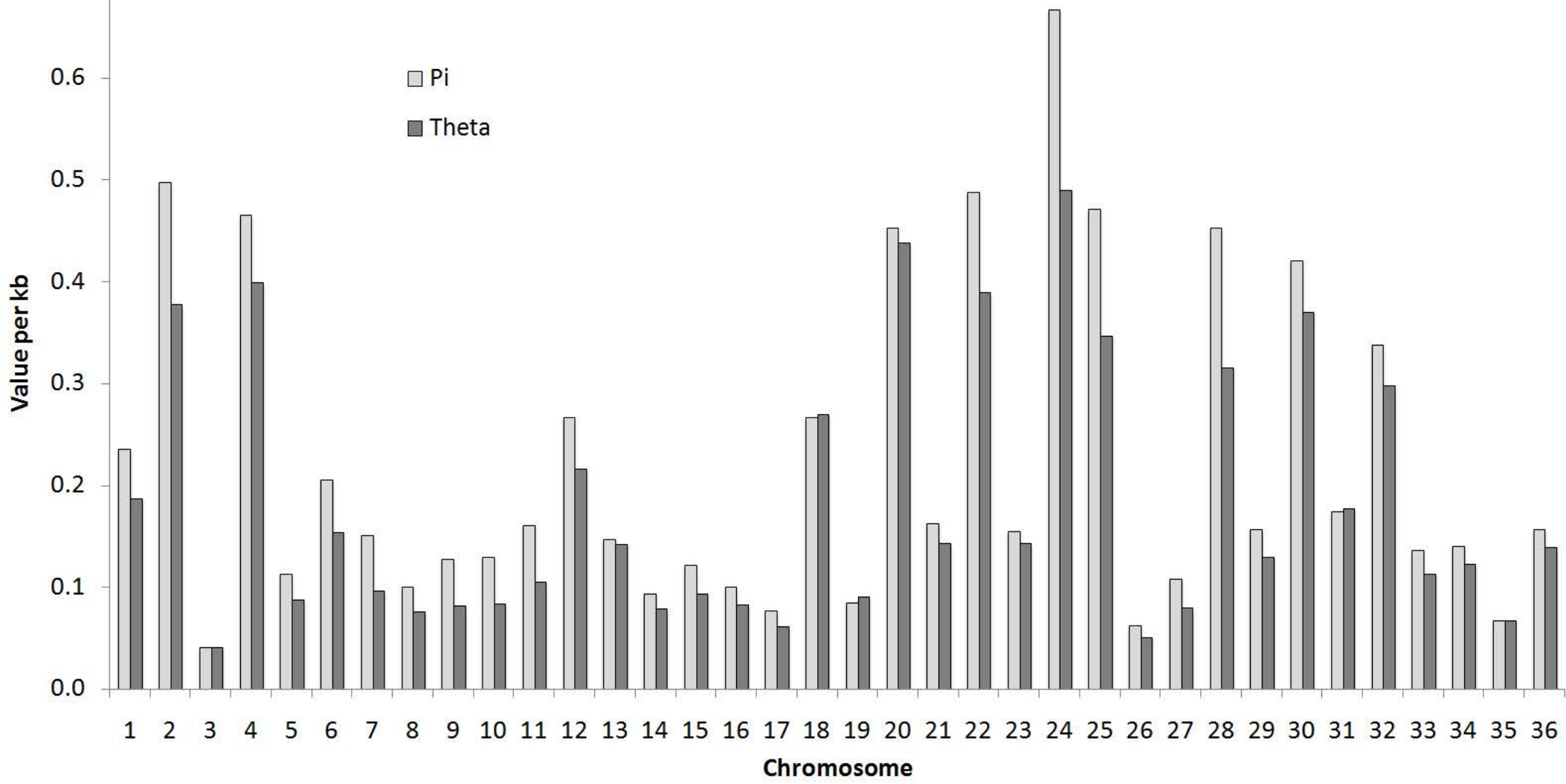

Supplement: Figure S9 — Extensive variability of diversity between chromosomes in the CUK strains. Diversity for each chromosome was calculated for the 12 Turkish strains using the mean nucleotide diversity (Pi, π) [55] and Watterson's theta (θ) [56] per kb for each chromosome. π most strongly reflected intermediate-level variants, and θ lower-frequency ones: under neutrality these should be approximately equal but recombination generally will increase the π to θ ratio. 4,268 genes contained fixed SNPs compared to the L. infantum genome and 1,820 had variation within the 12 strains – 1,042 genes had both inter- and intra-specific variation. (PDF) [file pgen.1004092.s009.pdf]

A

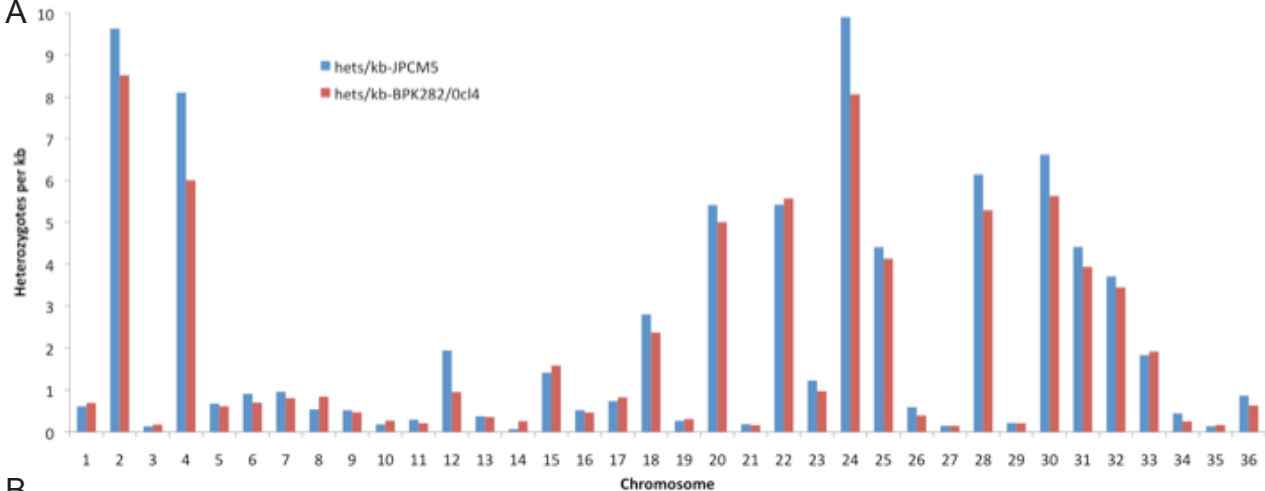

B

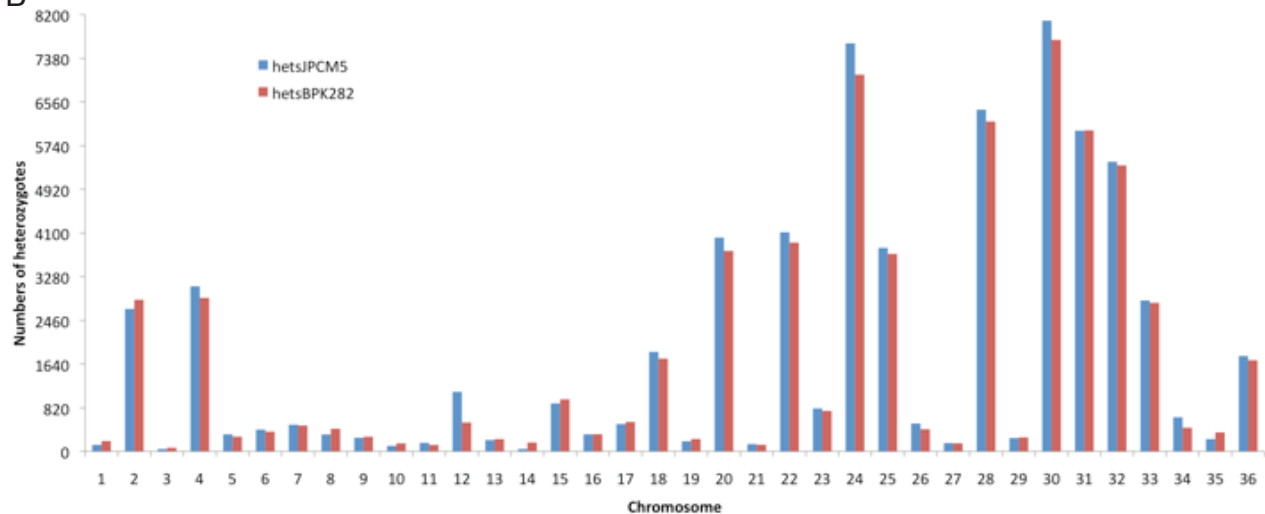

Supplement: Figure S10 — Heterozygosity across chromosomes across the 12 CUK strains for each chromosome using L. infantum JPCM5 and L. donovani BPK282/0cl4 reference genomes. Heterozygosity within the 12 Turkish strains for each chromosome using L. infantum JPCM5 (blue) and L. donovani BPK282/0cl4 (red) reference genomes was measured per kb (upper panel) and in total numbers (lower panel). The L. infantum JPCM5 (blue) reference genome provided a better resolution of heterozygous alleles than L. donovani BPK282/0cl4 (red) because of its closer genetic distance. No association between SNP heterozygosity and chromosome copy number was evident, suggesting that chromosome copy number variation was recent rather than ancestrally stable. (PDF) [file pgen.1004092.s010.pdf]

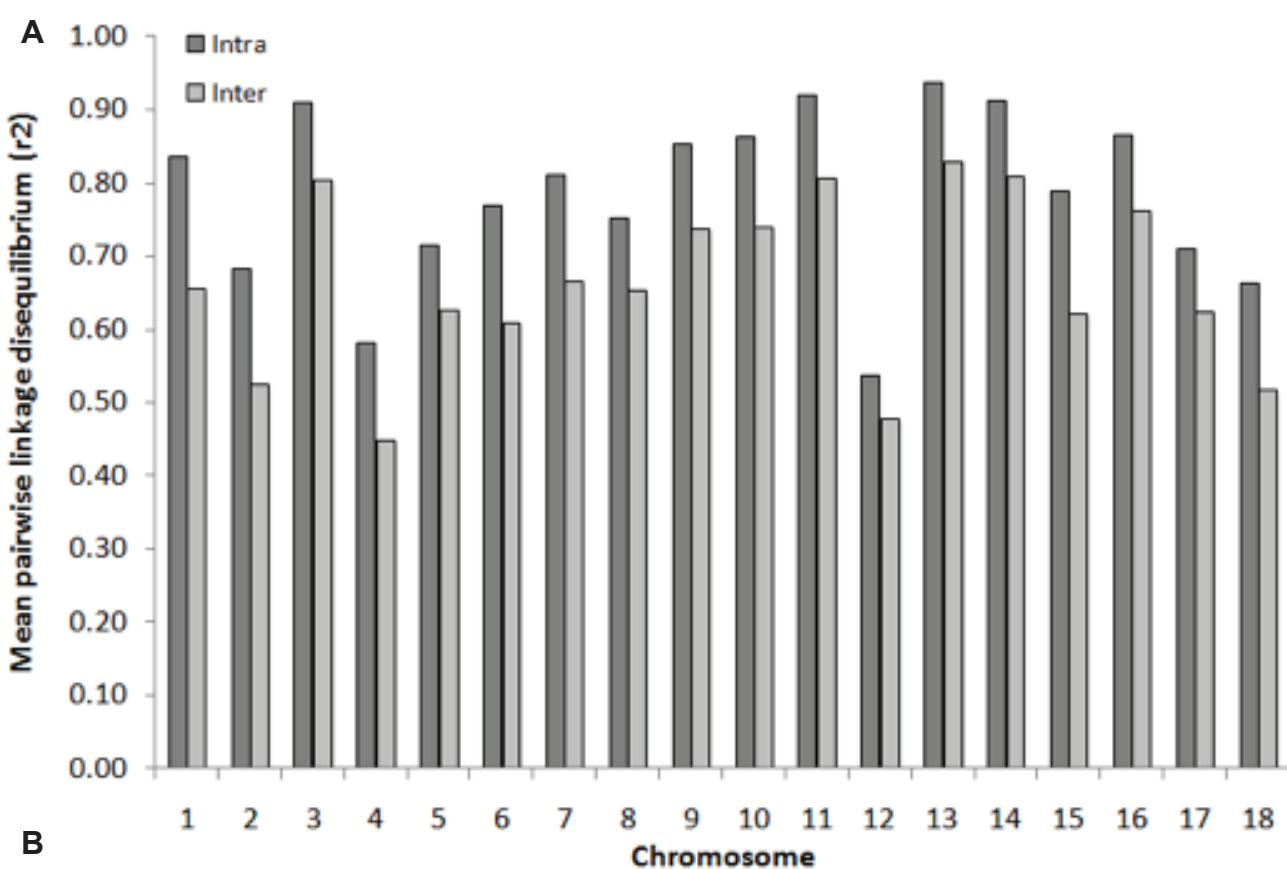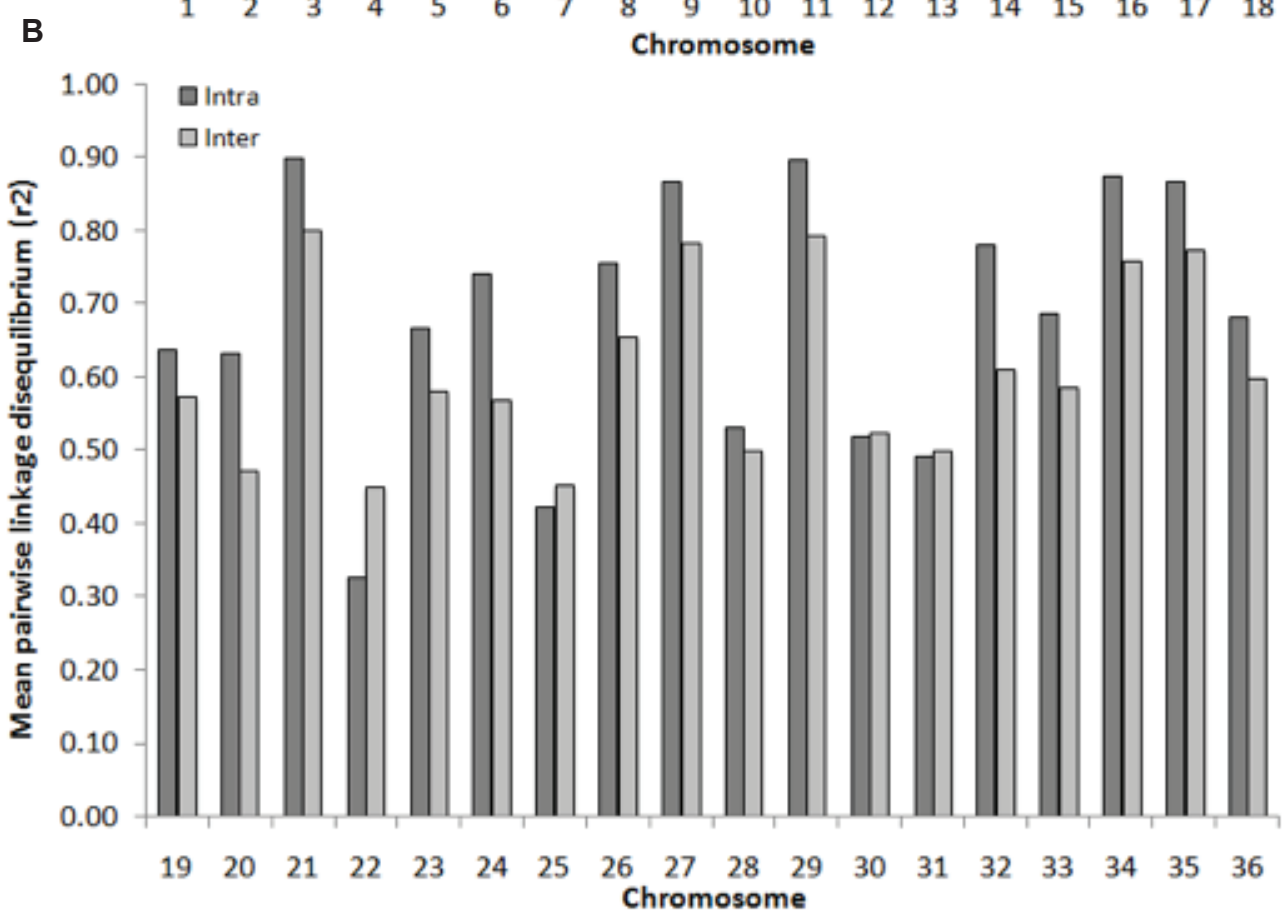

Supplement: Figure S11 — Mean intra- and inter-chromosomal pairwise linkage disequilibrium in the CUK strains. Intra- and inter-chromosomal linkage disequilibrium (LD) in the CUK strains was calculated for each SNP with all others on the same (intra) or a different (inter) chromosome for (A) variants segregating within the Turkish population only (“recent” polymorphism) and (B) including those fixed differences between the CUK isolates and the L. infantum JPCM5 genome (“hybrid” polymorphism). Values displayed are the averages across all SNP pairs for each comparison. Interchromosomal LD for both comparisons was higher on all 36 chromosomes except for 22, 25, 30 and 31, suggesting that recombination may be more frequent on the latter relative to other chromosomes. Mean LD for “recent” pairs on the same chromosomes (0.633±0.415 for 7,080,857 pairs) was higher than for pairs on separate chromosomes (0.584±0.441 for 143,126,165 pairs; t-test p<0.0001). Standard deviation values for “recent” LD within chromosomes were large but showed little variation, ranging from 0.42 to 0.52. Similarly, those between chromosomes were also uniform (0.44–0.50). This suggested inherited recombination events that occurred since the original hybridisation event were specific to certain chromosomes only: extensive recombination would result in intrachromosomal LD values much higher than interchromosomal ones. (PDF) [file pgen.1004092.s011.pdf]

Chromosome (10kb blocks)

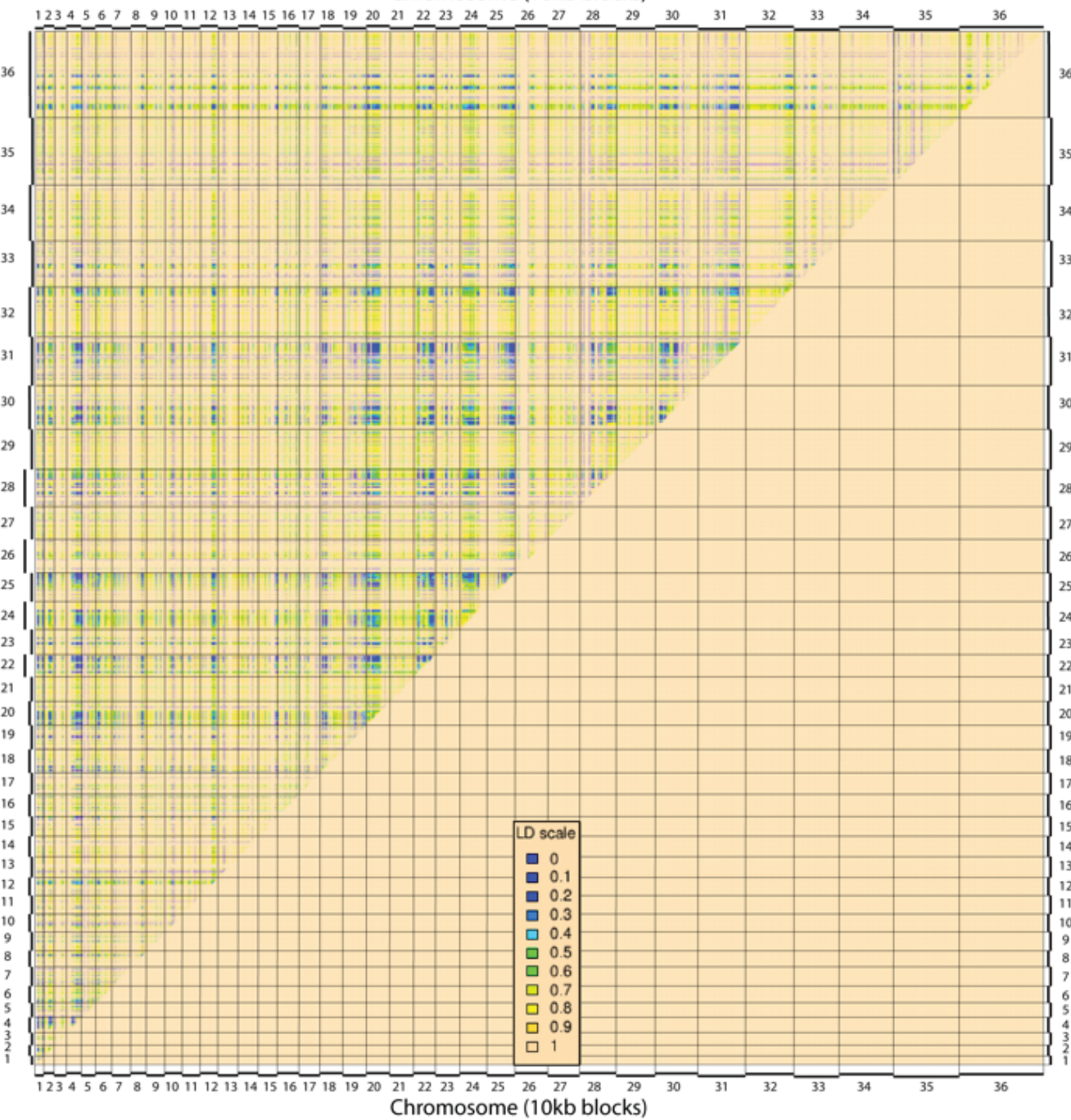

Supplement: Figure S12 — Genome-wide linkage disequilibrium in the CUK population. Intra-and inter-chromosomal linkage disequilibrium (LD) across the genome using SNPs variable within the 12 CUK strains. Values displayed are the average LD (r2) for all SNPs between pairs of 10 kb blocks scaled from low (blue) to high (beige) LD. Intra-chromosomal LD is shown in boxes along the diagonal; inter-chromosomal values are shown above this. The LD patterns provide evidence of extensive LD between chromosomes. Blocks with low LD represent putative recombination breakpoints that occurred since the hybridisation event. (PDF) [file pgen.1004092.s012.pdf]

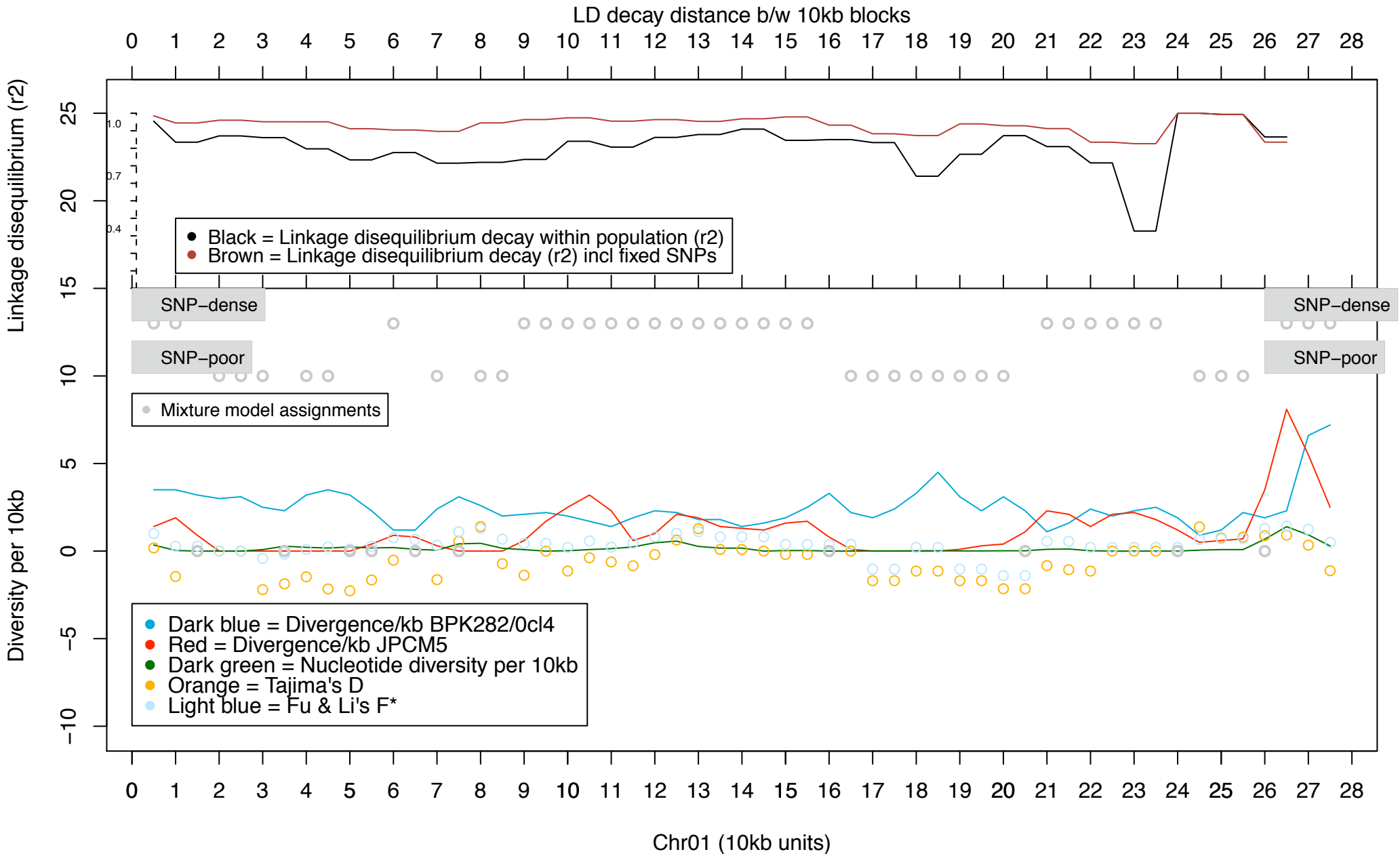

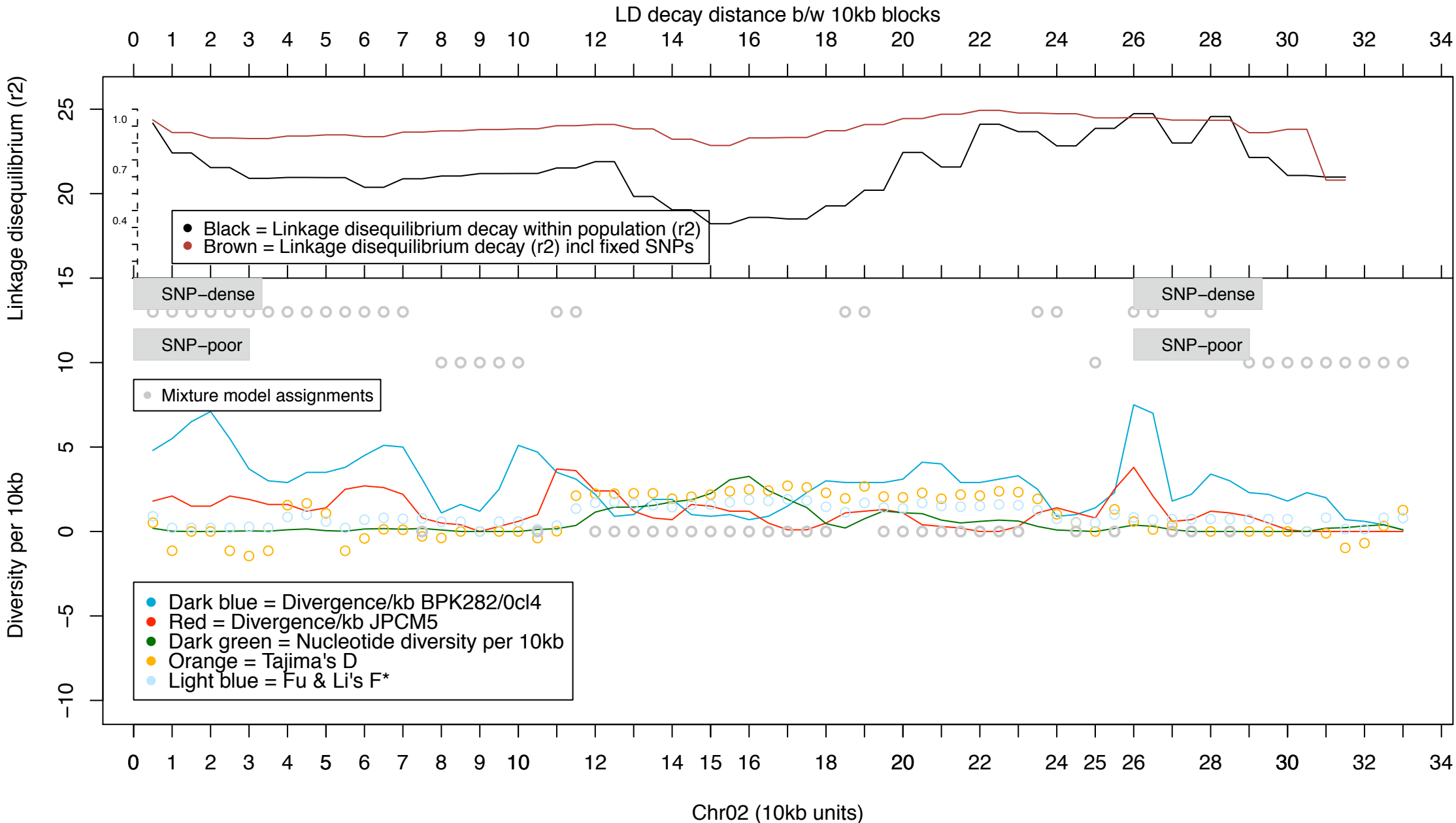

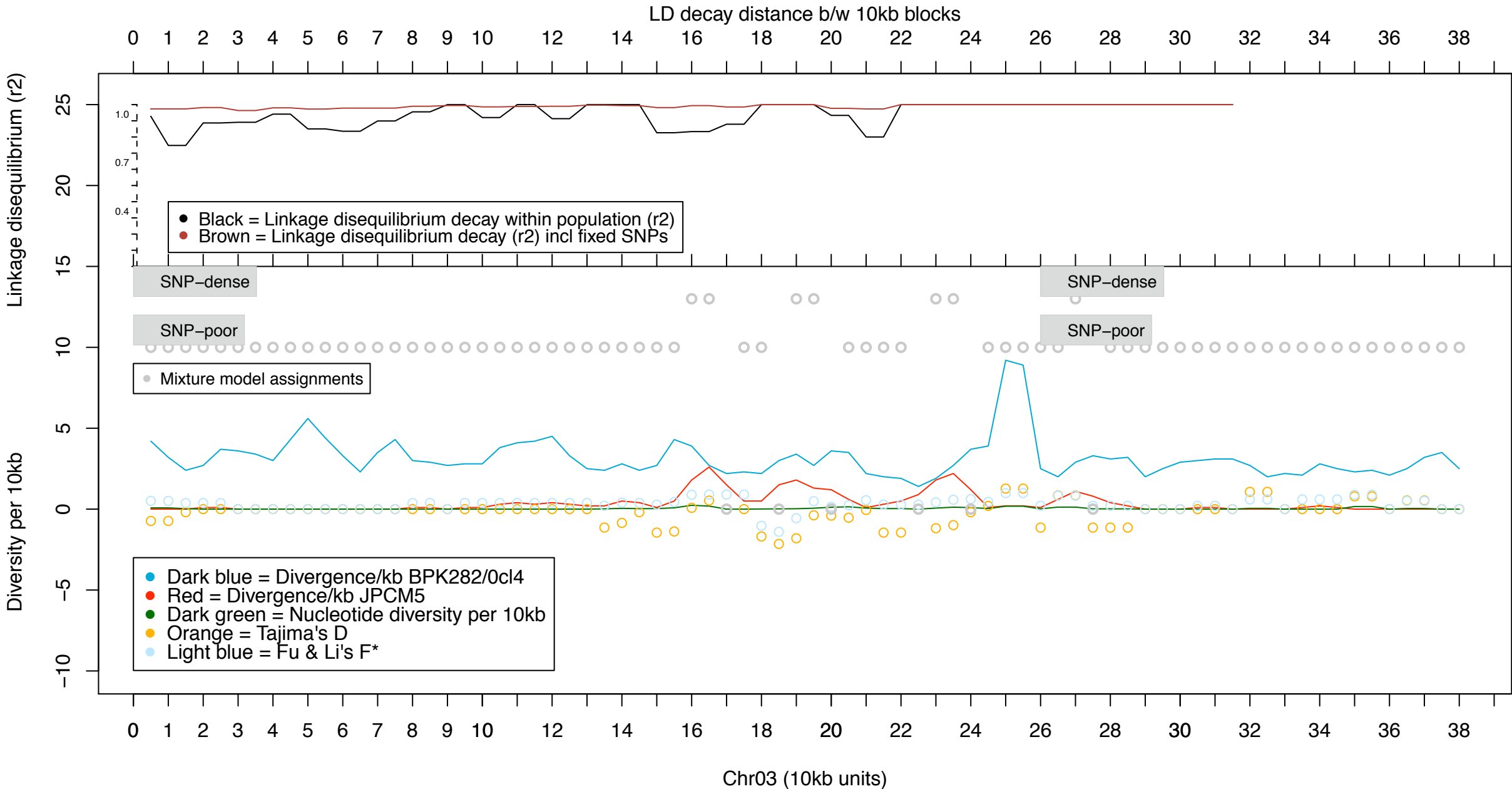

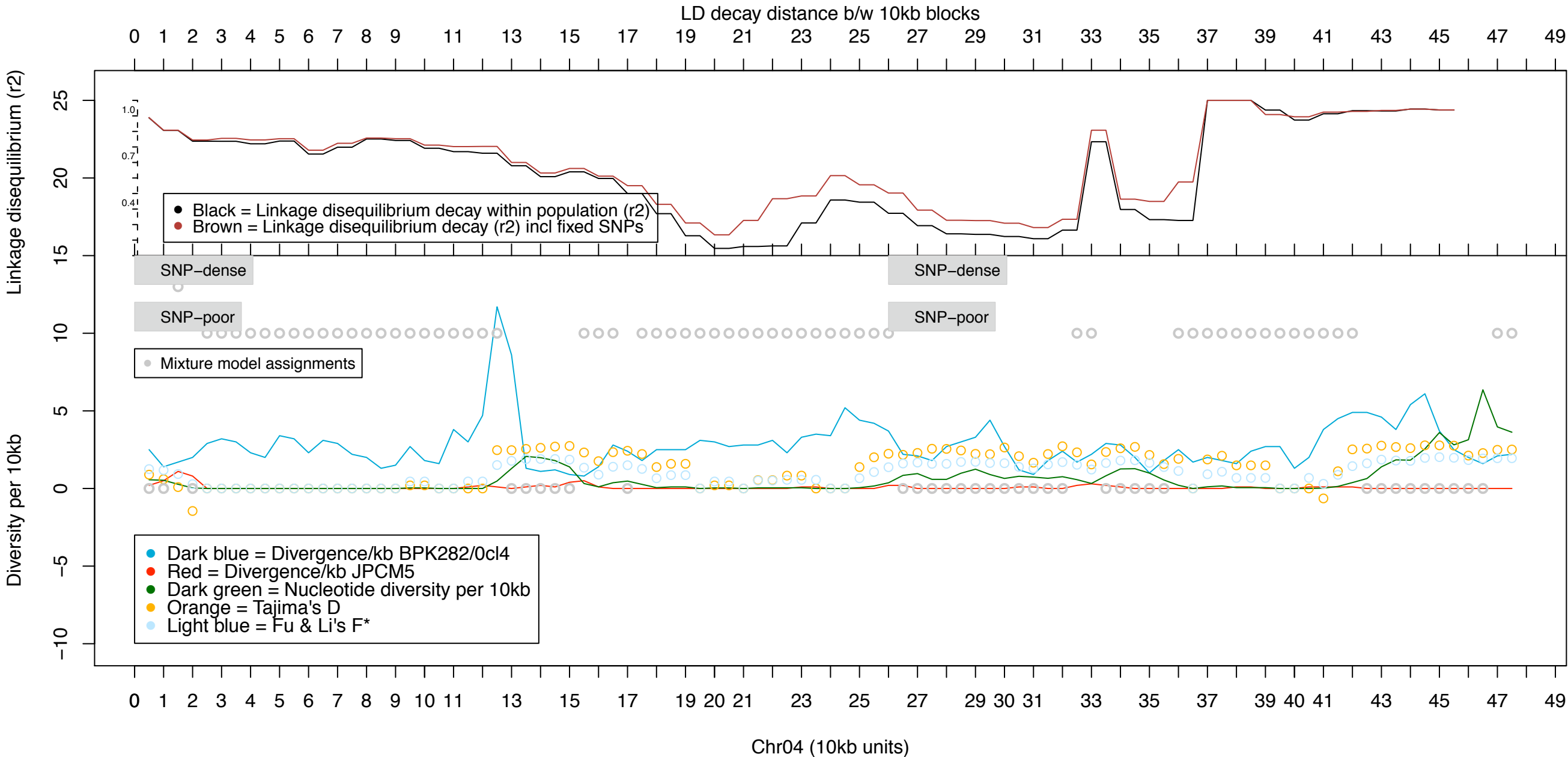

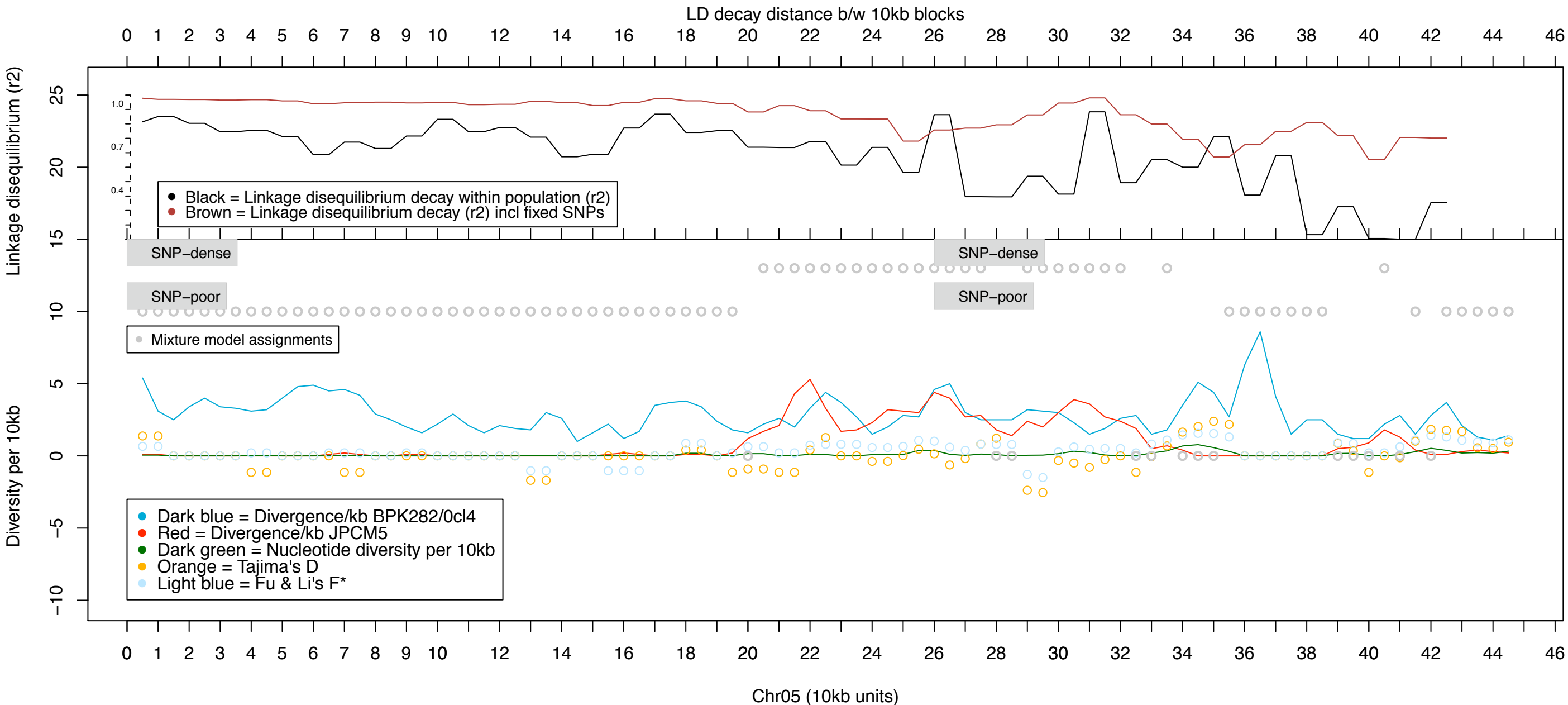

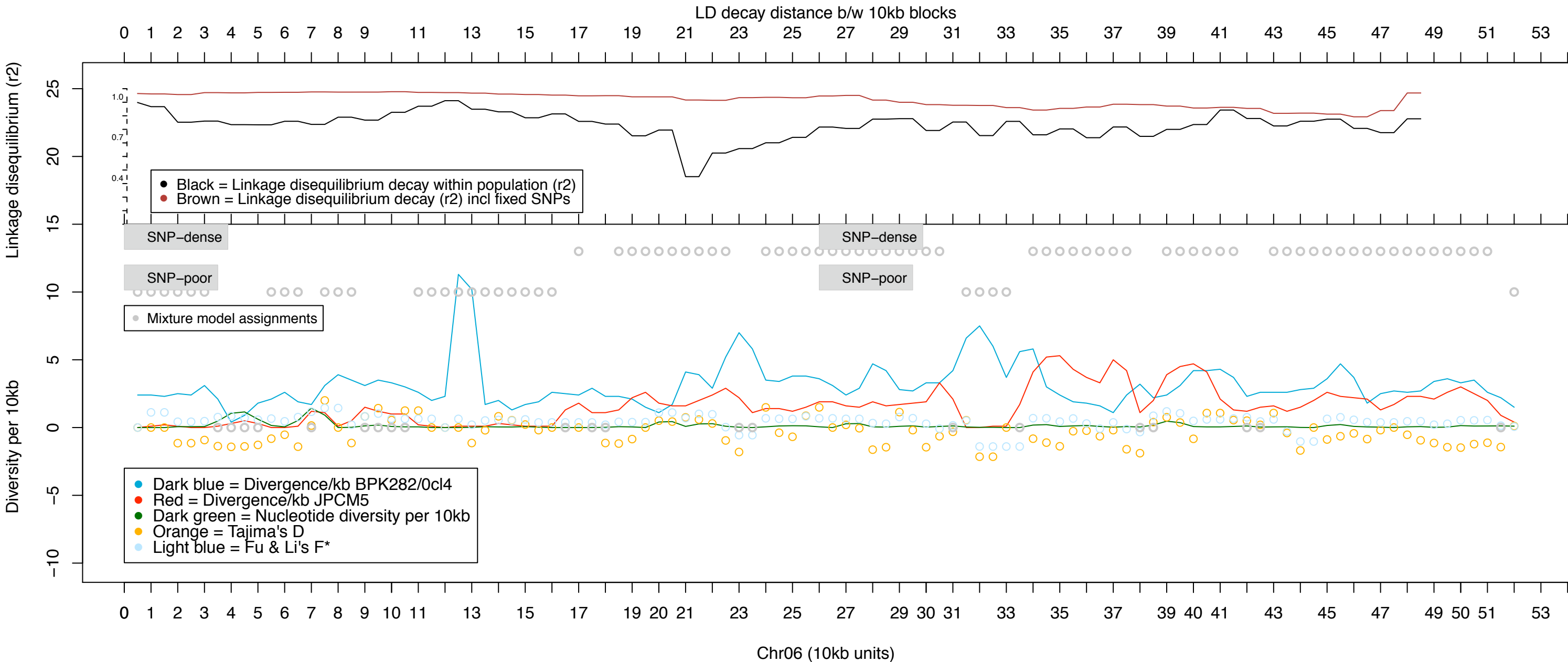

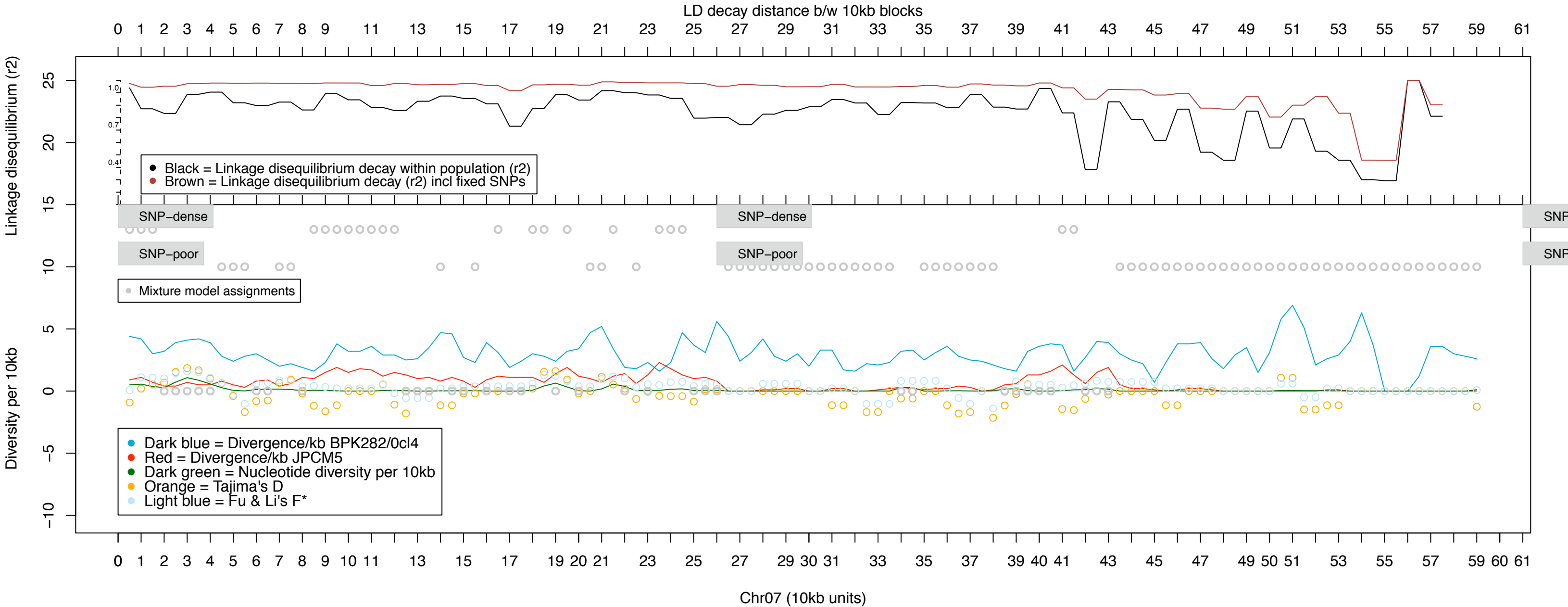

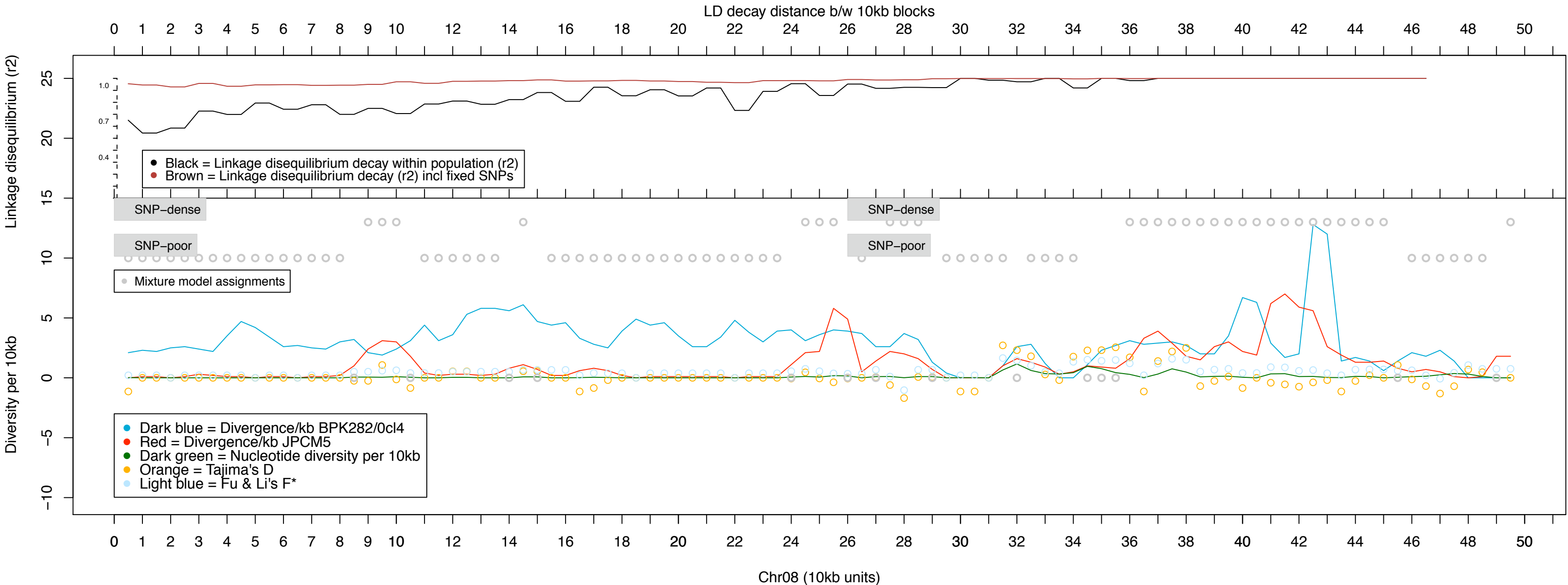

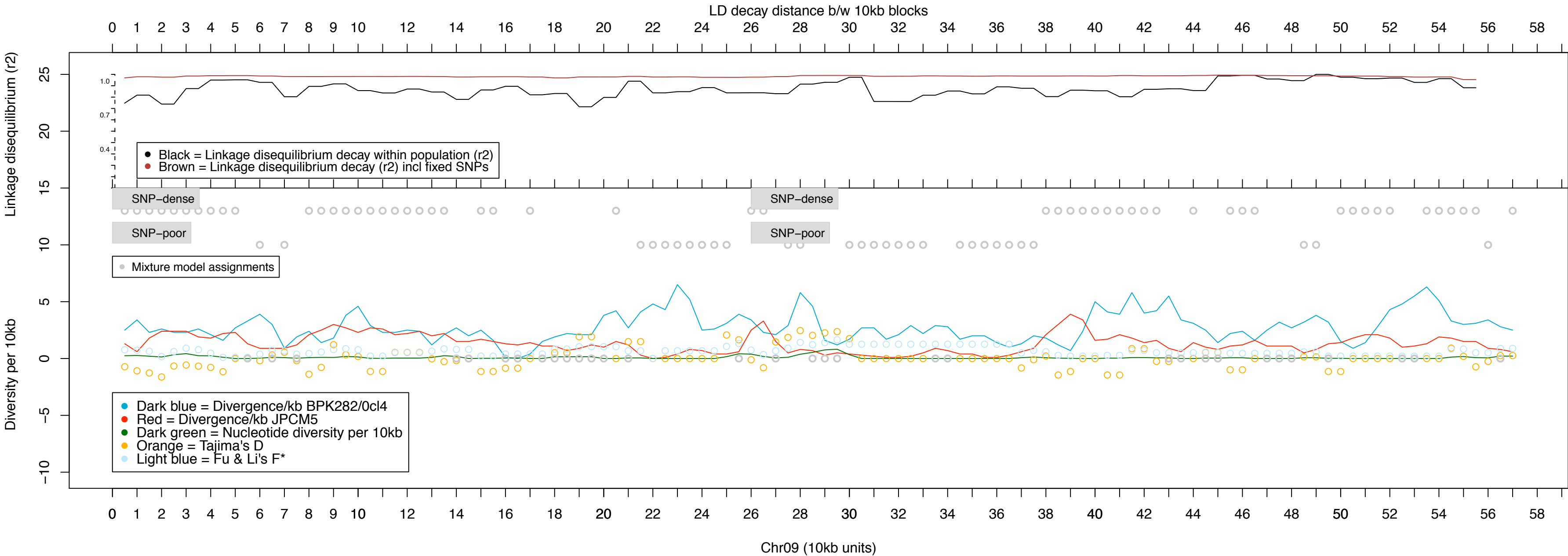

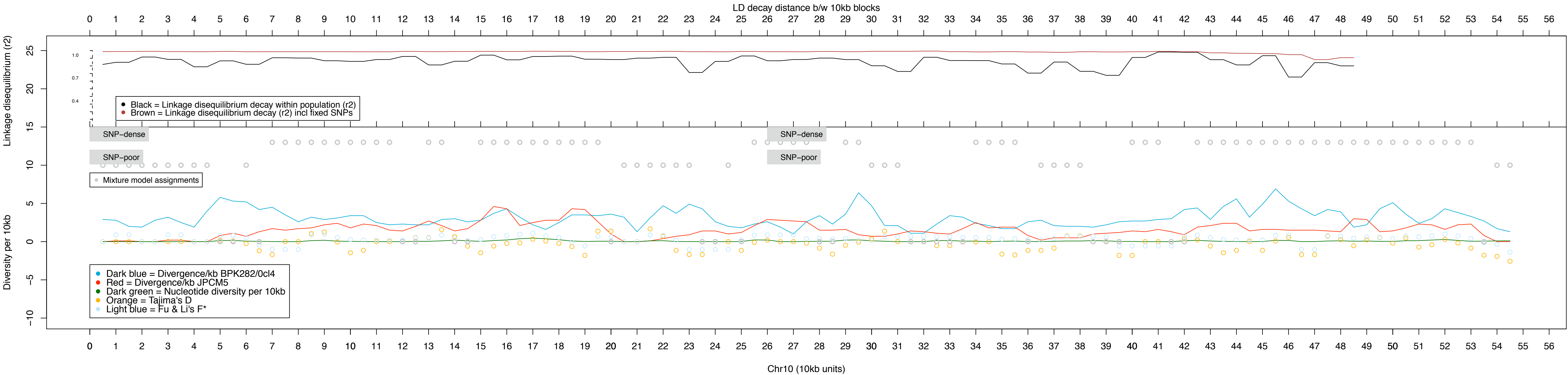

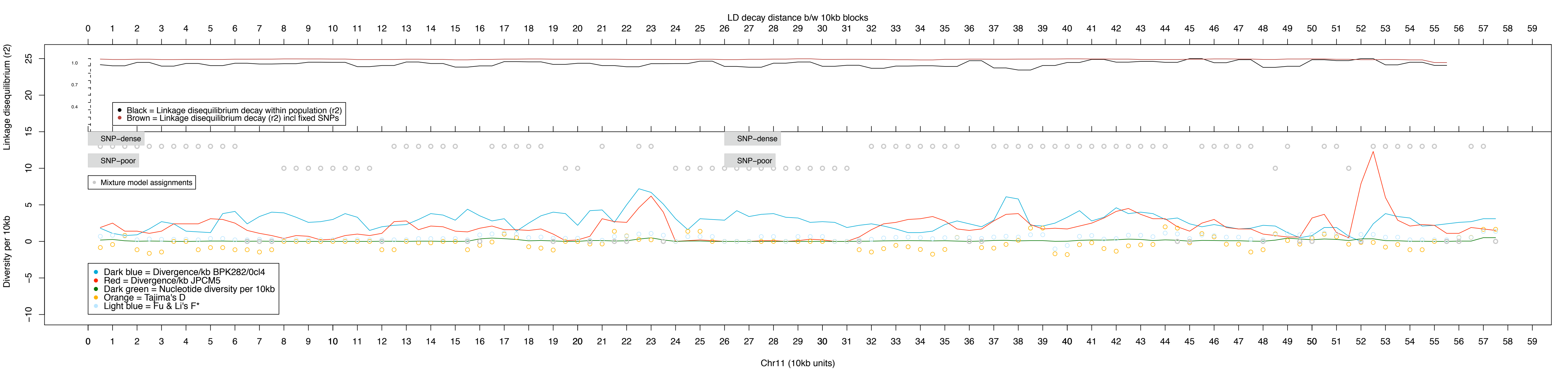

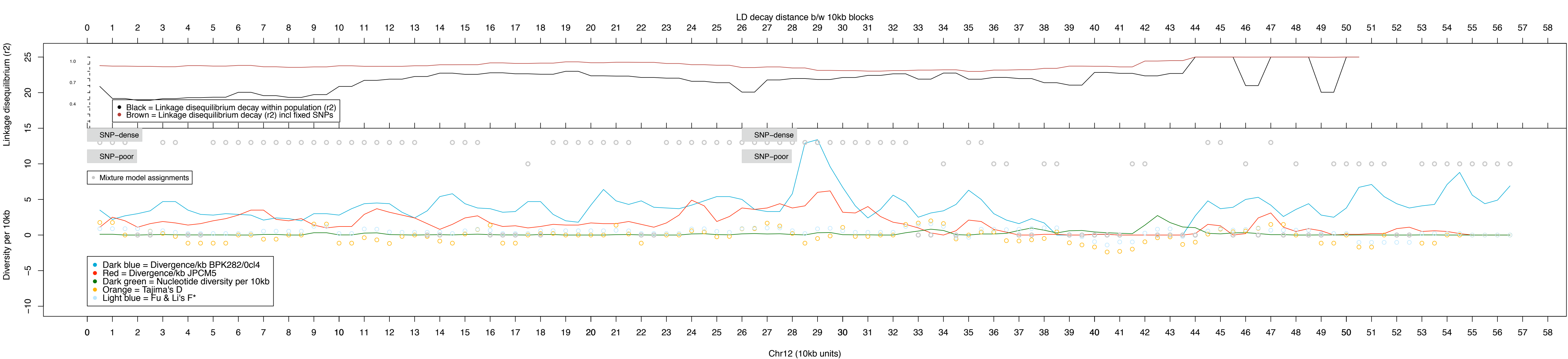

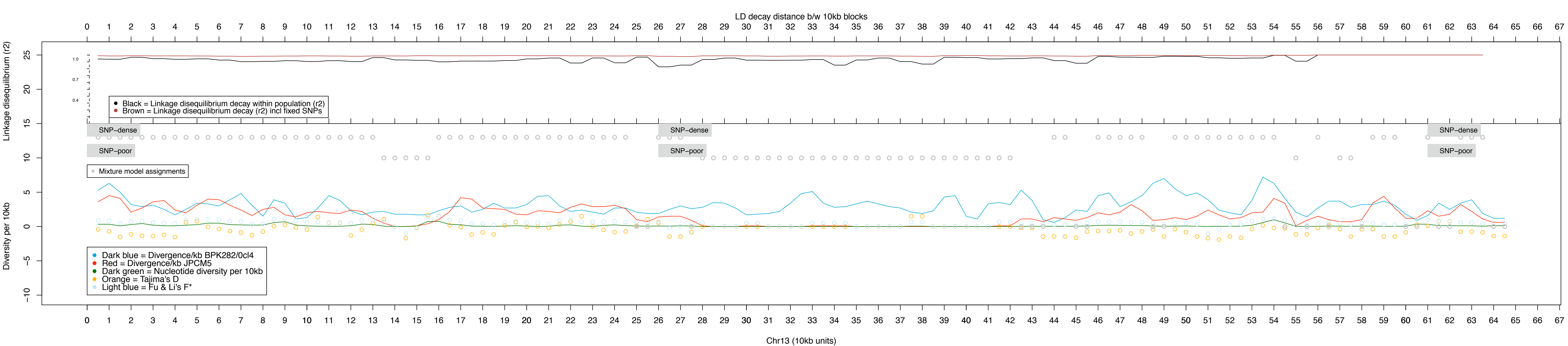

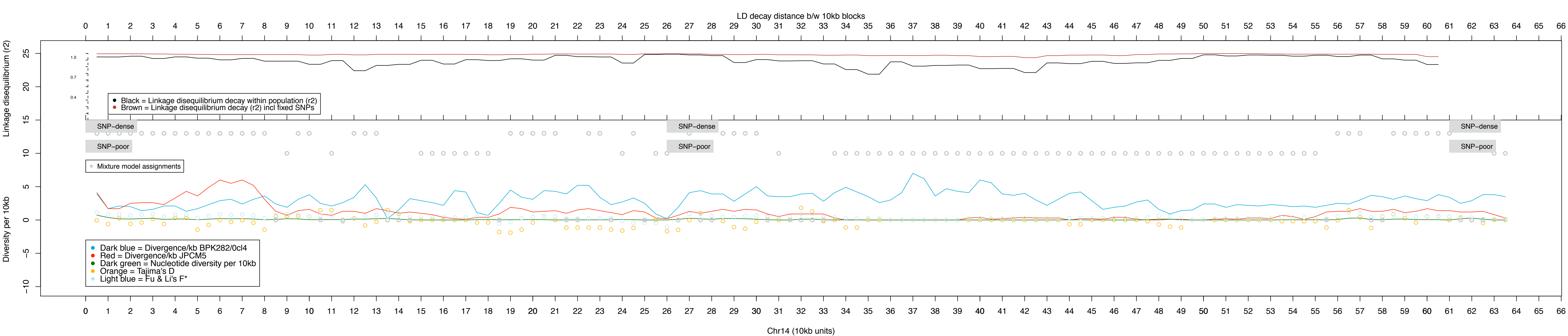

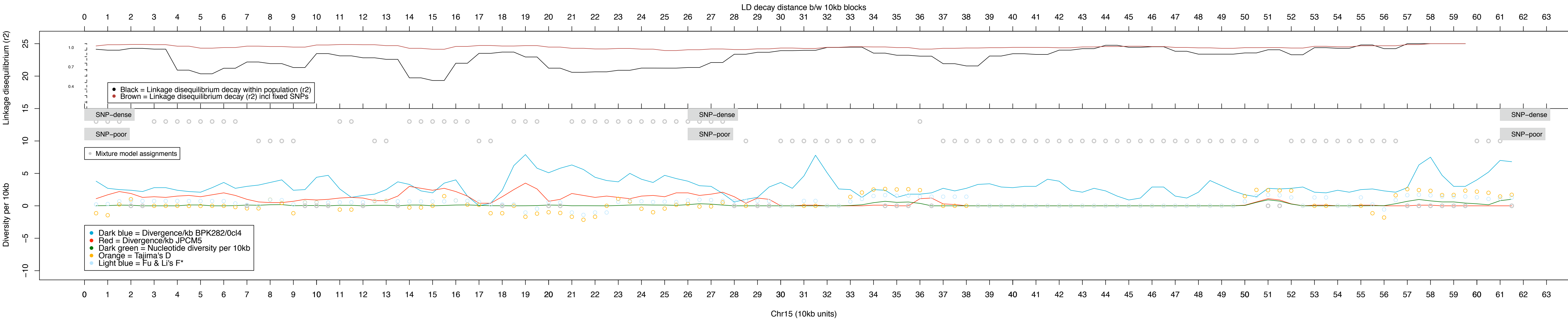

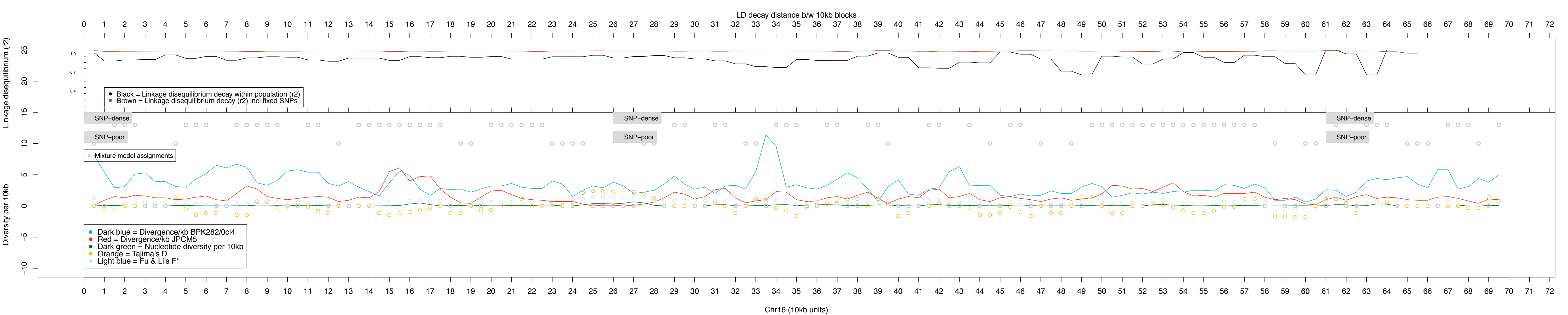

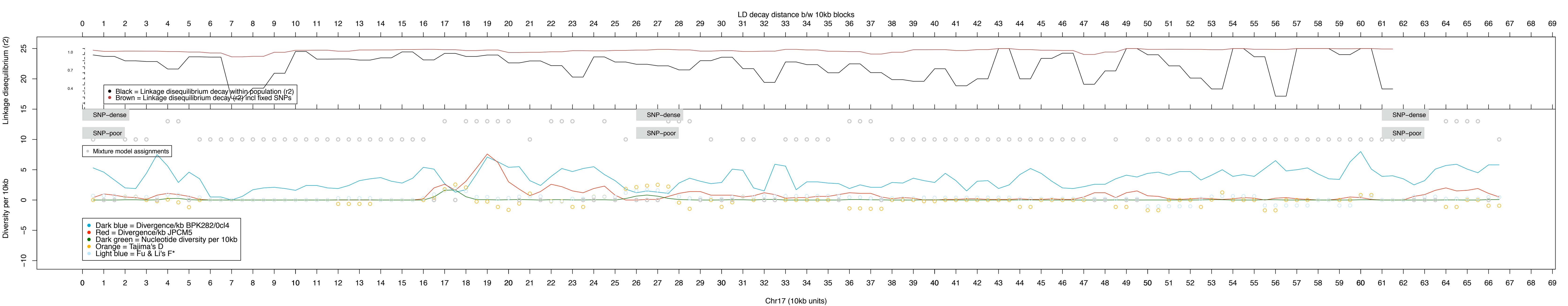

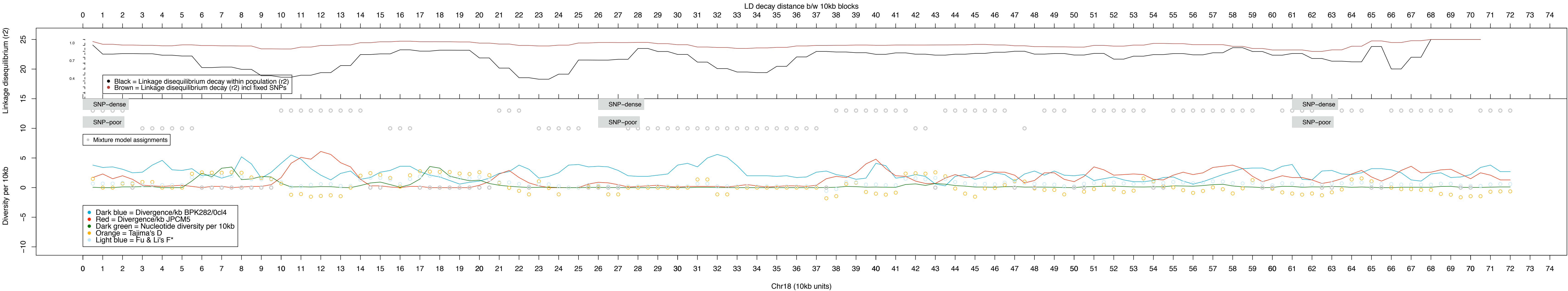

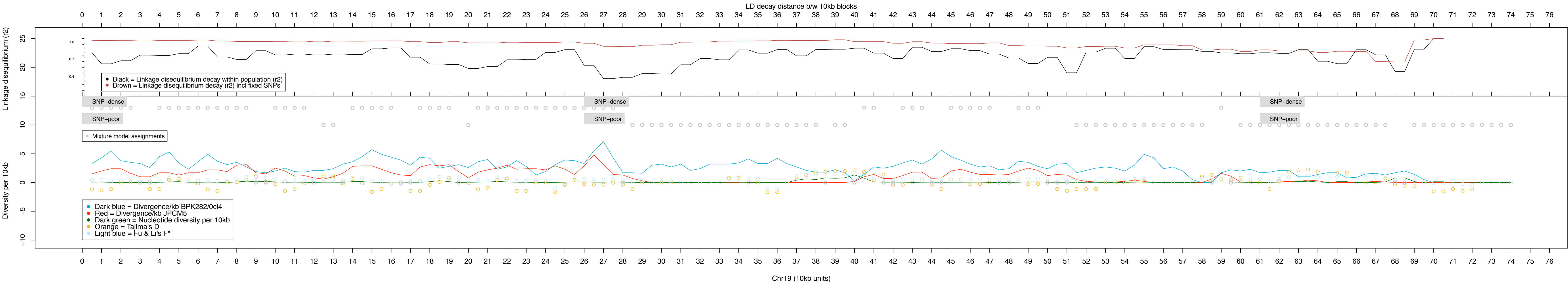

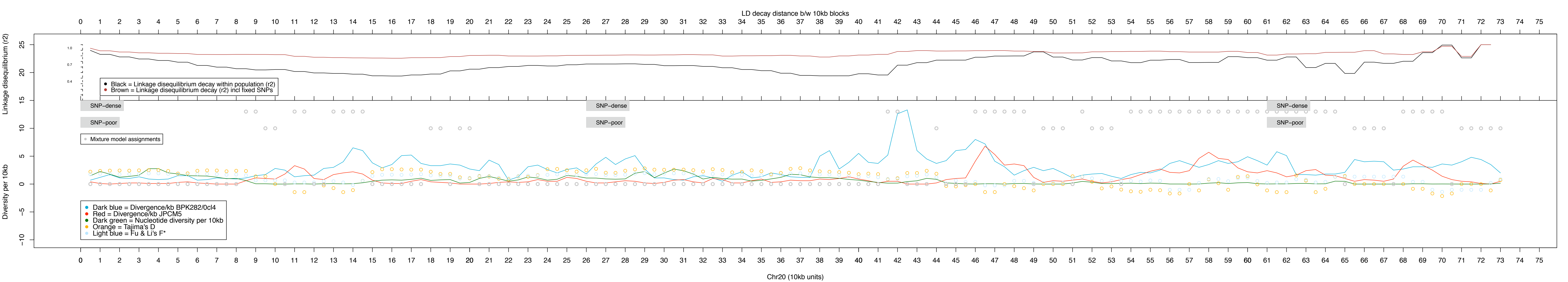

Linkage disequilibrium (r<sup>2</sup>)

LD decay distance b/w 10kb blocks

Diversity per 10kb

Chr21 (10kb units)

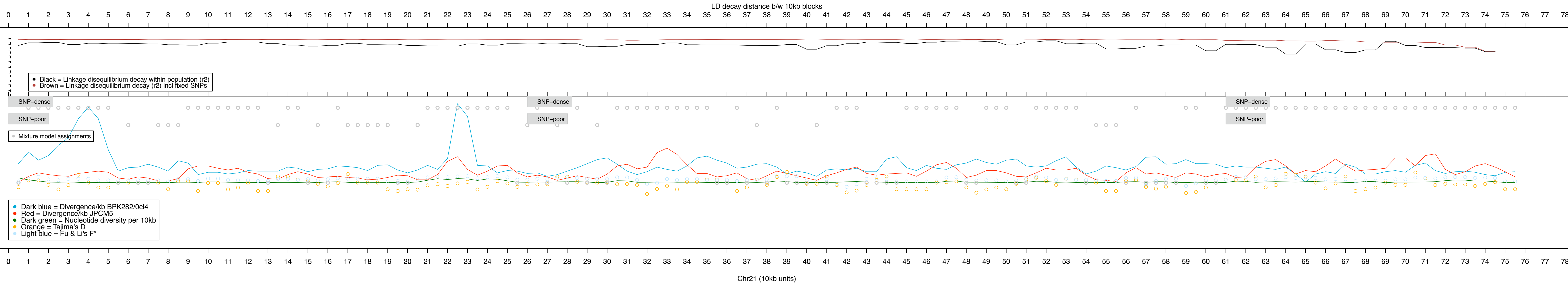

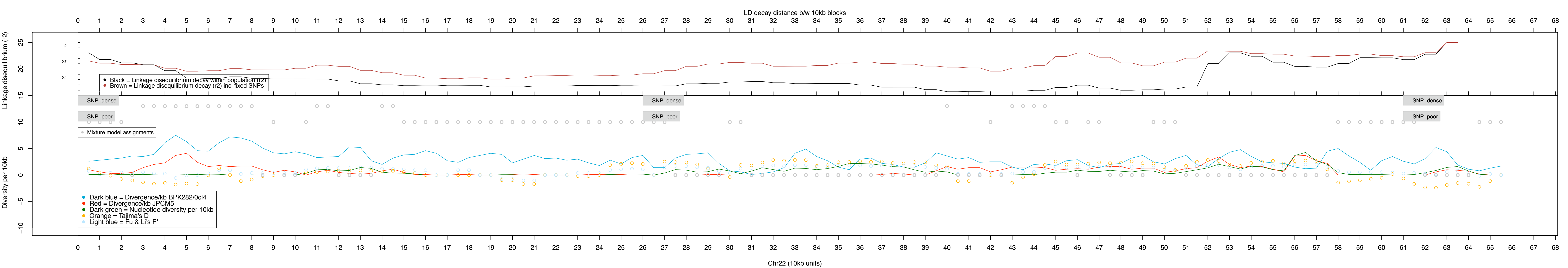

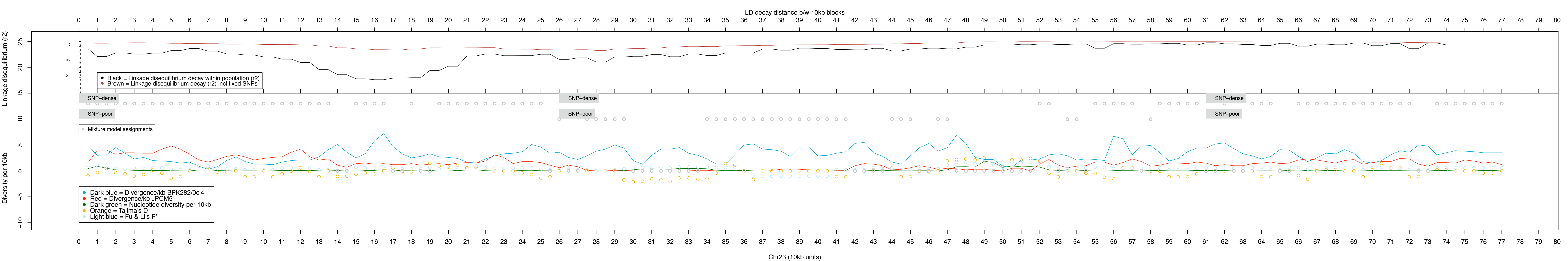

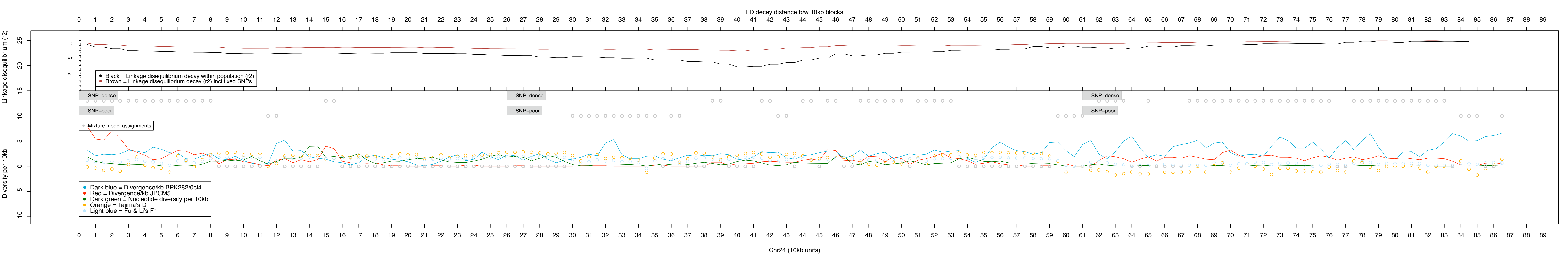

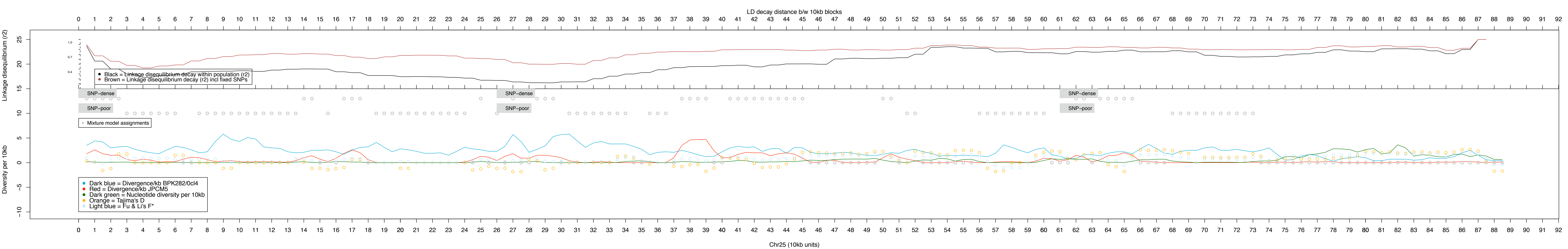

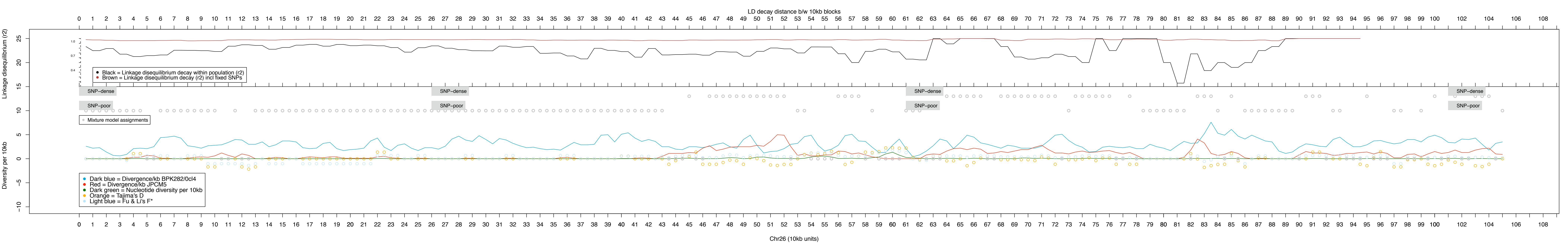

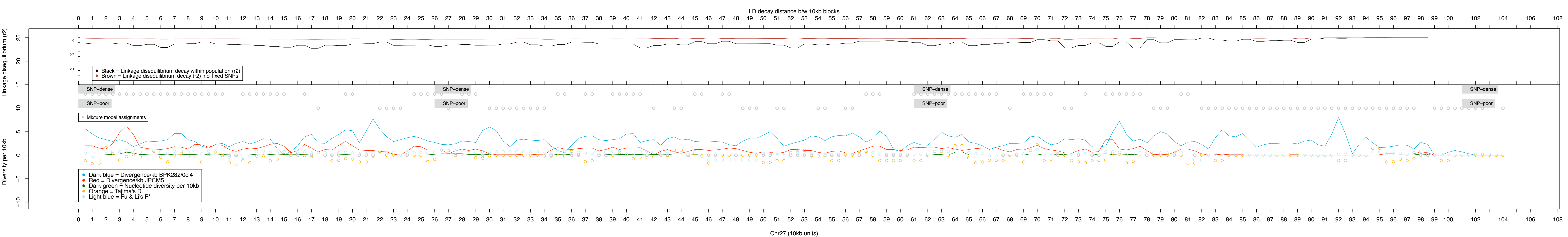

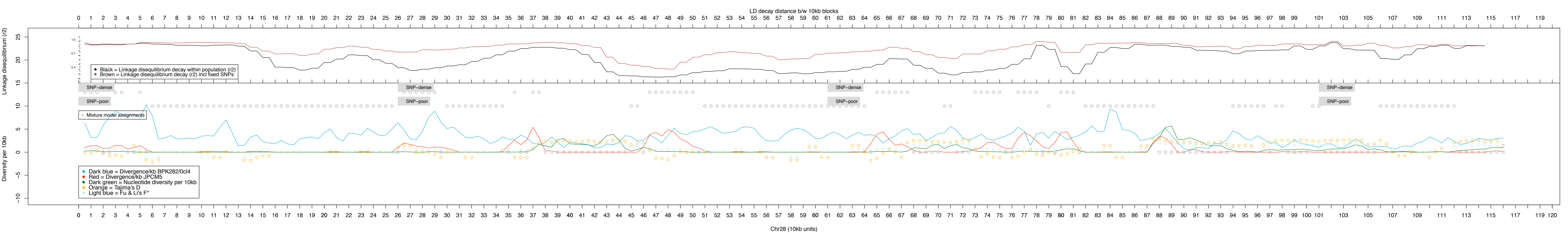

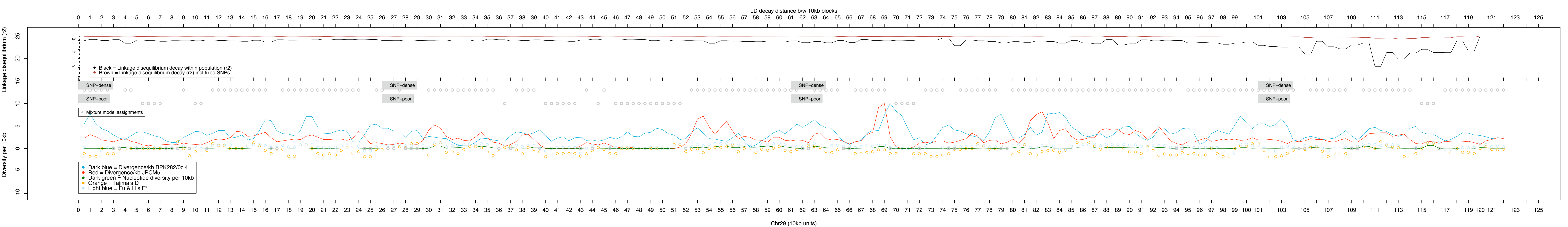

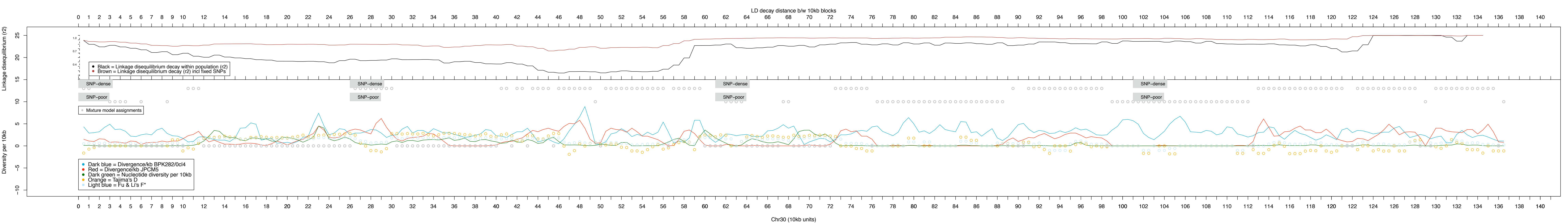

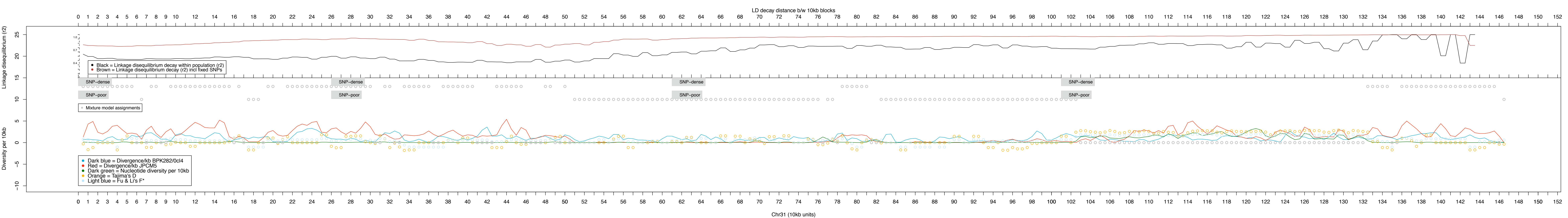

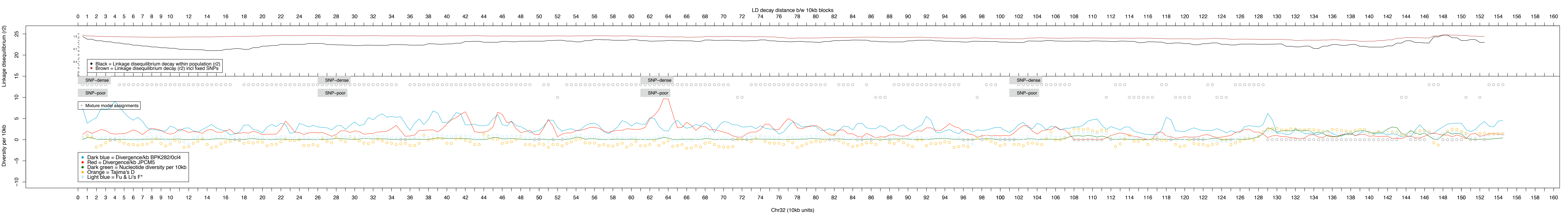

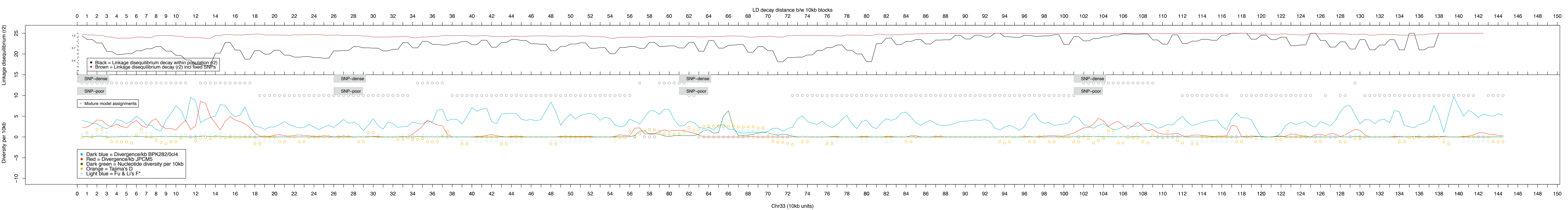

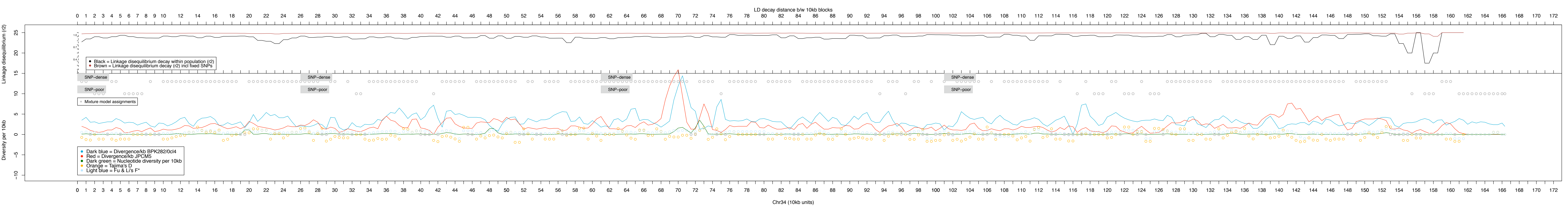

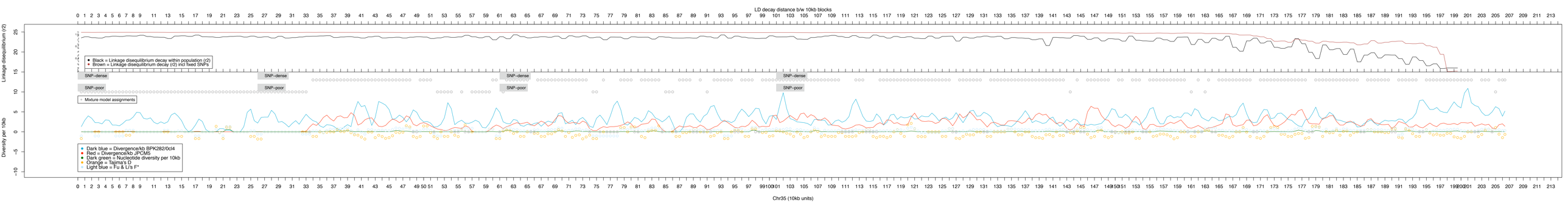

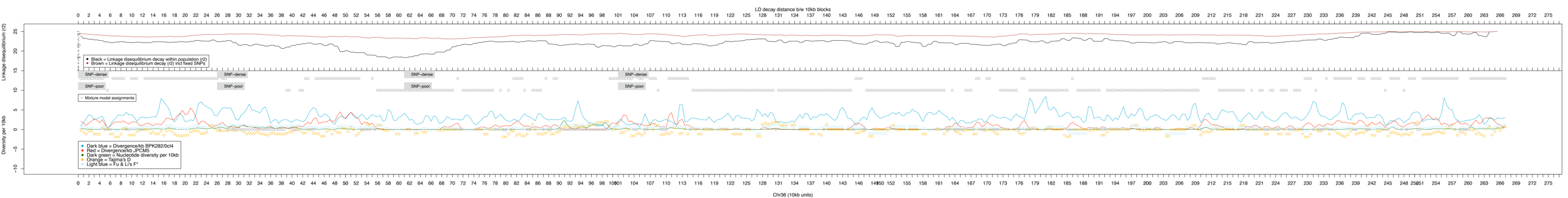

Supplement: Figure S14 — Decay of linkage equilibrium between SNP pairs with distance for each chromosome. Linkage disequilibrium (LD) decay with distance for the entire chromosome. The LD (r2 between SNP pairs, y-axis) is on the inset (ranging from 0 to 1). The distance (x-axis) is shown in units of 10 kb. LD decay varied considerably between chromosomes. Bottom: Diversity at each chromosome region shown in 10 kb blocks for the fixed SNPs per kb compared to L. infantum JPCM5 (red), those relative to L. donovani BPK282/0cl4 (blue), the nucleotide diversity (π) per 10 kb for variation within the CUK population (green), and the summary statistic Tajima's D. Note the difference scale for π compared to the fixed SNPs. Tajima's D compares the level of intermediate- and low-frequency variants; here positive D values tended to occur at possible recombination blocks. (PDF) [file pgen.1004092.s014.pdf]

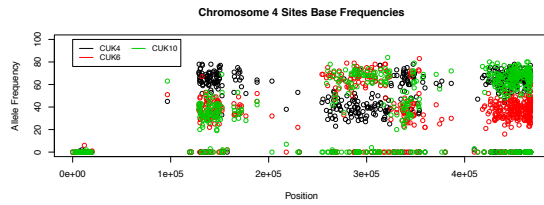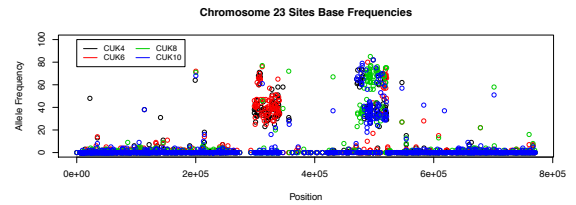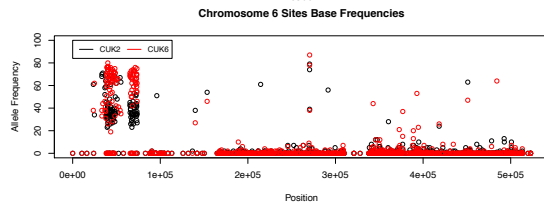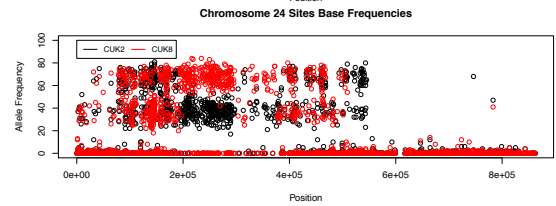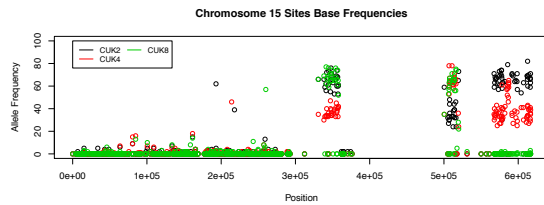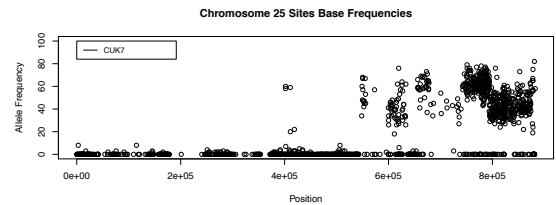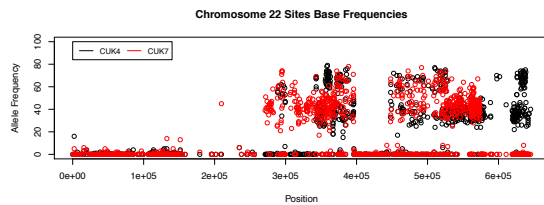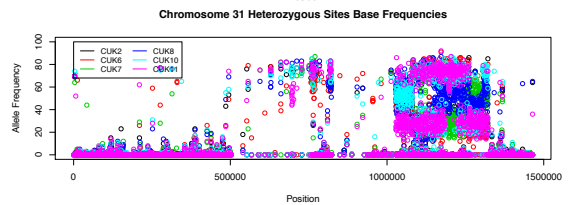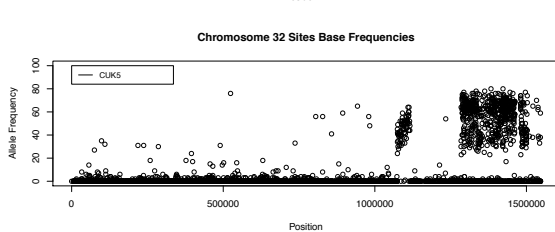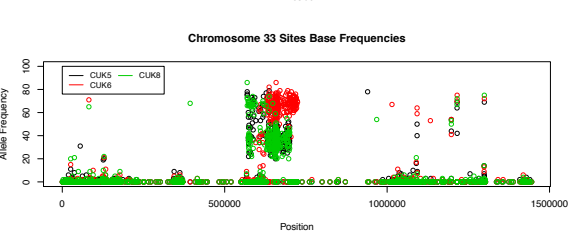

Supplement: Figure S15 — Variation in allele frequencies for heterozygous sites on multisomic CUK chromosomes. Analyses are shown for Chromosomes 4,6,15,22–25,31–33. Shifts in allele frequencies from 0.33 to 0.66 on trisomic chromosomes or 0.25 to 0.75 or 0.5 on tetrasomic chromosomes underscore recombination sites. (PDF) [file pgen.1004092.s015.pdf]

A

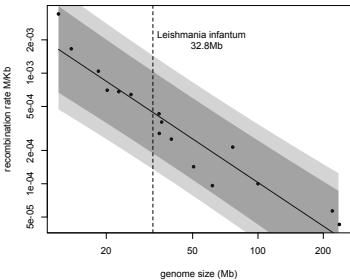

B

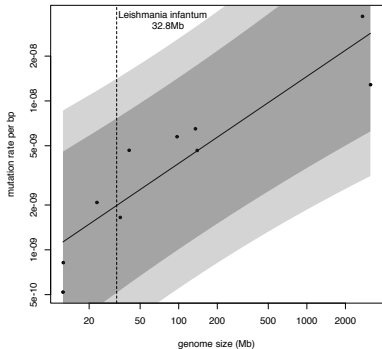

Supplement: Figure S16 — Regression model estimates of mutation rate and recombination rate in Leishmania infantum. (A) Recombination rate and (B) mutation rate both show a consistent relationship with genome size across eukaryotic species. (A) Points show data for eukaryotic species from [55]; (B) Points show data from [56]. In both panels, the solid line represents the predicted relationship based on a log-log regression model, with dark and light shading represent 95% and 99% prediction intervals, respectively. Dashed line indicates the intercept for Leshmania infantum, with an estimated genome size of 32.8 Mb. (PDF) [file pgen.1004092.s016.pdf]

(A)

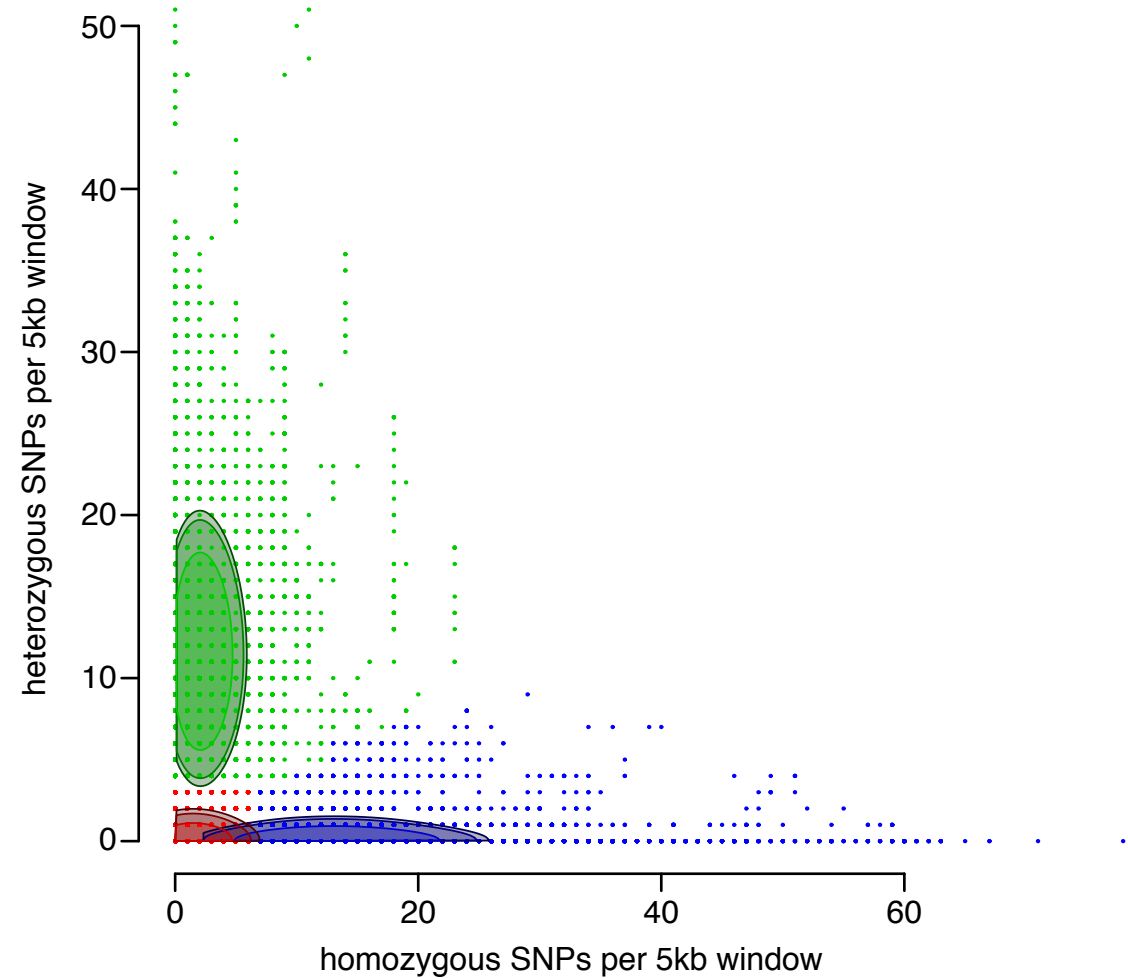

(B) (i)

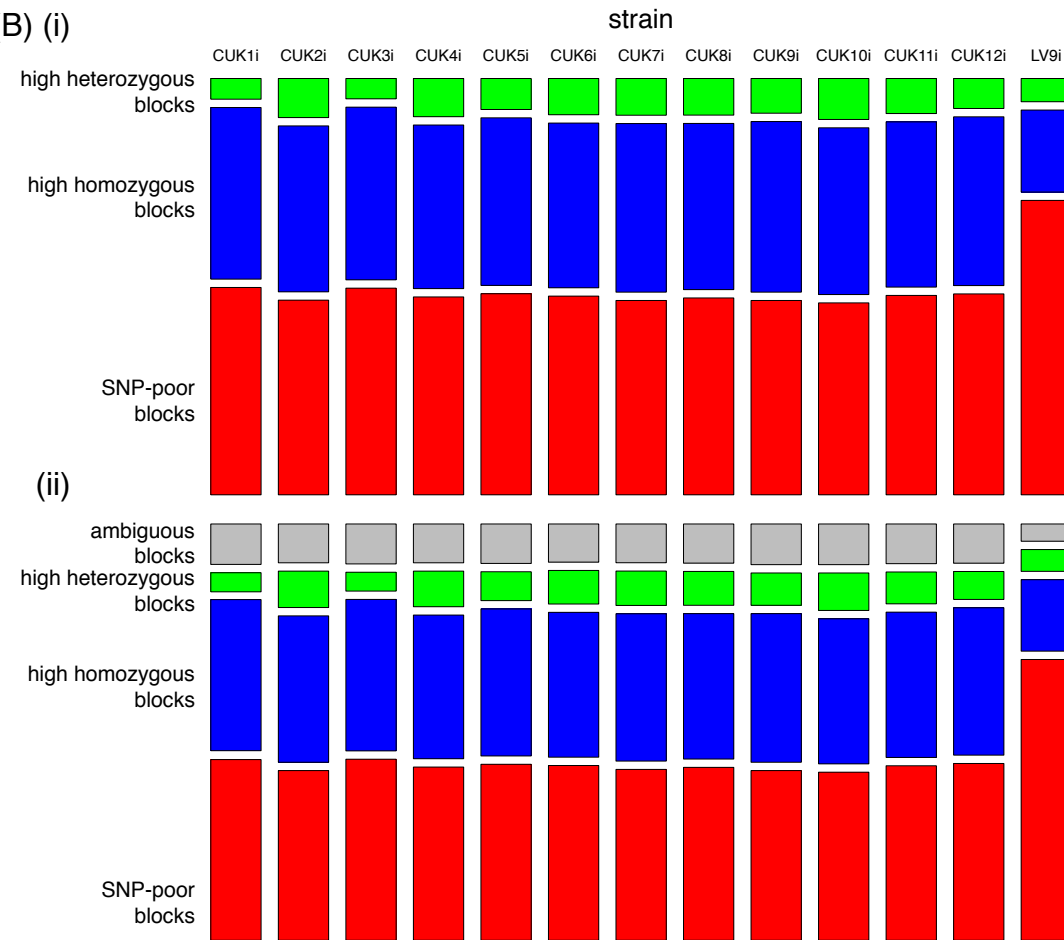

(C)

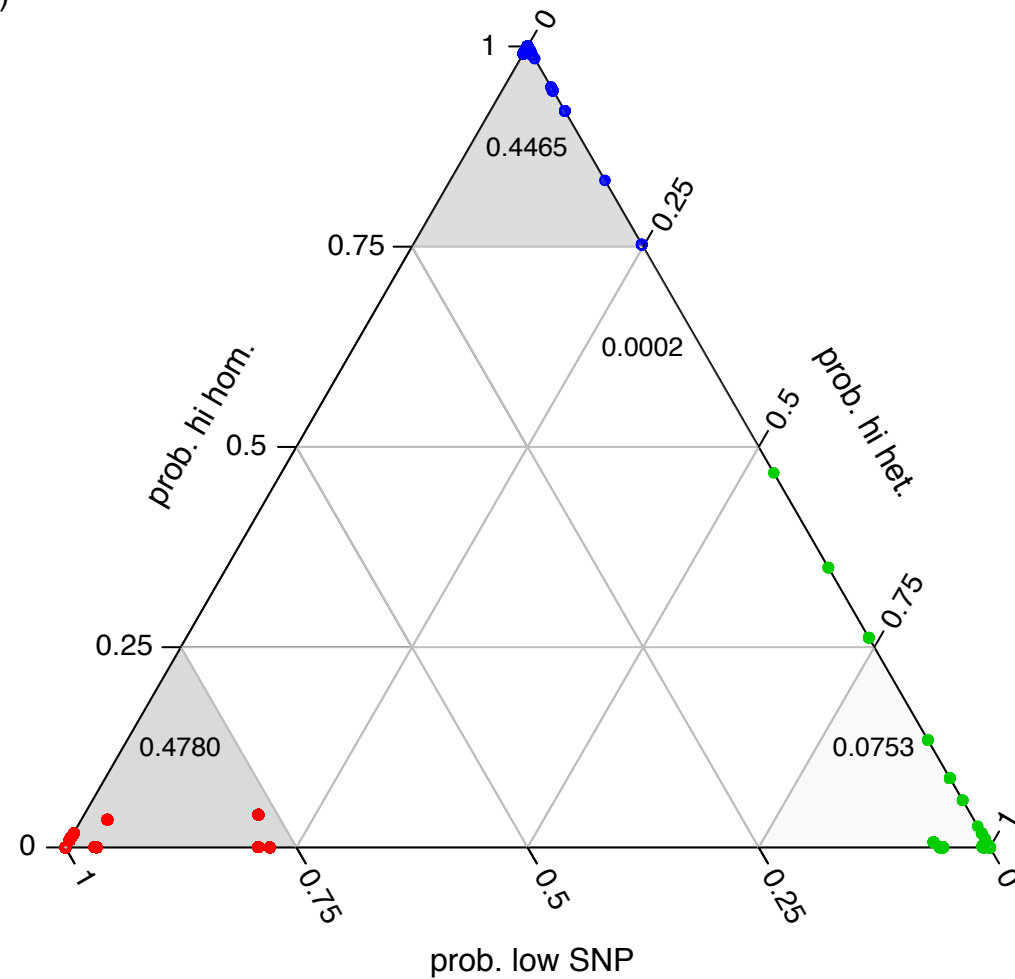

Supplement: Figure S17 — Detailed results of the mixture model analysis. (A) Counts of heterozygous and homozygous SNPs in non-overlapping 5 kb windows along the genomes of 12 CUK strains (points), assigned to three classes (point colours) by maximum posterior probability of cluster membership under the 3-component 2-dimensional Poisson mixture model. Ellipses represent density of each of the three model components, with different shading representing the 95%, 90% and 75% quantiles of the probability distributions for each. (B) Proportion of each genome assigned to each of the three categories (i) by maximum posterior probability and (ii) where windows with cluster assignment probability <0.9 are treated as ambiguous. (C) Posterior probability of cluster membership for each 5 kb window (points), represented as a ternary plot. Point colours represent maximum posterior probability cluster assignment; triangle shading density is proportional to number of windows assigned with equivalent probabilities to the three clusters. (PDF) [file pgen.1004092.s017.pdf]
