# Supplementary material for: Genomic Confirmation of Hybridisation and Recent Inbreeding in a Vector-Isolated Leishmania Population
Source: PLoS Genet. 2014 Jan 16;10(1):e1004092. doi: 10.1371/journal.pgen.1004092 (PMC3894156; doi:10.1371/journal.pgen.1004092)

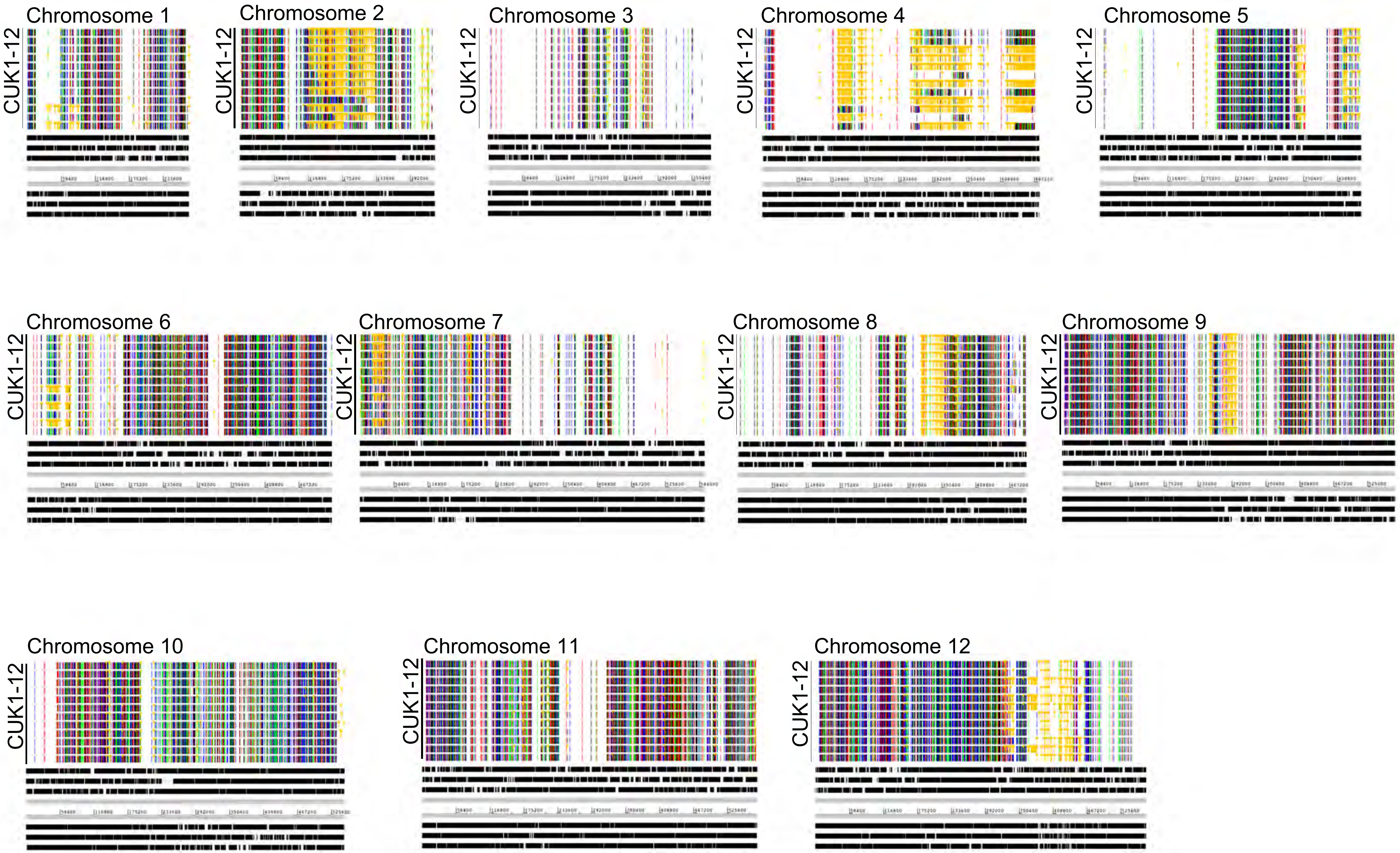
Chromosome 1
CUK1-12
Chromosome 2
CUK1-12
Chromosome 3
CUK1-12
Chromosome 4
CUK1-12
Chromosome 5
CUK1-12
Chromosome 6
CUK1-12
Chromosome 7
CUK1-12
Chromosome 8
CUK1-12
Chromosome 9
CUK1-12
Chromosome 10
CUK1-12
Chromosome 11
CUK1-12
Chromosome 12
CUK1-12

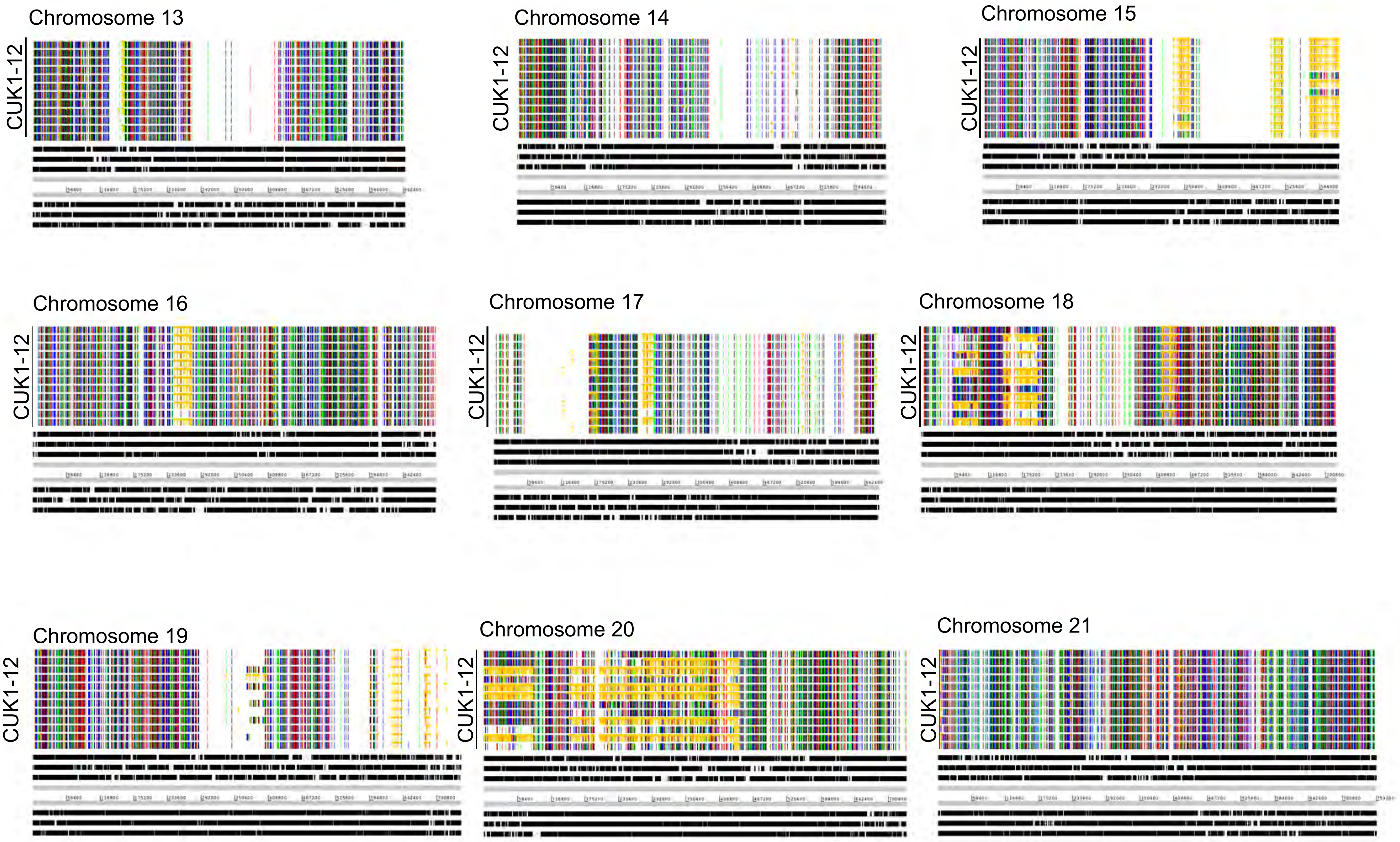
Chromosome 13
CUK1-12
Chromosome 14
CUK1-12
Chromosome 15
CUK1-12
Chromosome 16
CUK1-12
Chromosome 17
CUK1-12
Chromosome 18
CUK1-12
Chromosome 19
CUK1-12
Chromosome 20
CUK1-12
Chromosome 21
CUK1-12

Chromosome 22

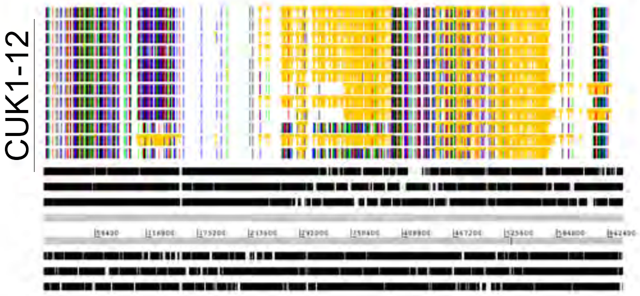

Chromosome 23

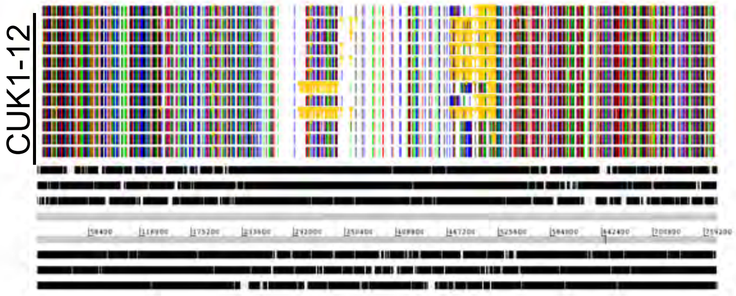

Chromosome 24

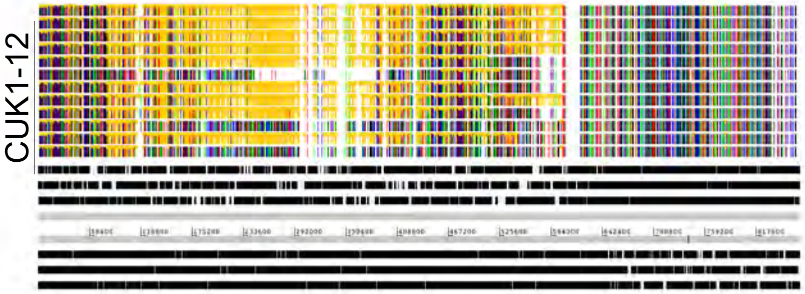

Chromosome 25

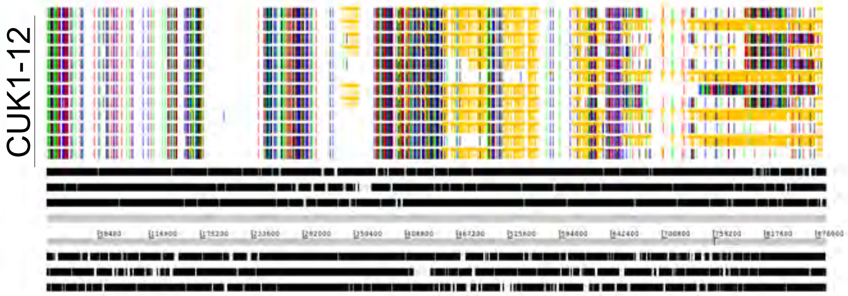

Chromosome 26

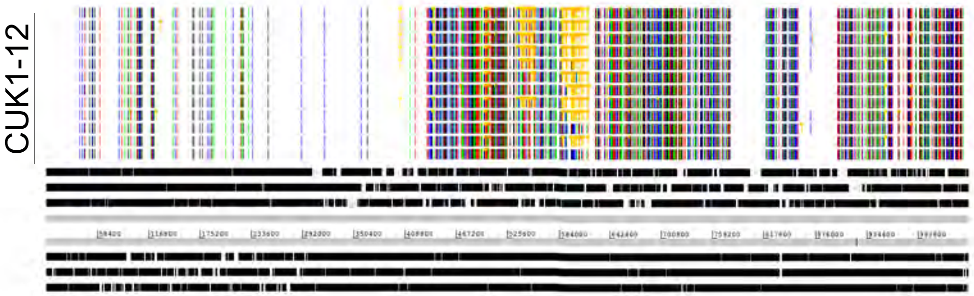

Chromosome 27

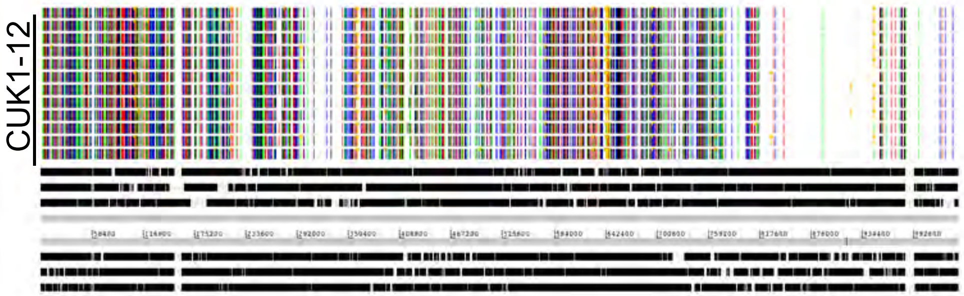

Chromosome 28

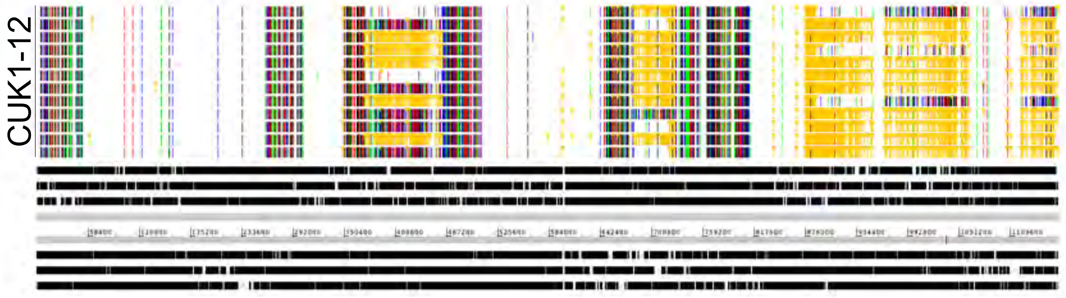

Chromosome 29

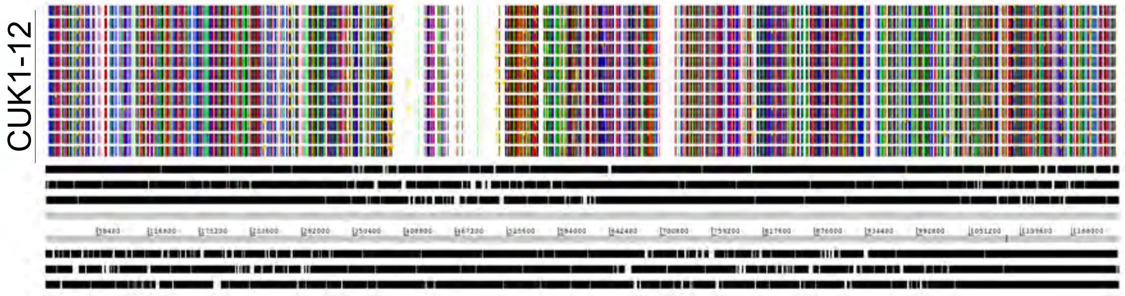

Chromosome 30

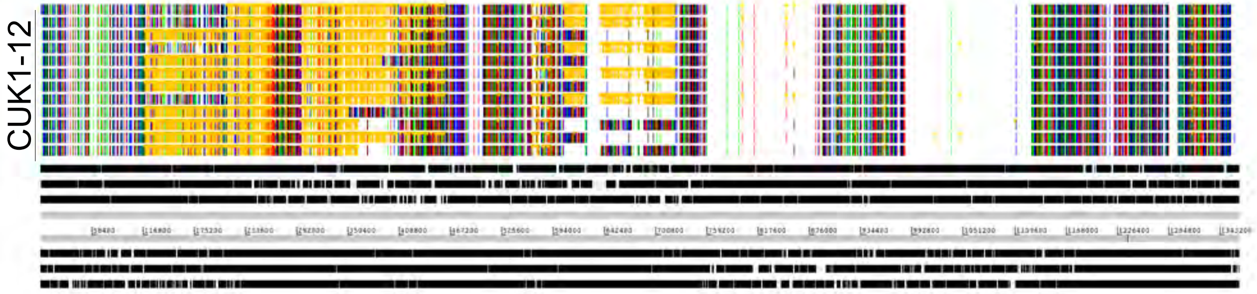

Chromosome 31

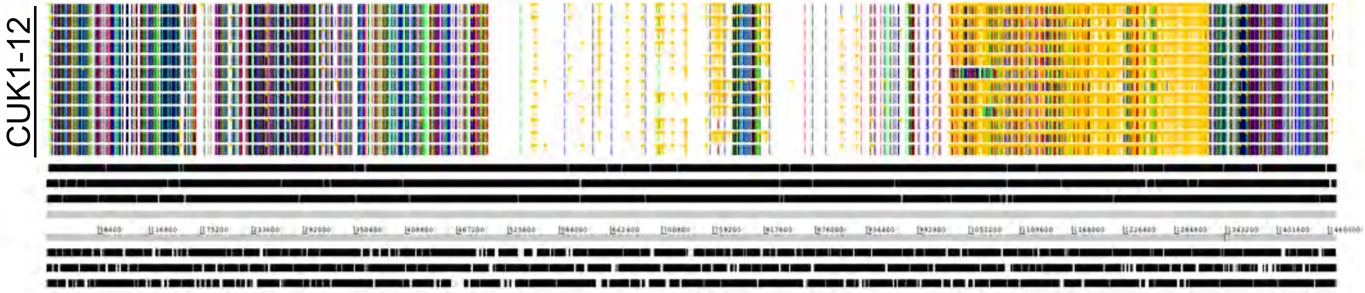

Chromosome 32

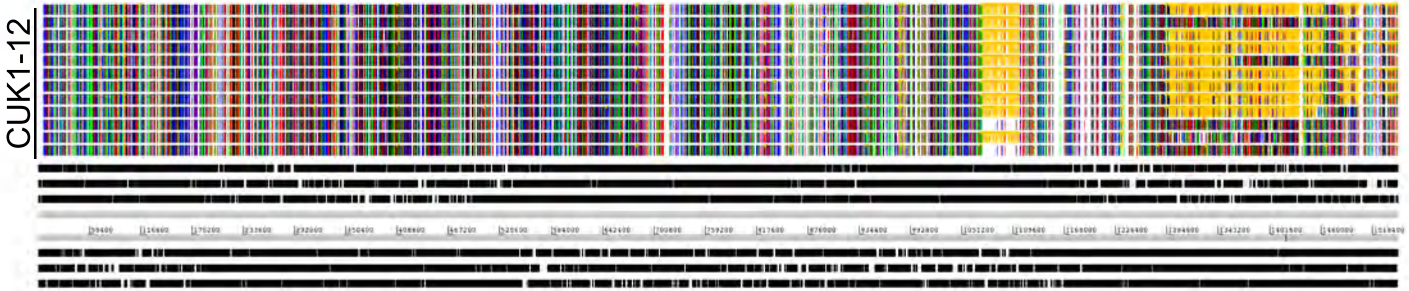

Chromosome 33

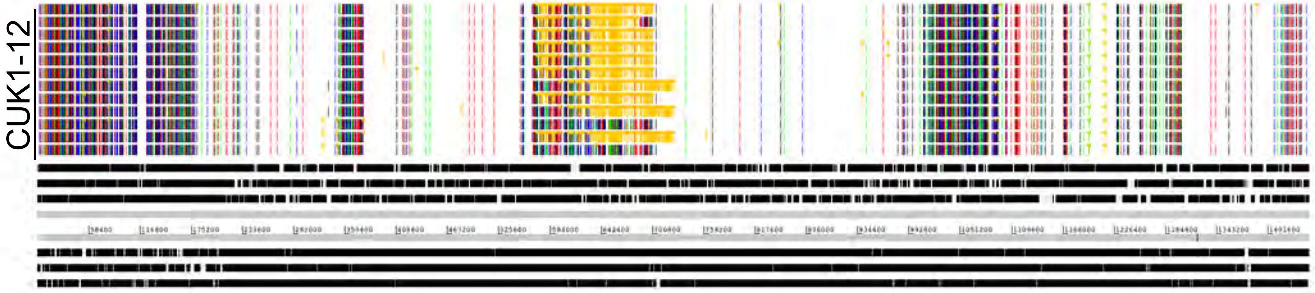

Chromosome 34

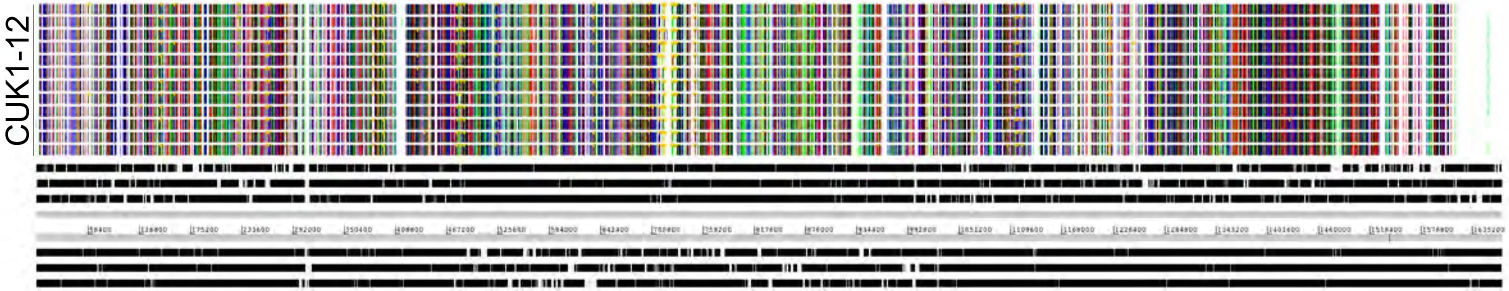

Chromosome 35

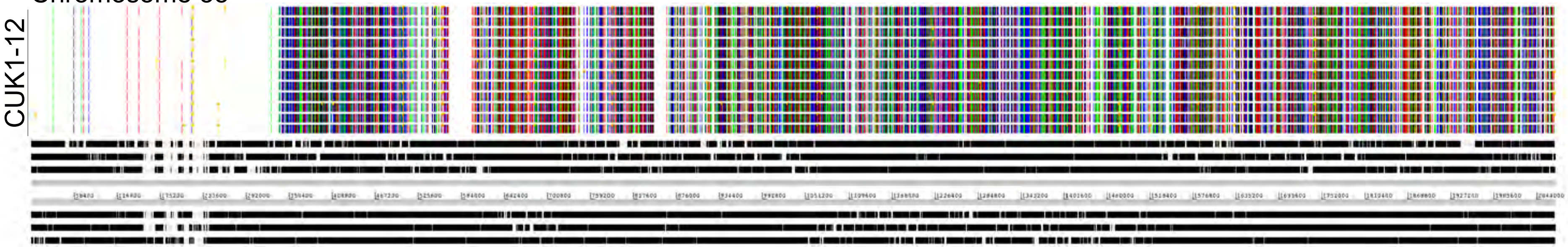

Chromosome 36

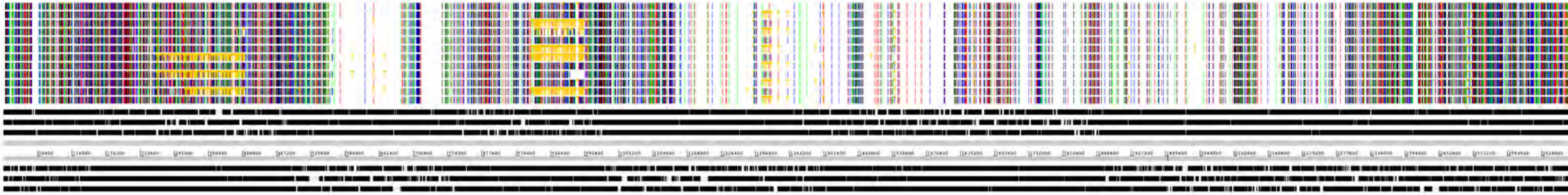

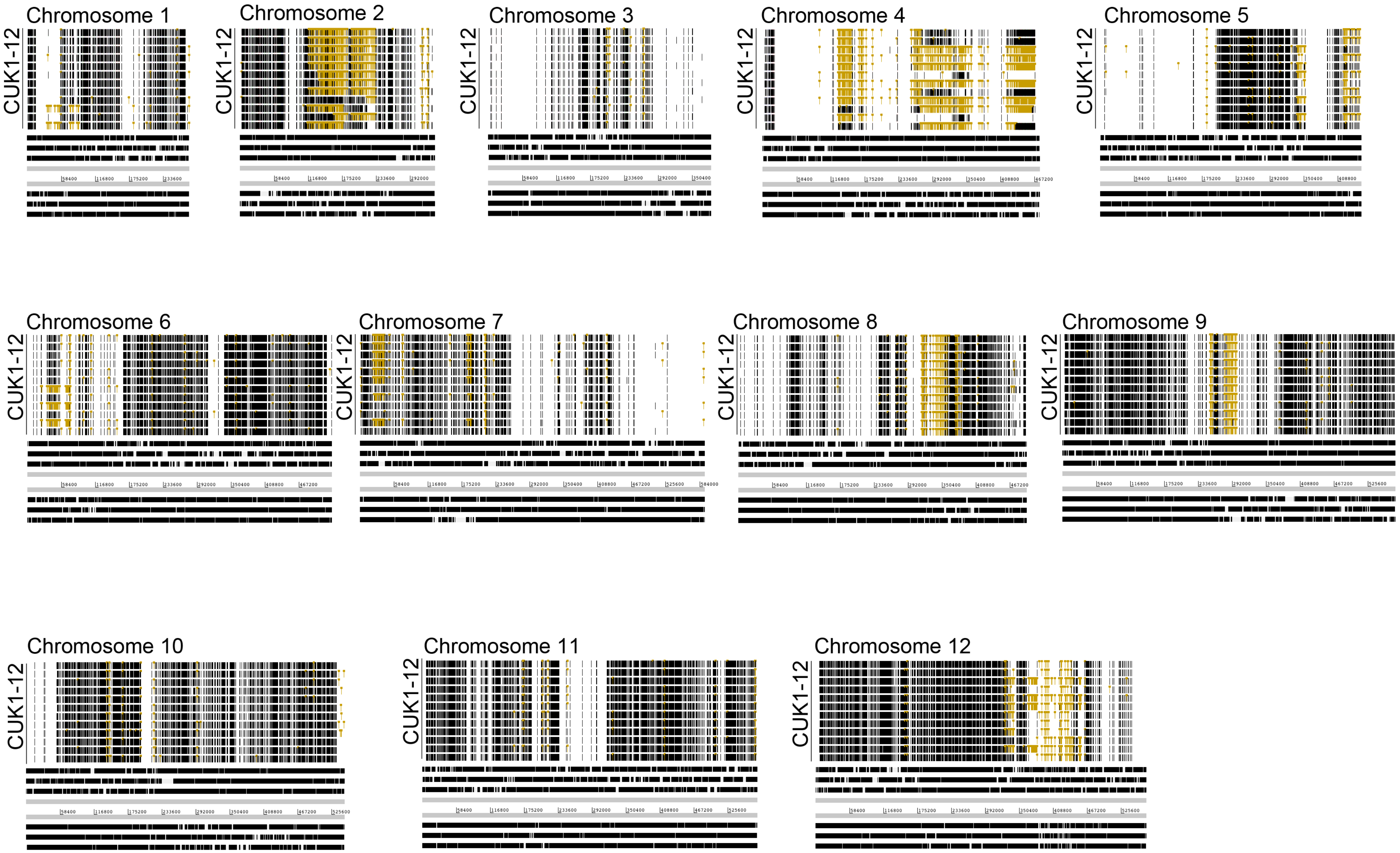
Chromosome 1
CUK1-12
Chromosome 2
CUK1-12
Chromosome 3
CUK1-12
Chromosome 4
CUK1-12
Chromosome 5
CUK1-12
Chromosome 6
CUK1-12
Chromosome 7
CUK1-12
Chromosome 8
CUK1-12
Chromosome 9
CUK1-12
Chromosome 10
CUK1-12
Chromosome 11
CUK1-12
Chromosome 12
CUK1-12

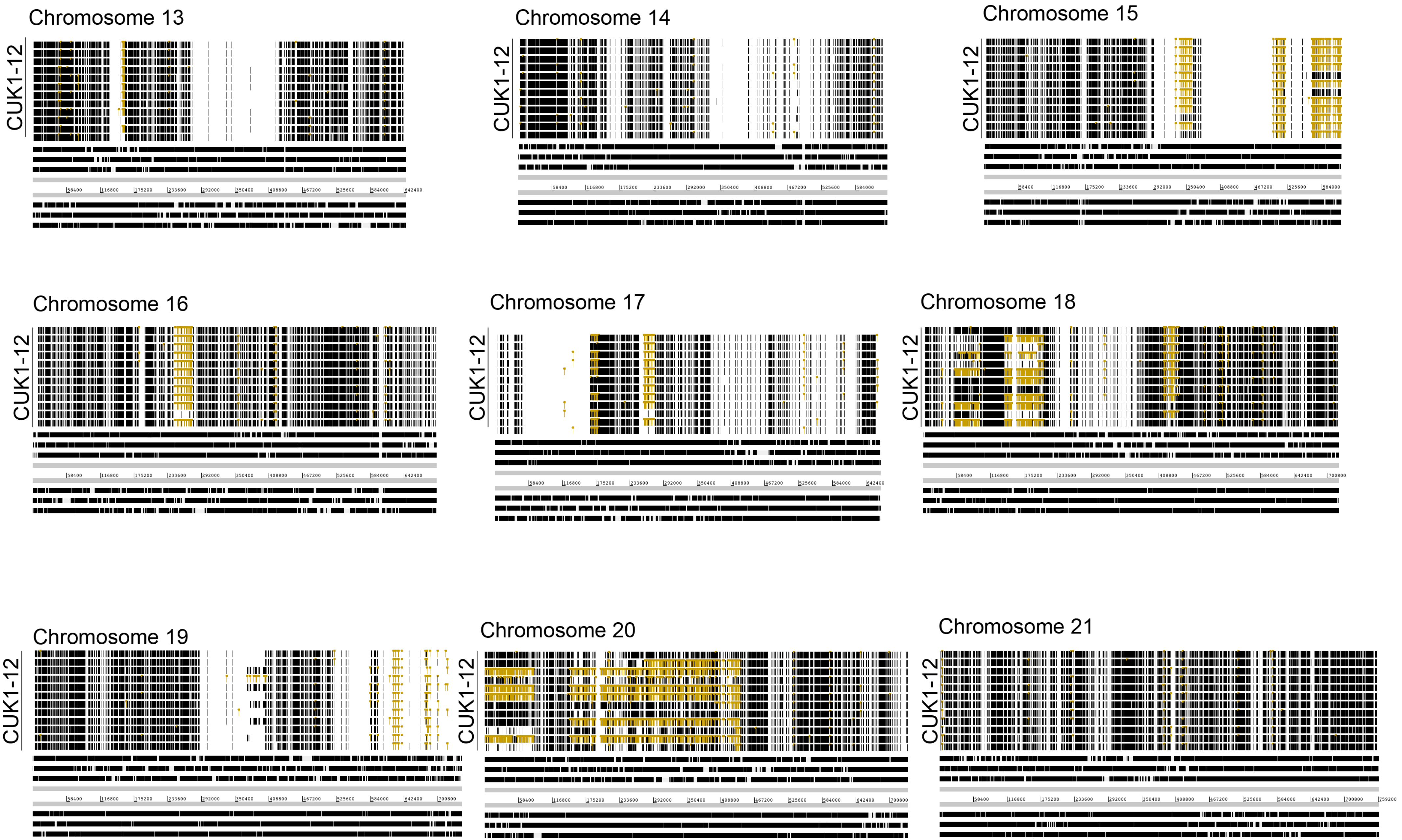
Chromosome 13
CUK1-12
Chromosome 14
CUK1-12
Chromosome 15
CUK1-12
Chromosome 16
CUK1-12
Chromosome 17
CUK1-12
Chromosome 18
CUK1-12
Chromosome 19
CUK1-12
Chromosome 20
CUK1-12
Chromosome 21
CUK1-12

Chromosome 22

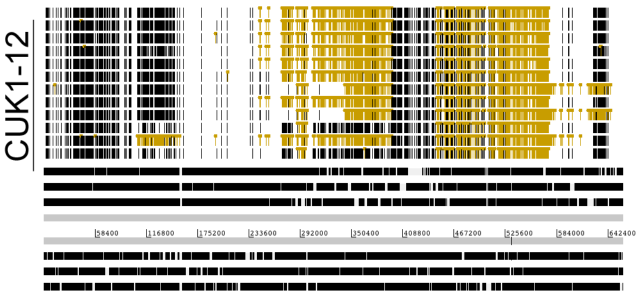

Chromosome 23

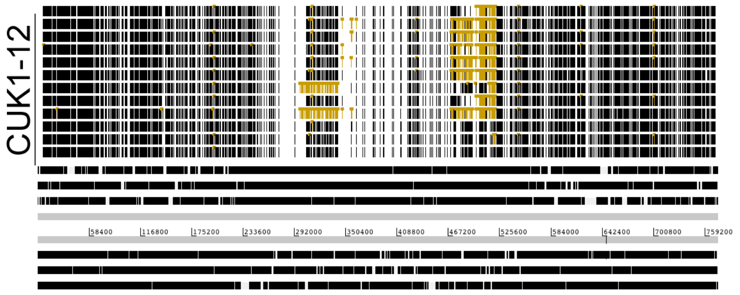

Chromosome 24

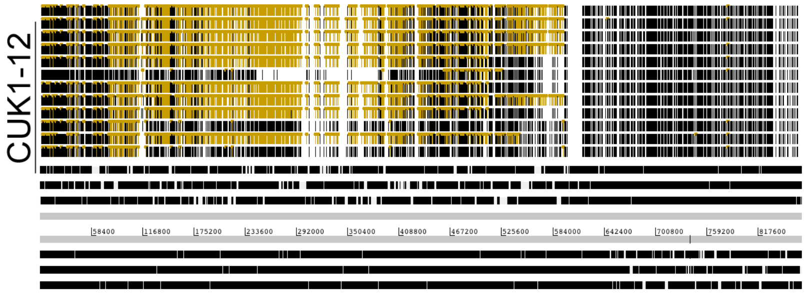

Chromosome 25

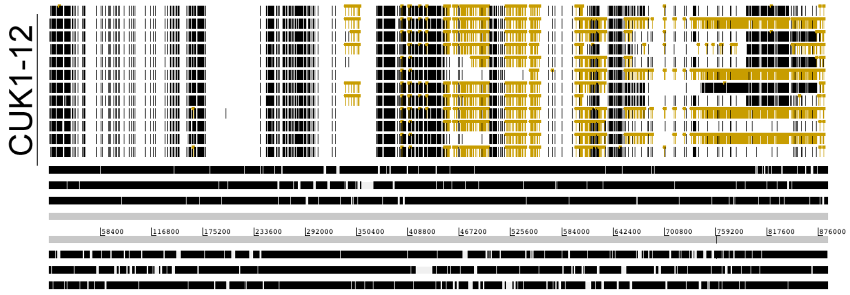

Chromosome 26

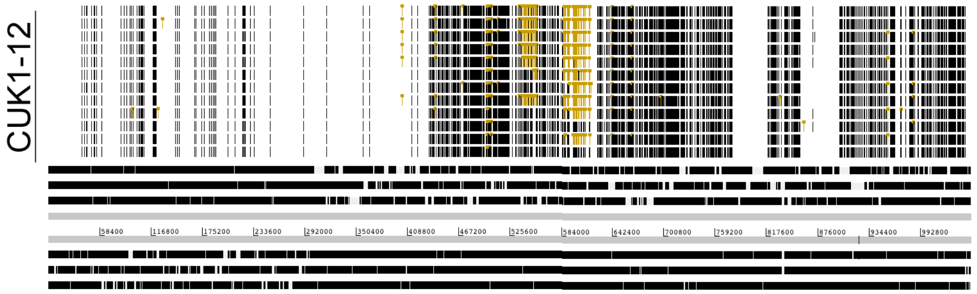

Chromosome 27

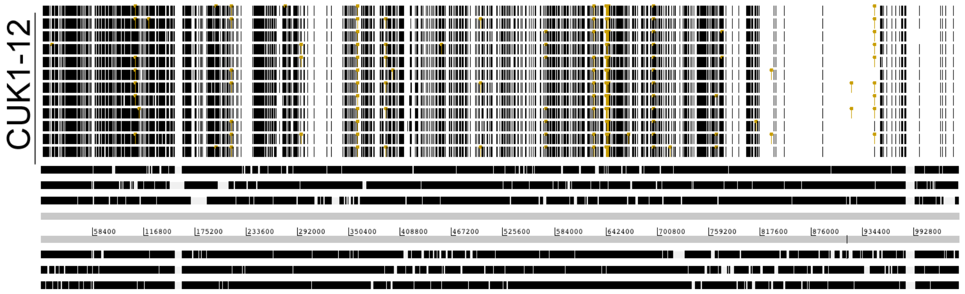

Chromosome 28

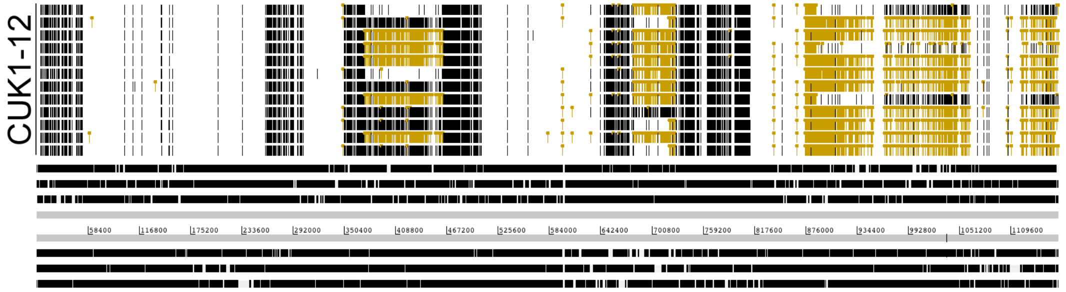

Chromosome 29

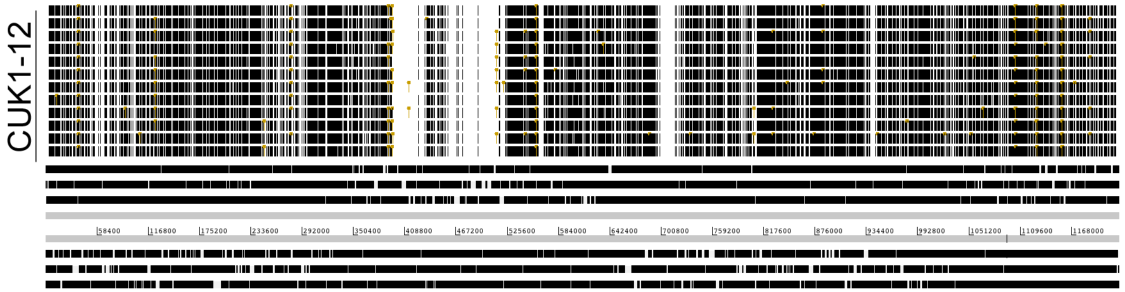

Chromosome 30

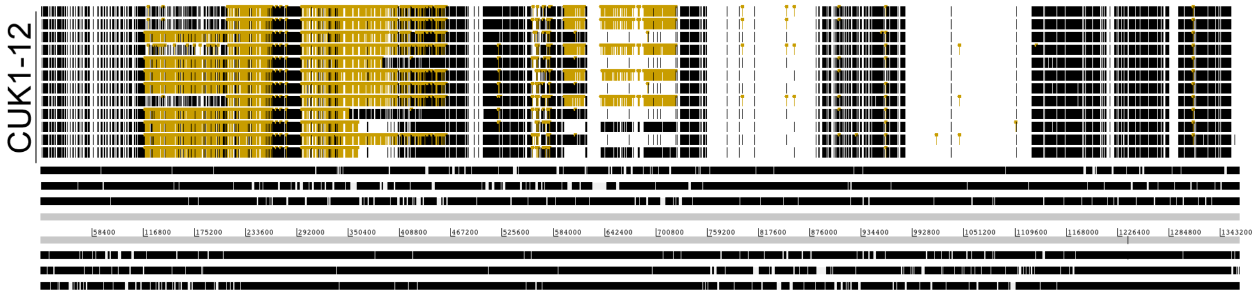

Chromosome 31

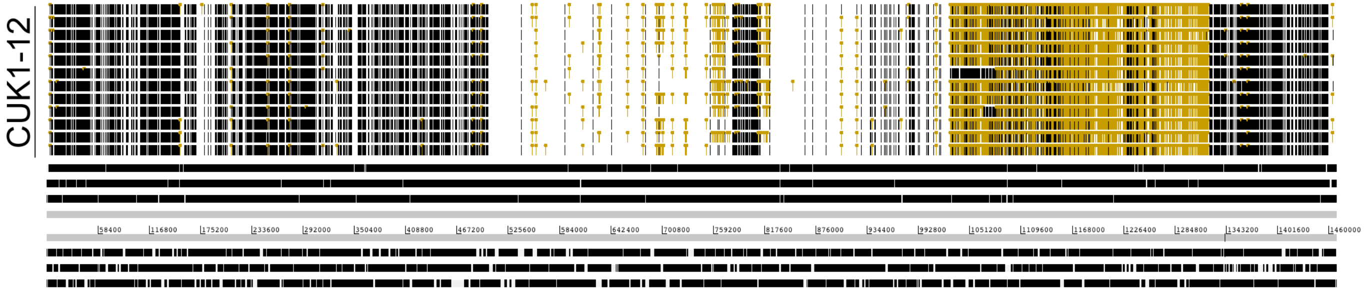

Chromosome 32

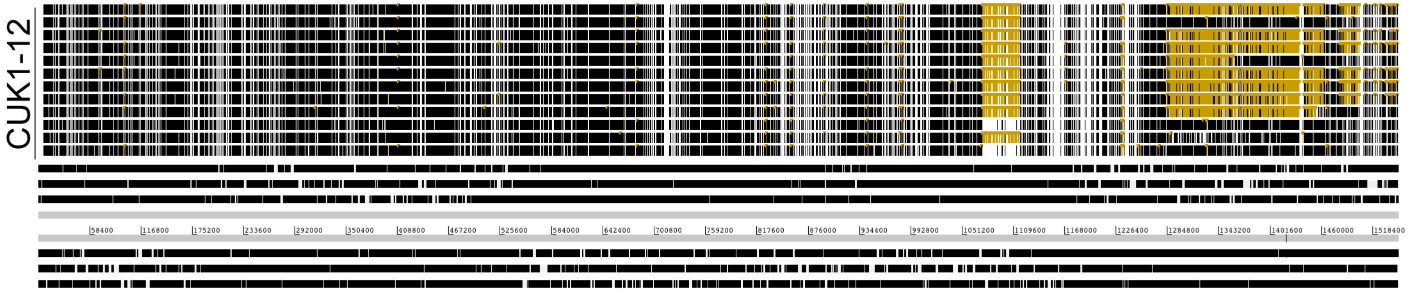

Chromosome 33

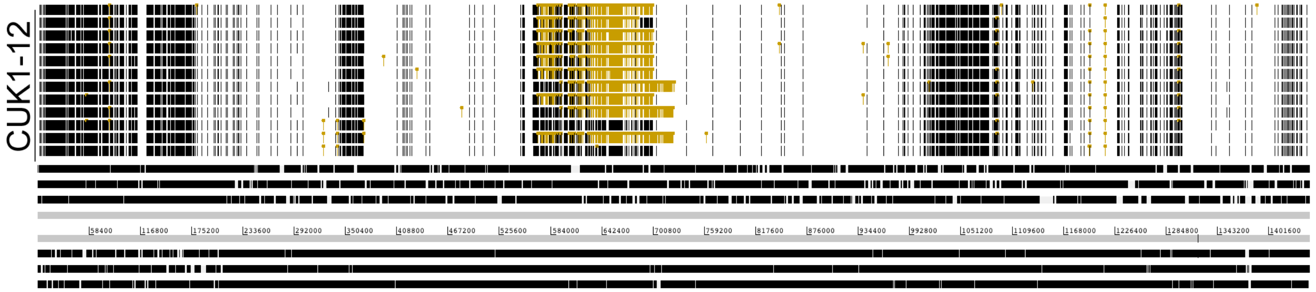

Chromosome 34

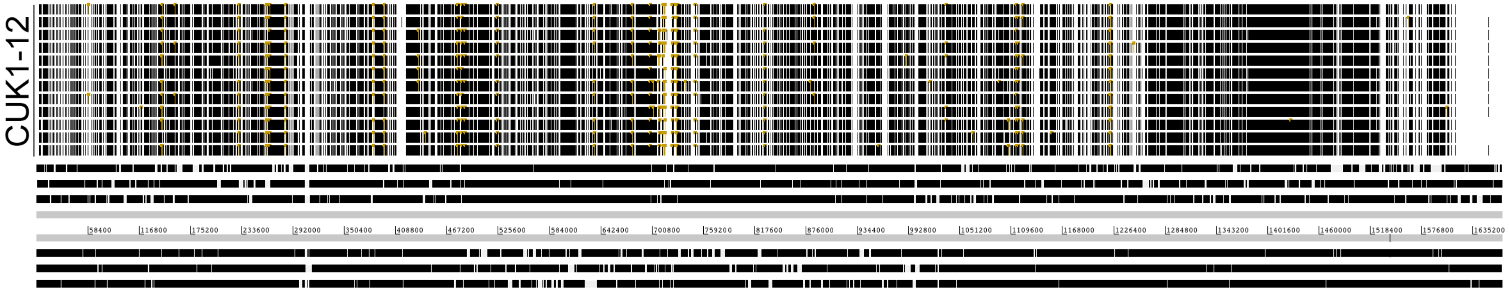

Chromosome 35

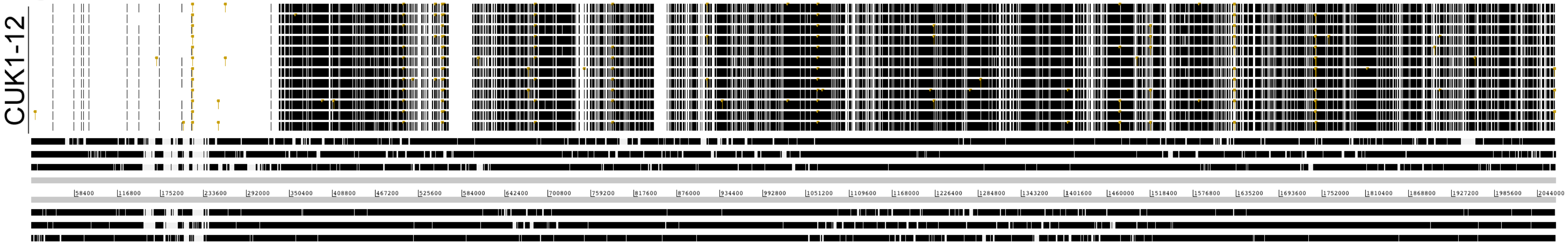

Chromosome 36

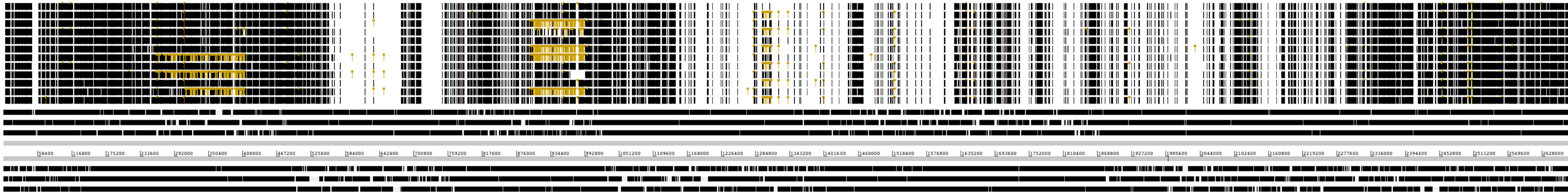

Supplement: Figure S4 — Genome-wide patterns of polymorphism in the CUK strains. (A) All single-nucleotide polymorphisms (SNPs) are shown with respect to L. infantum JCPM5 in all chromosomes for all 12 strains. Orange ‘blocked’ bars indicate heterozygous positions; green, red, blue and black bars indicate homozygous variant calls of A, C, G and T respectively. Isolates 1–12 are shown in order from the bottom to top track of the figures for each chromosome. (B) The same plots in (A) are shown with orange ‘blocked’ bars indicating heterozygous positions and black bars indicating homozygous variant calls. (ZIP) [file pgen.1004092.s004.zip › Figure S4.pdf]
