# Supplementary material for: Genomic Confirmation of Hybridisation and Recent Inbreeding in a Vector-Isolated Leishmania Population
Source: PLoS Genet. 2014 Jan 16;10(1):e1004092. doi: 10.1371/journal.pgen.1004092 (PMC3894156; doi:10.1371/journal.pgen.1004092)

Figure S8A. Phylogenies of phased sites that split haplotypes into 2 distinct clades

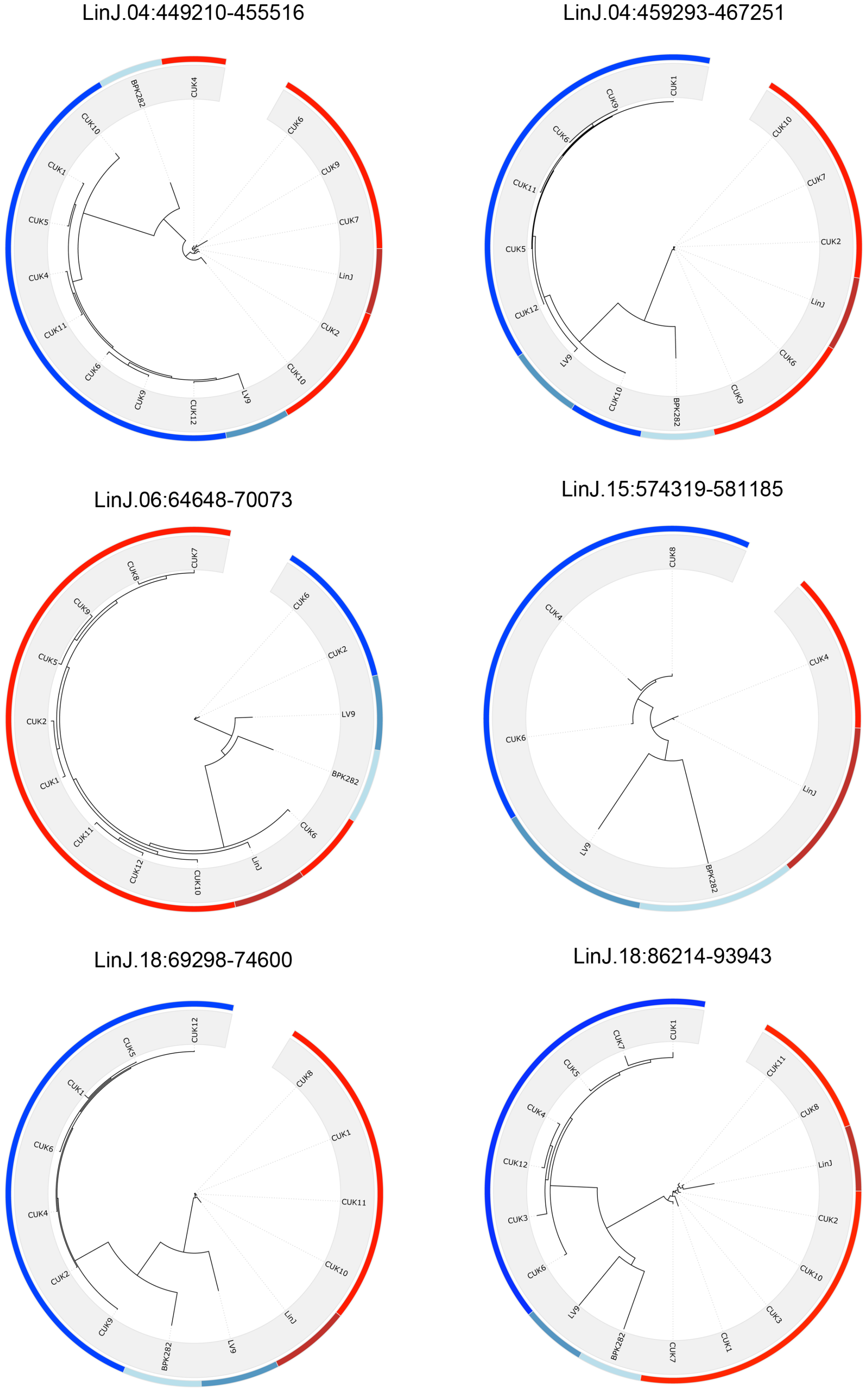

LinJ.20:249270-254568

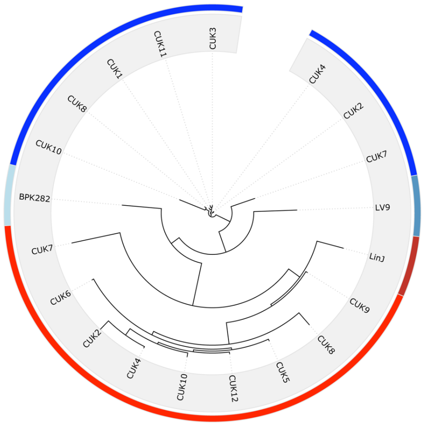

LinJ.20:260564-266481

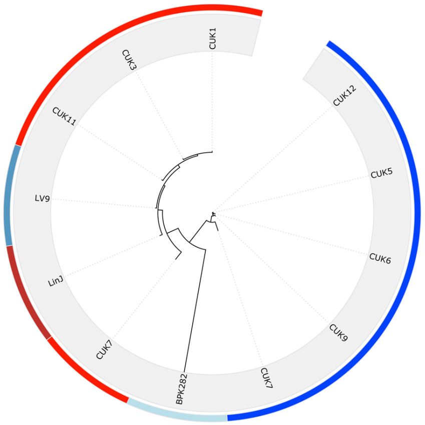

LinJ.20:282669-293094

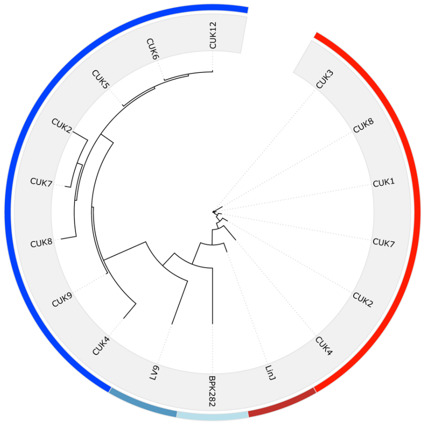

LinJ.20:388933-394463

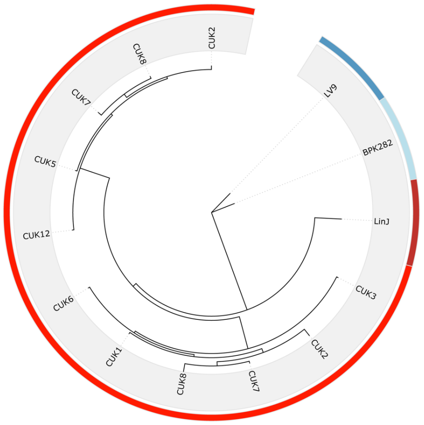

LinJ.22:130574-136277

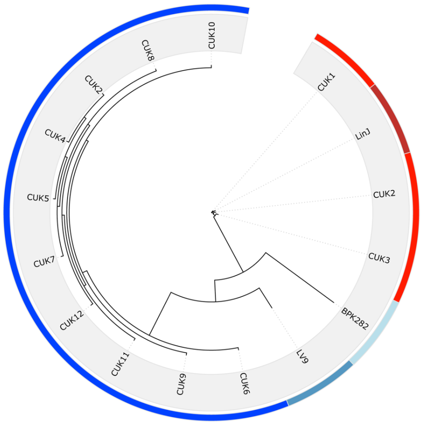

LinJ.22:357126-362978

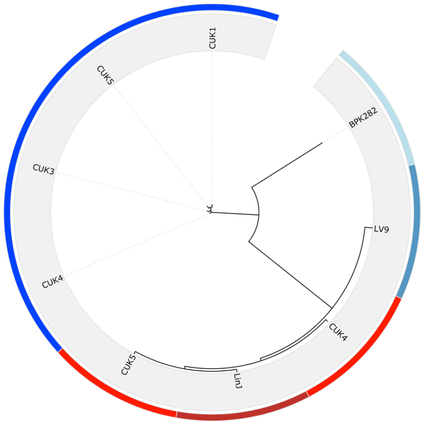

LinJ.22:517430-523241

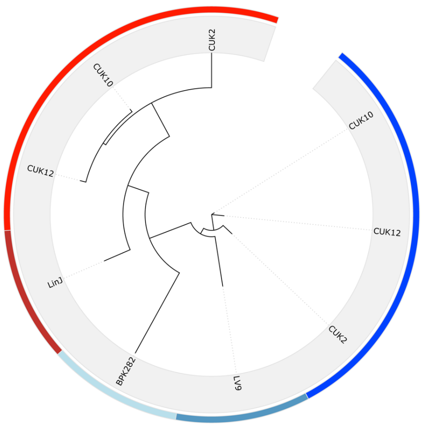

LinJ.22:560728-566558

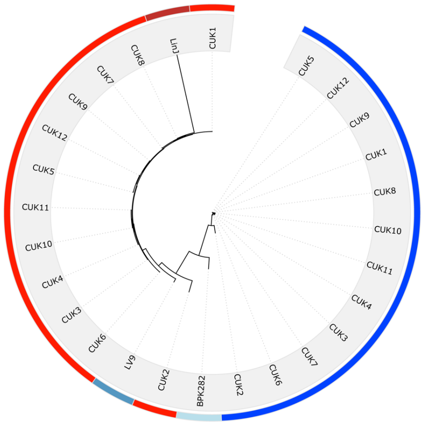

LinJ.22:569430-574457

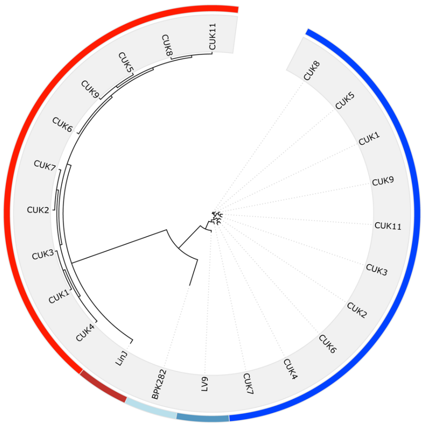

LinJ.23:487317-494203

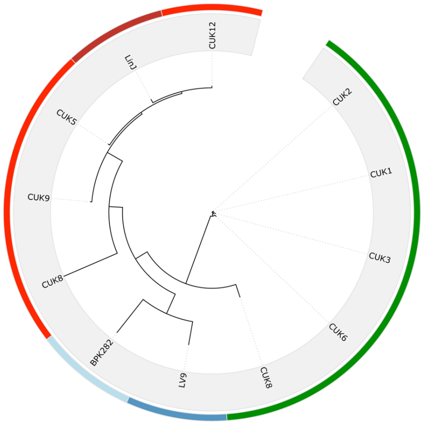

LinJ.24:166522-170956

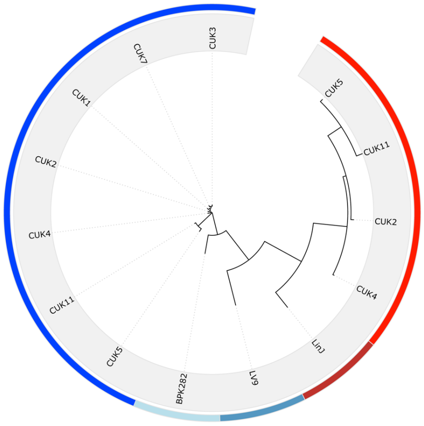

LinJ.24:283189-291818

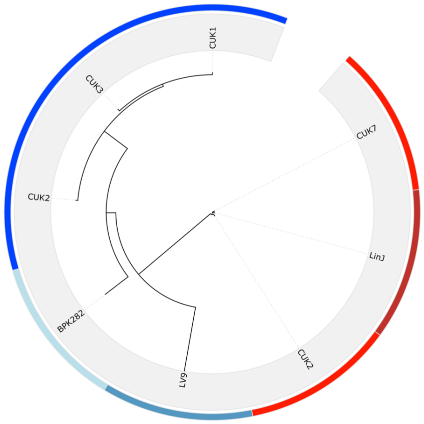

LinJ.24:460086-465883

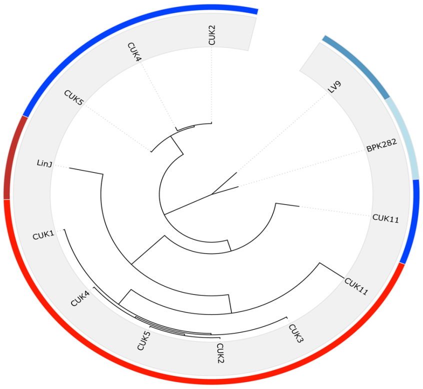

LinJ.25:763395-769691

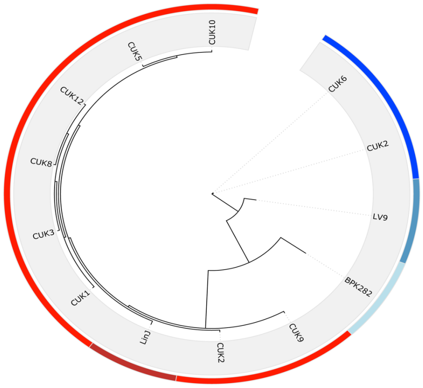

LinJ.25:800101-806017

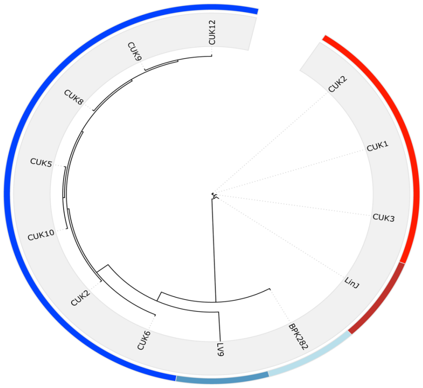

LinJ.25:810784-817004

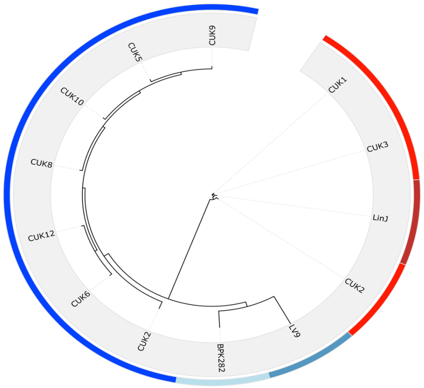

LinJ.25:830911-837533

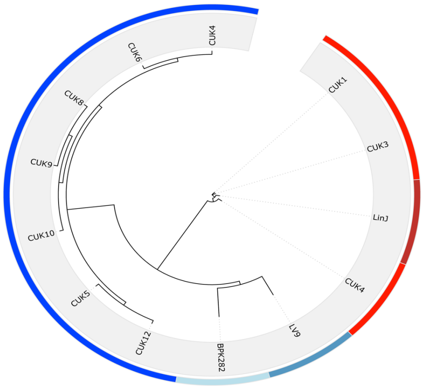

LinJ.28:389167-395657

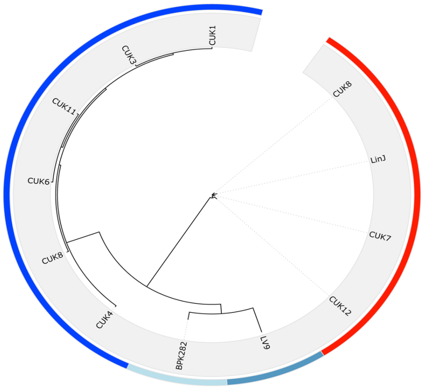

LinJ.28:692069-696673

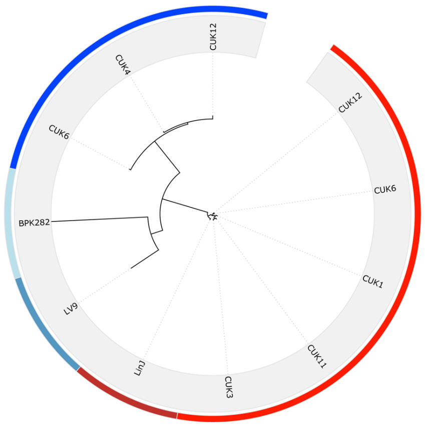

LinJ.28:709935-715210

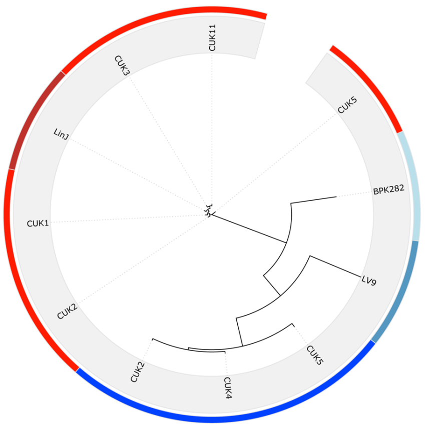

LinJ.28:884359-892076

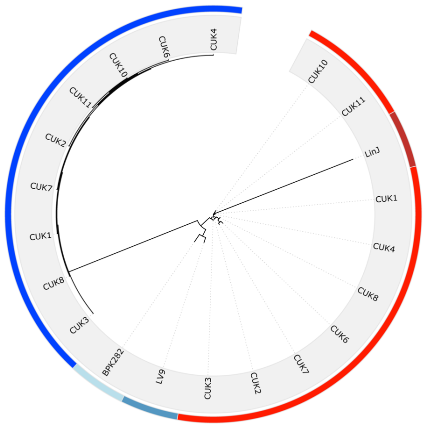

LinJ.28:904266-909811

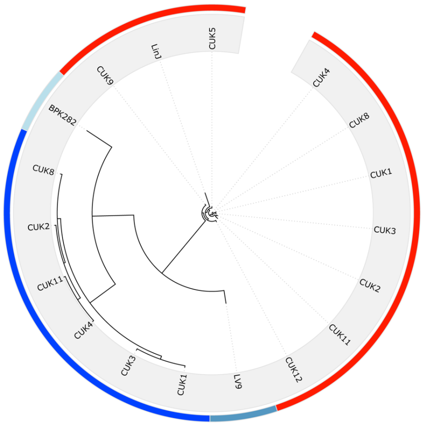

LinJ.28:911099-918442

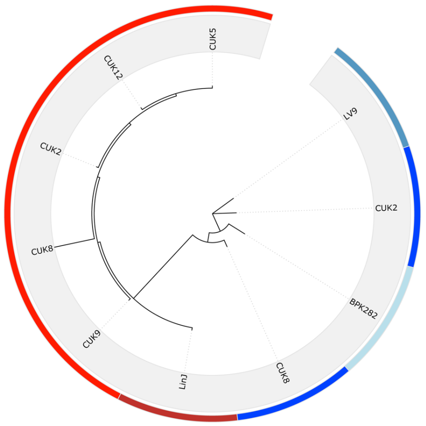

LinJ.30:126396-134786

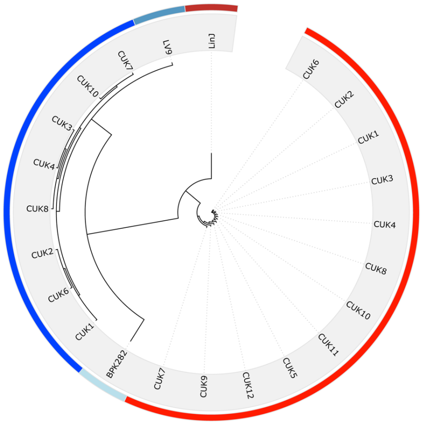

LinJ.30:147138-152234

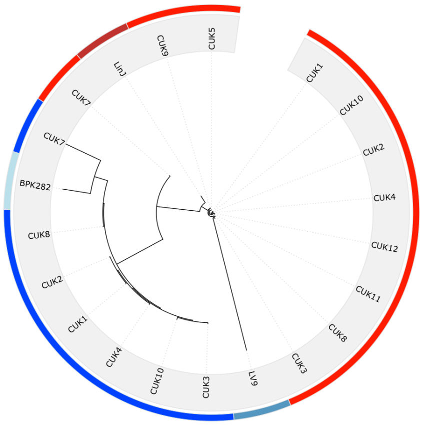

LinJ.30:192738-199344

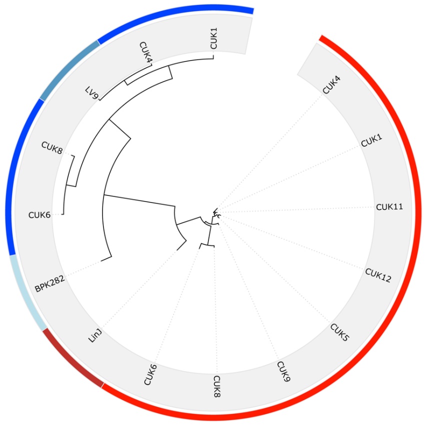

LinJ.30:229882-233565

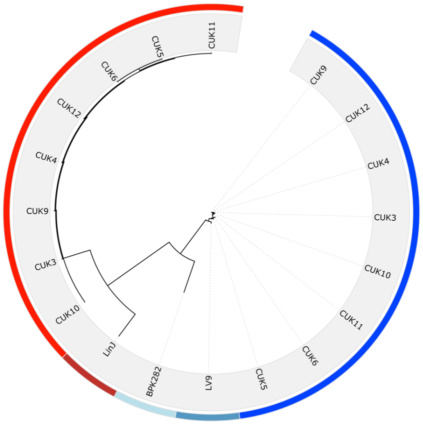

LinJ.30:253015-258382

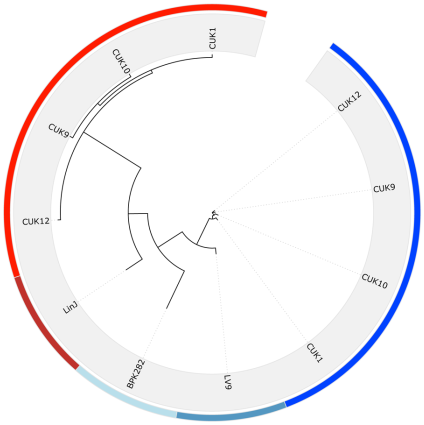

LinJ.30:348662-354563

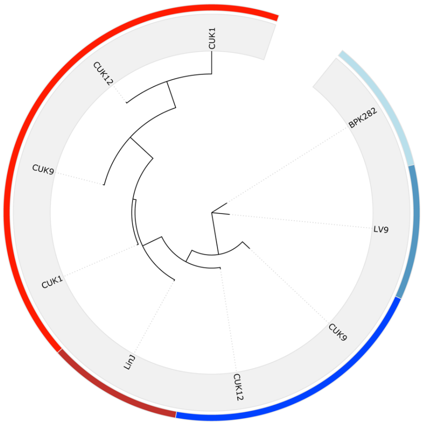

LinJ.30:449969-455055

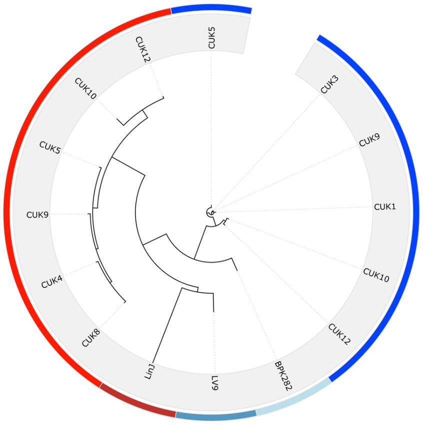

LinJ.30:596044-610110

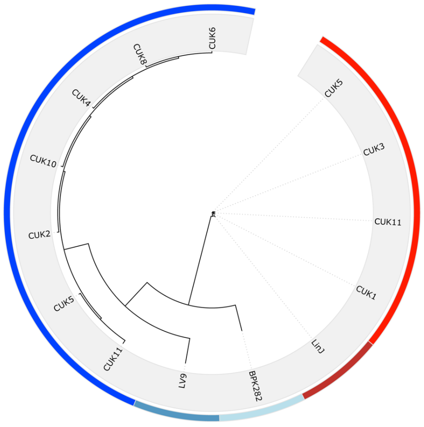

LinJ.30:708341-716284

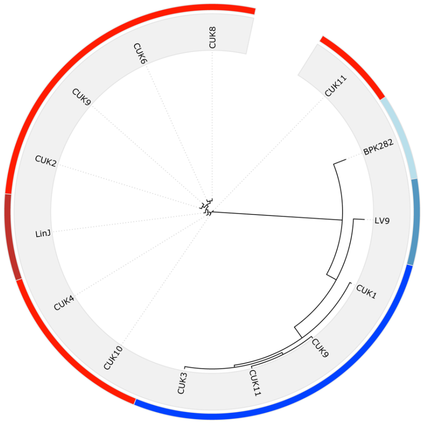

LinJ.31:762603-767918

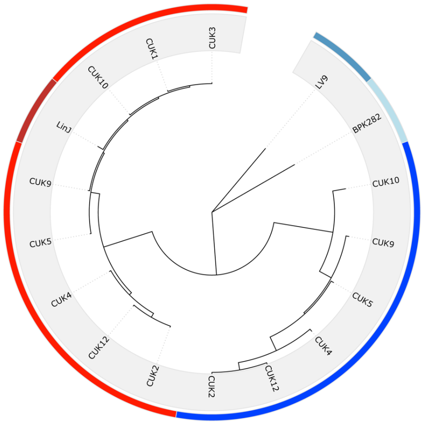

LinJ.31:1049672-1052348

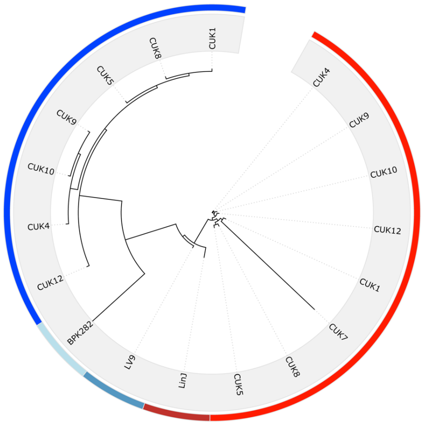

LinJ.31:1104988-1110043

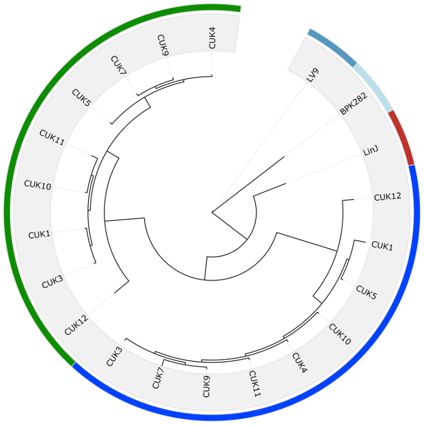

LinJ.31:1120603-1125945

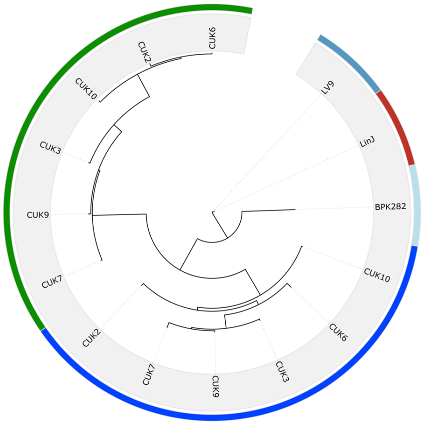

LinJ.31:1134743-1137038

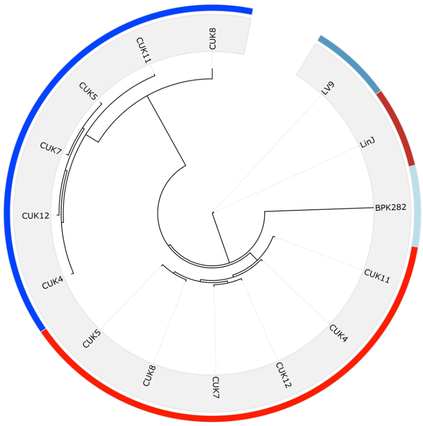

LinJ.31:1148753-1153776

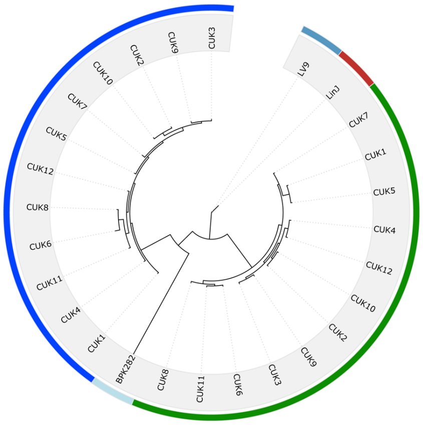

LinJ.31:1157215-1162577

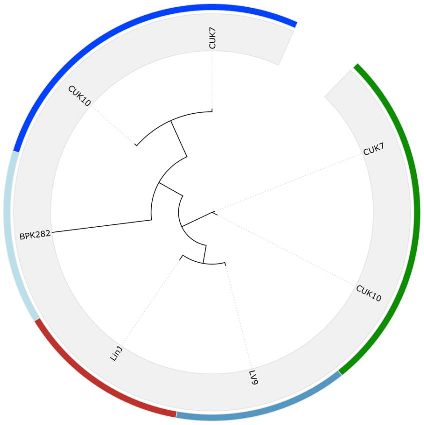

LinJ.31:1176231-181083

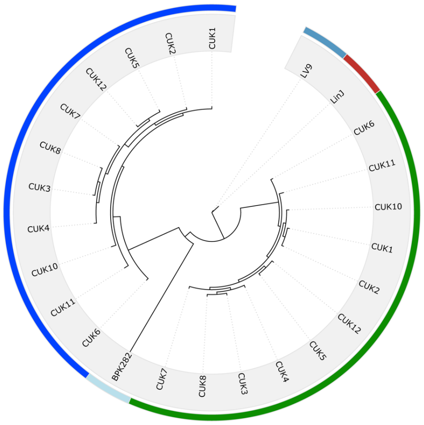

LinJ.31:1203777-1208607

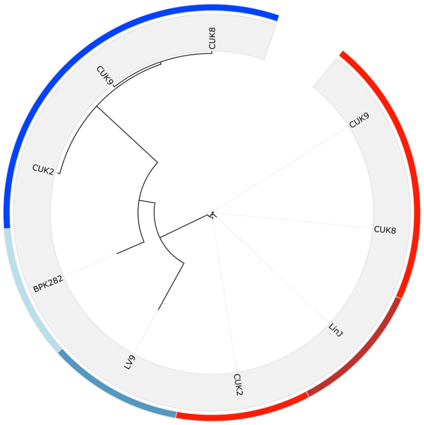

LinJ.31:1231038-1236915

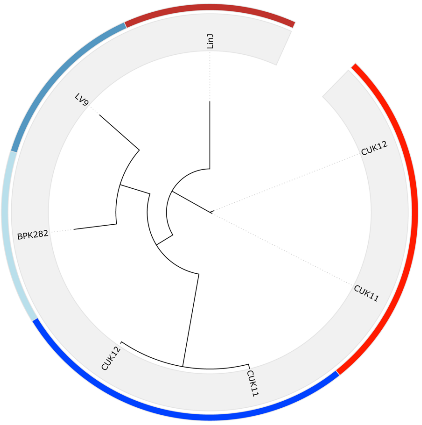

LinJ.31:1293289-1298458

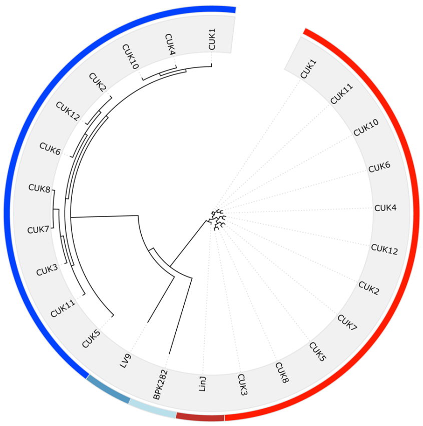

LinJ.32:1287340-1291973

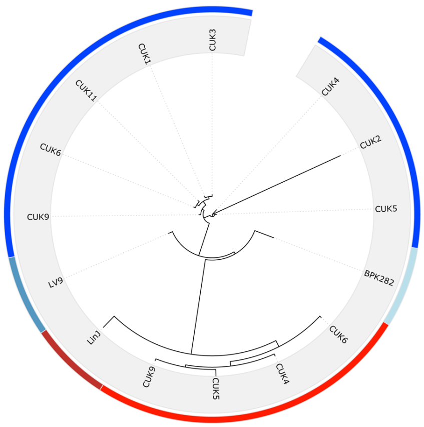

LinJ.32:1302487-1308247

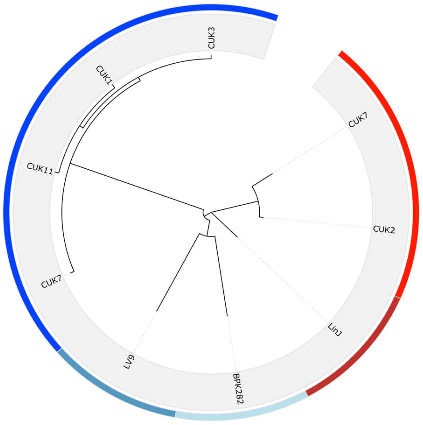

LinJ.32:1314120-1320130

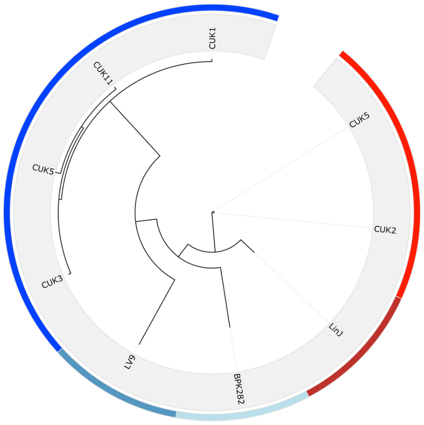

LinJ.32:1318973-1324945

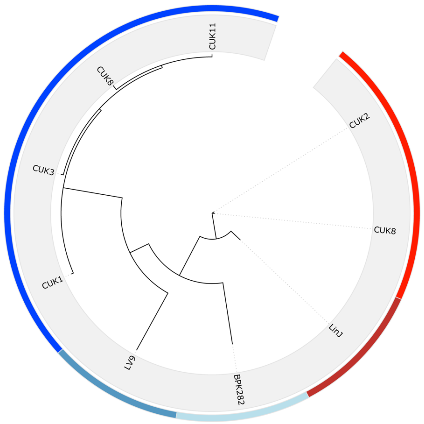

LinJ.32:1335455-1341677

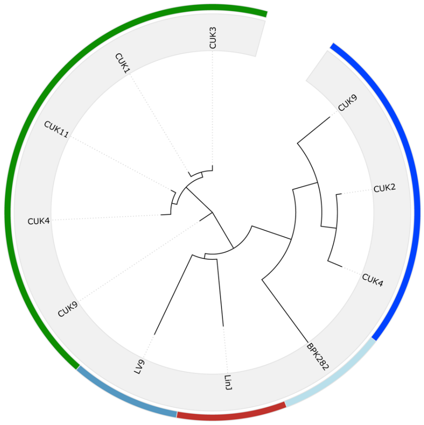

LinJ.32:1370359-1374522

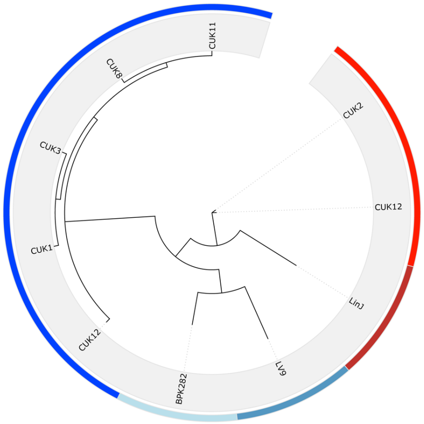

LinJ.32:1394804-1400764

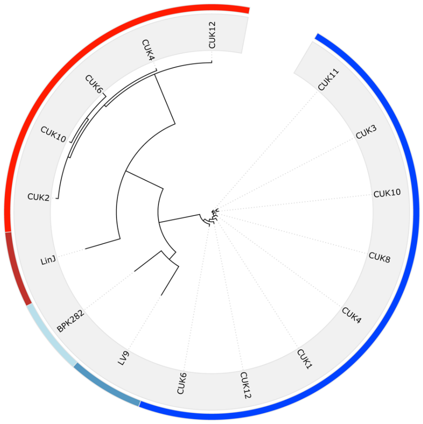

LinJ.32:1417202-1426971

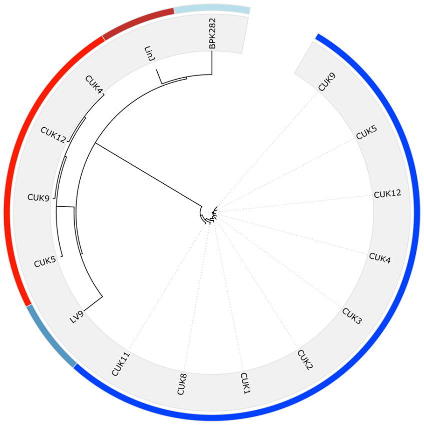

LinJ.32:1442360-1449850

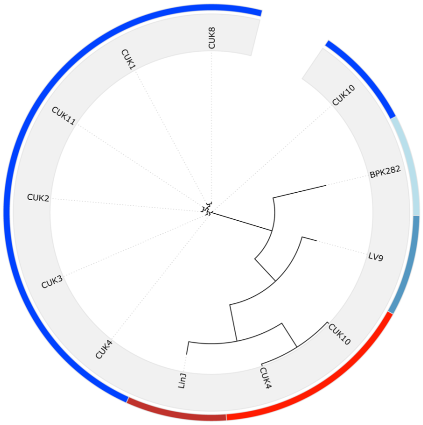

LinJ.32:1456581-1462029

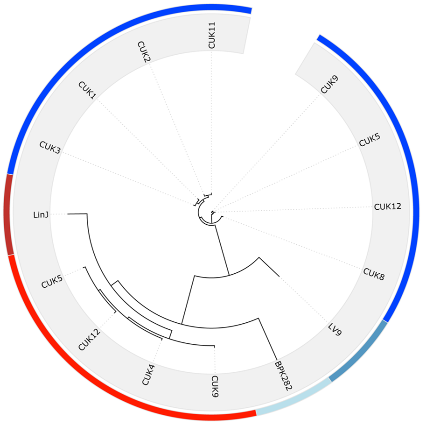

LinJ.33:656919-662144

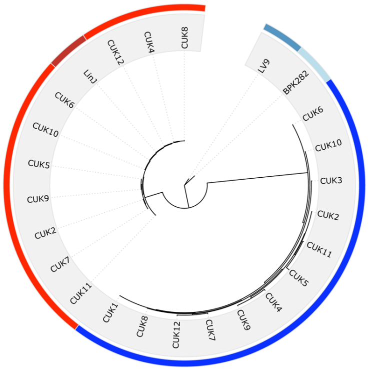

Figure S8B. Phylogenies of phased sites which fail to reconstruct 2 distinct clades.

LinJ.22:294640-300111

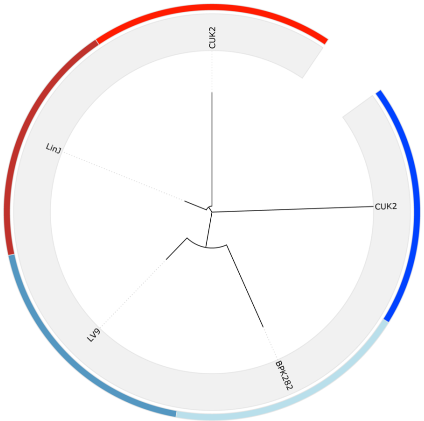

LinJ.22:349990-356220

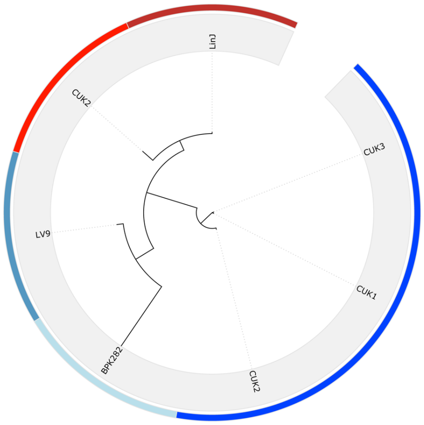

LinJ.22:536378-542307

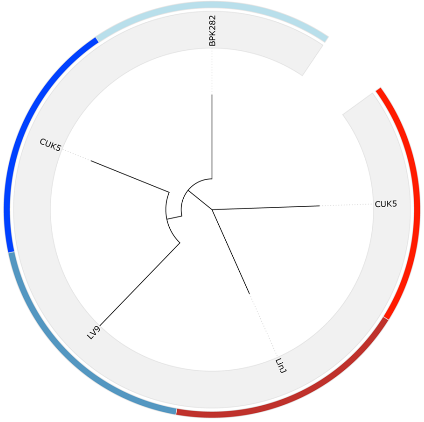

LinJ.22:629481-639429

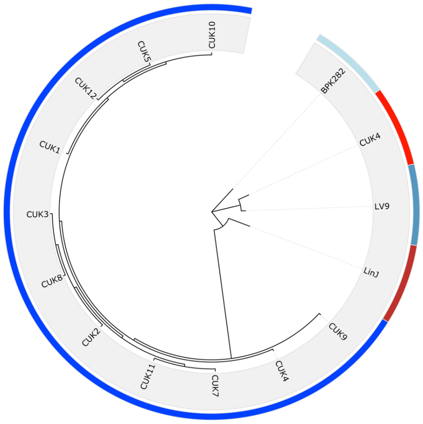

LinJ.24:89857-93534

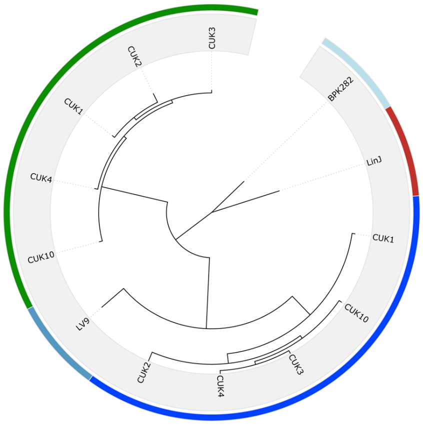

LinJ.24:139469-146710

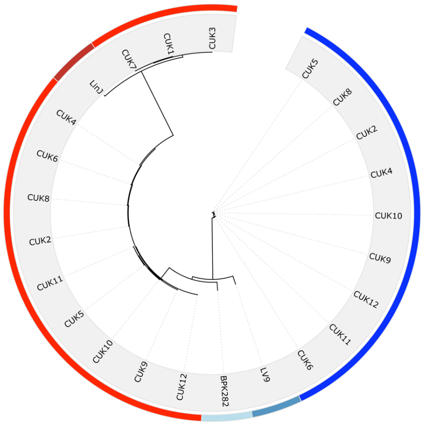

LinJ.30:338617-344387

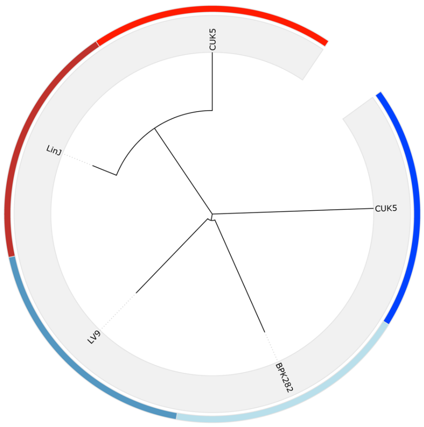

LinJ.30:694274-704362

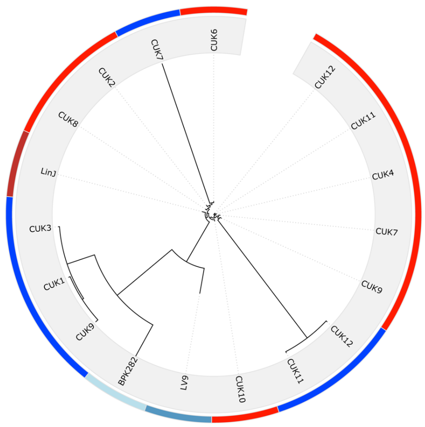

LinJ.31:1030268-1036249

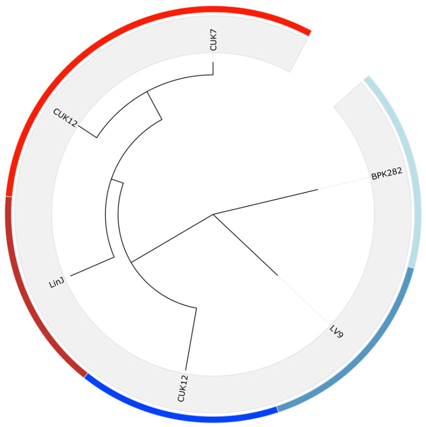

LinJ.31:1076440-1081476

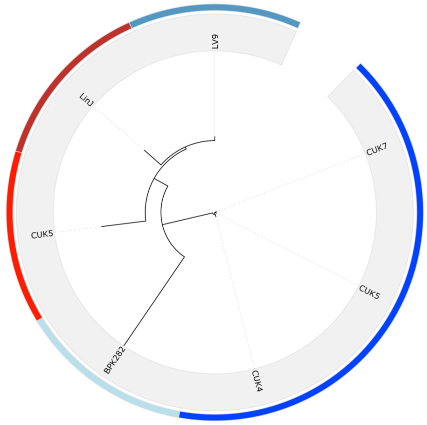

LinJ.36:325472-330802

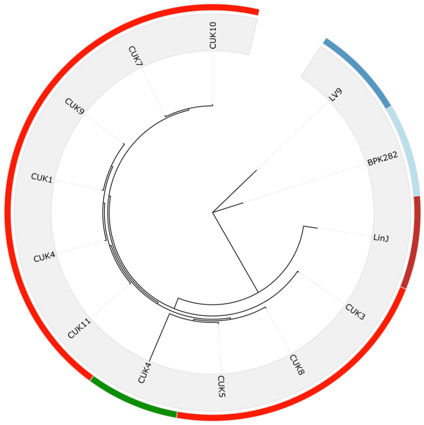

LinJ.36:353451-358936

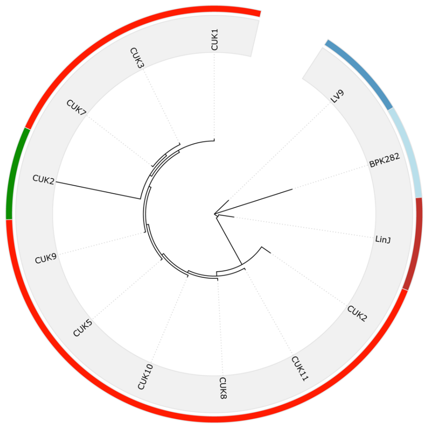

LinJ.36:381464-388903

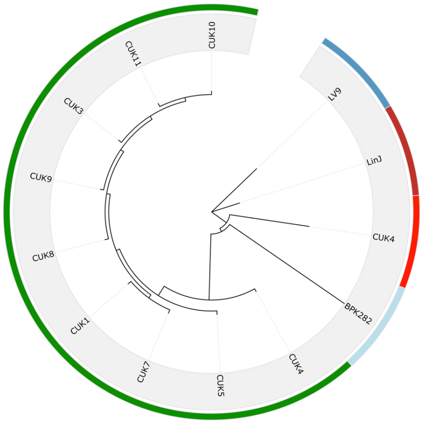

LinJ.24:128176-133206

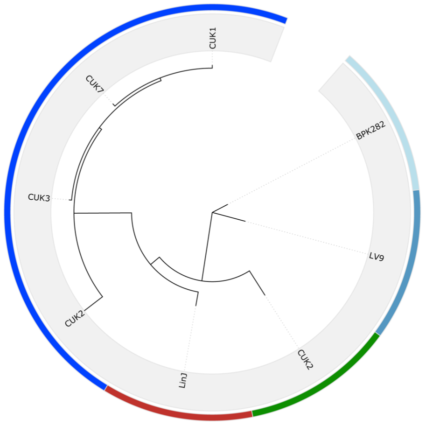

Supplement: Figure S8 — Maximum-likelihood phylogenies of CUK isolates and sequenced L. donovani complex genomes based on phased haplotypes. Maximum-likelihood phylogenies are shown for all regions in which at least one CUK isolate could be phased to produce haplotype sequences of at least 5 kb. (A) 60 out of 74 phylogenies show different haplotypes for CUK isolates from two different clades, while (B) 14 do not show this pattern. Titles of each phylogeny indicate the chromosome number and location of phased region on the JPCM5 reference sequence. Colours indicate sets of different haplotypes, with the three reference genomes consistently coloured throughput. (PDF) [file pgen.1004092.s008.pdf]
