# Supplementary material for: Genomic Confirmation of Hybridisation and Recent Inbreeding in a Vector-Isolated Leishmania Population
Source: PLoS Genet. 2014 Jan 16;10(1):e1004092. doi: 10.1371/journal.pgen.1004092 (PMC3894156; doi:10.1371/journal.pgen.1004092)
Supplement: Figure S13 — Linkage disequilibrium per chromosome in the CUK population. Values displayed are the average LD (r2) for all SNPs segregating in the CUK population, between pairs of 10 kb blocks scaled from low (blue) to high (beige) LD for each pair of blocks on the same chromosome, for each chromosome in the L. infantum genome. (ZIP) [file pgen.1004092.s013.zip › Figure S13.pdf]

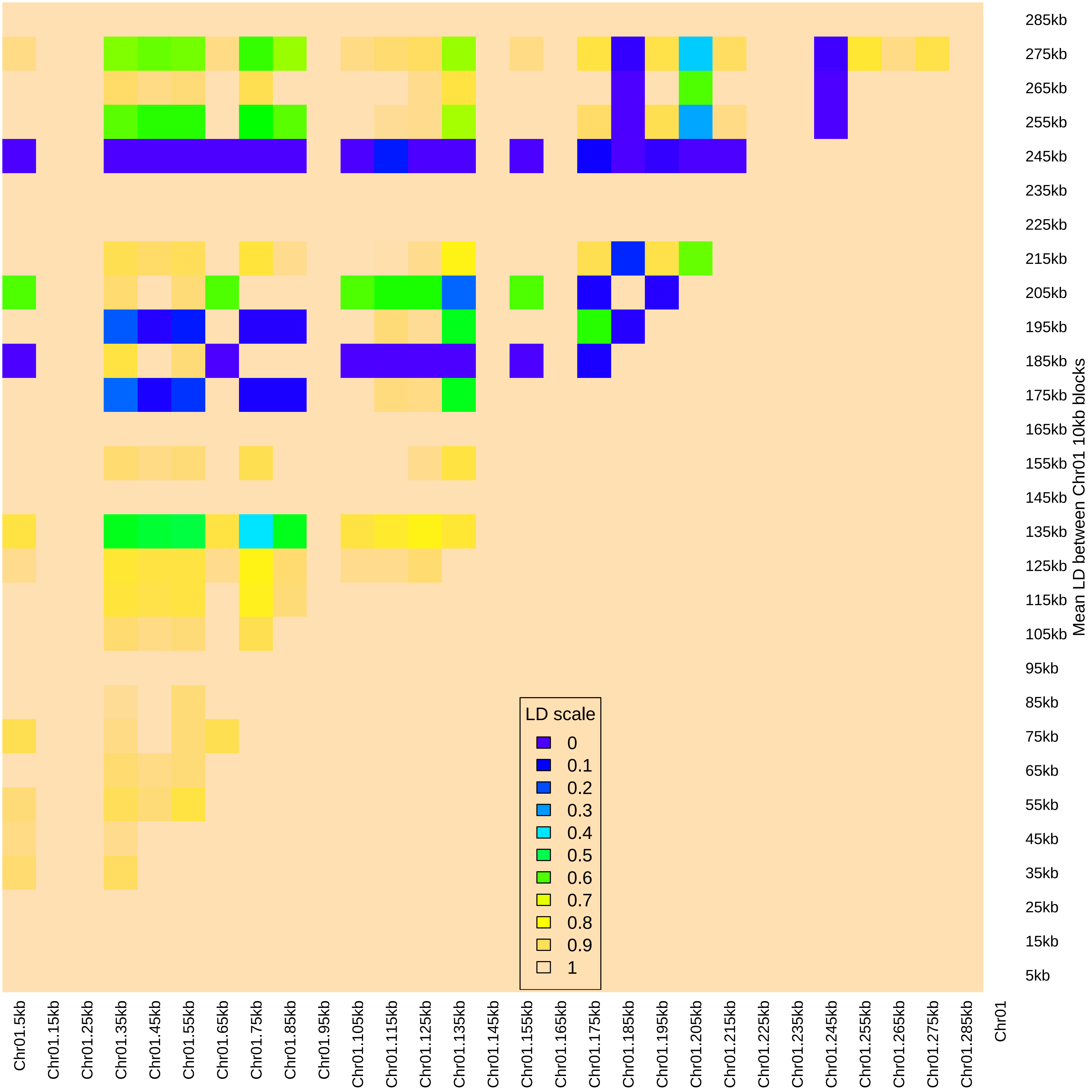

LD scale
0
0.1
0.2
0.3
0.4
0.5
0.6
0.7
0.8
0.9
1
Mean LD between Chr01 10kb blocks
285kb
275kb
265kb
255kb
245kb
235kb
225kb
215kb
205kb
195kb
185kb
175kb
165kb
155kb
145kb
135kb
125kb
115kb
105kb
95kb
85kb
75kb
65kb
55kb
45kb
35kb
25kb
15kb
5kb
Chr01.5kb
Chr01.15kb
Chr01.25kb
Chr01.35kb
Chr01.45kb
Chr01.55kb
Chr01.65kb
Chr01.75kb
Chr01.85kb
Chr01.95kb
Chr01.105kb
Chr01.115kb
Chr01.125kb
Chr01.135kb
Chr01.145kb
Chr01.155kb
Chr01.165kb
Chr01.175kb
Chr01.185kb
Chr01.195kb
Chr01.205kb
Chr01.215kb
Chr01.225kb
Chr01.235kb
Chr01.245kb
Chr01.255kb
Chr01.265kb
Chr01.275kb
Chr01.285kb
Chr01

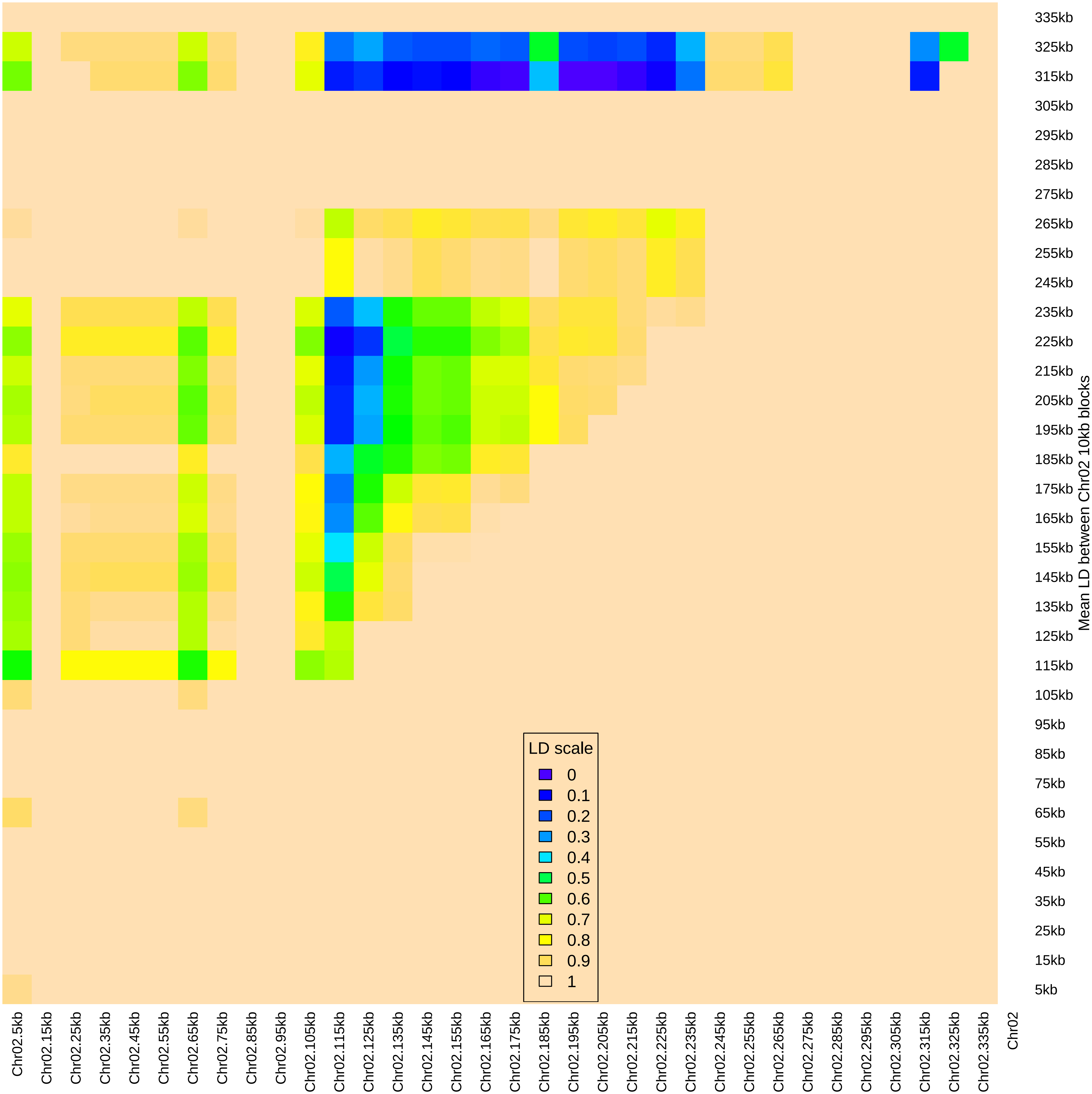

335kb
325kb
315kb
305kb
295kb
285kb
275kb
265kb
255kb
245kb
235kb
225kb
215kb
205kb
195kb
185kb
175kb
165kb
155kb
145kb
135kb
125kb
115kb
105kb
95kb
85kb
75kb
65kb
55kb
45kb
35kb
25kb
15kb
5kb
Mean LD between Chr02 10kb blocks
LD scale
0
0.1
0.2
0.3
0.4
0.5
0.6
0.7
0.8
0.9
1
Chr02.5kb
Chr02.15kb
Chr02.25kb
Chr02.35kb
Chr02.45kb
Chr02.55kb
Chr02.65kb
Chr02.75kb
Chr02.85kb
Chr02.95kb
Chr02.105kb
Chr02.115kb
Chr02.125kb
Chr02.135kb
Chr02.145kb
Chr02.155kb
Chr02.165kb
Chr02.175kb
Chr02.185kb
Chr02.195kb
Chr02.205kb
Chr02.215kb
Chr02.225kb
Chr02.235kb
Chr02.245kb
Chr02.255kb
Chr02.265kb
Chr02.275kb
Chr02.285kb
Chr02.295kb
Chr02.305kb
Chr02.315kb
Chr02.325kb
Chr02.335kb
Chr02

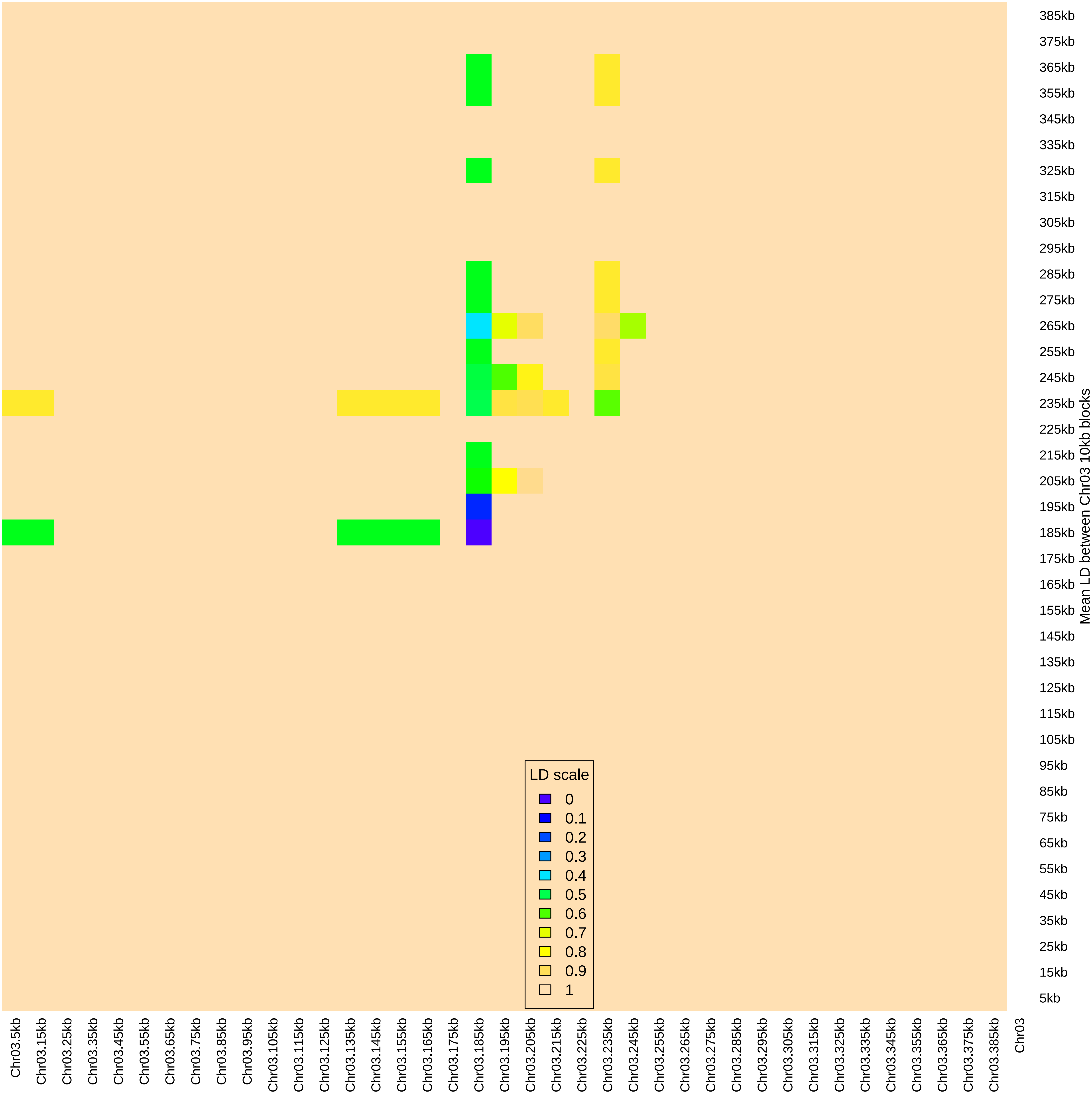
LD scale
0
0.1
0.2
0.3
0.4
0.5
0.6
0.7
0.8
0.9
1
Mean LD between Chr03 10kb blocks
385kb
375kb
365kb
355kb
345kb
335kb
325kb
315kb
305kb
295kb
285kb
275kb
265kb
255kb
245kb
235kb
225kb
215kb
205kb
195kb
185kb
175kb
165kb
155kb
145kb
135kb
125kb
115kb
105kb
95kb
85kb
75kb
65kb
55kb
45kb
35kb
25kb
15kb
5kb
Chr03.5kb
Chr03.15kb
Chr03.25kb
Chr03.35kb
Chr03.45kb
Chr03.55kb
Chr03.65kb
Chr03.75kb
Chr03.85kb
Chr03.95kb
Chr03.105kb
Chr03.115kb
Chr03.125kb
Chr03.135kb
Chr03.145kb
Chr03.155kb
Chr03.165kb
Chr03.175kb
Chr03.185kb
Chr03.195kb
Chr03.205kb
Chr03.215kb
Chr03.225kb
Chr03.235kb
Chr03.245kb
Chr03.255kb
Chr03.265kb
Chr03.275kb
Chr03.285kb
Chr03.295kb
Chr03.305kb
Chr03.315kb
Chr03.325kb
Chr03.335kb
Chr03.345kb
Chr03.355kb
Chr03.365kb
Chr03.375kb
Chr03.385kb
Chr03

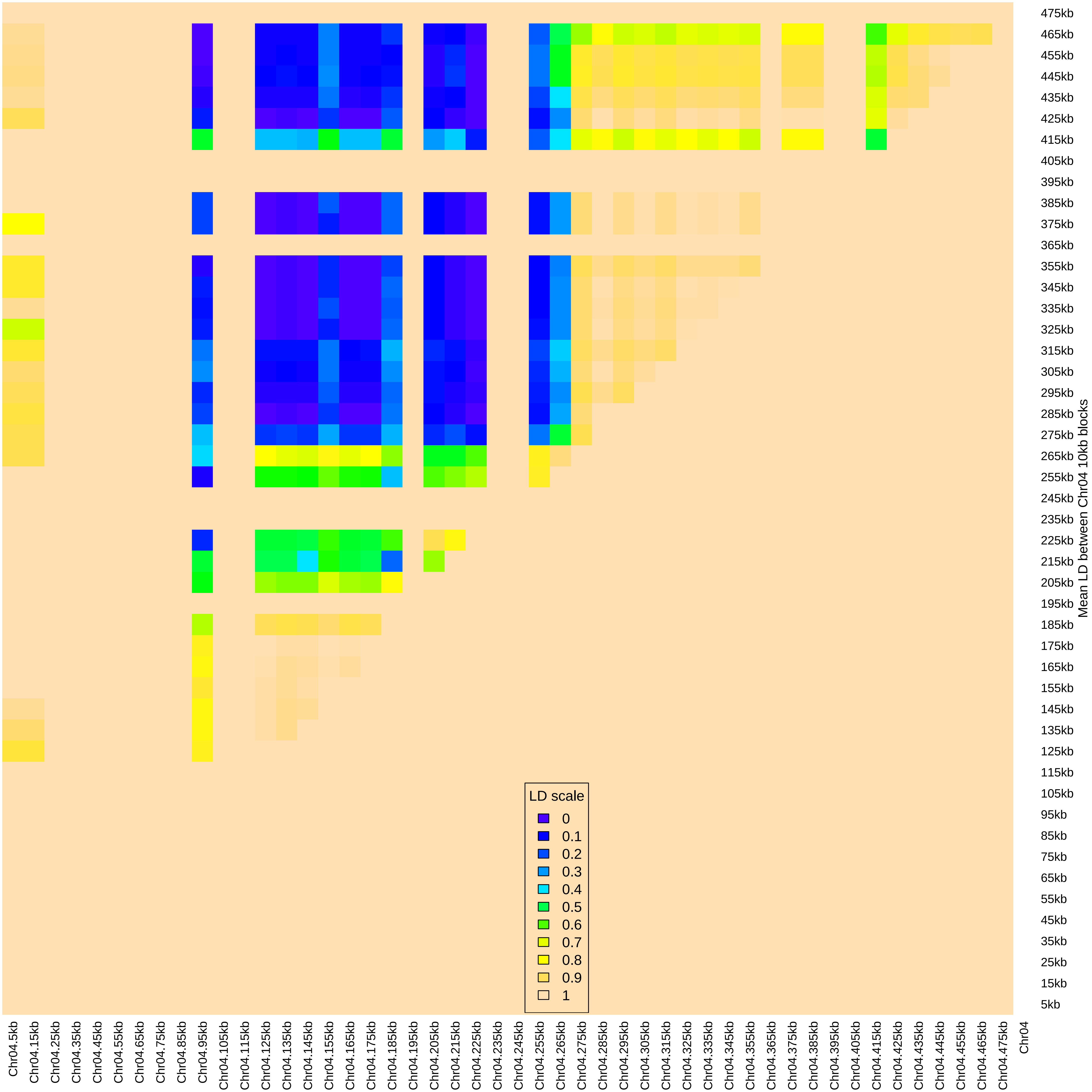
Mean LD between Chr04 10kb blocks
LD scale
0
0.1
0.2
0.3
0.4
0.5
0.6
0.7
0.8
0.9
1
475kb
465kb
455kb
445kb
435kb
425kb
415kb
405kb
395kb
385kb
375kb
365kb
355kb
345kb
335kb
325kb
315kb
305kb
295kb
285kb
275kb
265kb
255kb
245kb
235kb
225kb
215kb
205kb
195kb
185kb
175kb
165kb
155kb
145kb
135kb
125kb
115kb
105kb
95kb
85kb
75kb
65kb
55kb
45kb
35kb
25kb
15kb
5kb
Chr04.5kb
Chr04.15kb
Chr04.25kb
Chr04.35kb
Chr04.45kb
Chr04.55kb
Chr04.65kb
Chr04.75kb
Chr04.85kb
Chr04.95kb
Chr04.105kb
Chr04.115kb
Chr04.125kb
Chr04.135kb
Chr04.145kb
Chr04.155kb
Chr04.165kb
Chr04.175kb
Chr04.185kb
Chr04.195kb
Chr04.205kb
Chr04.215kb
Chr04.225kb
Chr04.235kb
Chr04.245kb
Chr04.255kb
Chr04.265kb
Chr04.275kb
Chr04.285kb
Chr04.295kb
Chr04.305kb
Chr04.315kb
Chr04.325kb
Chr04.335kb
Chr04.345kb
Chr04.355kb
Chr04.365kb
Chr04.375kb
Chr04.385kb
Chr04.395kb
Chr04.405kb
Chr04.415kb
Chr04.425kb
Chr04.435kb
Chr04.445kb
Chr04.455kb
Chr04.465kb
Chr04.475kb
Chr04

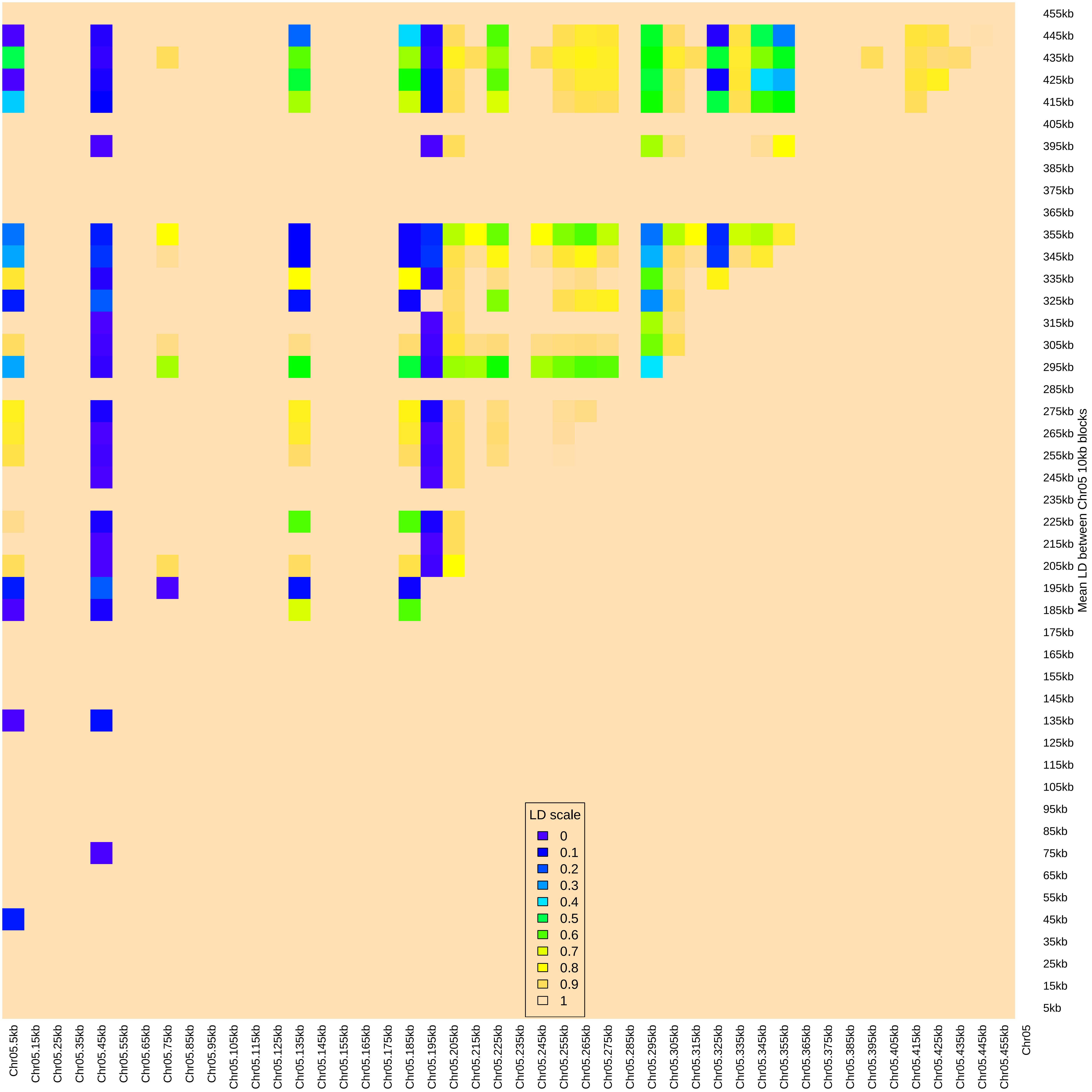
LD scale
0
0.1
0.2
0.3
0.4
0.5
0.6
0.7
0.8
0.9
1
Mean LD between Chr05 10kb blocks
Chr05

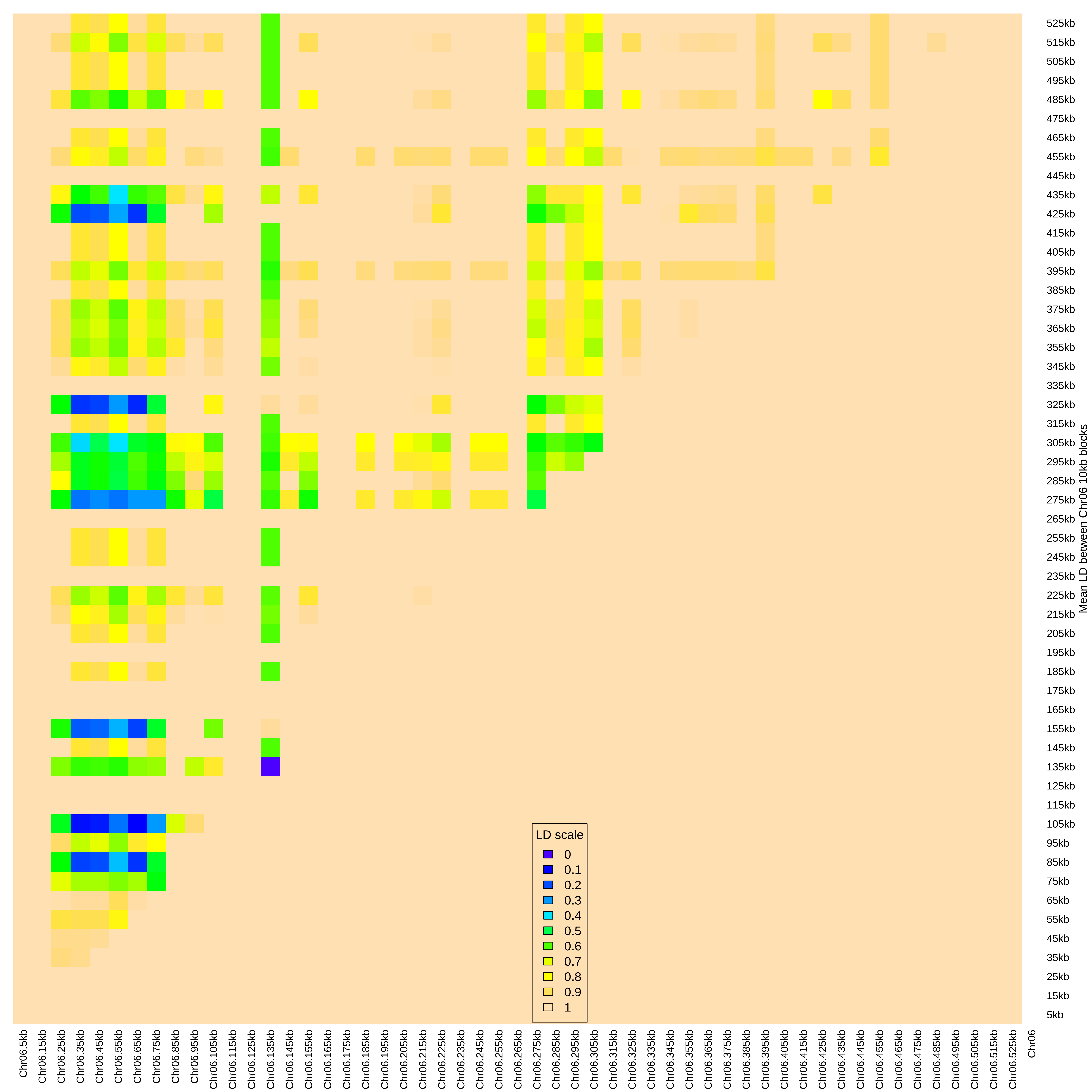

LD scale
0
0.1
0.2
0.3
0.4
0.5
0.6
0.7
0.8
0.9
1
Mean LD between Chr06 10kb blocks
525kb
515kb
505kb
495kb
485kb
475kb
465kb
455kb
445kb
435kb
425kb
415kb
405kb
395kb
385kb
375kb
365kb
355kb
345kb
335kb
325kb
315kb
305kb
295kb
285kb
275kb
265kb
255kb
245kb
235kb
225kb
215kb
205kb
195kb
185kb
175kb
165kb
155kb
145kb
135kb
125kb
115kb
105kb
95kb
85kb
75kb
65kb
55kb
45kb
35kb
25kb
15kb
5kb
Chr06.5kb
Chr06.15kb
Chr06.25kb
Chr06.35kb
Chr06.45kb
Chr06.55kb
Chr06.65kb
Chr06.75kb
Chr06.85kb
Chr06.95kb
Chr06.105kb
Chr06.115kb
Chr06.125kb
Chr06.135kb
Chr06.145kb
Chr06.155kb
Chr06.165kb
Chr06.175kb
Chr06.185kb
Chr06.195kb
Chr06.205kb
Chr06.215kb
Chr06.225kb
Chr06.235kb
Chr06.245kb
Chr06.255kb
Chr06.265kb
Chr06.275kb
Chr06.285kb
Chr06.295kb
Chr06.305kb
Chr06.315kb
Chr06.325kb
Chr06.335kb
Chr06.345kb
Chr06.355kb
Chr06.365kb
Chr06.375kb
Chr06.385kb
Chr06.395kb
Chr06.405kb
Chr06.415kb
Chr06.425kb
Chr06.435kb
Chr06.445kb
Chr06.455kb
Chr06.465kb
Chr06.475kb
Chr06.485kb
Chr06.495kb
Chr06.505kb
Chr06.515kb
Chr06.525kb
Chr06

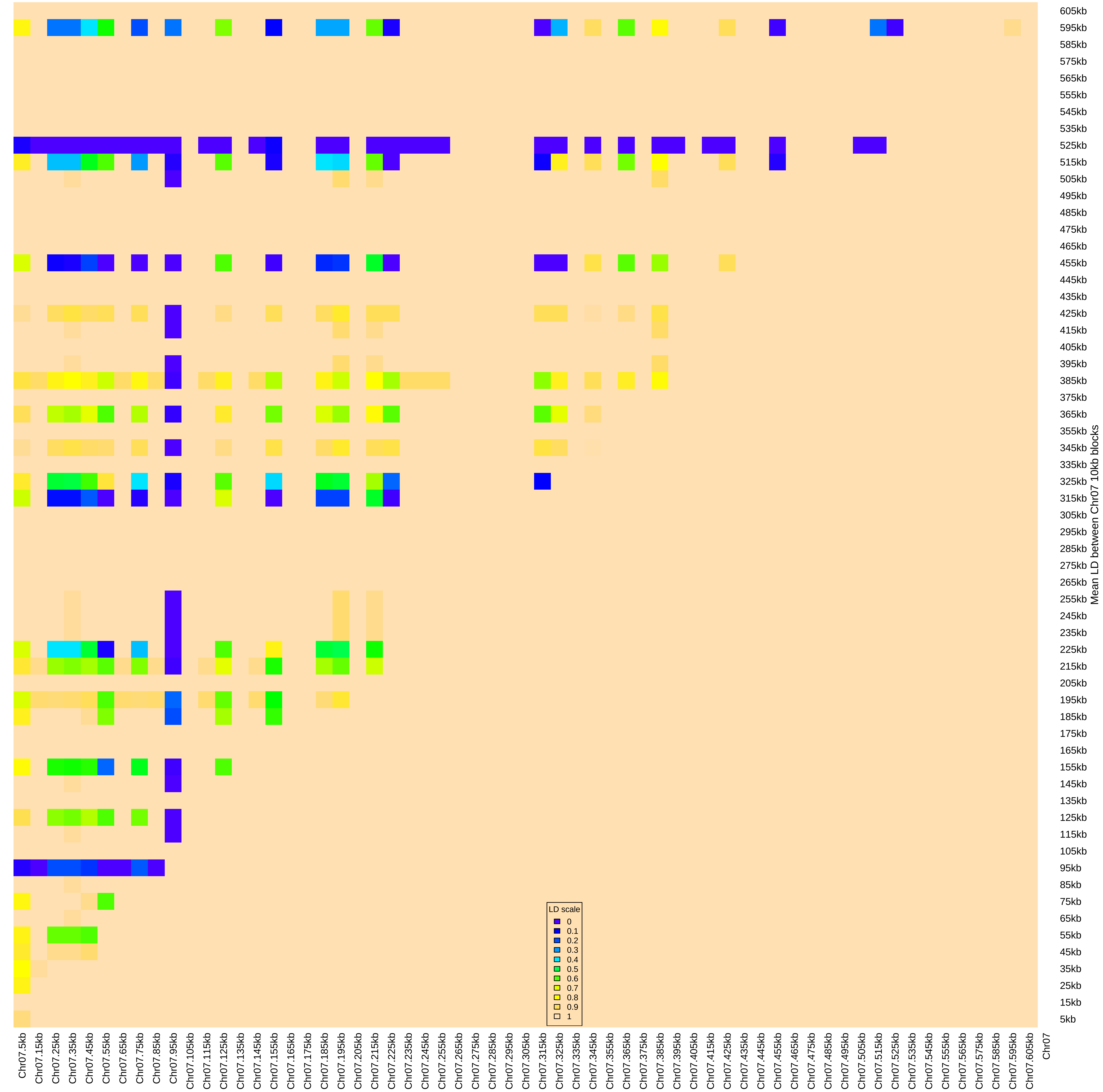

LD scale
0
0.1
0.2
0.3
0.4
0.5
0.6
0.7
0.8
0.9
1
Mean LD between Chr07 10kb blocks
Chr07

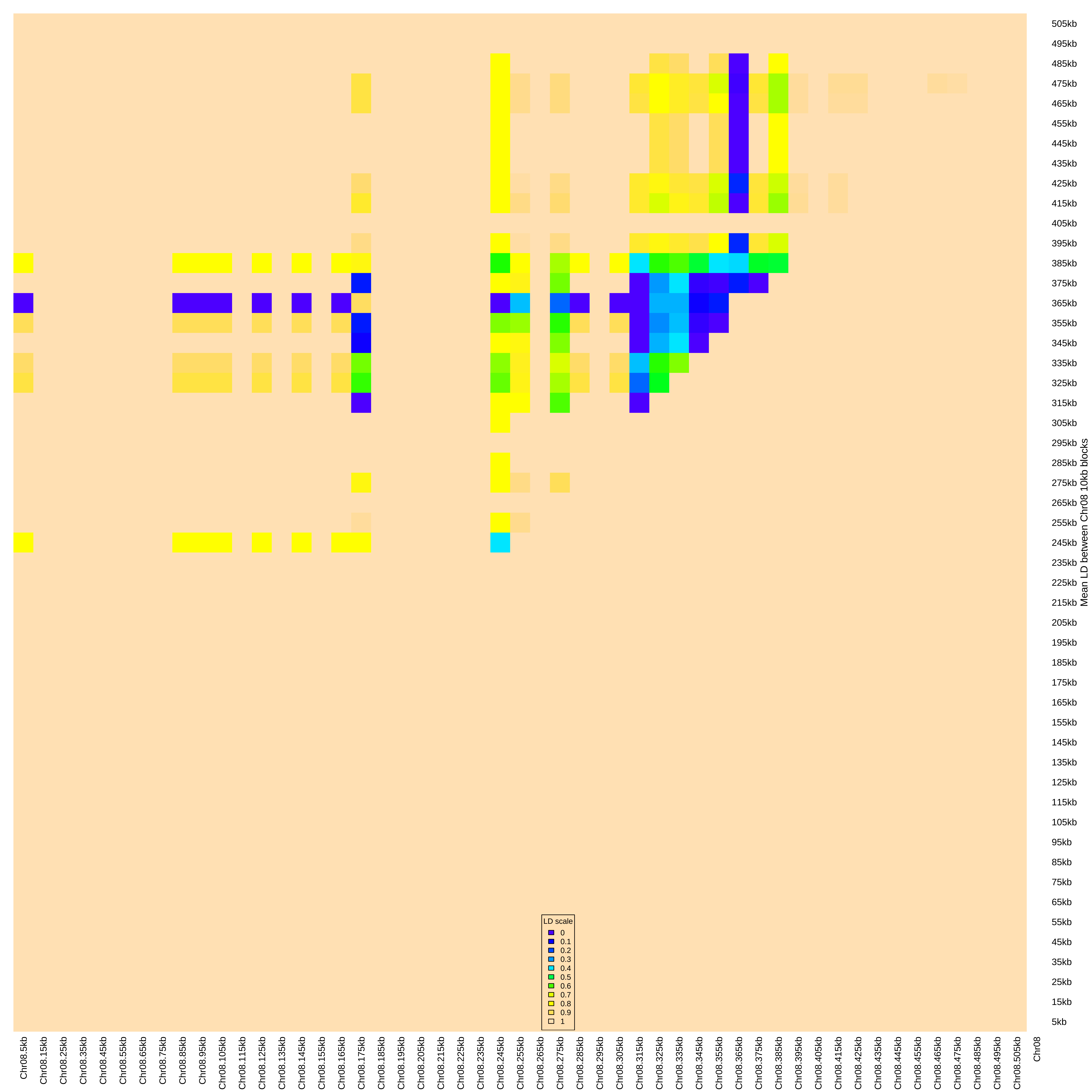

Mean LD between Chr08 10kb blocks
LD scale
0
0.1
0.2
0.3
0.4
0.5
0.6
0.7
0.8
0.9
1
505kb
495kb
485kb
475kb
465kb
455kb
445kb
435kb
425kb
415kb
405kb
395kb
385kb
375kb
365kb
355kb
345kb
335kb
325kb
315kb
305kb
295kb
285kb
275kb
265kb
255kb
245kb
235kb
225kb
215kb
205kb
195kb
185kb
175kb
165kb
155kb
145kb
135kb
125kb
115kb
105kb
95kb
85kb
75kb
65kb
55kb
45kb
35kb
25kb
15kb
5kb
Chr08.5kb
Chr08.15kb
Chr08.25kb
Chr08.35kb
Chr08.45kb
Chr08.55kb
Chr08.65kb
Chr08.75kb
Chr08.85kb
Chr08.95kb
Chr08.105kb
Chr08.115kb
Chr08.125kb
Chr08.135kb
Chr08.145kb
Chr08.155kb
Chr08.165kb
Chr08.175kb
Chr08.185kb
Chr08.195kb
Chr08.205kb
Chr08.215kb
Chr08.225kb
Chr08.235kb
Chr08.245kb
Chr08.255kb
Chr08.265kb
Chr08.275kb
Chr08.285kb
Chr08.295kb
Chr08.305kb
Chr08.315kb
Chr08.325kb
Chr08.335kb
Chr08.345kb
Chr08.355kb
Chr08.365kb
Chr08.375kb
Chr08.385kb
Chr08.395kb
Chr08.405kb
Chr08.415kb
Chr08.425kb
Chr08.435kb
Chr08.445kb
Chr08.455kb
Chr08.465kb
Chr08.475kb
Chr08.485kb
Chr08.495kb
Chr08.505kb
Chr08

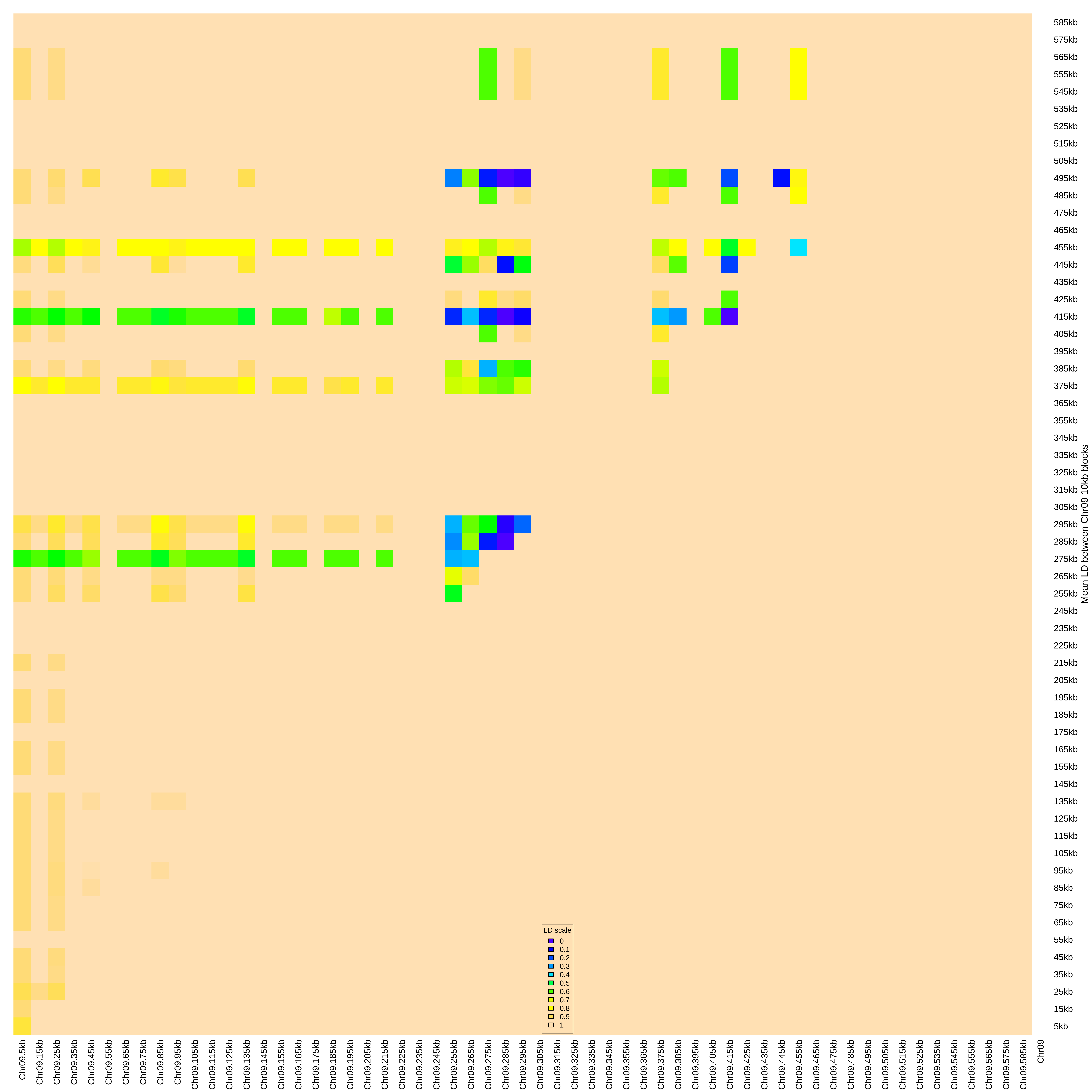

LD scale
0
0.1
0.2
0.3
0.4
0.5
0.6
0.7
0.8
0.9
1
Mean LD between Chr09 10kb blocks
Chr09

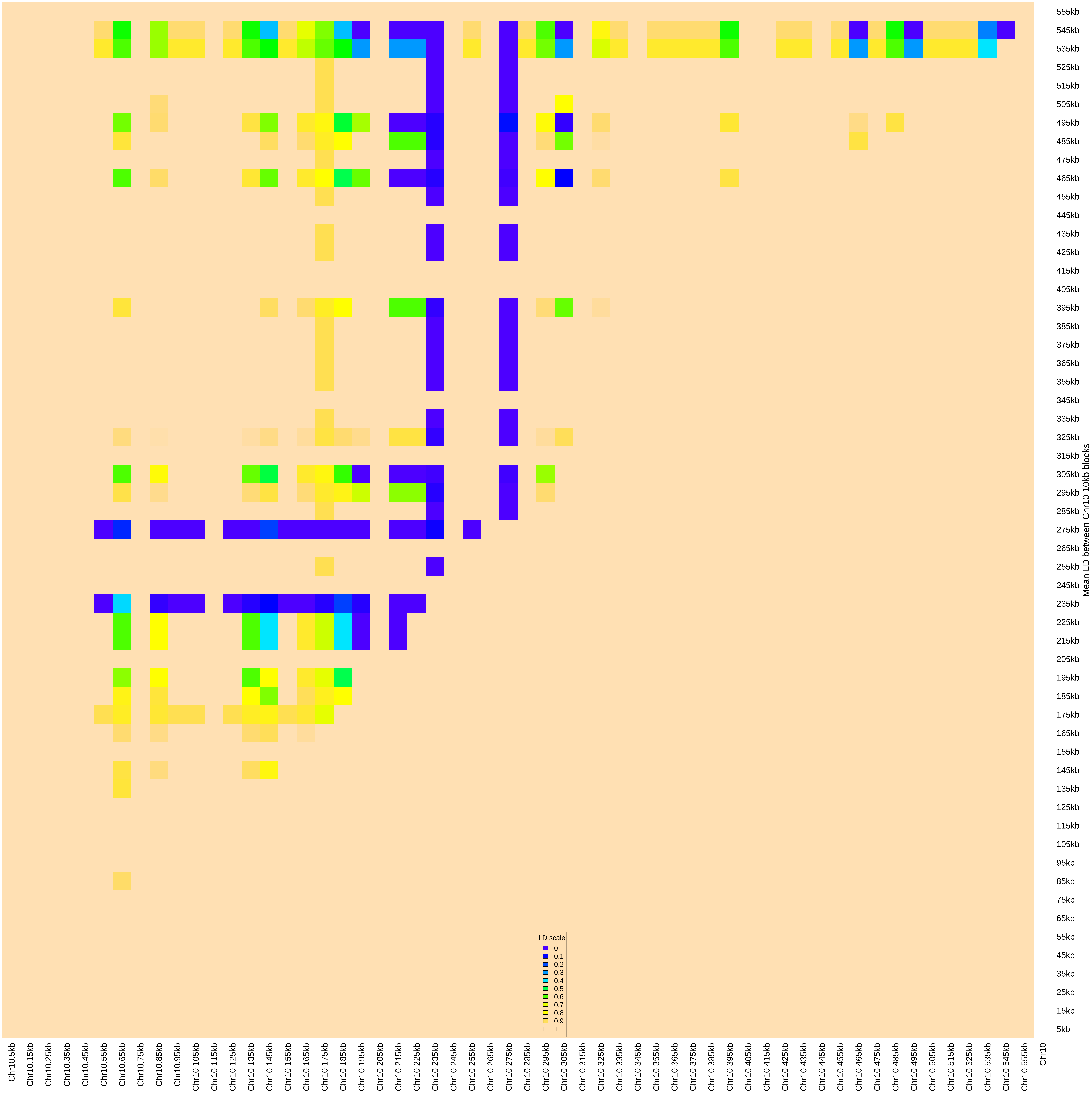
Mean LD between Chr10 10kb blocks
Chr10
LD scale
0
0.1
0.2
0.3
0.4
0.5
0.6
0.7
0.8
0.9
1
555kb 545kb 535kb 525kb 515kb 505kb 495kb 485kb 475kb 465kb 455kb 445kb 435kb 425kb 415kb 405kb 395kb 385kb 375kb 365kb 355kb 345kb 335kb 325kb 315kb 305kb 295kb 285kb 275kb 265kb 255kb 245kb 235kb 225kb 215kb 205kb 195kb 185kb 175kb 165kb 155kb 145kb 135kb 125kb 115kb 105kb 95kb 85kb 75kb 65kb 55kb 45kb 35kb 25kb 15kb 5kb
Chr10.5kb Chr10.15kb Chr10.25kb Chr10.35kb Chr10.45kb Chr10.55kb Chr10.65kb Chr10.75kb Chr10.85kb Chr10.95kb Chr10.105kb Chr10.115kb Chr10.125kb Chr10.135kb Chr10.145kb Chr10.155kb Chr10.165kb Chr10.175kb Chr10.185kb Chr10.195kb Chr10.205kb Chr10.215kb Chr10.225kb Chr10.235kb Chr10.245kb Chr10.255kb Chr10.265kb Chr10.275kb Chr10.285kb Chr10.295kb Chr10.305kb Chr10.315kb Chr10.325kb Chr10.335kb Chr10.345kb Chr10.355kb Chr10.365kb Chr10.375kb Chr10.385kb Chr10.395kb Chr10.405kb Chr10.415kb Chr10.425kb Chr10.435kb Chr10.445kb Chr10.455kb Chr10.465kb Chr10.475kb Chr10.485kb Chr10.495kb Chr10.505kb Chr10.515kb Chr10.525kb Chr10.535kb Chr10.545kb Chr10.555kb

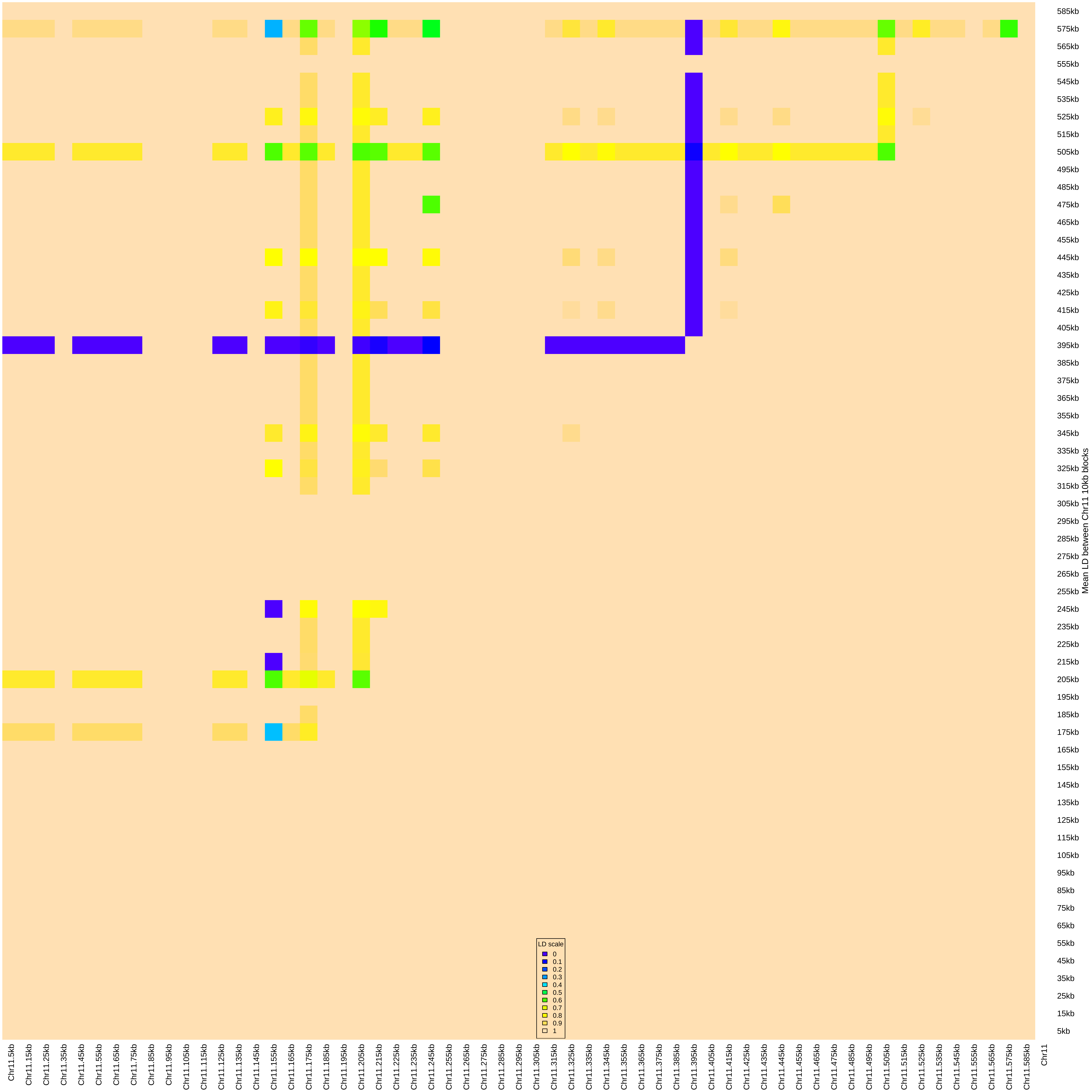
Mean LD between Chr11 10kb blocks
LD scale
0
0.1
0.2
0.3
0.4
0.5
0.6
0.7
0.8
0.9
1
585kb
575kb
565kb
555kb
545kb
535kb
525kb
515kb
505kb
495kb
485kb
475kb
465kb
455kb
445kb
435kb
425kb
415kb
405kb
395kb
385kb
375kb
365kb
355kb
345kb
335kb
325kb
315kb
305kb
295kb
285kb
275kb
265kb
255kb
245kb
235kb
225kb
215kb
205kb
195kb
185kb
175kb
165kb
155kb
145kb
135kb
125kb
115kb
105kb
95kb
85kb
75kb
65kb
55kb
45kb
35kb
25kb
15kb
5kb
Chr11.5kb
Chr11.15kb
Chr11.25kb
Chr11.35kb
Chr11.45kb
Chr11.55kb
Chr11.65kb
Chr11.75kb
Chr11.85kb
Chr11.95kb
Chr11.105kb
Chr11.115kb
Chr11.125kb
Chr11.135kb
Chr11.145kb
Chr11.155kb
Chr11.165kb
Chr11.175kb
Chr11.185kb
Chr11.195kb
Chr11.205kb
Chr11.215kb
Chr11.225kb
Chr11.235kb
Chr11.245kb
Chr11.255kb
Chr11.265kb
Chr11.275kb
Chr11.285kb
Chr11.295kb
Chr11.305kb
Chr11.315kb
Chr11.325kb
Chr11.335kb
Chr11.345kb
Chr11.355kb
Chr11.365kb
Chr11.375kb
Chr11.385kb
Chr11.395kb
Chr11.405kb
Chr11.415kb
Chr11.425kb
Chr11.435kb
Chr11.445kb
Chr11.455kb
Chr11.465kb
Chr11.475kb
Chr11.485kb
Chr11.495kb
Chr11.505kb
Chr11.515kb
Chr11.525kb
Chr11.535kb
Chr11.545kb
Chr11.555kb
Chr11.565kb
Chr11.575kb
Chr11.585kb
Chr11

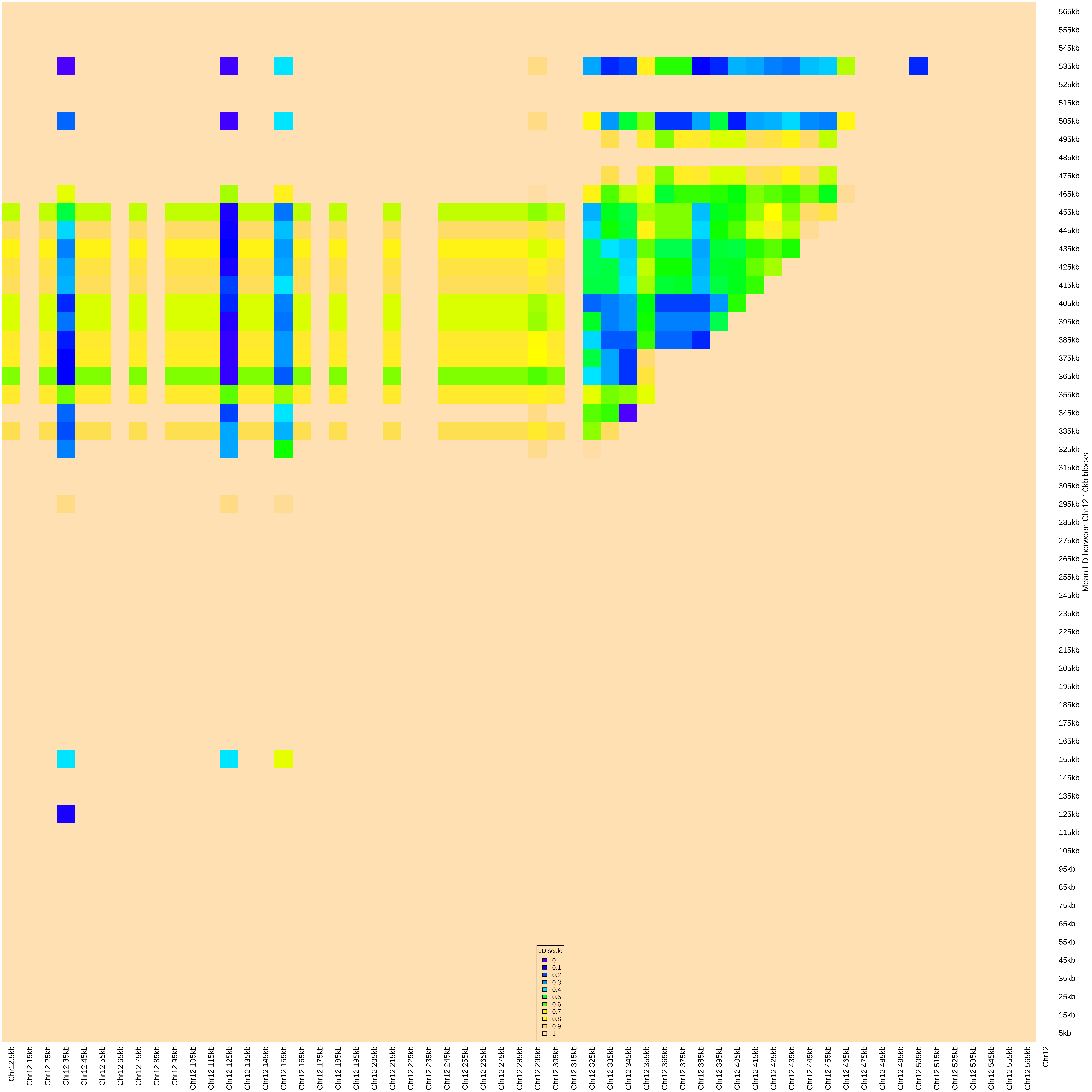
Mean LD between Chr12 10kb blocks
565kb
555kb
545kb
535kb
525kb
515kb
505kb
495kb
485kb
475kb
465kb
455kb
445kb
435kb
425kb
415kb
405kb
395kb
385kb
375kb
365kb
355kb
345kb
335kb
325kb
315kb
305kb
295kb
285kb
275kb
265kb
255kb
245kb
235kb
225kb
215kb
205kb
195kb
185kb
175kb
165kb
155kb
145kb
135kb
125kb
115kb
105kb
95kb
85kb
75kb
65kb
55kb
45kb
35kb
25kb
15kb
5kb
LD scale
0
0.1
0.2
0.3
0.4
0.5
0.6
0.7
0.8
0.9
1
Chr12.5kb
Chr12.15kb
Chr12.25kb
Chr12.35kb
Chr12.45kb
Chr12.55kb
Chr12.65kb
Chr12.75kb
Chr12.85kb
Chr12.95kb
Chr12.105kb
Chr12.115kb
Chr12.125kb
Chr12.135kb
Chr12.145kb
Chr12.155kb
Chr12.165kb
Chr12.175kb
Chr12.185kb
Chr12.195kb
Chr12.205kb
Chr12.215kb
Chr12.225kb
Chr12.235kb
Chr12.245kb
Chr12.255kb
Chr12.265kb
Chr12.275kb
Chr12.285kb
Chr12.295kb
Chr12.305kb
Chr12.315kb
Chr12.325kb
Chr12.335kb
Chr12.345kb
Chr12.355kb
Chr12.365kb
Chr12.375kb
Chr12.385kb
Chr12.395kb
Chr12.405kb
Chr12.415kb
Chr12.425kb
Chr12.435kb
Chr12.445kb
Chr12.455kb
Chr12.465kb
Chr12.475kb
Chr12.485kb
Chr12.495kb
Chr12.505kb
Chr12.515kb
Chr12.525kb
Chr12.535kb
Chr12.545kb
Chr12.555kb
Chr12.565kb
Chr12

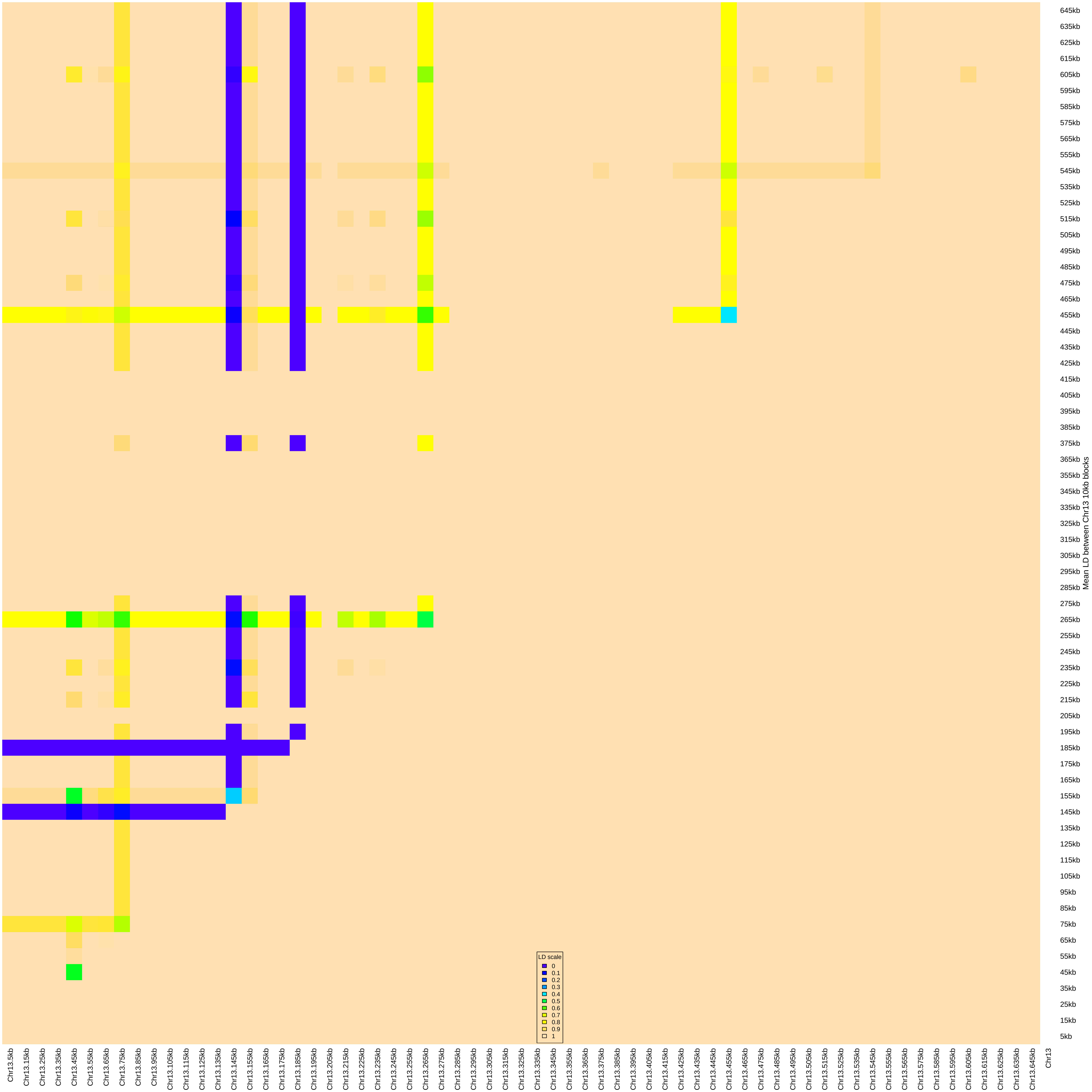
Mean LD between Chr13 10kb blocks
LD scale
0
0.1
0.2
0.3
0.4
0.5
0.6
0.7
0.8
0.9
1
645kb
635kb
625kb
615kb
605kb
595kb
585kb
575kb
565kb
555kb
545kb
535kb
525kb
515kb
505kb
495kb
485kb
475kb
465kb
455kb
445kb
435kb
425kb
415kb
405kb
395kb
385kb
375kb
365kb
355kb
345kb
335kb
325kb
315kb
305kb
295kb
285kb
275kb
265kb
255kb
245kb
235kb
225kb
215kb
205kb
195kb
185kb
175kb
165kb
155kb
145kb
135kb
125kb
115kb
105kb
95kb
85kb
75kb
65kb
55kb
45kb
35kb
25kb
15kb
5kb
Chr13.5kb
Chr13.15kb
Chr13.25kb
Chr13.35kb
Chr13.45kb
Chr13.55kb
Chr13.65kb
Chr13.75kb
Chr13.85kb
Chr13.95kb
Chr13.105kb
Chr13.115kb
Chr13.125kb
Chr13.135kb
Chr13.145kb
Chr13.155kb
Chr13.165kb
Chr13.175kb
Chr13.185kb
Chr13.195kb
Chr13.205kb
Chr13.215kb
Chr13.225kb
Chr13.235kb
Chr13.245kb
Chr13.255kb
Chr13.265kb
Chr13.275kb
Chr13.285kb
Chr13.295kb
Chr13.305kb
Chr13.315kb
Chr13.325kb
Chr13.335kb
Chr13.345kb
Chr13.355kb
Chr13.365kb
Chr13.375kb
Chr13.385kb
Chr13.395kb
Chr13.405kb
Chr13.415kb
Chr13.425kb
Chr13.435kb
Chr13.445kb
Chr13.455kb
Chr13.465kb
Chr13.475kb
Chr13.485kb
Chr13.495kb
Chr13.505kb
Chr13.515kb
Chr13.525kb
Chr13.535kb
Chr13.545kb
Chr13.555kb
Chr13.565kb
Chr13.575kb
Chr13.585kb
Chr13.595kb
Chr13.605kb
Chr13.615kb
Chr13.625kb
Chr13.635kb
Chr13.645kb
Chr13

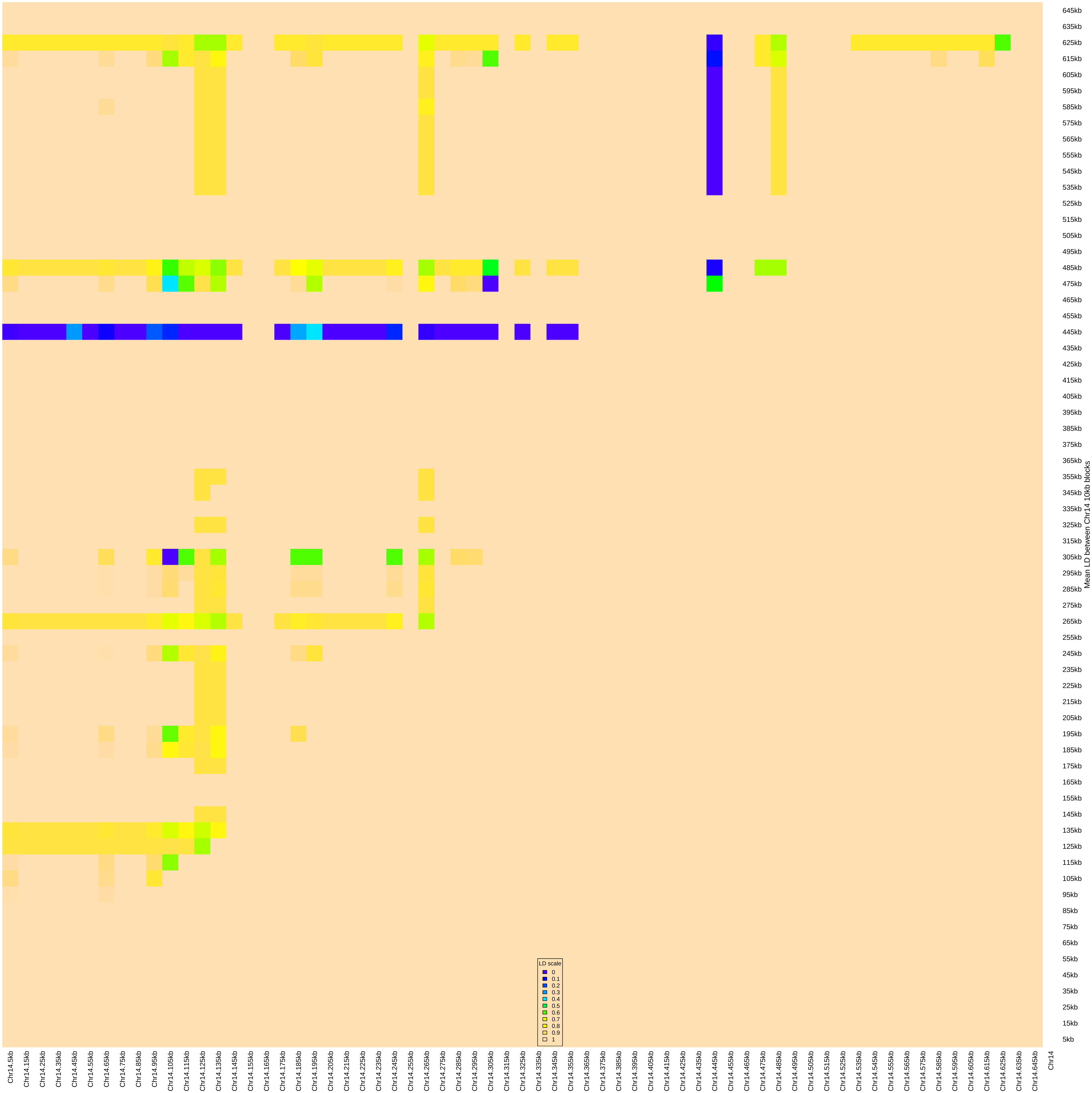
LD scale
0
0.1
0.2
0.3
0.4
0.5
0.6
0.7
0.8
0.9
1
Mean LD between Chr14 10kb blocks
Chr14

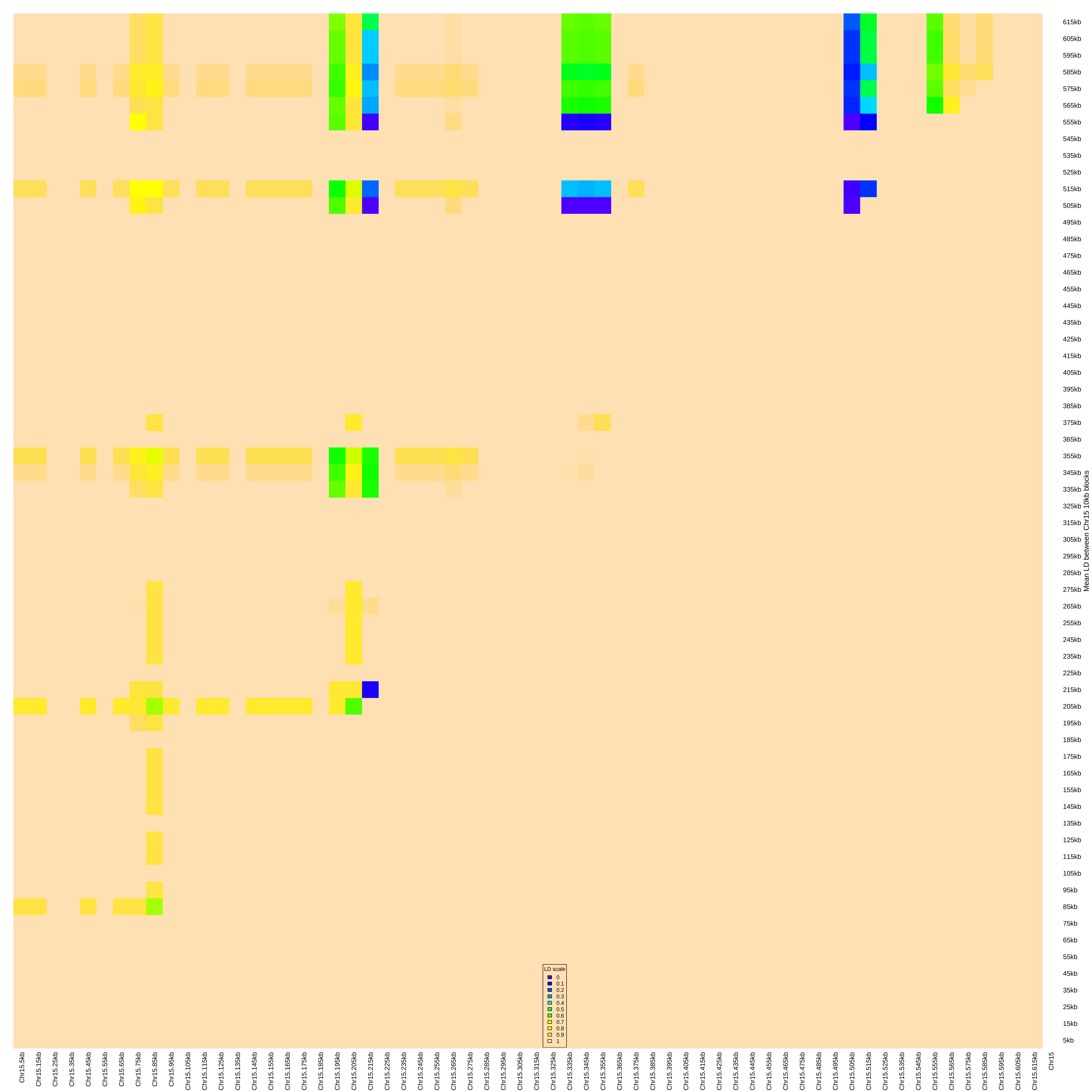
Mean LD between Chr15 10kb blocks
LD scale
0
0.1
0.2
0.3
0.4
0.5
0.6
0.7
0.8
0.9
1
615kb
605kb
595kb
585kb
575kb
565kb
555kb
545kb
535kb
525kb
515kb
505kb
495kb
485kb
475kb
465kb
455kb
445kb
435kb
425kb
415kb
405kb
395kb
385kb
375kb
365kb
355kb
345kb
335kb
325kb
315kb
305kb
295kb
285kb
275kb
265kb
255kb
245kb
235kb
225kb
215kb
205kb
195kb
185kb
175kb
165kb
155kb
145kb
135kb
125kb
115kb
105kb
95kb
85kb
75kb
65kb
55kb
45kb
35kb
25kb
15kb
5kb
Chr15.5kb
Chr15.15kb
Chr15.25kb
Chr15.35kb
Chr15.45kb
Chr15.55kb
Chr15.65kb
Chr15.75kb
Chr15.85kb
Chr15.95kb
Chr15.105kb
Chr15.115kb
Chr15.125kb
Chr15.135kb
Chr15.145kb
Chr15.155kb
Chr15.165kb
Chr15.175kb
Chr15.185kb
Chr15.195kb
Chr15.205kb
Chr15.215kb
Chr15.225kb
Chr15.235kb
Chr15.245kb
Chr15.255kb
Chr15.265kb
Chr15.275kb
Chr15.285kb
Chr15.295kb
Chr15.305kb
Chr15.315kb
Chr15.325kb
Chr15.335kb
Chr15.345kb
Chr15.355kb
Chr15.365kb
Chr15.375kb
Chr15.385kb
Chr15.395kb
Chr15.405kb
Chr15.415kb
Chr15.425kb
Chr15.435kb
Chr15.445kb
Chr15.455kb
Chr15.465kb
Chr15.475kb
Chr15.485kb
Chr15.495kb
Chr15.505kb
Chr15.515kb
Chr15.525kb
Chr15.535kb
Chr15.545kb
Chr15.555kb
Chr15.565kb
Chr15.575kb
Chr15.585kb
Chr15.595kb
Chr15.605kb
Chr15.615kb
Chr15

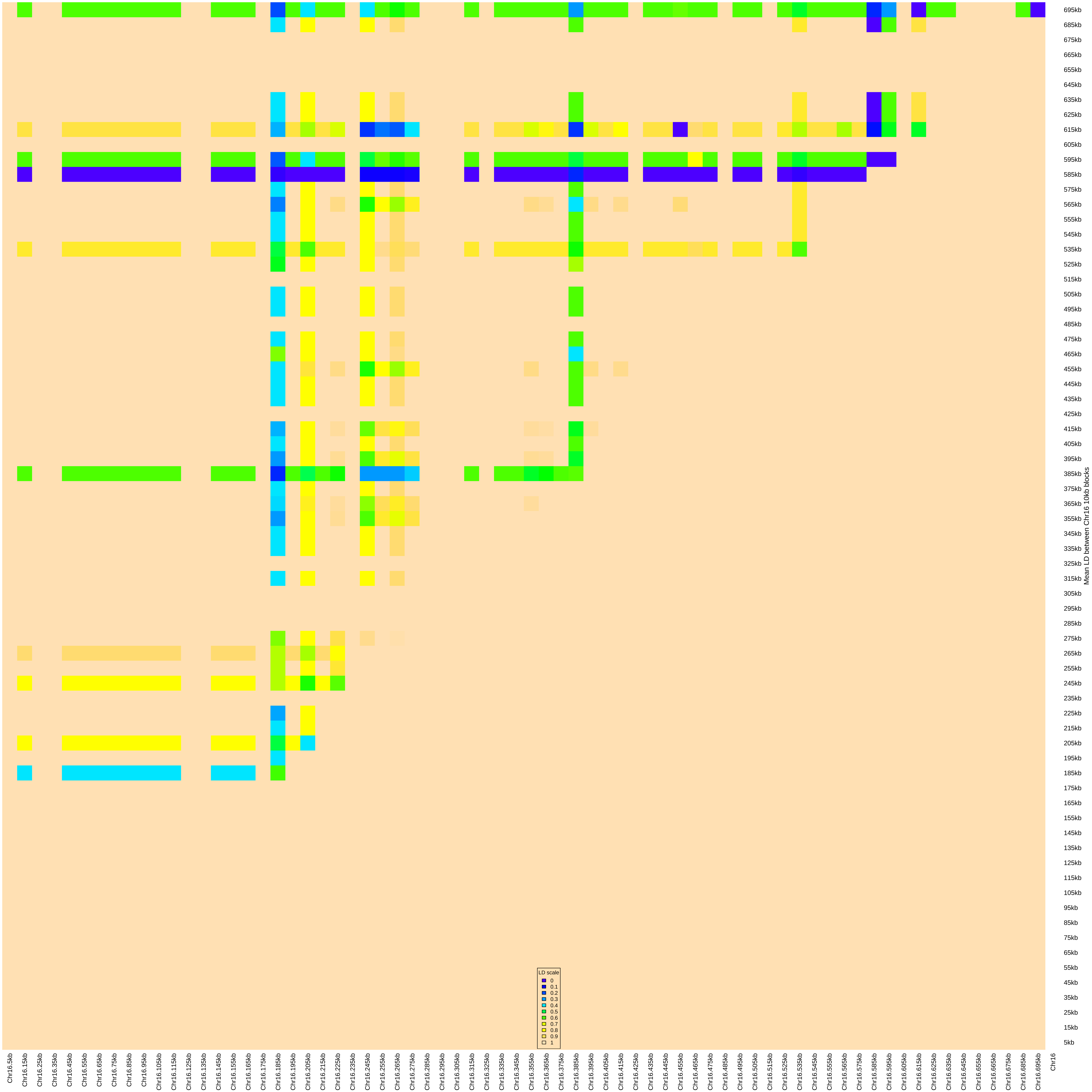
Mean LD between Chr16 10kb blocks
695kb
685kb
675kb
665kb
655kb
645kb
635kb
625kb
615kb
605kb
595kb
585kb
575kb
565kb
555kb
545kb
535kb
525kb
515kb
505kb
495kb
485kb
475kb
465kb
455kb
445kb
435kb
425kb
415kb
405kb
395kb
385kb
375kb
365kb
355kb
345kb
335kb
325kb
315kb
305kb
295kb
285kb
275kb
265kb
255kb
245kb
235kb
225kb
215kb
205kb
195kb
185kb
175kb
165kb
155kb
145kb
135kb
125kb
115kb
105kb
95kb
85kb
75kb
65kb
55kb
45kb
35kb
25kb
15kb
5kb
Chr16.5kb
Chr16.15kb
Chr16.25kb
Chr16.35kb
Chr16.45kb
Chr16.55kb
Chr16.65kb
Chr16.75kb
Chr16.85kb
Chr16.95kb
Chr16.105kb
Chr16.115kb
Chr16.125kb
Chr16.135kb
Chr16.145kb
Chr16.155kb
Chr16.165kb
Chr16.175kb
Chr16.185kb
Chr16.195kb
Chr16.205kb
Chr16.215kb
Chr16.225kb
Chr16.235kb
Chr16.245kb
Chr16.255kb
Chr16.265kb
Chr16.275kb
Chr16.285kb
Chr16.295kb
Chr16.305kb
Chr16.315kb
Chr16.325kb
Chr16.335kb
Chr16.345kb
Chr16.355kb
Chr16.365kb
Chr16.375kb
Chr16.385kb
Chr16.395kb
Chr16.405kb
Chr16.415kb
Chr16.425kb
Chr16.435kb
Chr16.445kb
Chr16.455kb
Chr16.465kb
Chr16.475kb
Chr16.485kb
Chr16.495kb
Chr16.505kb
Chr16.515kb
Chr16.525kb
Chr16.535kb
Chr16.545kb
Chr16.555kb
Chr16.565kb
Chr16.575kb
Chr16.585kb
Chr16.595kb
Chr16.605kb
Chr16.615kb
Chr16.625kb
Chr16.635kb
Chr16.645kb
Chr16.655kb
Chr16.665kb
Chr16.675kb
Chr16.685kb
Chr16.695kb
Chr16
LD scale
0
0.1
0.2
0.3
0.4
0.5
0.6
0.7
0.8
0.9
1

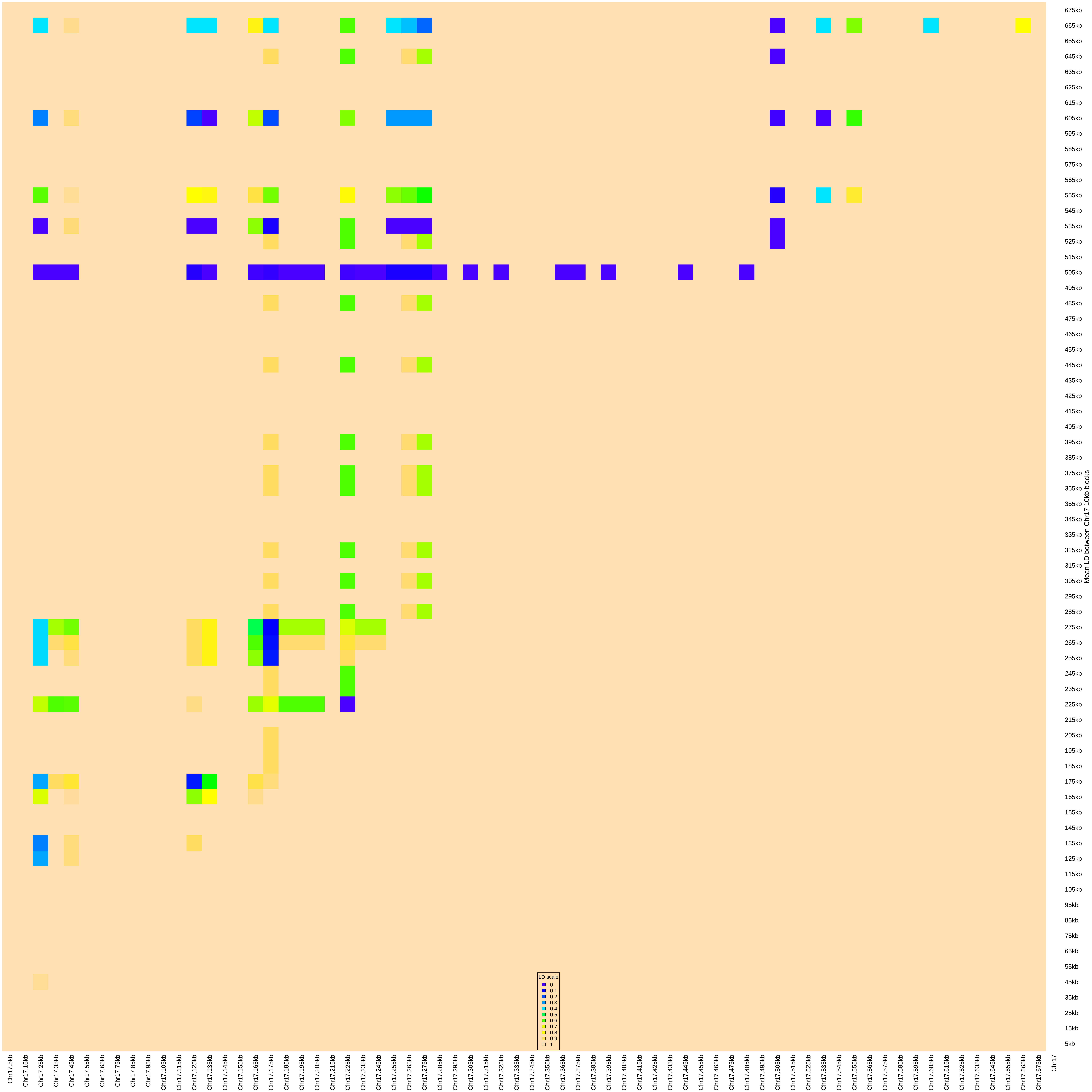

Mean LD between Chr17 10kb blocks
LD scale
0
0.1
0.2
0.3
0.4
0.5
0.6
0.7
0.8
0.9
1
675kb
665kb
655kb
645kb
635kb
625kb
615kb
605kb
595kb
585kb
575kb
565kb
555kb
545kb
535kb
525kb
515kb
505kb
495kb
485kb
475kb
465kb
455kb
445kb
435kb
425kb
415kb
405kb
395kb
385kb
375kb
365kb
355kb
345kb
335kb
325kb
315kb
305kb
295kb
285kb
275kb
265kb
255kb
245kb
235kb
225kb
215kb
205kb
195kb
185kb
175kb
165kb
155kb
145kb
135kb
125kb
115kb
105kb
95kb
85kb
75kb
65kb
55kb
45kb
35kb
25kb
15kb
5kb
Chr17.5kb
Chr17.15kb
Chr17.25kb
Chr17.35kb
Chr17.45kb
Chr17.55kb
Chr17.65kb
Chr17.75kb
Chr17.85kb
Chr17.95kb
Chr17.105kb
Chr17.115kb
Chr17.125kb
Chr17.135kb
Chr17.145kb
Chr17.155kb
Chr17.165kb
Chr17.175kb
Chr17.185kb
Chr17.195kb
Chr17.205kb
Chr17.215kb
Chr17.225kb
Chr17.235kb
Chr17.245kb
Chr17.255kb
Chr17.265kb
Chr17.275kb
Chr17.285kb
Chr17.295kb
Chr17.305kb
Chr17.315kb
Chr17.325kb
Chr17.335kb
Chr17.345kb
Chr17.355kb
Chr17.365kb
Chr17.375kb
Chr17.385kb
Chr17.395kb
Chr17.405kb
Chr17.415kb
Chr17.425kb
Chr17.435kb
Chr17.445kb
Chr17.455kb
Chr17.465kb
Chr17.475kb
Chr17.485kb
Chr17.495kb
Chr17.505kb
Chr17.515kb
Chr17.525kb
Chr17.535kb
Chr17.545kb
Chr17.555kb
Chr17.565kb
Chr17.575kb
Chr17.585kb
Chr17.595kb
Chr17.605kb
Chr17.615kb
Chr17.625kb
Chr17.635kb
Chr17.645kb
Chr17.655kb
Chr17.665kb
Chr17.675kb
Chr17

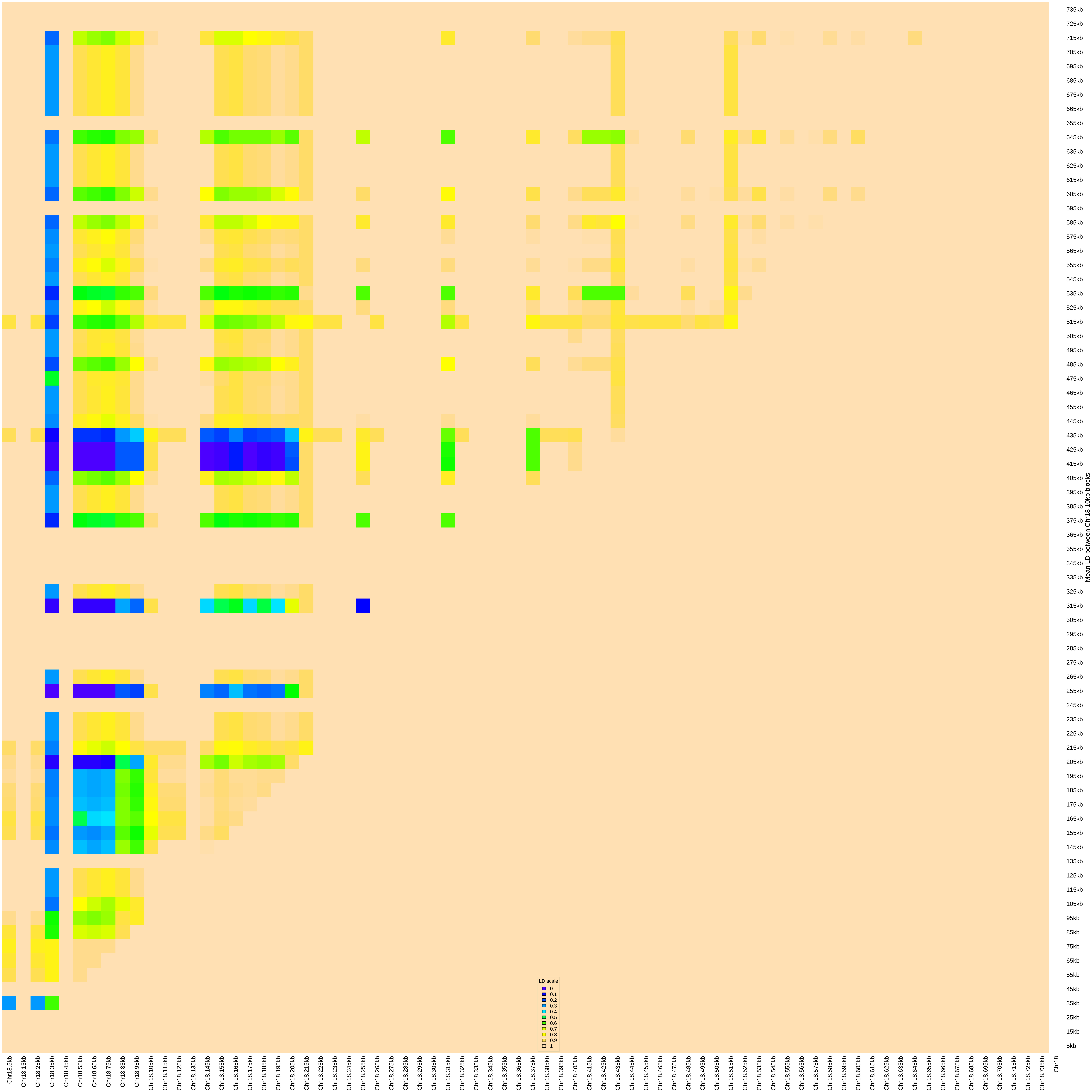
Mean LD between Chr18 10kb blocks
LD scale
0
0.1
0.2
0.3
0.4
0.5
0.6
0.7
0.8
0.9
1
735kb
725kb
715kb
705kb
695kb
685kb
675kb
665kb
655kb
645kb
635kb
625kb
615kb
605kb
595kb
585kb
575kb
565kb
555kb
545kb
535kb
525kb
515kb
505kb
495kb
485kb
475kb
465kb
455kb
445kb
435kb
425kb
415kb
405kb
395kb
385kb
375kb
365kb
355kb
345kb
335kb
325kb
315kb
305kb
295kb
285kb
275kb
265kb
255kb
245kb
235kb
225kb
215kb
205kb
195kb
185kb
175kb
165kb
155kb
145kb
135kb
125kb
115kb
105kb
95kb
85kb
75kb
65kb
55kb
45kb
35kb
25kb
15kb
5kb
Chr18.5kb
Chr18.15kb
Chr18.25kb
Chr18.35kb
Chr18.45kb
Chr18.55kb
Chr18.65kb
Chr18.75kb
Chr18.85kb
Chr18.95kb
Chr18.105kb
Chr18.115kb
Chr18.125kb
Chr18.135kb
Chr18.145kb
Chr18.155kb
Chr18.165kb
Chr18.175kb
Chr18.185kb
Chr18.195kb
Chr18.205kb
Chr18.215kb
Chr18.225kb
Chr18.235kb
Chr18.245kb
Chr18.255kb
Chr18.265kb
Chr18.275kb
Chr18.285kb
Chr18.295kb
Chr18.305kb
Chr18.315kb
Chr18.325kb
Chr18.335kb
Chr18.345kb
Chr18.355kb
Chr18.365kb
Chr18.375kb
Chr18.385kb
Chr18.395kb
Chr18.405kb
Chr18.415kb
Chr18.425kb
Chr18.435kb
Chr18.445kb
Chr18.455kb
Chr18.465kb
Chr18.475kb
Chr18.485kb
Chr18.495kb
Chr18.505kb
Chr18.515kb
Chr18.525kb
Chr18.535kb
Chr18.545kb
Chr18.555kb
Chr18.565kb
Chr18.575kb
Chr18.585kb
Chr18.595kb
Chr18.605kb
Chr18.615kb
Chr18.625kb
Chr18.635kb
Chr18.645kb
Chr18.655kb
Chr18.665kb
Chr18.675kb
Chr18.685kb
Chr18.695kb
Chr18.705kb
Chr18.715kb
Chr18.725kb
Chr18.735kb
Chr18

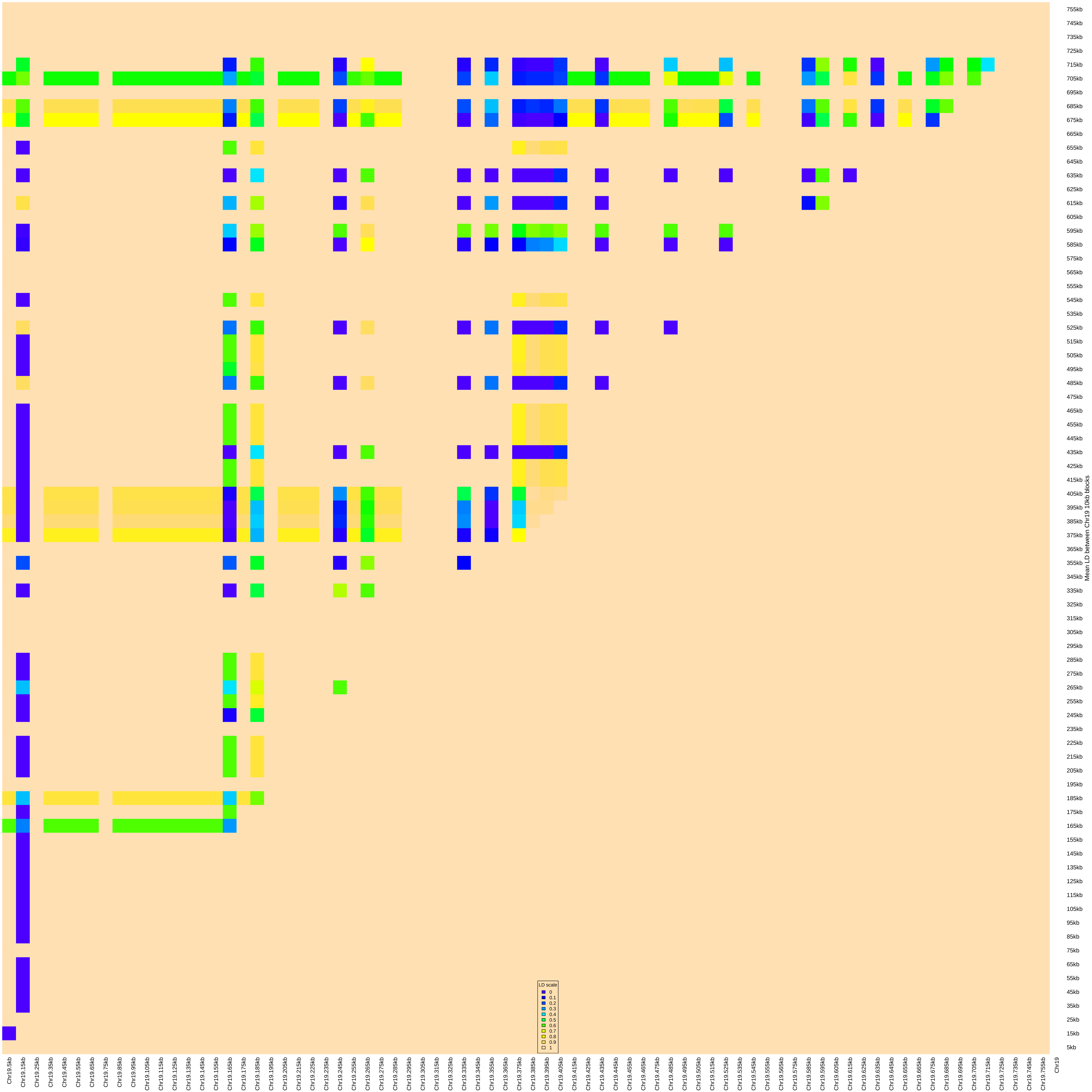

Mean LD between Chr19 10kb blocks
LD scale
0
0.1
0.2
0.3
0.4
0.5
0.6
0.7
0.8
0.9
1
755kb
745kb
735kb
725kb
715kb
705kb
695kb
685kb
675kb
665kb
655kb
645kb
635kb
625kb
615kb
605kb
595kb
585kb
575kb
565kb
555kb
545kb
535kb
525kb
515kb
505kb
495kb
485kb
475kb
465kb
455kb
445kb
435kb
425kb
415kb
405kb
395kb
385kb
375kb
365kb
355kb
345kb
335kb
325kb
315kb
305kb
295kb
285kb
275kb
265kb
255kb
245kb
235kb
225kb
215kb
205kb
195kb
185kb
175kb
165kb
155kb
145kb
135kb
125kb
115kb
105kb
95kb
85kb
75kb
65kb
55kb
45kb
35kb
25kb
15kb
5kb
Chr19.5kb
Chr19.15kb
Chr19.25kb
Chr19.35kb
Chr19.45kb
Chr19.55kb
Chr19.65kb
Chr19.75kb
Chr19.85kb
Chr19.95kb
Chr19.105kb
Chr19.115kb
Chr19.125kb
Chr19.135kb
Chr19.145kb
Chr19.155kb
Chr19.165kb
Chr19.175kb
Chr19.185kb
Chr19.195kb
Chr19.205kb
Chr19.215kb
Chr19.225kb
Chr19.235kb
Chr19.245kb
Chr19.255kb
Chr19.265kb
Chr19.275kb
Chr19.285kb
Chr19.295kb
Chr19.305kb
Chr19.315kb
Chr19.325kb
Chr19.335kb
Chr19.345kb
Chr19.355kb
Chr19.365kb
Chr19.375kb
Chr19.385kb
Chr19.395kb
Chr19.405kb
Chr19.415kb
Chr19.425kb
Chr19.435kb
Chr19.445kb
Chr19.455kb
Chr19.465kb
Chr19.475kb
Chr19.485kb
Chr19.495kb
Chr19.505kb
Chr19.515kb
Chr19.525kb
Chr19.535kb
Chr19.545kb
Chr19.555kb
Chr19.565kb
Chr19.575kb
Chr19.585kb
Chr19.595kb
Chr19.605kb
Chr19.615kb
Chr19.625kb
Chr19.635kb
Chr19.645kb
Chr19.655kb
Chr19.665kb
Chr19.675kb
Chr19.685kb
Chr19.695kb
Chr19.705kb
Chr19.715kb
Chr19.725kb
Chr19.735kb
Chr19.745kb
Chr19.755kb
Chr19

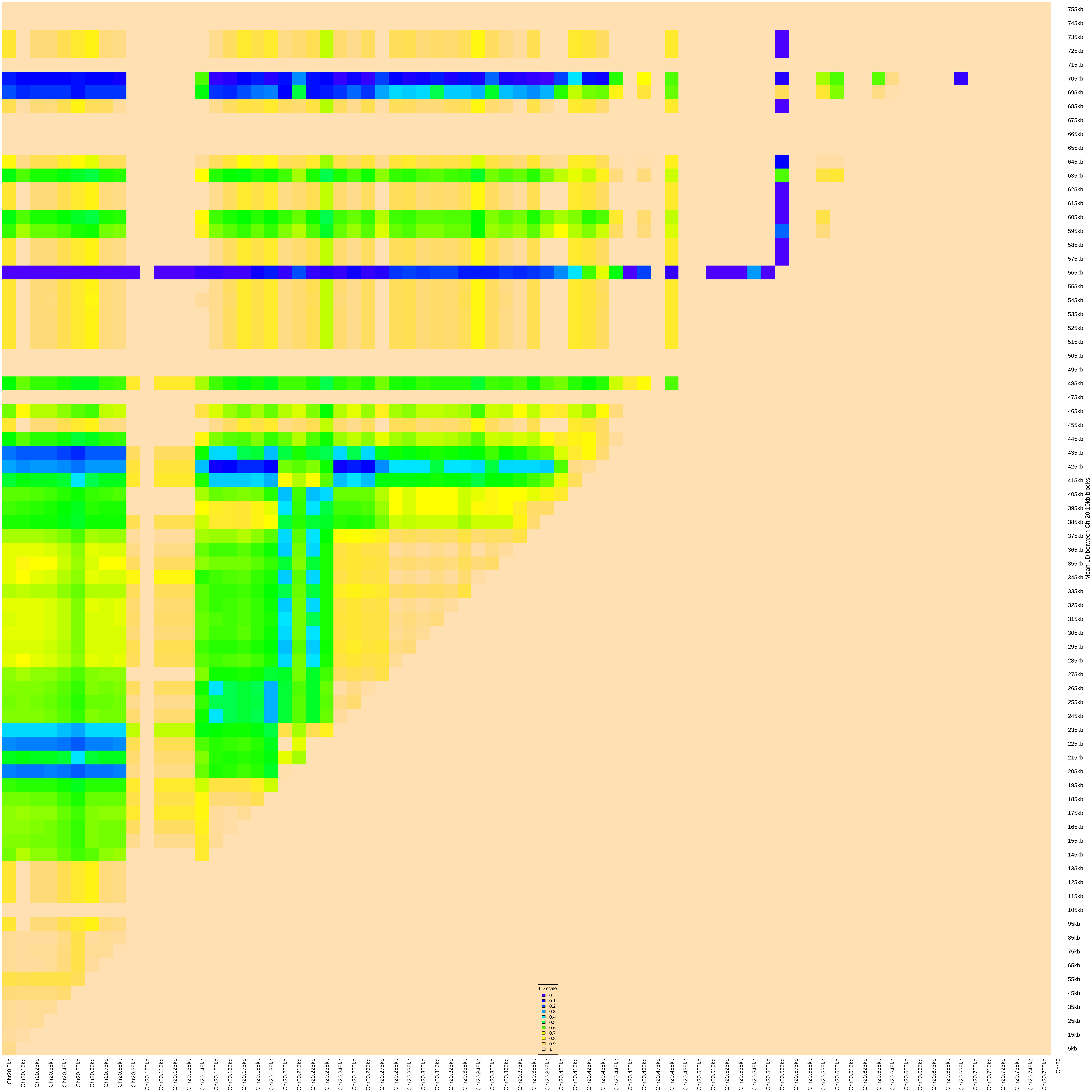

755kb
745kb
735kb
725kb
715kb
705kb
695kb
685kb
675kb
665kb
655kb
645kb
635kb
625kb
615kb
605kb
595kb
585kb
575kb
565kb
555kb
545kb
535kb
525kb
515kb
505kb
495kb
485kb
475kb
465kb
455kb
445kb
435kb
425kb
415kb
405kb
395kb
385kb
375kb
365kb
355kb
345kb
335kb
325kb
315kb
305kb
295kb
285kb
275kb
265kb
255kb
245kb
235kb
225kb
215kb
205kb
195kb
185kb
175kb
165kb
155kb
145kb
135kb
125kb
115kb
105kb
95kb
85kb
75kb
65kb
55kb
45kb
35kb
25kb
15kb
5kb
Mean LD between Chr20 10kb blocks
LD scale
0
0.1
0.2
0.3
0.4
0.5
0.6
0.7
0.8
0.9
1
Chr20.5kb
Chr20.15kb
Chr20.25kb
Chr20.35kb
Chr20.45kb
Chr20.55kb
Chr20.65kb
Chr20.75kb
Chr20.85kb
Chr20.95kb
Chr20.105kb
Chr20.115kb
Chr20.125kb
Chr20.135kb
Chr20.145kb
Chr20.155kb
Chr20.165kb
Chr20.175kb
Chr20.185kb
Chr20.195kb
Chr20.205kb
Chr20.215kb
Chr20.225kb
Chr20.235kb
Chr20.245kb
Chr20.255kb
Chr20.265kb
Chr20.275kb
Chr20.285kb
Chr20.295kb
Chr20.305kb
Chr20.315kb
Chr20.325kb
Chr20.335kb
Chr20.345kb
Chr20.355kb
Chr20.365kb
Chr20.375kb
Chr20.385kb
Chr20.395kb
Chr20.405kb
Chr20.415kb
Chr20.425kb
Chr20.435kb
Chr20.445kb
Chr20.455kb
Chr20.465kb
Chr20.475kb
Chr20.485kb
Chr20.495kb
Chr20.505kb
Chr20.515kb
Chr20.525kb
Chr20.535kb
Chr20.545kb
Chr20.555kb
Chr20.565kb
Chr20.575kb
Chr20.585kb
Chr20.595kb
Chr20.605kb
Chr20.615kb
Chr20.625kb
Chr20.635kb
Chr20.645kb
Chr20.655kb
Chr20.665kb
Chr20.675kb
Chr20.685kb
Chr20.695kb
Chr20.705kb
Chr20.715kb
Chr20.725kb
Chr20.735kb
Chr20.745kb
Chr20.755kb
Chr20

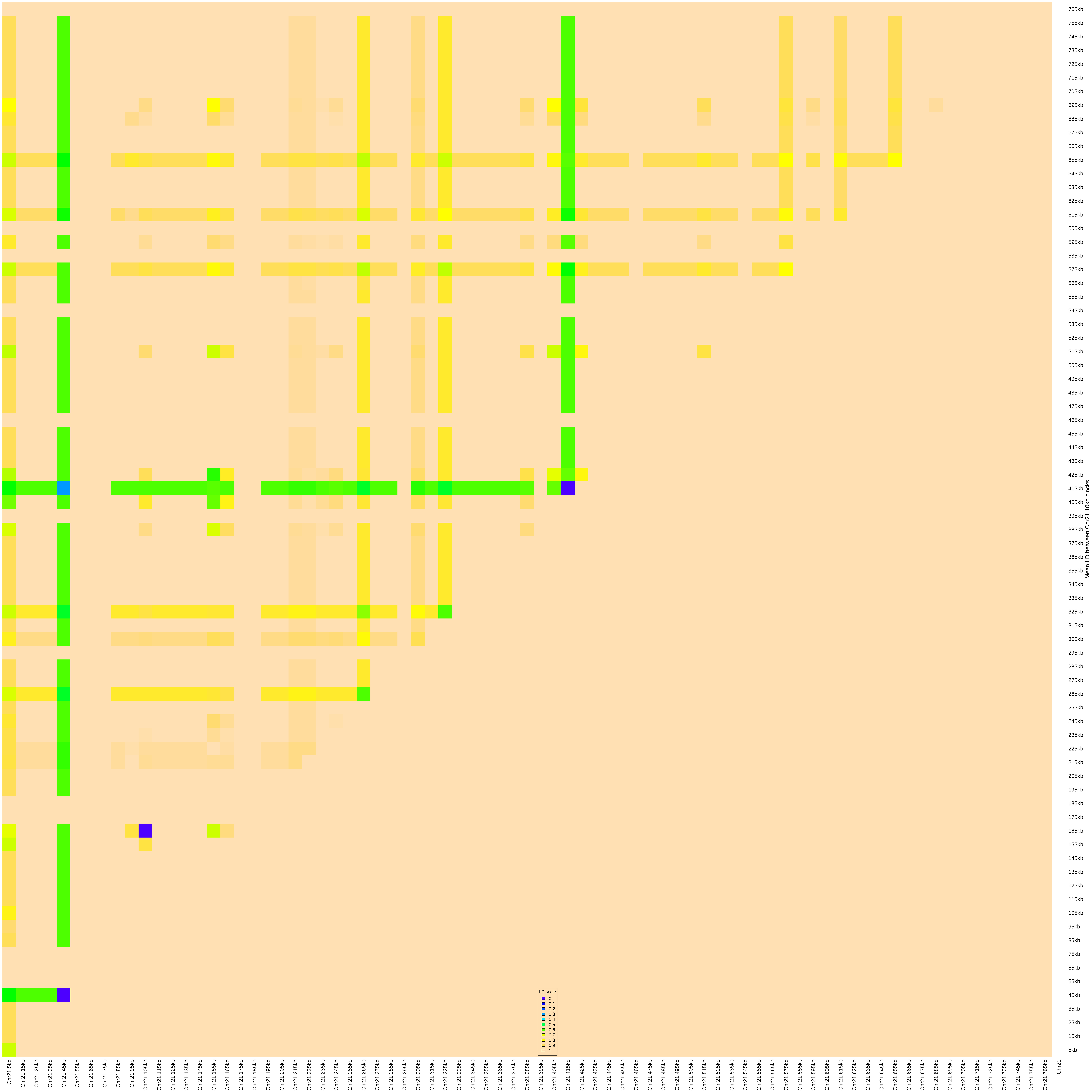
Mean LD between Chr21 10kb blocks
LD scale
0
0.1
0.2
0.3
0.4
0.5
0.6
0.7
0.8
0.9
1
765kb
755kb
745kb
735kb
725kb
715kb
705kb
695kb
685kb
675kb
665kb
655kb
645kb
635kb
625kb
615kb
605kb
595kb
585kb
575kb
565kb
555kb
545kb
535kb
525kb
515kb
505kb
495kb
485kb
475kb
465kb
455kb
445kb
435kb
425kb
415kb
405kb
395kb
385kb
375kb
365kb
355kb
345kb
335kb
325kb
315kb
305kb
295kb
285kb
275kb
265kb
255kb
245kb
235kb
225kb
215kb
205kb
195kb
185kb
175kb
165kb
155kb
145kb
135kb
125kb
115kb
105kb
95kb
85kb
75kb
65kb
55kb
45kb
35kb
25kb
15kb
5kb
Chr21.5kb
Chr21.15kb
Chr21.25kb
Chr21.35kb
Chr21.45kb
Chr21.55kb
Chr21.65kb
Chr21.75kb
Chr21.85kb
Chr21.95kb
Chr21.105kb
Chr21.115kb
Chr21.125kb
Chr21.135kb
Chr21.145kb
Chr21.155kb
Chr21.165kb
Chr21.175kb
Chr21.185kb
Chr21.195kb
Chr21.205kb
Chr21.215kb
Chr21.225kb
Chr21.235kb
Chr21.245kb
Chr21.255kb
Chr21.265kb
Chr21.275kb
Chr21.285kb
Chr21.295kb
Chr21.305kb
Chr21.315kb
Chr21.325kb
Chr21.335kb
Chr21.345kb
Chr21.355kb
Chr21.365kb
Chr21.375kb
Chr21.385kb
Chr21.395kb
Chr21.405kb
Chr21.415kb
Chr21.425kb
Chr21.435kb
Chr21.445kb
Chr21.455kb
Chr21.465kb
Chr21.475kb
Chr21.485kb
Chr21.495kb
Chr21.505kb
Chr21.515kb
Chr21.525kb
Chr21.535kb
Chr21.545kb
Chr21.555kb
Chr21.565kb
Chr21.575kb
Chr21.585kb
Chr21.595kb
Chr21.605kb
Chr21.615kb
Chr21.625kb
Chr21.635kb
Chr21.645kb
Chr21.655kb
Chr21.665kb
Chr21.675kb
Chr21.685kb
Chr21.695kb
Chr21.705kb
Chr21.715kb
Chr21.725kb
Chr21.735kb
Chr21.745kb
Chr21.755kb
Chr21.765kb
Chr21

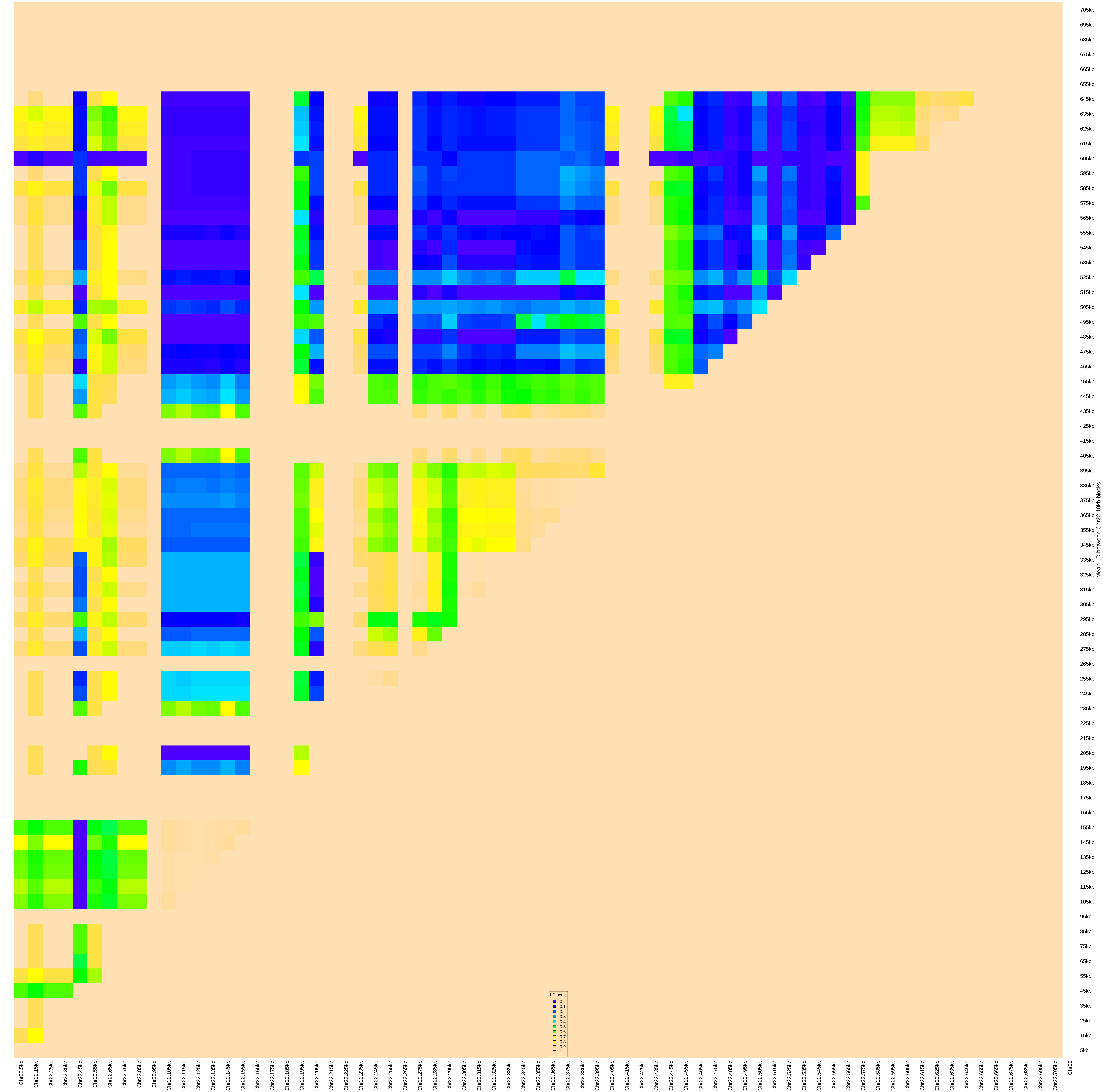
705kb
695kb
685kb
675kb
665kb
655kb
645kb
635kb
625kb
615kb
605kb
595kb
585kb
575kb
565kb
555kb
545kb
535kb
525kb
515kb
505kb
495kb
485kb
475kb
465kb
455kb
445kb
435kb
425kb
415kb
405kb
395kb
385kb
375kb
365kb
355kb
345kb
335kb
325kb
315kb
305kb
295kb
285kb
275kb
265kb
255kb
245kb
235kb
225kb
215kb
205kb
195kb
185kb
175kb
165kb
155kb
145kb
135kb
125kb
115kb
105kb
95kb
85kb
75kb
65kb
55kb
45kb
35kb
25kb
15kb
5kb
Mean LD between Chr22 10kb blocks
LD scale
0
0.1
0.2
0.3
0.4
0.5
0.6
0.7
0.8
0.9
1
Chr22.5kb
Chr22.15kb
Chr22.25kb
Chr22.35kb
Chr22.45kb
Chr22.55kb
Chr22.65kb
Chr22.75kb
Chr22.85kb
Chr22.95kb
Chr22.105kb
Chr22.115kb
Chr22.125kb
Chr22.135kb
Chr22.145kb
Chr22.155kb
Chr22.165kb
Chr22.175kb
Chr22.185kb
Chr22.195kb
Chr22.205kb
Chr22.215kb
Chr22.225kb
Chr22.235kb
Chr22.245kb
Chr22.255kb
Chr22.265kb
Chr22.275kb
Chr22.285kb
Chr22.295kb
Chr22.305kb
Chr22.315kb
Chr22.325kb
Chr22.335kb
Chr22.345kb
Chr22.355kb
Chr22.365kb
Chr22.375kb
Chr22.385kb
Chr22.395kb
Chr22.405kb
Chr22.415kb
Chr22.425kb
Chr22.435kb
Chr22.445kb
Chr22.455kb
Chr22.465kb
Chr22.475kb
Chr22.485kb
Chr22.495kb
Chr22.505kb
Chr22.515kb
Chr22.525kb
Chr22.535kb
Chr22.545kb
Chr22.555kb
Chr22.565kb
Chr22.575kb
Chr22.585kb
Chr22.595kb
Chr22.605kb
Chr22.615kb
Chr22.625kb
Chr22.635kb
Chr22.645kb
Chr22.655kb
Chr22.665kb
Chr22.675kb
Chr22.685kb
Chr22.695kb
Chr22.705kb
Chr22

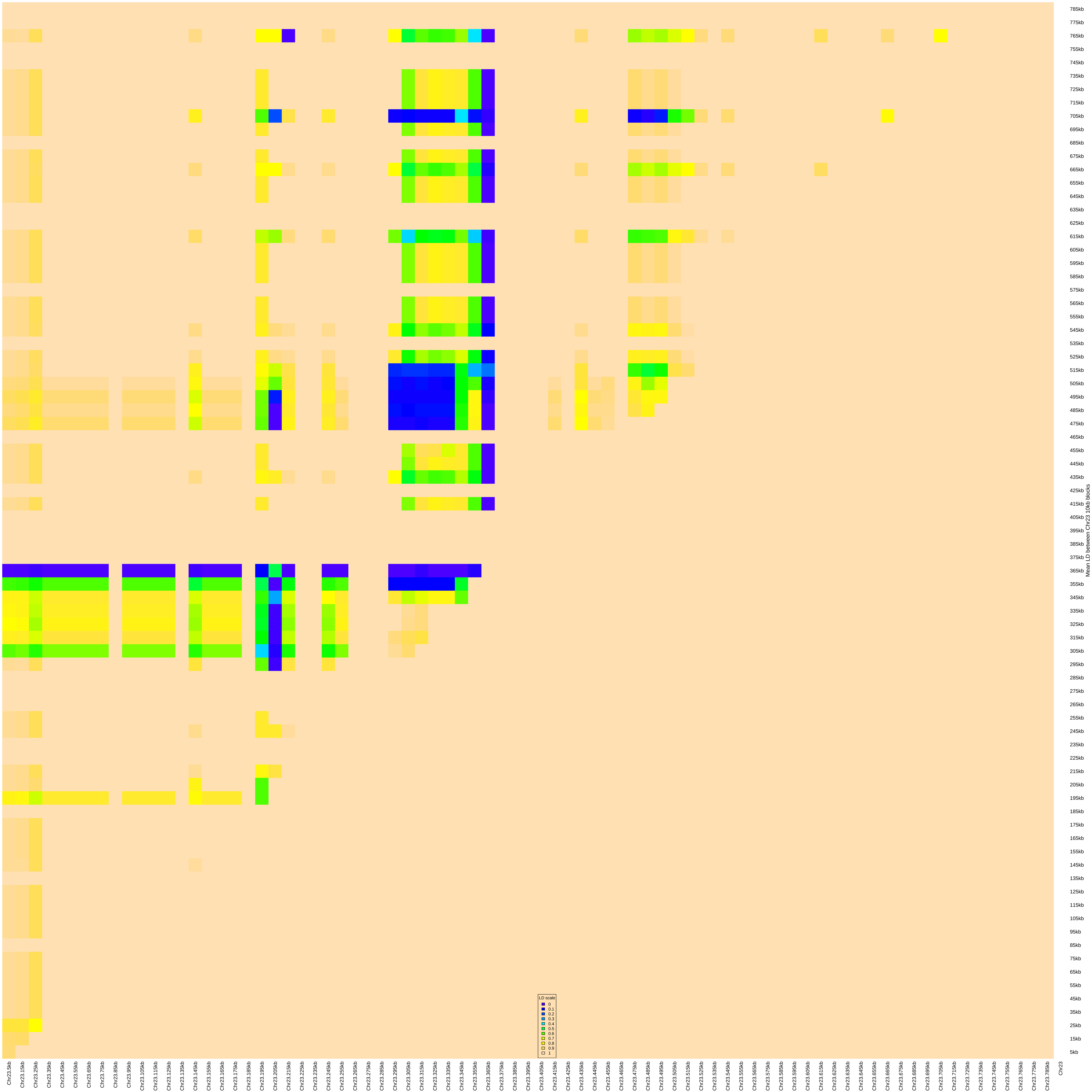
Mean LD between Chr23 10kb blocks
LD scale
0
0.1
0.2
0.3
0.4
0.5
0.6
0.7
0.8
0.9
1
785kb
775kb
765kb
755kb
745kb
735kb
725kb
715kb
705kb
695kb
685kb
675kb
665kb
655kb
645kb
635kb
625kb
615kb
605kb
595kb
585kb
575kb
565kb
555kb
545kb
535kb
525kb
515kb
505kb
495kb
485kb
475kb
465kb
455kb
445kb
435kb
425kb
415kb
405kb
395kb
385kb
375kb
365kb
355kb
345kb
335kb
325kb
315kb
305kb
295kb
285kb
275kb
265kb
255kb
245kb
235kb
225kb
215kb
205kb
195kb
185kb
175kb
165kb
155kb
145kb
135kb
125kb
115kb
105kb
95kb
85kb
75kb
65kb
55kb
45kb
35kb
25kb
15kb
5kb
Chr23.5kb
Chr23.15kb
Chr23.25kb
Chr23.35kb
Chr23.45kb
Chr23.55kb
Chr23.65kb
Chr23.75kb
Chr23.85kb
Chr23.95kb
Chr23.105kb
Chr23.115kb
Chr23.125kb
Chr23.135kb
Chr23.145kb
Chr23.155kb
Chr23.165kb
Chr23.175kb
Chr23.185kb
Chr23.195kb
Chr23.205kb
Chr23.215kb
Chr23.225kb
Chr23.235kb
Chr23.245kb
Chr23.255kb
Chr23.265kb
Chr23.275kb
Chr23.285kb
Chr23.295kb
Chr23.305kb
Chr23.315kb
Chr23.325kb
Chr23.335kb
Chr23.345kb
Chr23.355kb
Chr23.365kb
Chr23.375kb
Chr23.385kb
Chr23.395kb
Chr23.405kb
Chr23.415kb
Chr23.425kb
Chr23.435kb
Chr23.445kb
Chr23.455kb
Chr23.465kb
Chr23.475kb
Chr23.485kb
Chr23.495kb
Chr23.505kb
Chr23.515kb
Chr23.525kb
Chr23.535kb
Chr23.545kb
Chr23.555kb
Chr23.565kb
Chr23.575kb
Chr23.585kb
Chr23.595kb
Chr23.605kb
Chr23.615kb
Chr23.625kb
Chr23.635kb
Chr23.645kb
Chr23.655kb
Chr23.665kb
Chr23.675kb
Chr23.685kb
Chr23.695kb
Chr23.705kb
Chr23.715kb
Chr23.725kb
Chr23.735kb
Chr23.745kb
Chr23.755kb
Chr23.765kb
Chr23.775kb
Chr23.785kb
Chr23

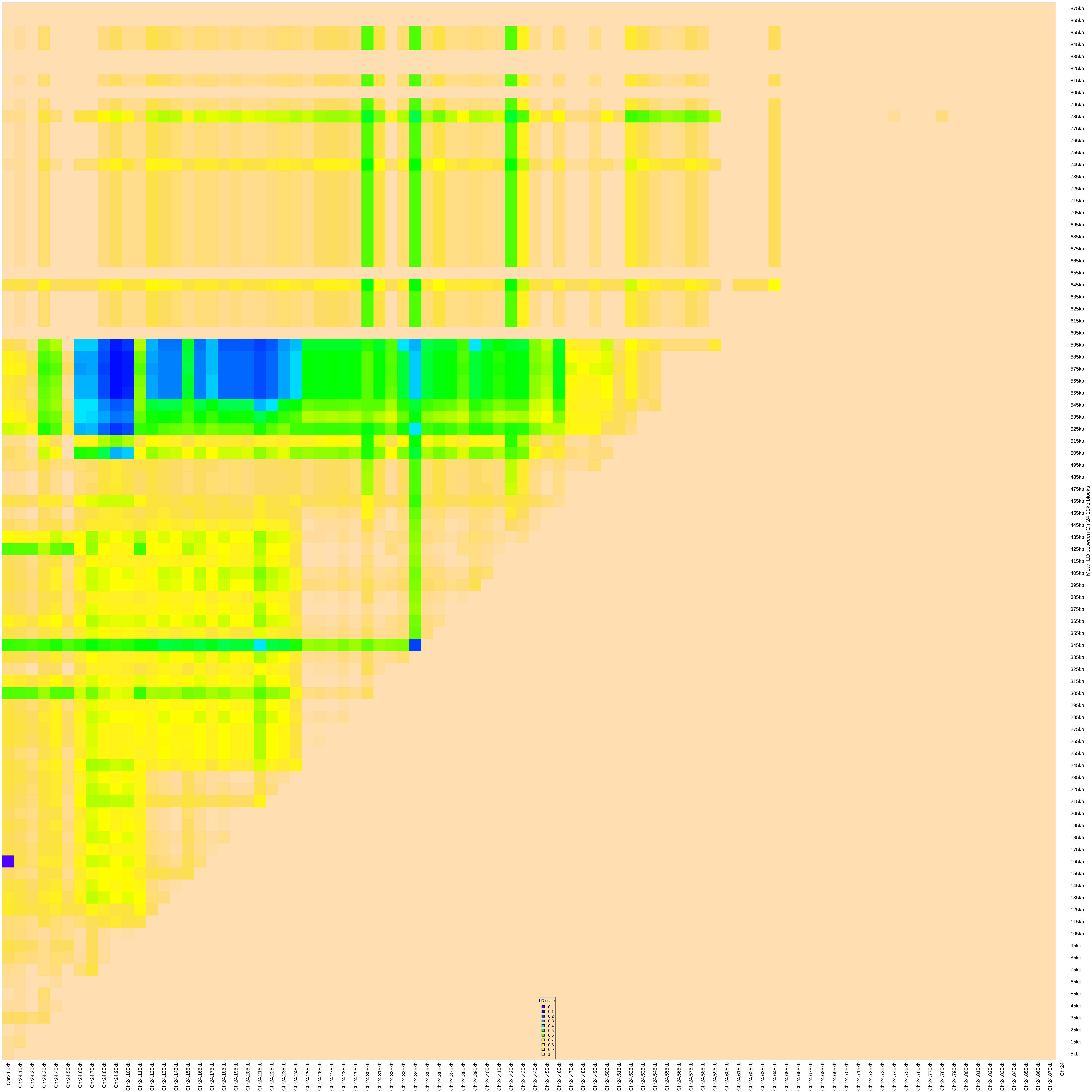
Mean LD between Chr24 10kb blocks
LD scale
0
0.1
0.2
0.3
0.4
0.5
0.6
0.7
0.8
0.9
1
875kb
865kb
855kb
845kb
835kb
825kb
815kb
805kb
795kb
785kb
775kb
765kb
755kb
745kb
735kb
725kb
715kb
705kb
695kb
685kb
675kb
665kb
655kb
645kb
635kb
625kb
615kb
605kb
595kb
585kb
575kb
565kb
555kb
545kb
535kb
525kb
515kb
505kb
495kb
485kb
475kb
465kb
455kb
445kb
435kb
425kb
415kb
405kb
395kb
385kb
375kb
365kb
355kb
345kb
335kb
325kb
315kb
305kb
295kb
285kb
275kb
265kb
255kb
245kb
235kb
225kb
215kb
205kb
195kb
185kb
175kb
165kb
155kb
145kb
135kb
125kb
115kb
105kb
95kb
85kb
75kb
65kb
55kb
45kb
35kb
25kb
15kb
5kb
Chr24.5kb
Chr24.15kb
Chr24.25kb
Chr24.35kb
Chr24.45kb
Chr24.55kb
Chr24.65kb
Chr24.75kb
Chr24.85kb
Chr24.95kb
Chr24.105kb
Chr24.115kb
Chr24.125kb
Chr24.135kb
Chr24.145kb
Chr24.155kb
Chr24.165kb
Chr24.175kb
Chr24.185kb
Chr24.195kb
Chr24.205kb
Chr24.215kb
Chr24.225kb
Chr24.235kb
Chr24.245kb
Chr24.255kb
Chr24.265kb
Chr24.275kb
Chr24.285kb
Chr24.295kb
Chr24.305kb
Chr24.315kb
Chr24.325kb
Chr24.335kb
Chr24.345kb
Chr24.355kb
Chr24.365kb
Chr24.375kb
Chr24.385kb
Chr24.395kb
Chr24.405kb
Chr24.415kb
Chr24.425kb
Chr24.435kb
Chr24.445kb
Chr24.455kb
Chr24.465kb
Chr24.475kb
Chr24.485kb
Chr24.495kb
Chr24.505kb
Chr24.515kb
Chr24.525kb
Chr24.535kb
Chr24.545kb
Chr24.555kb
Chr24.565kb
Chr24.575kb
Chr24.585kb
Chr24.595kb
Chr24.605kb
Chr24.615kb
Chr24.625kb
Chr24.635kb
Chr24.645kb
Chr24.655kb
Chr24.665kb
Chr24.675kb
Chr24.685kb
Chr24.695kb
Chr24.705kb
Chr24.715kb
Chr24.725kb
Chr24.735kb
Chr24.745kb
Chr24.755kb
Chr24.765kb
Chr24.775kb
Chr24.785kb
Chr24.795kb
Chr24.805kb
Chr24.815kb
Chr24.825kb
Chr24.835kb
Chr24.845kb
Chr24.855kb
Chr24.865kb
Chr24.875kb
Chr24

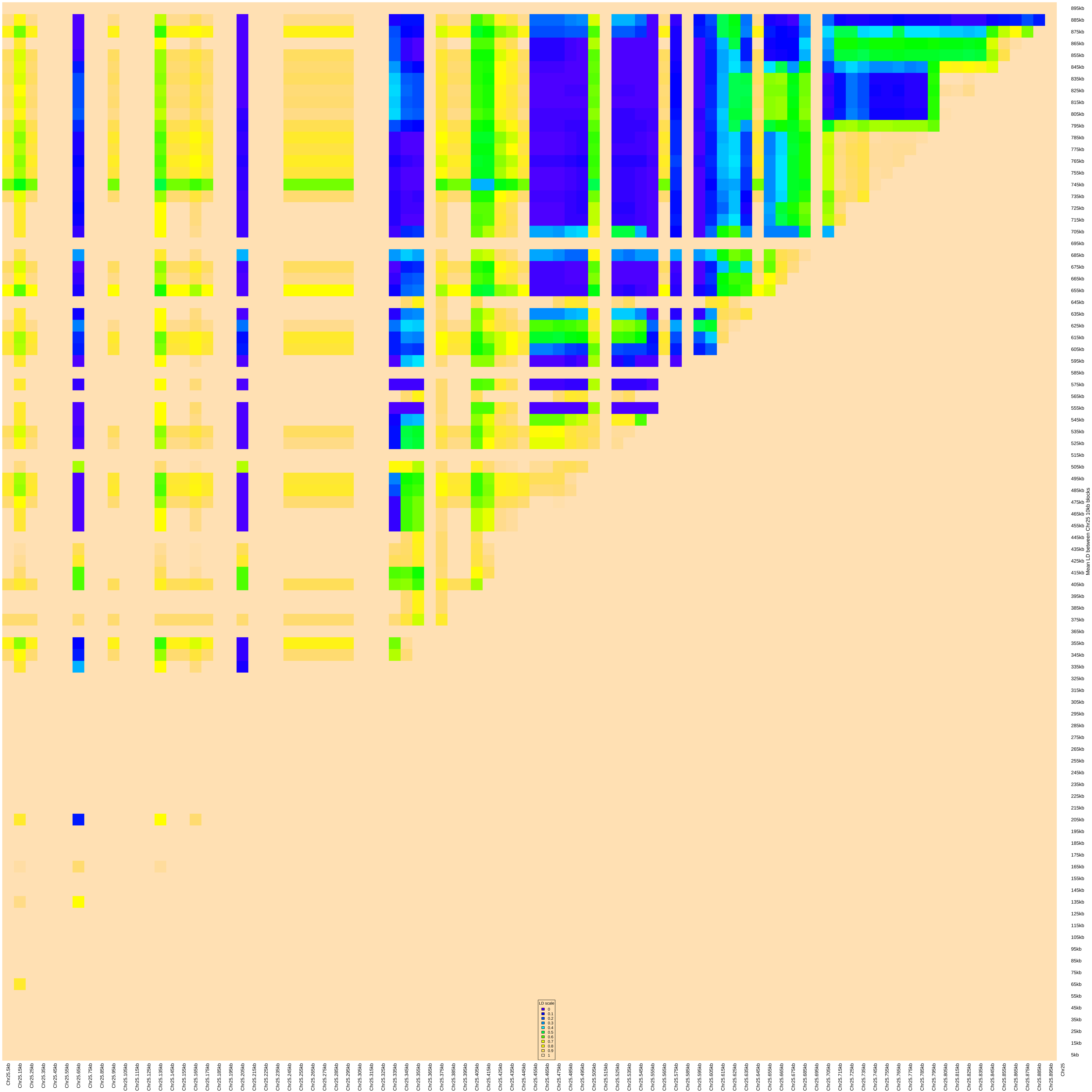
Mean LD between Chr25 10kb blocks
LD scale
0
0.1
0.2
0.3
0.4
0.5
0.6
0.7
0.8
0.9
1
895kb
885kb
875kb
865kb
855kb
845kb
835kb
825kb
815kb
805kb
795kb
785kb
775kb
765kb
755kb
745kb
735kb
725kb
715kb
705kb
695kb
685kb
675kb
665kb
655kb
645kb
635kb
625kb
615kb
605kb
595kb
585kb
575kb
565kb
555kb
545kb
535kb
525kb
515kb
505kb
495kb
485kb
475kb
465kb
455kb
445kb
435kb
425kb
415kb
405kb
395kb
385kb
375kb
365kb
355kb
345kb
335kb
325kb
315kb
305kb
295kb
285kb
275kb
265kb
255kb
245kb
235kb
225kb
215kb
205kb
195kb
185kb
175kb
165kb
155kb
145kb
135kb
125kb
115kb
105kb
95kb
85kb
75kb
65kb
55kb
45kb
35kb
25kb
15kb
5kb
Chr25.5kb
Chr25.15kb
Chr25.25kb
Chr25.35kb
Chr25.45kb
Chr25.55kb
Chr25.65kb
Chr25.75kb
Chr25.85kb
Chr25.95kb
Chr25.105kb
Chr25.115kb
Chr25.125kb
Chr25.135kb
Chr25.145kb
Chr25.155kb
Chr25.165kb
Chr25.175kb
Chr25.185kb
Chr25.195kb
Chr25.205kb
Chr25.215kb
Chr25.225kb
Chr25.235kb
Chr25.245kb
Chr25.255kb
Chr25.265kb
Chr25.275kb
Chr25.285kb
Chr25.295kb
Chr25.305kb
Chr25.315kb
Chr25.325kb
Chr25.335kb
Chr25.345kb
Chr25.355kb
Chr25.365kb
Chr25.375kb
Chr25.385kb
Chr25.395kb
Chr25.405kb
Chr25.415kb
Chr25.425kb
Chr25.435kb
Chr25.445kb
Chr25.455kb
Chr25.465kb
Chr25.475kb
Chr25.485kb
Chr25.495kb
Chr25.505kb
Chr25.515kb
Chr25.525kb
Chr25.535kb
Chr25.545kb
Chr25.555kb
Chr25.565kb
Chr25.575kb
Chr25.585kb
Chr25.595kb
Chr25.605kb
Chr25.615kb
Chr25.625kb
Chr25.635kb
Chr25.645kb
Chr25.655kb
Chr25.665kb
Chr25.675kb
Chr25.685kb
Chr25.695kb
Chr25.705kb
Chr25.715kb
Chr25.725kb
Chr25.735kb
Chr25.745kb
Chr25.755kb
Chr25.765kb
Chr25.775kb
Chr25.785kb
Chr25.795kb
Chr25.805kb
Chr25.815kb
Chr25.825kb
Chr25.835kb
Chr25.845kb
Chr25.855kb
Chr25.865kb
Chr25.875kb
Chr25.885kb
Chr25.895kb
Chr25

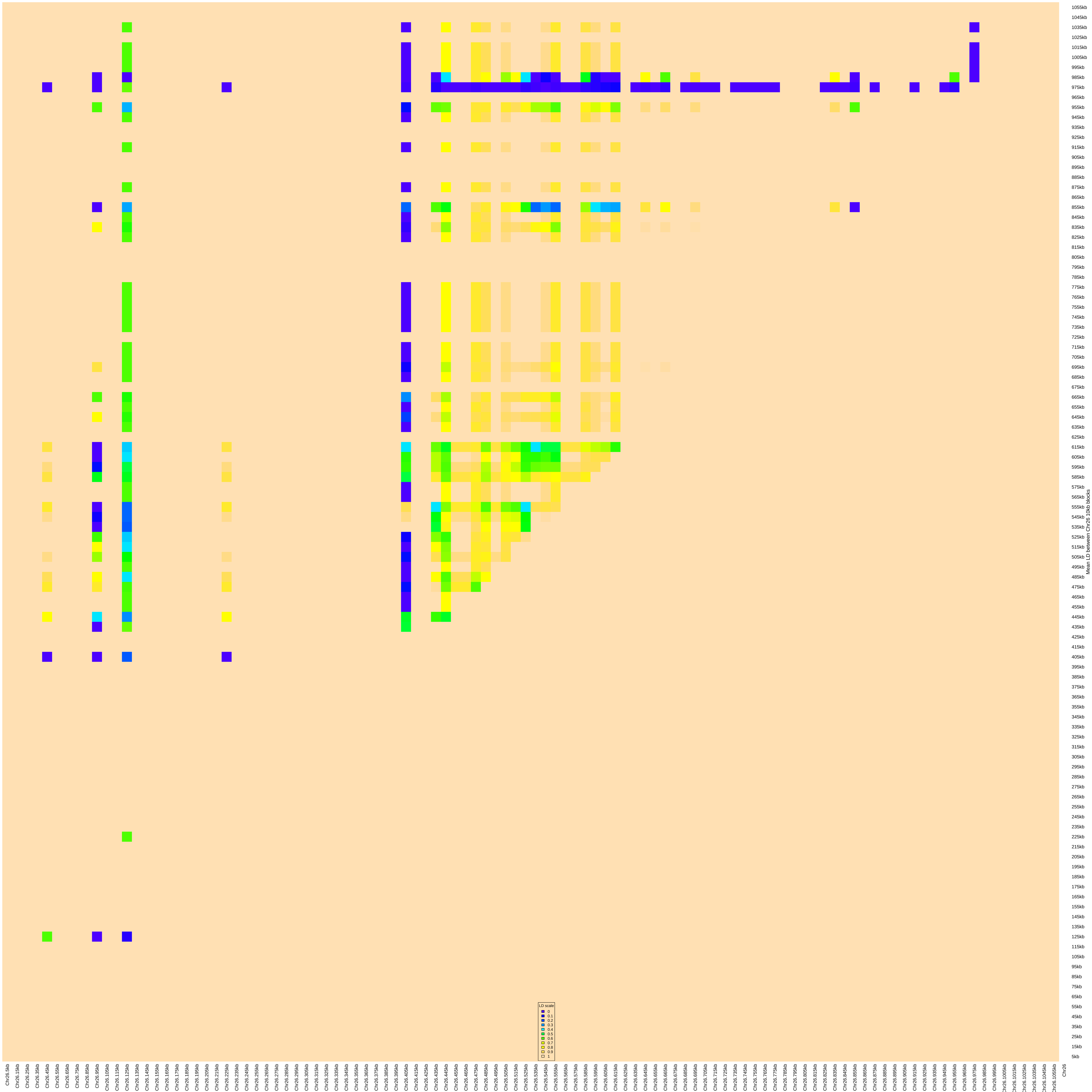
Mean LD between Chr26 10kb blocks
LD scale
0
0.1
0.2
0.3
0.4
0.5
0.6
0.7
0.8
0.9
1
Chr26

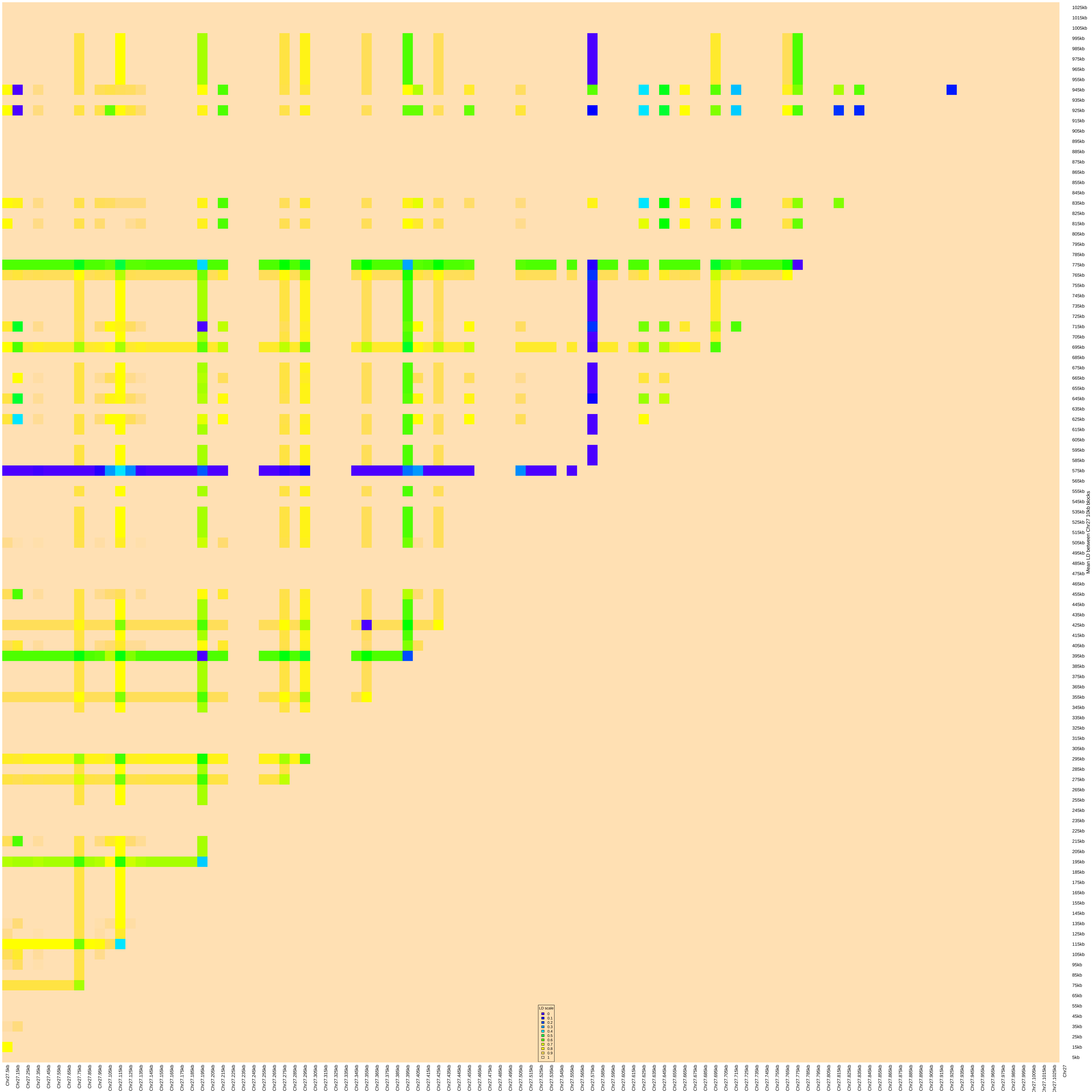
Mean LD between Chr27 10kb blocks
LD scale
0
0.1
0.2
0.3
0.4
0.5
0.6
0.7
0.8
0.9
1
1025kb
1015kb
1005kb
995kb
985kb
975kb
965kb
955kb
945kb
935kb
925kb
915kb
905kb
895kb
885kb
875kb
865kb
855kb
845kb
835kb
825kb
815kb
805kb
795kb
785kb
775kb
765kb
755kb
745kb
735kb
725kb
715kb
705kb
695kb
685kb
675kb
665kb
655kb
645kb
635kb
625kb
615kb
605kb
595kb
585kb
575kb
565kb
555kb
545kb
535kb
525kb
515kb
505kb
495kb
485kb
475kb
465kb
455kb
445kb
435kb
425kb
415kb
405kb
395kb
385kb
375kb
365kb
355kb
345kb
335kb
325kb
315kb
305kb
295kb
285kb
275kb
265kb
255kb
245kb
235kb
225kb
215kb
205kb
195kb
185kb
175kb
165kb
155kb
145kb
135kb
125kb
115kb
105kb
95kb
85kb
75kb
65kb
55kb
45kb
35kb
25kb
15kb
5kb
Chr27.5kb
Chr27.15kb
Chr27.25kb
Chr27.35kb
Chr27.45kb
Chr27.55kb
Chr27.65kb
Chr27.75kb
Chr27.85kb
Chr27.95kb
Chr27.105kb
Chr27.115kb
Chr27.125kb
Chr27.135kb
Chr27.145kb
Chr27.155kb
Chr27.165kb
Chr27.175kb
Chr27.185kb
Chr27.195kb
Chr27.205kb
Chr27.215kb
Chr27.225kb
Chr27.235kb
Chr27.245kb
Chr27.255kb
Chr27.265kb
Chr27.275kb
Chr27.285kb
Chr27.295kb
Chr27.305kb
Chr27.315kb
Chr27.325kb
Chr27.335kb
Chr27.345kb
Chr27.355kb
Chr27.365kb
Chr27.375kb
Chr27.385kb
Chr27.395kb
Chr27.405kb
Chr27.415kb
Chr27.425kb
Chr27.435kb
Chr27.445kb
Chr27.455kb
Chr27.465kb
Chr27.475kb
Chr27.485kb
Chr27.495kb
Chr27.505kb
Chr27.515kb
Chr27.525kb
Chr27.535kb
Chr27.545kb
Chr27.555kb
Chr27.565kb
Chr27.575kb
Chr27.585kb
Chr27.595kb
Chr27.605kb
Chr27.615kb
Chr27.625kb
Chr27.635kb
Chr27.645kb
Chr27.655kb
Chr27.665kb
Chr27.675kb
Chr27.685kb
Chr27.695kb
Chr27.705kb
Chr27.715kb
Chr27.725kb
Chr27.735kb
Chr27.745kb
Chr27.755kb
Chr27.765kb
Chr27.775kb
Chr27.785kb
Chr27.795kb
Chr27.805kb
Chr27.815kb
Chr27.825kb
Chr27.835kb
Chr27.845kb
Chr27.855kb
Chr27.865kb
Chr27.875kb
Chr27.885kb
Chr27.895kb
Chr27.905kb
Chr27.915kb
Chr27.925kb
Chr27.935kb
Chr27.945kb
Chr27.955kb
Chr27.965kb
Chr27.975kb
Chr27.985kb
Chr27.995kb
Chr27.1005kb
Chr27.1015kb
Chr27.1025kb
Chr27

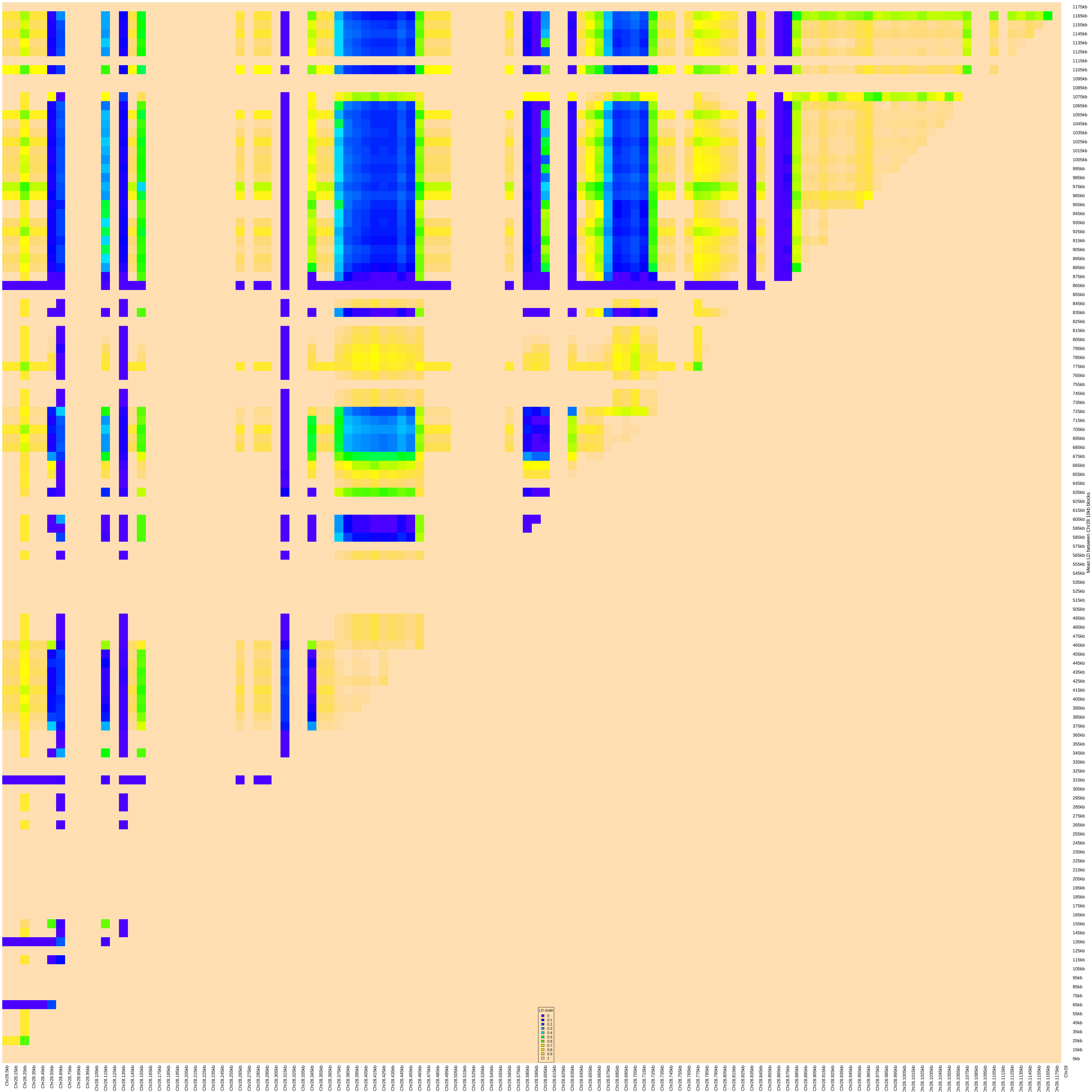
Mean LD between Chr28 10kb blocks
Chr28
LD scale
0
0.1
0.2
0.3
0.4
0.5
0.6
0.7
0.8
0.9
1

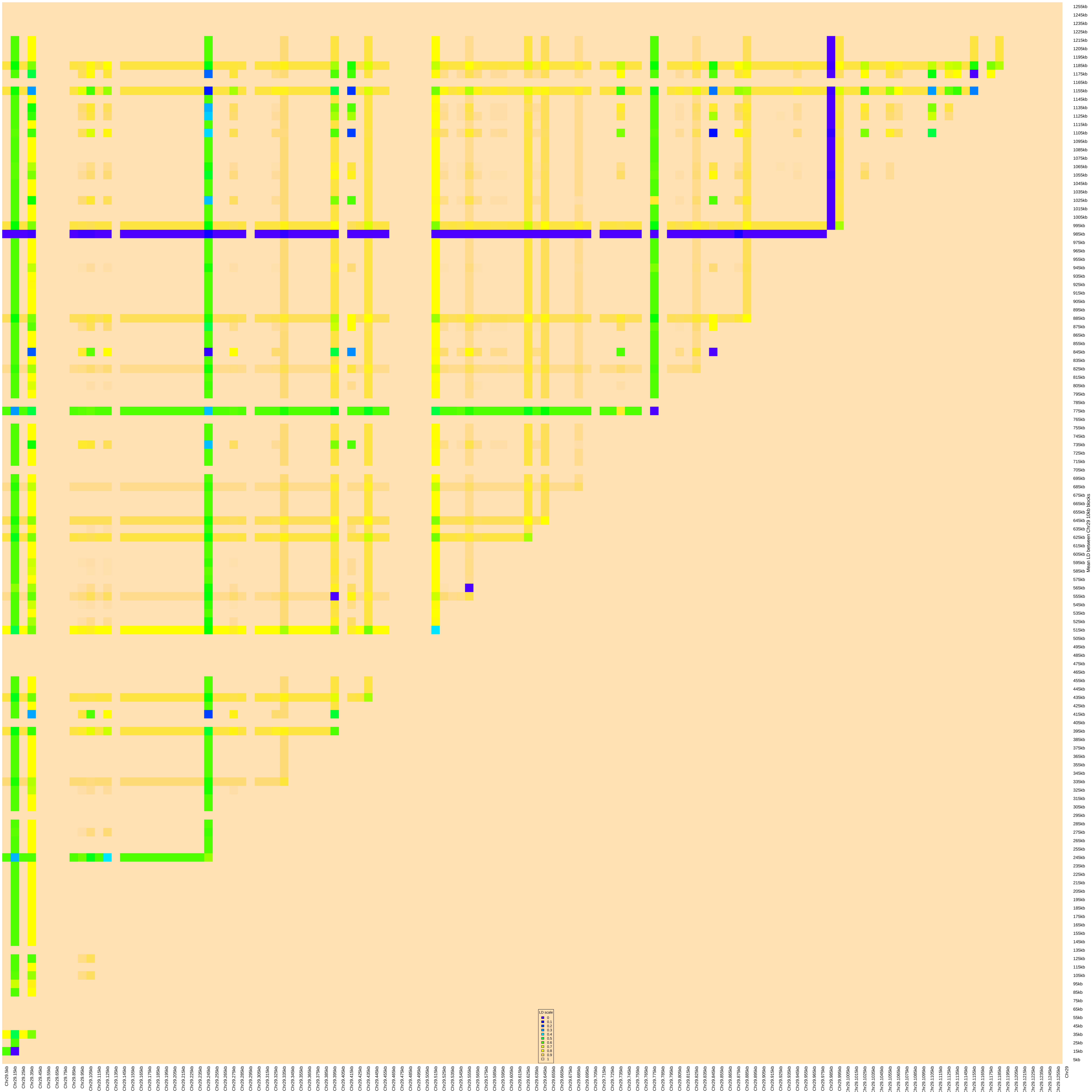
Mean LD between Chr29 10kb blocks
Chr29
LD scale
0
0.1
0.2
0.3
0.4
0.5
0.6
0.7
0.8
0.9
1

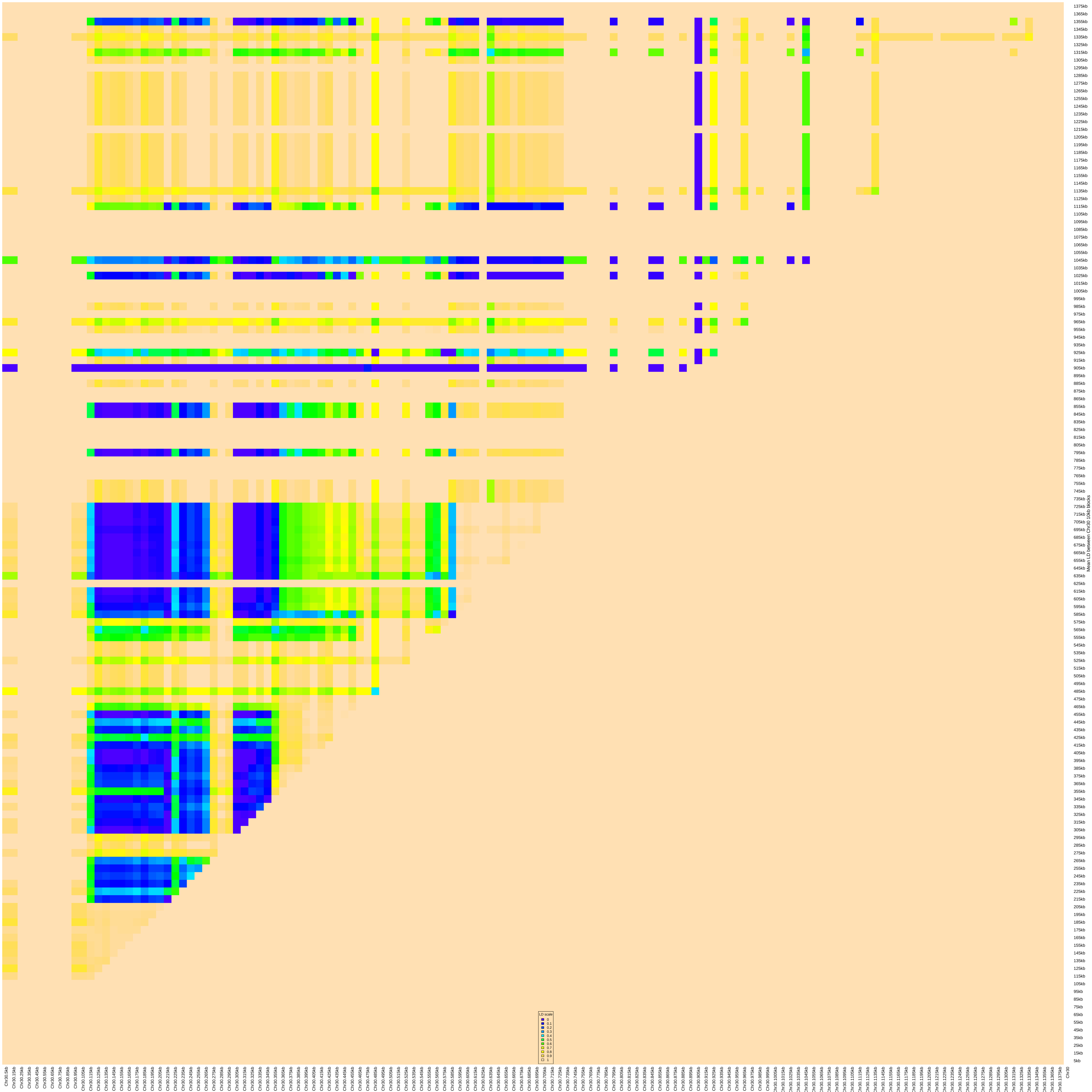
Mean LD between Chr30 10kb blocks
LD scale
0
0.1
0.2
0.3
0.4
0.5
0.6
0.7
0.8
0.9
1

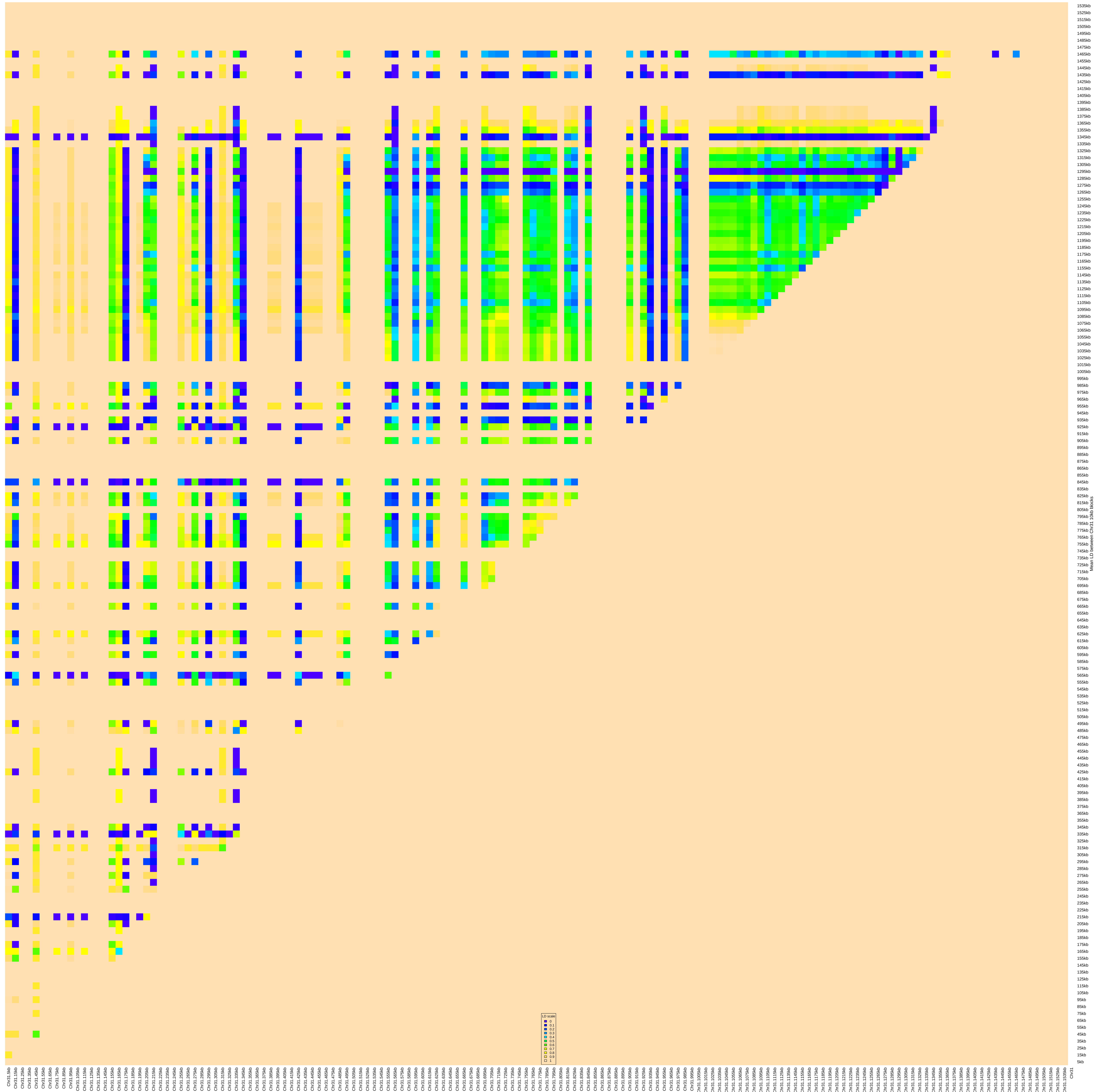
LD scale
0
0.1
0.2
0.3
0.4
0.5
0.6
0.7
0.8
0.9
1
Mean LD between Chr31 10kb blocks
Chr31

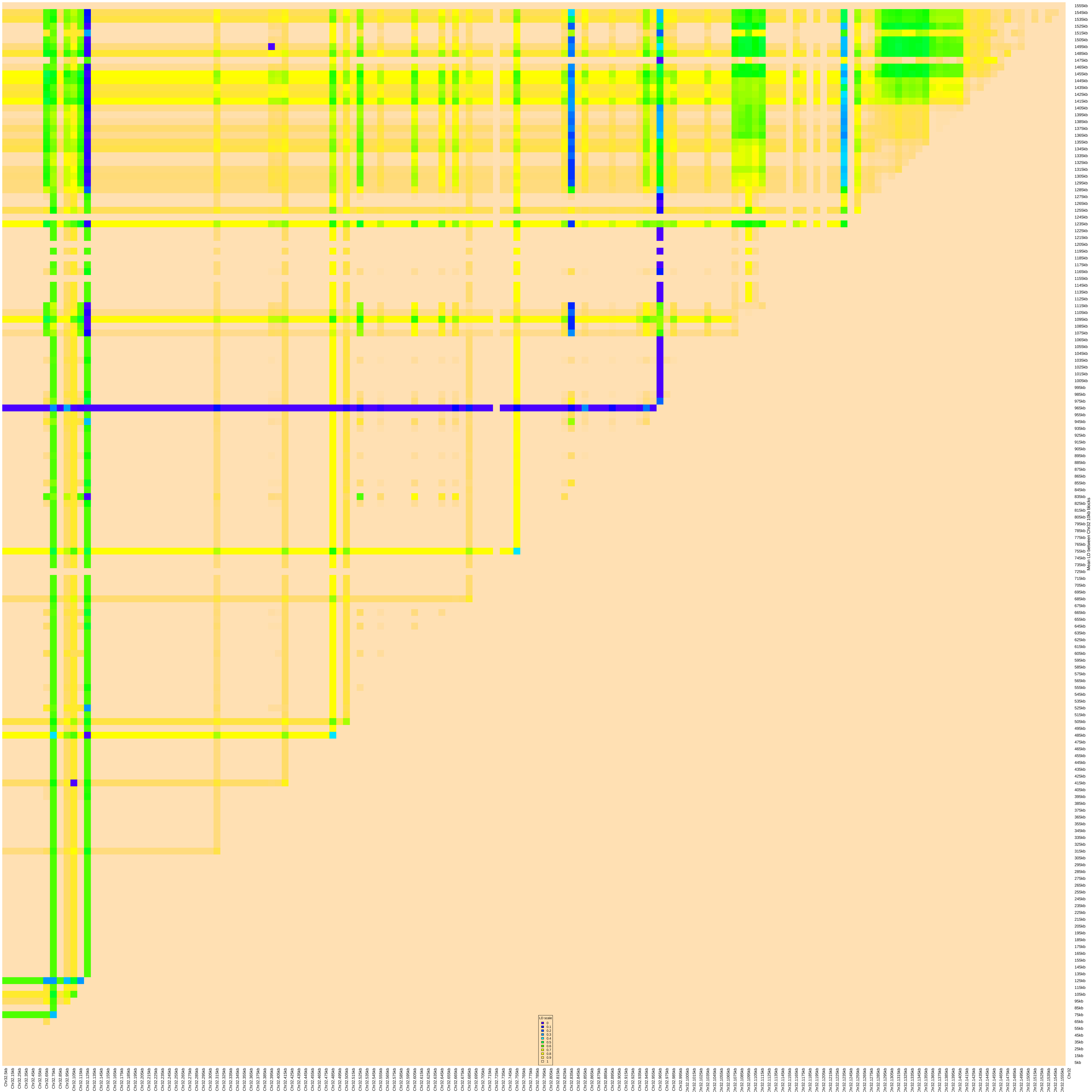
Mean LD between Chr32 10kb blocks
LD scale
0
0.1
0.2
0.3
0.4
0.5
0.6
0.7
0.8
0.9
1

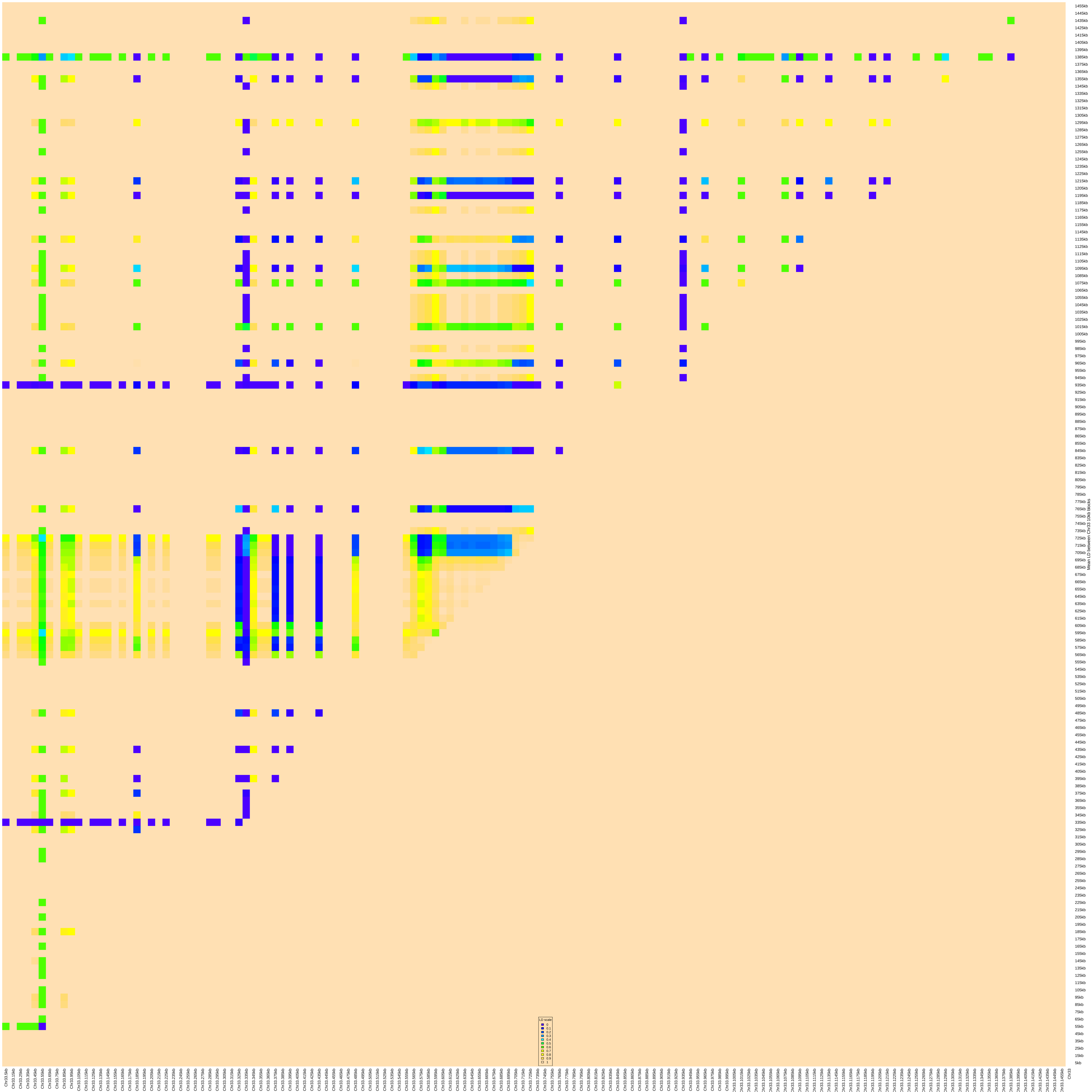
Mean LD between Chr33 10kb blocks
LD scale
0
0.1
0.2
0.3
0.4
0.5
0.6
0.7
0.8
0.9
1

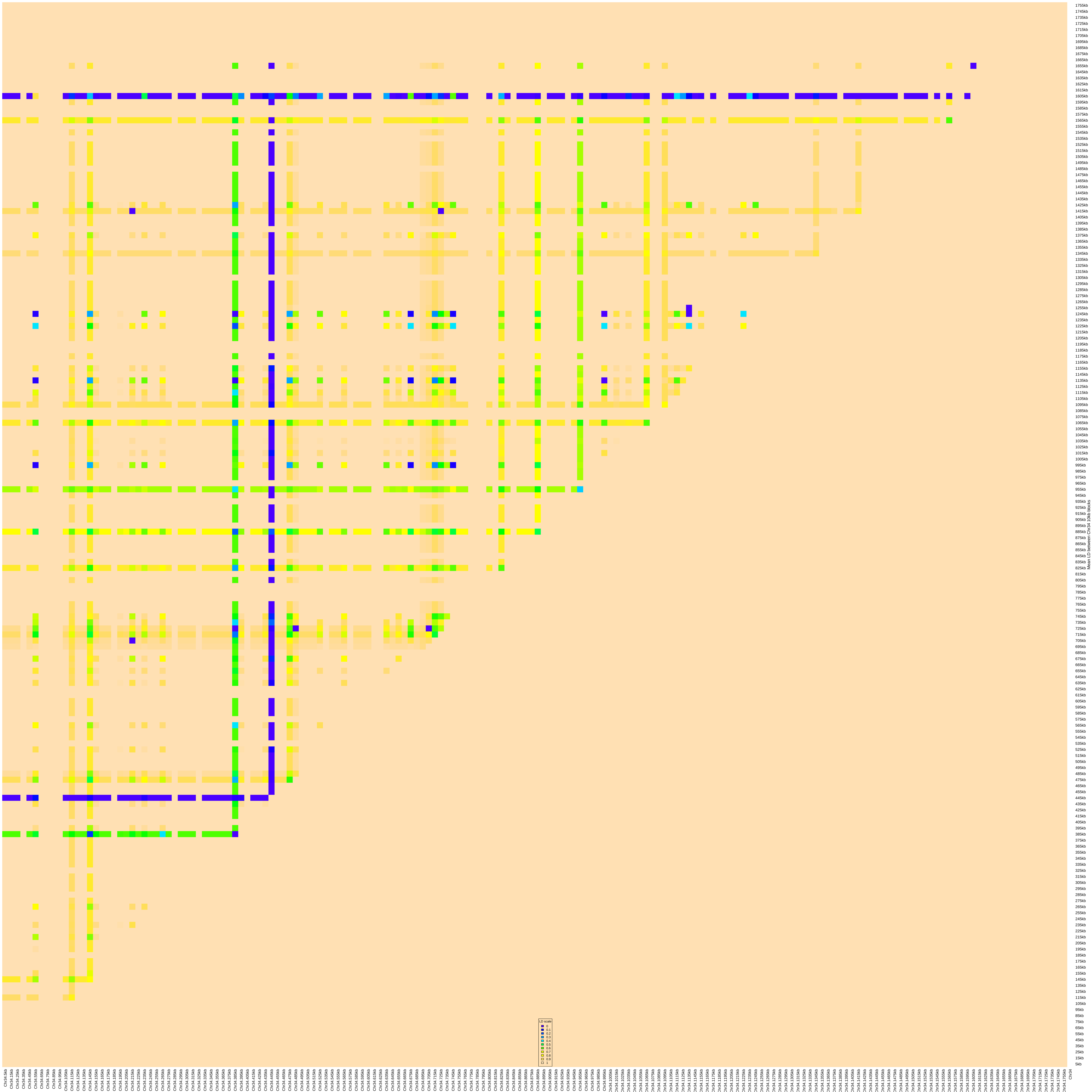
Mean LD between Chr34 10kb blocks
Chr34
LD scale
0
0.1
0.2
0.3
0.4
0.5
0.6
0.7
0.8
0.9
1

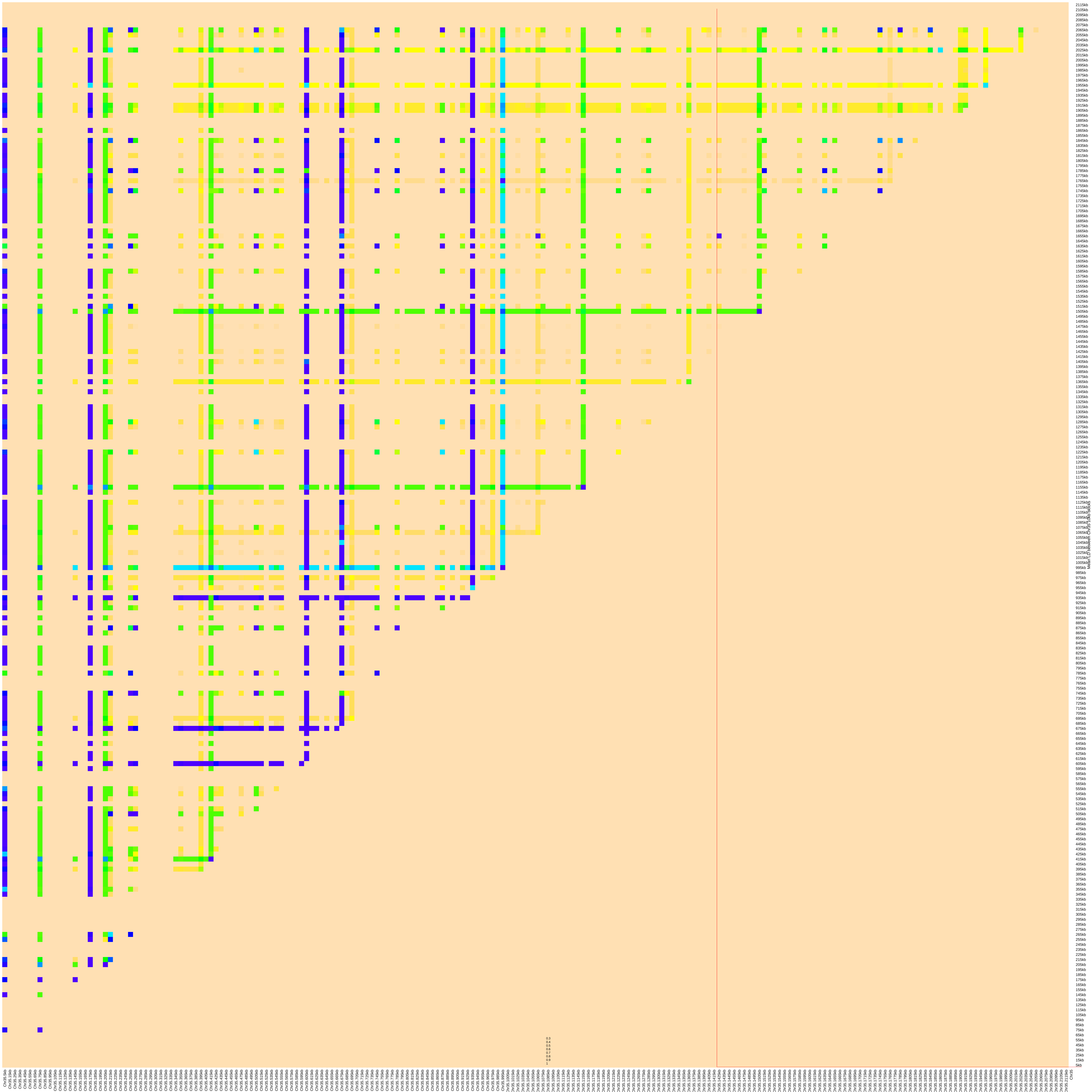
0.3
0.4
0.5
0.6
0.7
0.8
0.9
1

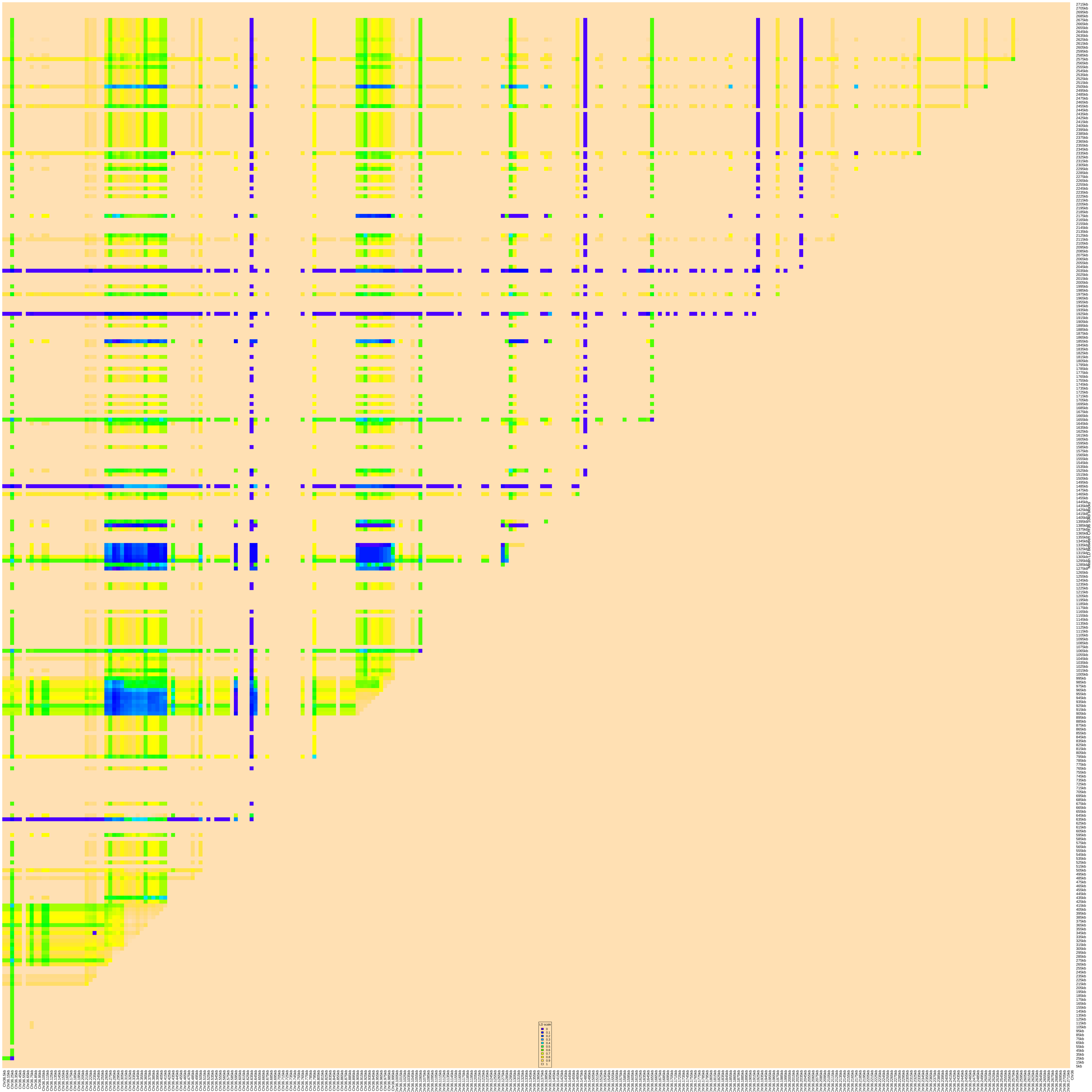
LD scale
0
0.1
0.2
0.3
0.4
0.5
0.6
0.7
0.8
0.9
1
