# Supplementary material for: Genomic Confirmation of Hybridisation and Recent Inbreeding in a Vector-Isolated Leishmania Population
Source: PLoS Genet. 2014 Jan 16;10(1):e1004092. doi: 10.1371/journal.pgen.1004092 (PMC3894156; doi:10.1371/journal.pgen.1004092)
Supplement: Table S1 — Source of the CUK strains analysed in this study. Data are provided on the WHO code, source (vector or host), geographical location and coordinates, date of isolation for each strain used (referred to as the CUK strains (1–12) throughout) and accession numbers for the DNA sequence data. (PDF) [file pgen.1004092.s018.pdf]

Table S1. Source of the CUK strains analysed in this study.

WHO code/name	Origin	Locality	Coordinates	Date of isolation	Accession number for sequence data
MHOM/TR/2005/HUM 1 (CUK1)	human isolate (male 27 yr)	Tepecikören/ Zerdali	37°21'51''N, 35°37'39''E	23.9. 2005	ERS026255
ITOB/TR/2005/TOB1 (CUK2)	*Phlebotomus tobbi*	Zerdali, house Y	37°25'57''N, 35°37'17''E	23.9. 2005	ERS026256
ITOB/TR/2005/TOB2 (CUK3)	*Phlebotomus tobbi*	Otluk, house G	37°18'2''N, 35°31'7''E	26.9. 2005	ERS026257
ITOB/TR/2006/TOB4 (CUK4)	*Phlebotomus tobbi*	Pirsuntanli, house S	37°17'40''N, 36°19'12''E	19.9. 2006	ERS026258
ITOB/TR/2006/TOB5 (CUK5)	*Phlebotomus tobbi*	Boyali, house K	37°17'50''N, 36°21'39''E	19.9. 2006	ERS026259
ITOB/TR/2006/TOB6 (CUK6)	*Phlebotomus tobbi*	Pirsuntanli, house S	37°17'40''N, 36°19'12''E	19.9. 2006	ERS026260
ITOB/TR/2006/TOB7 (CUK7)	*Phlebotomus tobbi*	Pirsuntanli, house S	37°17'40''N, 36°19'12''E	19.9. 2006	ERS026261
ITOB/TR/2006/TOB8 (CUK8)	*Phlebotomus tobbi*	Boyali, house K	37°17'50''N, 36°21'39''E	16.9. 2006	ERS026262
ITOB/TR/2007/TOB9 (CUK9)	*Phlebotomus tobbi*	Bayandirli, house B	37°18'41''N, 36°22'51''E	25.9. 2007	ERS026263
ITOB/TR/2007/TOB10 (CUK10)	*Phlebotomus tobbi*	Bayandirli, house A	37°18'41''N, 36°22'51''E	25.9. 2007	ERS026264
ITOB/TR/2007/TOB11 (CUK11)	*Phlebotomus tobbi*	Bayandirli, house A	37°18'41''N, 36°22'51''E	25.9. 2007	ERS026265
ITOB/TR/2007/TOB12 (CUK12)	*Phlebotomus tobbi*	Bayandirli house A	37°18'41''N, 36°22'51''E	25.9. 2007	ERS026266

Data are provided on the WHO code, source (vector or host), geographical location and coordinates, date of isolation and sequence data for each strain used; these are referred to as the CUK strains (1-12) throughout. Accession numbers are for the sequence read data in the European Nucleotide Archive (ENA). Data for all 12 isolates can be obtained via study ERP000588 (http://www.ebi.ac.uk/ena/data/view/ERP000588).
