# Supplementary material for: Genomic Confirmation of Hybridisation and Recent Inbreeding in a Vector-Isolated Leishmania Population
Source: PLoS Genet. 2014 Jan 16;10(1):e1004092. doi: 10.1371/journal.pgen.1004092 (PMC3894156; doi:10.1371/journal.pgen.1004092)
Supplement: Table S2 — Genome-wide read-mapping coverage levels for all 12 CUK strains compared to (A, top) the L. infantum and (B, bottom) the L. donovani reference genomes. (PDF) [file pgen.1004092.s019.pdf]

Table S2. Genome-wide read-mapping coverage levels for the 12 CUK strains relative to the *L. infantum* (A) and *L. donovani* (B) reference genomes.

Strain	Total #sites	Coverage	
		Mean	Median
CUK1	31,477,655	51.8	50
CUK2	31,470,599	50.1	49
CUK3	31,465,185	49.8	49
CUK4	31,487,776	57.5	56
CUK5	31,469,622	48.6	47
CUK6	31,458,190	44.1	43
CUK7	31,464,764	44.5	43
CUK8	31,452,421	48.0	47
CUK9	31,477,028	52.1	51
CUK10	31,317,674	38.3	38
CUK11	31,459,366	45.5	45
CUK12	31,496,928	64.7	63

(A) Illumina Hiseq 2000 76bp reads were mapping with Smalt v0.6.1 (www.sanger.ac.uk/resources/software/smalt/) to the *L. infantum* reference genome JPCM5 (Rogers et al. 2011) for all chromosomes; those mapping to non-chromosomal contigs and kDNA regions are not shown.

Strain	Total #sites	Coverage	
		Mean	Median
CUK1	31,157,536	39.4	38
CUK2	31,158,746	46.5	45
CUK3	31,166,501	66.2	63
CUK4	31,162,767	53.0	50
CUK5	31,161,969	51.2	49
CUK6	31,162,099	51.0	49
CUK7	31,166,594	58.9	57
CUK8	31,161,902	49.7	48
CUK9	31,156,791	45.2	43
CUK10	31,159,872	45.5	43
CUK11	31,159,513	49.1	47
CUK12	31,165,994	53.3	51

(B) The same reads mapped to the *L. donovani* reference genome BPK282/0cl4 (Downing et al. 2011).
