# Supplementary material for: Genomic Confirmation of Hybridisation and Recent Inbreeding in a Vector-Isolated Leishmania Population
Source: PLoS Genet. 2014 Jan 16;10(1):e1004092. doi: 10.1371/journal.pgen.1004092 (PMC3894156; doi:10.1371/journal.pgen.1004092)
Supplement: Table S3 — Numbers of variable sites in each CUK strain. (PDF) [file pgen.1004092.s020.pdf]

Table S3. Numbers of variable sites in each CUK strain.

Strain	SNPs	Homozygous	Heterozygous
CUK1	11,091	7,857	3,234
CUK2	11,979	5,410	6,569
CUK3	10,961	7,668	3,293
CUK4	13,008	6,306	6,702
CUK5	11,797	6,641	5,156
CUK6	12,246	6,413	5,833
CUK7	11,515	5,119	6,396
CUK8	11,911	5,872	6,039
CUK9	11,208	5,354	5,854
CUK10	11,262	4,890	6,372
CUK11	11,937	6,397	5,540
CUK12	12,106	6,940	5,166

SNPs were called using the *L. infantum* JPCM5 reference genome: heterozygous alleles were determined using read depth coverage and quality metrics.
