# Supplementary material for: Genomic Confirmation of Hybridisation and Recent Inbreeding in a Vector-Isolated Leishmania Population
Source: PLoS Genet. 2014 Jan 16;10(1):e1004092. doi: 10.1371/journal.pgen.1004092 (PMC3894156; doi:10.1371/journal.pgen.1004092)
Supplement: Table S4 — The number of pairwise differences between CUK strains. (PDF) [file pgen.1004092.s021.pdf]

Table S4. The number of pair-wise differences between the CUK strains.

SNPs	CUK1	CUK2	CUK3	CUK4	CUK5	CUK6	CUK7	CUK8	CUK9	CUK10	CUK11	CUK12
CUK1												
CUK2	6,329											
CUK3	2,012	6,149										
CUK4	5,792	4,289	5,802									
CUK5	5,527	5,834	5,463	5,165								
CUK6	6,165	4,730	6,113	3,549	5,064							
CUK7	5,657	5,086	5,563	4,871	5,102	4,986						
CUK8	5,659	4,572	5,607	4,821	4,876	4,504	5,242					
CUK9	5,579	5,960	5,527	5,467	2,924	5,074	5,582	4,844				
CUK10	5,691	4,380	5,531	4,461	5,012	4,446	4,902	3,678	4,992			
CUK11	5,168	5,253	5,048	4,954	3,589	4,903	4,919	4,343	3,941	4,783		
CUK12	5,517	5,704	5,439	5,117	2,424	5,214	4,768	4,988	2,884	5,032	3,737	

Hom	CUK1	CUK2	CUK3	CUK4	CUK5	CUK6	CUK7	CUK8	CUK9	CUK10	CUK11	CUK12
CUK1												
CUK2	3,000											
CUK3	1,562	2,808										
CUK4	3,227	1,896	3,220									
CUK5	3,105	2,648	3,103	2,093								
CUK6	4,294	2,339	4,256	1,840	2,728							
CUK7	2,899	2,240	2,758	2,293	2,294	2,630						
CUK8	3,260	2,389	3,251	2,367	2,424	2,363	2,285					
CUK9	2,873	2,264	2,869	2,006	2,105	2,540	2,155	2,201				
CUK10	3,175	2,289	3,033	2,102	2,885	2,577	2,333	2,125	2,403			
CUK11	2,361	2,760	2,347	2,471	1,905	2,533	2,470	1,889	2,030	2,347		
CUK12	3,021	2,280	3,032	2,045	1,504	2,892	2,179	2,322	1,888	2,641	2,095	

Het	CUK1	CUK2	CUK3	CUK4	CUK5	CUK6	CUK7	CUK8	CUK9	CUK10	CUK11	CUK12
CUK1												
CUK2	3,329											
CUK3	450	3,341										
CUK4	2,565	2,393	2,582									
CUK5	2,422	3,186	2,360	3,072								
CUK6	1,871	2,391	1,857	1,709	2,336							
CUK7	2,758	2,846	2,805	2,578	2,808	2,356						
CUK8	2,399	2,183	2,356	2,454	2,452	2,141	2,957					
CUK9	2,706	3,696	2,658	3,461	819	2,534	3,427	2,643				
CUK10	2,516	2,091	2,498	2,359	2,127	1,869	2,569	1,553	2,589			
CUK11	2,807	2,493	2,701	2,483	1,684	2,370	2,449	2,454	1,911	2,436		
CUK12	2,496	3,424	2,407	3,072	920	2,322	2,589	2,666	996	2,391	1,642	

The number of single nucleotide differences between the 12 CUK strains (shown for the entire region over which SNPs could be called; 29.5 Mb of the 32 Mb haploid genome). **SNPs**, the total number of SNPs between strain pairs; **Hom**, the number of homozygous SNPs; **Het**, the number of heterozygous ones.
